# Global Analysis of the Sporulation Pathway of *Clostridium difficile*

**DOI:** 10.1371/journal.pgen.1003660

**Published:** 2013-08-08

**Authors:** Kelly A. Fimlaid, Jeffrey P. Bond, Kristin C. Schutz, Emily E. Putnam, Jacqueline M. Leung, Trevor D. Lawley, Aimee Shen

**Affiliations:** 1 Department of Microbiology and Molecular Genetics, University of Vermont, Burlington, Vermont, United States of America; 2 Program in Cellular, Molecular & Biomedical Sciences, University of Vermont, Burlington, Vermont, United States of America; 3 Microbial Pathogenesis Laboratory, The Wellcome Trust Sanger Institute, Wellcome Trust Genome Campus, Hinxton, United Kingdom; University of Geneva Medical School, Switzerland

## Abstract

The Gram-positive, spore-forming pathogen *Clostridium difficile* is the leading definable cause of healthcare-associated diarrhea worldwide. *C. difficile* infections are difficult to treat because of their frequent recurrence, which can cause life-threatening complications such as pseudomembranous colitis. The spores of *C. difficile* are responsible for these high rates of recurrence, since they are the major transmissive form of the organism and resistant to antibiotics and many disinfectants. Despite the importance of spores to the pathogenesis of *C. difficile*, little is known about their composition or formation. Based on studies in *Bacillus subtilis* and other *Clostridium* spp., the sigma factors σ^F^, σ^E^, σ^G^, and σ^K^ are predicted to control the transcription of genes required for sporulation, although their specific functions vary depending on the organism. In order to determine the roles of σ^F^, σ^E^, σ^G^, and σ^K^ in regulating *C. difficile* sporulation, we generated loss-of-function mutations in genes encoding these sporulation sigma factors and performed RNA-Sequencing to identify specific sigma factor-dependent genes. This analysis identified 224 genes whose expression was collectively activated by sporulation sigma factors: 183 were σ^F^-dependent, 169 were σ^E^-dependent, 34 were σ^G^-dependent, and 31 were σ^K^-dependent. In contrast with *B. subtilis*, *C. difficile* σ^E^ was dispensable for σ^G^ activation, σ^G^ was dispensable for σ^K^ activation, and σ^F^ was required for post-translationally activating σ^G^. Collectively, these results provide the first genome-wide transcriptional analysis of genes induced by specific sporulation sigma factors in the Clostridia and highlight that diverse mechanisms regulate sporulation sigma factor activity in the Firmicutes.

## Author Summary

*C. difficile* is the leading cause of healthcare-associated infectious diarrhea in the United States in large part because of its ability to form spores. Since spores are resistant to most disinfectants and antibiotics, *C. difficile* infections frequently recur and are easily spread. Despite the importance of spores to *C. difficile* transmission, little is known about how spores are made. We set out to address this question by generating *C. difficile* mutants lacking regulatory factors required for sporulation and identifying genes that are regulated by these factors during spore formation using whole-genome RNA-Sequencing. We determined that the regulatory pathway controlling sporulation in *C. difficile* differs from related *Clostridium* species and the non-pathogenic model spore-former *Bacillus subtilis* and identified 314 genes that are induced during *C. difficile* spore development. Collectively, our study provides a framework for identifying *C. difficile* gene products that are essential for spore formation. Further characterization of these gene products may lead to the identification of diagnostic biomarkers and the development of new therapeutics.

## Introduction

*Clostridium difficile* is a Gram-positive, spore-forming, obligate anaerobe that causes gastrointestinal diseases including diarrhea, pseudomembranous colitis, and toxic megacolon [1]–[3]. *C. difficile* infections and *C. difficile*-related deaths have risen dramatically in the past decade, increasing the financial burden on health care systems [4]–[7]. While *C. difficile* is best known for causing hospital-acquired antibiotic-associated infections, recent epidemiologic studies indicate that community-acquired *C. difficile* infections are increasingly more common and associated with significant morbidity [6], [7]. A key element to the success of *C. difficile* as a pathogen is its ability to produce spores. Spores are resistant to most disinfectants and antibiotics, making them difficult to eliminate both from infected humans and the environment [1], [2], [8]. As a result, *C. difficile* spores disseminate readily from person to person and cause high rates of recurrent infections, which can lead to serious illness or even death [1]–[3], [9].

Although spores are critical to the pathogenesis of *C. difficile*, their composition and formation remain poorly characterized. Less than 25% of the spore coat proteins identified in the well-characterized spore-former *Bacillus subtilis* have homologs in *C. difficile* [10]. In contrast, the regulatory proteins that control spore coat gene expression and other sporulation events in *B. subtilis* are conserved in *C. difficile* and all other spore-forming Firmicutes [10]–[13]. These include the master sporulation transcriptional regulator, Spo0A, and the sporulation sigma factors σ^F^, σ^E^, σ^G^, and σ^K^.

In *B. subtilis* the sporulation sigma factors function at discrete stages during spore development to couple changes in gene expression with specific morphological changes in the cell [14]–[16]. The morphological changes begin with the formation of a polar septum, which creates two compartments, the mother cell and the forespore. The mother cell engulfs the forespore and guides the assembly of the spore until it lyses once spore maturation is complete. By coupling these developmental changes to the sequential activation of compartment-specific sporulation sigma factors, the mother cell and forespore produce divergent transcriptional profiles that coordinately lead to the formation of a dormant spore [16].

Sporulation gene transcription in *B. subtilis* begins with the activation of the transcription factor Spo0A, which in turn activates early sporulation gene transcription, such as the genes encoding the early sigma factors σ^F^ and σ^E^. σ^F^ is initially held inactive by an anti-σ factor and only undergoes activation after septum formation is complete; this mode of regulation couples σ^F^ activation in the forespore to a morphological event [17], [18]. Active σ^F^ induces the transcription of genes whose products mediate cleavage of an inhibitory pro-peptide from σ^E^ in the mother cell via trans-septum signaling [19]. Active σ^E^ induces the transcription of genes whose products lead to the activation of the late sporulation sigma factor σ^G^ in the forespore, which occurs during or after engulfment [20], [21]. Activated σ^G^ in the forespore subsequently induces the expression of genes whose products proteolytically activate σ^K^ in the mother cell via trans-septum signaling [22]. Notably, the activity of each sigma factor relies on the activation of the preceding sigma factor [11], [14]–[16], [23]. As a result, the sigma factors operate in a sequential, “criss-cross” manner and collectively control the expression of hundreds of genes during sporulation [24]–[26].

The regulatory pathway controlling sporulation sigma factor activation in *B. subtilis* is thought to be conserved across endospore-forming bacteria, since all four sigma factors are conserved [11], [12]. However, a growing body of work in the Clostridia suggests that diverse pathways regulate sporulation sigma factor activity in the Firmicutes. In *C. perfringens*, a *sigG^−^* mutant still produces cleaved σ^K^, suggesting that σ^G^ does not control the proteolytic activation of σ^K^ as it does in *B. subtilis* [27]. Furthermore, a *C. perfringens sigK^−^* mutant exhibits a phenotype more severe than a *B. subtilis sigE^−^* mutant in that it fails to initiate asymmetric division or produce σ^E^ [28], suggesting that in *C. perfringens* σ^K^ functions upstream of σ^E^. Indeed, *C. perfringens* σ^E^ and σ^K^ have been suggested to be dependent on each other for full activity, in contrast with *B. subtilis* [28]. A similar early sporulation defect has been observed in a *sigK^−^* mutant of *C. botulinum*, which also exhibits reduced expression of early sporulation genes *spo0A* and *sigF* [29]. In contrast with *B. subtilis* and *C. perfringens*, however, a *C. acetobutylicum sigF^−^* mutant does not initiate asymmetric division [30], and a *sigE^−^* mutant fails to complete asymmetric division [31]. In addition, a *C. acetobutylicum sigE^−^* mutant produces wildtype levels of σ^G^ [31] in contrast with *B. subtilis*, and a *sigG^−^* mutant exhibits elongated forespores and pleiotropic defects in coat and cortex formation [31].

To determine how these sporulation sigma factors regulate sporulation in *C. difficile*, we constructed mutations in the genes encoding the sporulation transcription factor Spo0A and the sigma factors σ^F^, σ^E^, σ^G^, and σ^K^ and determined the transcriptional profiles of these mutants using RNA-Sequencing (RNA-Seq). The transcriptional analyses, combined with cytological characterization of the sigma factor mutants, suggest that divergent mechanisms regulate the activity of σ^G^ and σ^K^ in *C. difficile* relative to *B. subtilis* and other *Clostridium* spp. In addition, these analyses have identified a set of 314 genes that are upregulated during sporulation in a Spo0A-, σ^F^-, σ^E^-, σ^G^-, and/or σ^K^-dependent manner. These sporulation-induced genes provide a framework for identifying and characterizing *C. difficile* spore proteins that may have diagnostic or therapeutic utility.

## Results

### *C. difficile* sporulation sigma factors are essential for mature spore formation

In order to identify genes that are regulated by the sporulation-specific sigma factors, we used a modified TargeTron gene knockout system to disrupt the genes encoding σ^F^, σ^E^, σ^G^, and σ^K^ in *C. difficile* [32]. This system uses a group II intron to insert an erythromycin resistance cassette into the target gene (Figure S1A). JIR8094 [33], an erythromycin-sensitive derivative of the sequenced *C. difficile* strain 630 [34], was used as the parental strain. As a control, we also constructed a targeted disruption in *spo0A*, which encodes the master regulator of sporulation Spo0A [35], [36]. Colony PCR of the intron-disrupted mutants confirmed the expected size change resulting from the intron insertion into the *spo0A*, *sigF*, *sigE*, *sigG*, and *sigK* genes (Figure S1B).

**Figure 1:**
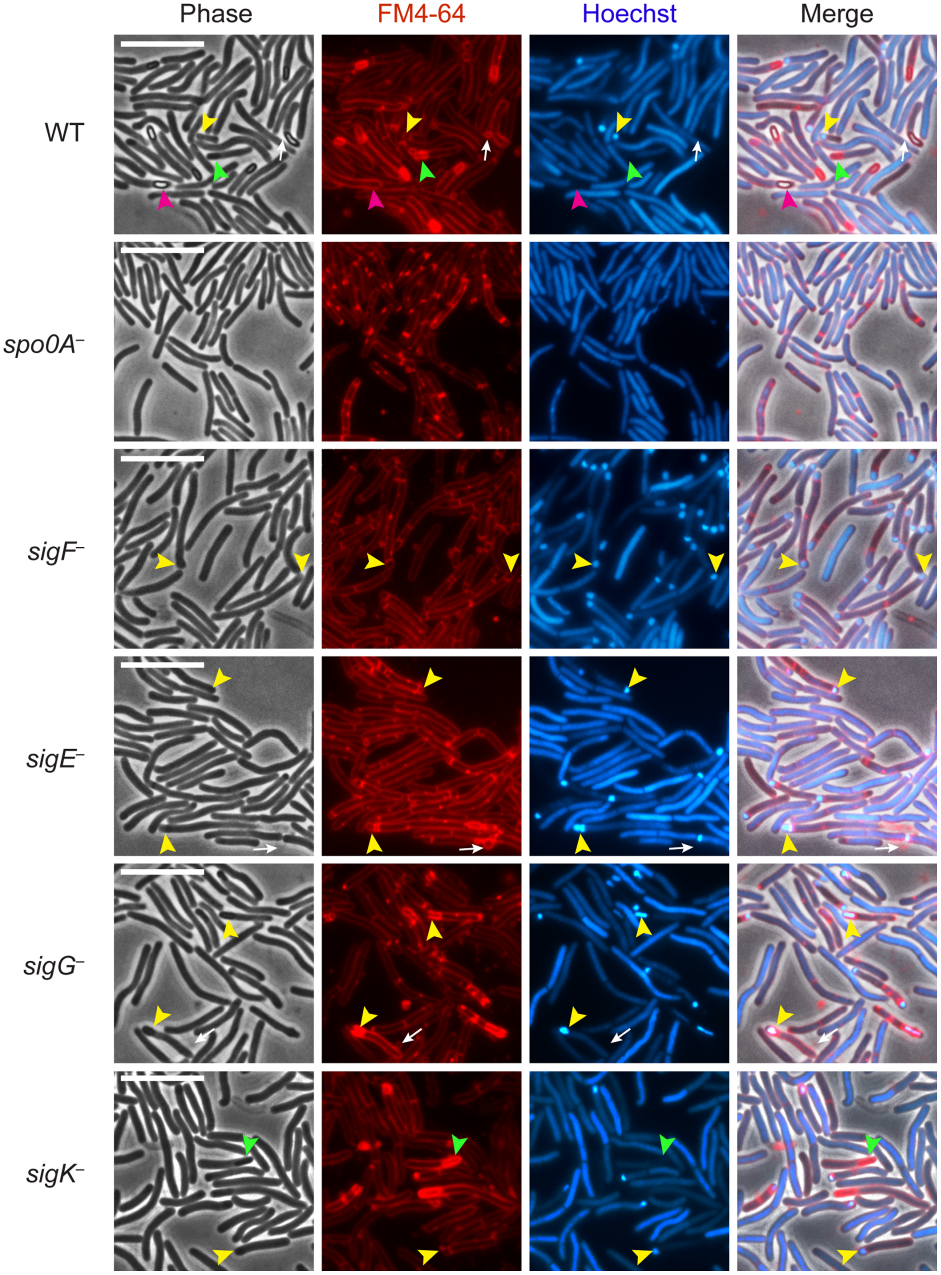
*C. difficile sigF* ^−^ sigE^−^, *sigG^−^*, and *sigK^−^* sigma factor mutants are defective in mature spore formation. *C. difficile* strains wildtype (WT), *spo0A^−^*, *sigF^−^*, *sigE^−^*, *sigG^−^*, and *sigK^−^* were grown on sporulation media for 18 hrs and evaluated by live phase-contrast and fluorescence microscopy. Phase contrast, FM4-64 membrane staining (red), nucleoid staining with Hoechst (blue), and the merge of these images are shown for each strain. Yellow arrowheads indicate forespore compartments that stain with FM4-64 and Hoechst; green arrowheads indicate phase-dark, immature forespores that stain with FM4-64 but not Hoechst; and pink arrowheads indicate phase-bright mature spores that exclude both the FM4-64 and Hoechst stains. Phase-bright spores were not observed in any of the mutant strains. Circular vesicles that were labeled by FM4-64, but not visible by phase-contrast microscopy (white arrows) were frequently observed in cultures grown on sporulation media, even in the *spo0A^−^* mutant (data not shown). Scale bars represent 10 µm.

To determine the effect of blocking sigma factor production on sporulation, the mutants were induced to sporulate on solid sporulation media and visualized by phase contrast microscopy [37]. It should be noted that sporulation is asynchronous in this assay, and the extent and timing of sporulation exhibits variability even between biological replicates (Figure S2). Nevertheless, after 18 hrs of growth, sufficient numbers of cells have initiated sporulation to detect the production of immature phase-dark forespores and mature phase-bright spores in the wildtype strain (Figure 1 and S2). In contrast, *spo0A* ^−^, *sigF* ^−^, *sigE* ^−^, *sigG* ^−^, and *sigK* ^−^ cultures failed to produce phase-bright spores (Figure 1). No phase-dark or phase-bright forespores were observed in the *spo0A^−^*, *sigF^−^*, or *sigE^−^* strains, suggesting a block early in sporulation.

Analysis of live, sporulating cultures with the lipophilic dye FM4-64 (to stain mother cell and forespore membranes) and Hoechst 33342 (to stain cell nucleoids) revealed polar septum formation in wild type and the sigma factor mutants but not in the *spo0A^−^* mutant (Figure 1). This result was consistent with the observation that Spo0A is necessary to induce the sporulation pathway in *C. difficile* [35], [36]. Overall, the proportion of sporulating cells detected by membrane and DNA staining in the culture was 25%, 41%, 24%, 26%, and 18% for wildtype, *sigF^−^*, *sigE^−^*, *sigG^−^*, and *sigK^−^*, respectively, as indicated by the presence of a polar septum, immature forespore compartment, or mature forespore (Table S1). Wildtype cultures contained a heterogenous population of sporulating cells at discrete stages of sporulation: 28% of sporulating cells exhibited intense DNA staining of an FM4-64-labeled forespore compartment (yellow arrows, Figure 1, Table S1); 28% showed phase-dark forespores that stained with both FM4-64 and Hoechst (Table S1), 28% exhibited phase-dark forespores that stained intensely with FM4-64 but not Hoechst (green arrows, Figure 1, Table S1), and 16% contained a phase-bright forespore that failed to be stained with either FM4-64 or Hoechst (pink arrows, Figure 1, Table S1). In contrast, *sigF^−^* and *sigE^−^* sporulating cells were arrested at the asymmetric division stage, with 95% and 92% of sporulating cells, respectively, exhibiting intense DNA staining of an FM4-64-labeled forespore compartment (yellow arrows, Figure 1, Table S1). The *sigG^−^* mutant strain was arrested at the phase-dark forespore stage, with 69% of sporulating cells exhibiting intense forespore membrane and nucleoid staining (yellow arrows, Figure 1, Table S1). While only 4% of the *sigG^−^* cells were observed to produce forespores that stained only with FM4-64, 44% of sporulating *sigK^−^* cells were captured at this stage of sporulation, a phenotype that was also observed in wildtype (green arrows, Figure 1, Table S1). Taken together, these results indicate that all four sporulation sigma factors are required to complete spore formation and suggest that σ^G^ is necessary to complete the stage of sporulation development required to exclude the Hoechst dye from staining the forespore chromosome. The results are also consistent with studies investigating *B. subtilis* forespore development, which indicate that nucleic acid stains are excluded earlier than membrane stains during spore development [38]–[40].

**Figure 2:**
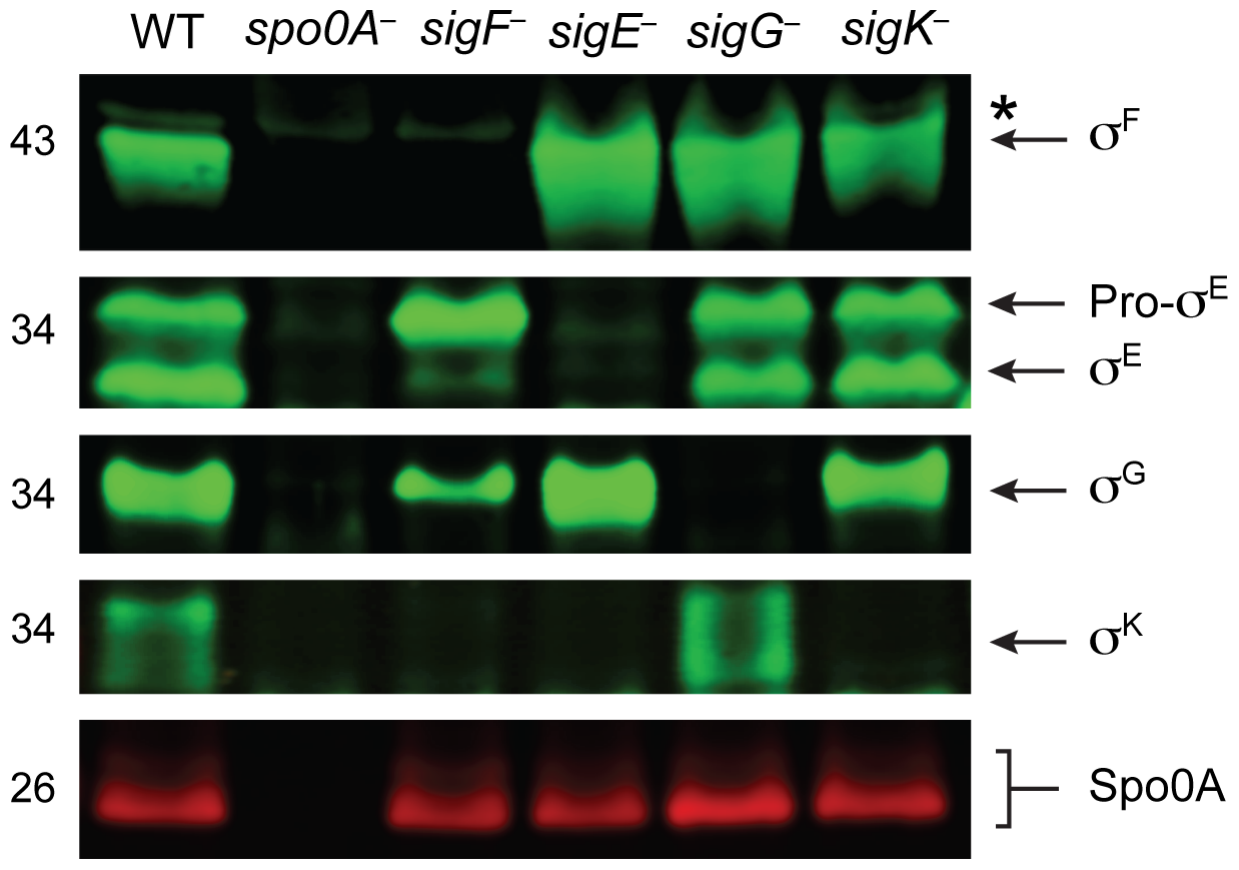
Analysis of sporulation sigma factor production in sporulation sigma factor mutants. Western blot analyses of σ^F^, σ^E^, σ^G^, σ^K^, and Spo0A, respectively, in wildtype (WT), *spo0A^−^*, *sigF^−^*, *sigE^−^*, *sigG^−^*, and *sigK^−^* strains grown for 18 hr on sporulation media using antibodies raised against Spo0A and the sporulation sigma factors. The * demarcates a non-specific band observed in the *sigF^−^* and *spo0A^−^* mutants. Pro-σ^E^ indicates full-length σ^E^ prior to pro-peptide removal.

To confirm that the gene disruptions prevented sigma factor production in each of the respective sigma factor mutants, we performed Western blot analyses using antibodies raised against *C. difficile* sigma factors. Similar to *B. subtilis*, Spo0A was required for the production of all the factors, and σ^F^ was observed in the *sigE^−^*, *sigG^−^*, and *sigK^−^* strains at wildtype levels (Figure 2, [41]). σ^E^ was detected in both its pro- and cleaved form in wildtype, *sigG^−^* and *sigK^−^* strains, whereas the majority of σ^E^ was unprocessed in the *sigF^−^* strain (Figure 2). This result slightly deviates from the *B. subtilis* model, where pro-σ^E^ processing is completely abrogated in a *B. subtilis sigF* ^−^ strain [42]. In contrast, a *C. perfringens sigF^−^* mutant fails to produce pro-σ^E^ altogether [27], and σ^E^ processing has not been demonstrated in *C. acetobutylicum* [31]. σ^K^ was present in wildtype and *sigG^−^* mutant strains but absent in the *sigF* ^−^ and *sigE^−^* strains (Figure 2), analogous to observations in *B. subtilis* where σ^E^ is required for *sigK* expression. A *C. perfringens sigE^−^* strain in contrast produces low amounts of σ^K^ [28]. Consistent with the observation that *C. difficile* σ^K^ lacks an N-terminal pro-peptide [43], no processing of σ^K^ was observed in wildtype *C. difficile* (Figure 2), even though σ^K^ undergoes proteolytic activation in *B. subtilis* and *C. perfringens* [27]. σ^G^ was detected in the *C. difficile sigF^−^*, *sigE* ^−^ and *sigK^−^* mutants (Figure 2) in contrast with studies of other endospore-forming bacteria, where σ^G^ activity and auto-activation of *sigG* transcription is partially dependent on σ^E^ in *B. subtilis* [44]–[46], and σ^G^ production depends on σ^F^ in *C. perfringens* and *C. acetobutylicum* [27], [30].

**Figure 3:**
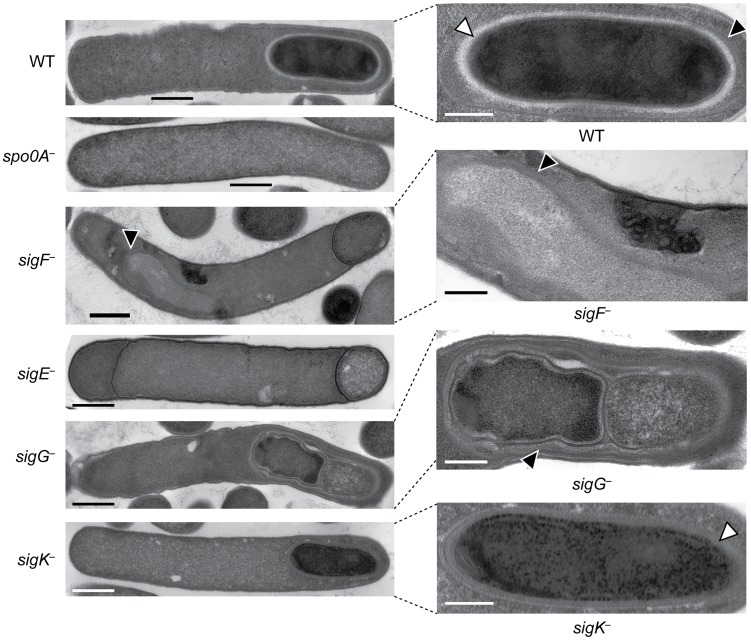
*C. difficile sigF* ^−^, *sigE^−^*, *sigG^−^*, and *sigK^−^* mutants are arrested at different stages of spore formation. Transmission electron microscopy (TEM) of wildtype, *spo0A^−^*, *sigF* ***^−^***, *sigE* ***^−^***, *sigG* ***^−^***, and *sigK* ***^−^*** strains at 18 hrs of growth on sporulation media. The forespore regions of wild type (WT), *sigG^−^*, and *sigK^−^* strains, and an electron-translucent region within the *sigF^−^* mutant mother cell cytosol, are shown on the right. Black triangles indicate regions that resemble coat layers, while white triangles indicate regions consistent with cortex. Scale bars represent 500 nm. Inset scale bars represent 250 nm.

We next performed transmission electron microscopy (TEM) to identify the precise developmental stage at which each sigma factor mutant was stalled. Cortex and coat layers were present on forespores in wildtype sporulating cells, while the *spo0A^−^* mutant exhibited no signs of spore formation (Figure 3). The *sigF* ^−^ mutant failed to progress beyond asymmetric division (Figure 3), similar to a *B. subtilis sigF^−^* mutant [47] but in contrast with a *C. acetobutylicum sigF^−^* mutant which does not initiate asymmetric division [30]. Nevertheless, unlike *B. subtilis*, a more electron-translucent region in the mother cell cytosol surrounded by electron dense layers was observed in some *sigF^−^* mutant cells; this region resembled mislocalized spore coat ([37], Figure 3). The *C. difficile sigE^−^* strain was arrested at the asymmetric division stage similar to the *sigF^−^* mutant, although electron-translucent regions surrounded by coat-like layers were not observed in any *sigE^−^* cell analyzed. The *C. difficile sigE^−^* mutant phenotype resembled the phenotype of *sigE^−^* mutants of *B. subtilis* [47] and *C. perfringens* [28], with frequent observations of disporic cells or cells with multiple septa at one pole (Figures 1 and 3). This observation was in contrast with a *C. acetobutylicum sigE^−^* mutant, which does not complete asymmetric division [31]. The *C. difficile sigG^−^* mutant produced forespores lacking an apparent cortex layer, similar to *B. subtilis* [21], [44]; however, unlike *B. subtilis*, the forespores were surrounded by thin layers that resembled the spore coat layers visible in wildtype cells (Figure 3). In addition, the *C. difficile sigG^−^* mutant exhibited pleiotropic defects including forespore ruffling, incomplete membrane fission during engulfment, and a septated forespore compartment (Figures 3 and S3). Quantitation of the prevalence of each phenotype revealed that forespore ruffling, incomplete engulfment, and a septated forespore compartment were observed in 98, 87 and 21% of *sigG^−^* cells, respectively (Figure S3). Lastly, the *C. difficile sigK* ^−^ mutant produced forespores surrounded by a layer that resembled the cortex layer of wildtype, but no coat layers were apparent (Figure 3). This phenotype was more similar to a *B. subtilis sigK^−^* mutant, which lacks both cortex and coat [22], than *C. perfringens*, which fails to initiate polar septum formation [28].

### Plasmid complementation rescues the sporulation defects of *sigF^−^*, *sigE* ^−^ *sigG* ^−^, and *sigK* ^−^ mutants

To validate that the observed mutant phenotypes were due to the targeted insertions, we complemented the mutant strains by expressing a wildtype copy of the gene encoding the corresponding sigma factor from a plasmid. We used either the pMTL83151 or pMTL84151 multicopy plasmids [48] to express the complementing genes or operons from their native promoters. The complementation constructs all restored production of phase-bright spores when expressed in their respective mutant backgrounds (Figure S4A), although phase-bright spore formation by the *sigK* complementation strain was delayed relative to wildtype. Western blot analysis further confirmed that the complementation constructs restored production of the respective sigma factor to wildtype levels (Figure S4B). TEM analysis revealed that all four complementation constructs restored coat and cortex formation to their respective mutant strains (Figure S5). Heat resistance assays to measure complementation strain sporulation efficiency revealed that the *sigF* ^−^ and *sigE* ^−^constructs fully complemented heat resistance relative to wildtype and that the *sigG* ^−^ and *sigK* ^−^ constructs partially complemented heat resistance (70 and 23%, respectively, Figure S4C).

### RNA-Seq analysis reveals the regulatory relationships between *C. difficile* sporulation sigma factors

While these analyses showed that σ^F^, σ^E^, σ^G^, and σ^K^ were all required for mature spore formation, they did not reveal which genes were being misregulated in the sporulation sigma factor mutants to produce their respective sporulation defects. To identify these genes and gain insight into the regulatory network controlling sporulation sigma factor activity, we used RNA-Sequencing (RNA-Seq) to transcriptionally profile our sporulation mutants and wild type during sporulation. Three biological replicates of wildtype, *spo0A* ^−^, and sporulation sigma factor mutant strains were grown on sporulation media (Figure S2), and RNA was isolated. Following DNase-treatment, ribosomal RNA depletion and reverse transcription, Illumina-based RNA-Seq was used to determine the complete transcriptome of wildtype *C. difficile* and the sporulation mutants. Genome coverage and sequencing counts for each strain and replicate can be found in Table S2.

The DeSeq variance analysis package [49] was used to identify genes that were downregulated by ≥4-fold with an adjusted p-value of ≤0.05 in the *spo0A* ^−^ strain relative to wild type. This pair-wise analysis identified 276 genes as being Spo0A-dependent (Table S3). Consistent with the role of Spo0A as the master regulator of sporulation, 65 of these genes were predicted to be involved in sporulation (Table S4) [11], [50]–[52]. Six of these Spo0A-dependent genes were recently identified as encoding components of the *C. difficile* spore coat [50], [53], and 36 sporulation-related genes (Table S4) were shown to depend on σ^H^, the stationary phase sigma factor that induces *spo0A* transcription in *C. difficile* [51] and *B. subtilis* [54]. σ^F^-, σ^E^-, σ^G^-, and σ^K^-dependent genes were identified by comparing the transcriptional profiles of the *sigF^−^*, *sigE^−^*, *sigG^−^*, and *sigK^−^* strains to wild type, respectively, using the same parameters as above. This analysis identified 183 genes as being dependent on σ^F^ for their expression (Table S5). One hundred eighteen of these σ^F^-dependent genes were also σ^E^-dependent (Table S6), indicating that σ^E^ has some activity in a *sigF^−^* mutant consistent with the reduced levels of cleaved σ^E^ being detected by Western blot (Figure 2); 29 of the σ^F^-dependent genes formed a separate subset of genes that were also σ^G^-dependent but σ^E^-independent. Indeed, the majority of the 34 σ^G^-dependent genes identified in this analysis were not dependent on σ^E^ for their expression (Table S7), since only four of the σ^G^-regulated genes were also σ^E^-regulated. Notably, none of the genes identified as being σ^G^-dependent required σ^K^ for their expression (Table S8), suggesting that the σ^G^ produced in the *sigE^−^* and *sigK^−^* mutants is active (Figure 2). This result differs from the *B. subtilis* model where σ^E^ is needed to fully activate σ^G^ function [20], [21], [46], [55], [56].

**Figure 4:**
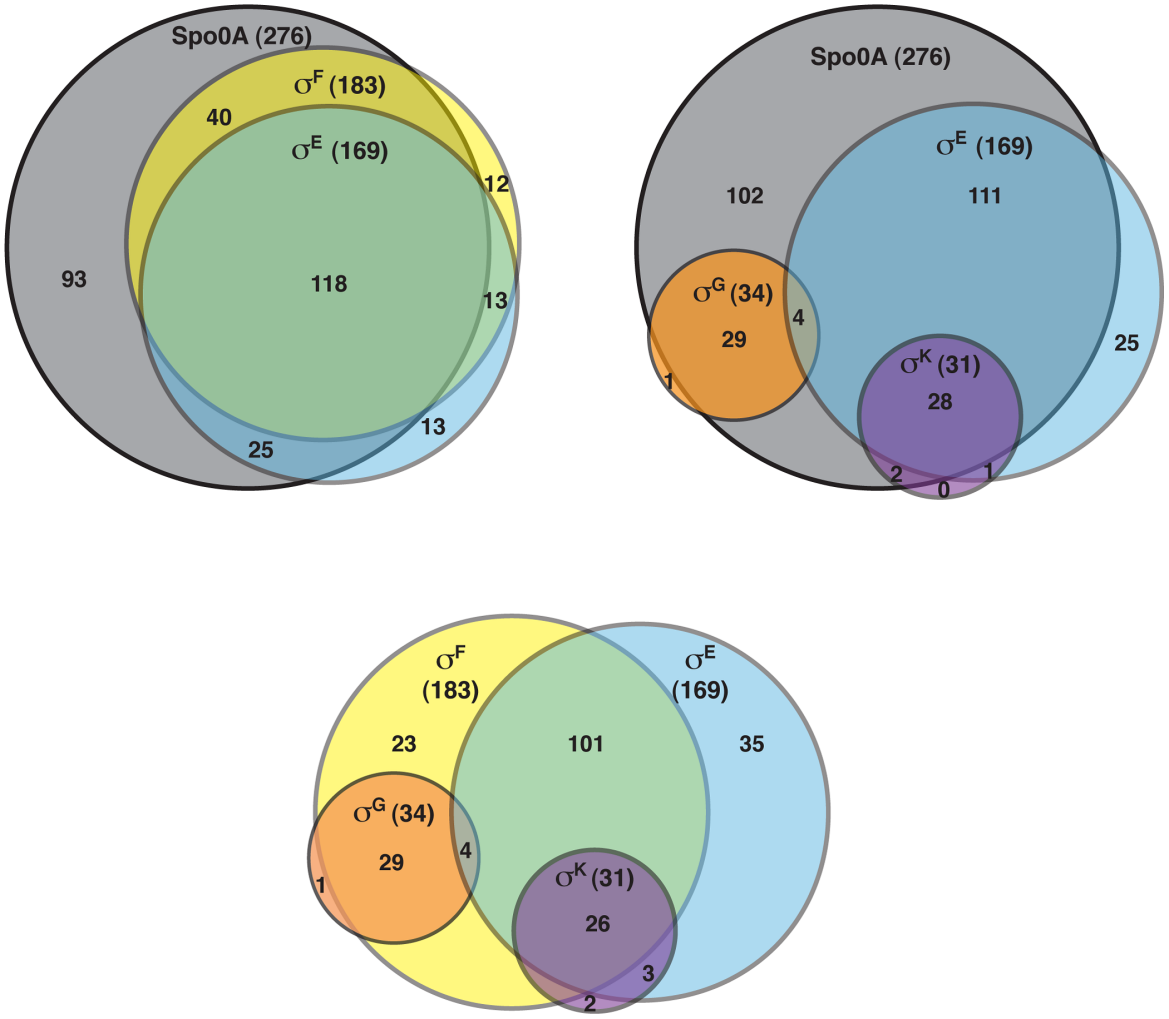
Venn diagram of genes identified as being dependent on either Spo0A, σ^F^-, σ^E^-, σ^G^-, and/or σ^K^-dependent as determined by RNA-Seq. Genes were defined as being dependent on their respective sigma factor for expression if their transcript levels were decreased by ≥4-fold with an adjusted p-value of ≤0.05 in the mutant strains relative to wild type. The genes identified in these analyses are listed in Tables S3, S5, S6, S7 and S8.

Of the 169 genes that depended on σ^E^ for their expression (Table S6), 85% and 78% of these genes were dependent on Spo0A and σ^F^, respectively (Figure 4). The expression of 29 of these genes was also σ^K^-dependent (Table S5). Indeed the majority of the 31 σ^K^-dependent genes were σ^E^-dependent (Table S8; Figure 4), consistent with σ^E^ being required for σ^K^ production (Figure 2). In contrast, as described earlier, no overlap was observed between σ^G^- and σ^K^-dependent genes (Figure 4). Taken together, the RNA-Seq analyses suggested that (1) a small subset of σ^F^-dependent genes are neither σ^E^, σ^G^, nor σ^K^-dependent; (2) σ^E^ activity depends on Spo0A and σ^F^ but not σ^G^ or σ^K^; (3) σ^K^ activity depends on Spo0A, σ^F^, and σ^E^ but not σ^G^, and (4) σ^G^ activity depends on Spo0A and σ^F^ but not σ^E^ or σ^K^. The latter two findings differ from the *B. subtilis* model, where the σ^K^-dependent genes are also σ^G^-dependent because σ^K^ activity depends on σ^G^ [11], [15], [22], and σ^G^-dependent genes are σ^E^-dependent because full activation of σ^G^ requires σ^E^ [20], [21], [46], [55], [56].

**Figure 5:**
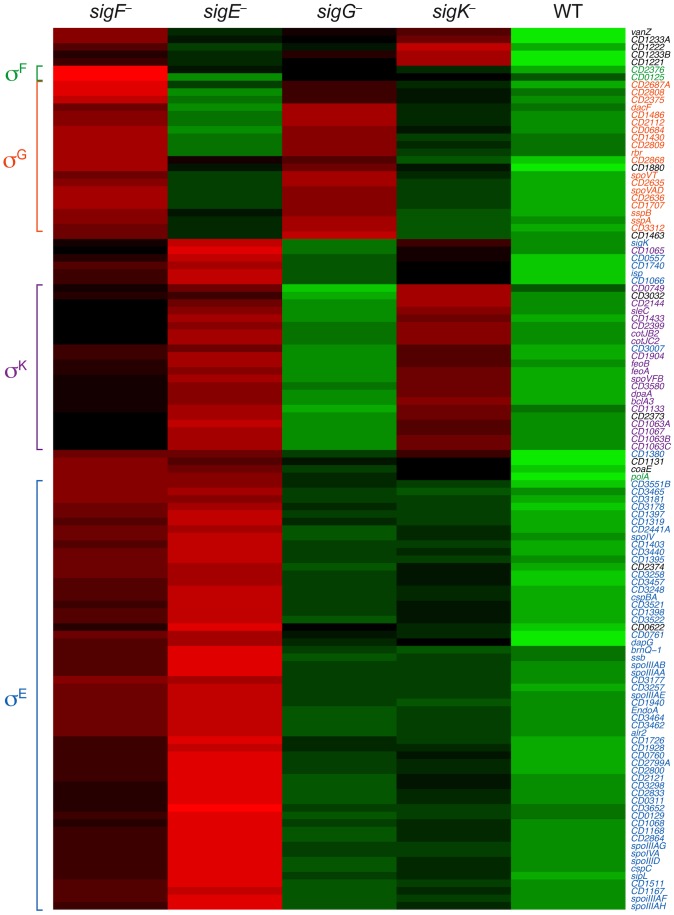
Comparison of Spo0A-dependent gene expression in wildtype and sporulation sigma factor mutants. Heat map representation of the genes that were downregulated by ≥4-fold with an adjusted p-value of ≤10^−5^ in the *spo0A* ^−^ strain relative to wild type. Expression levels of these genes in wildtype (WT), *sigF^−^*, *sigE^−^*, *sigG^−^*, and *sigK^−^* strains relative to *spo0A^−^* were centered, scaled, and mapped to a red-green color scale, with green indicating that the gene was upregulated in the strain relative to the other strains, and red indicating that the gene was downregulated relative to the centered expression level. σ^E^-regulated genes (blue), σ^K^-regulated genes (purple), σ^G^-regulated genes (orange), σ^F^-dependent genes (green) are colored as indicated, and clusters of coordinately regulated genes are bracketed. Genes colored in black were identified as depending only on Spo0A for expression.

To visually represent the differences in gene expression profiles between the sigma factor mutants and wild type, we generated a heat map for genes downregulated by ≥4-fold with an adjusted p-value of ≤10^−5^ in the *spo0A* ^−^ strain relative to wild type. The expression levels of wild type and the sigma factor mutants relative to *spo0A^−^* strain were centered, scaled, and mapped to a red-green color scale. The heat map revealed a cluster of genes that was poorly expressed in the *sigE^−^* mutant relative to the wildtype, *sigG^−^,* and *sigK^−^* strains; these genes were also expressed at reduced levels in the *sigF^−^* mutant (Figure 5) and were primarily σ^E^-dependent (Table S4). A separate cluster of genes was downregulated in both the *sigK^−^* and *sigE^−^* mutants relative to the wildtype and *sigG^−^* strains (Figure 5); these genes were all identified as σ^K^-dependent genes (Table S5). Another discrete cluster of genes was downregulated in the *sigG^−^* and *sigF^−^* strains relative to the wildtype, *sigE* ^−^, and *sigK^−^* strains (Figure 5); again, most of these genes were identified as σ^G^-dependent genes, although two genes were σ^F^-dependent but not σ^G^-dependent (Tables S5 and S7). Thus, identification of variably expressed genes between the strains confirmed the findings of our earlier pair-wise analyses: σ^F^-dependent genes were largely Spo0A-dependent, σ^E^-dependent genes were largely σ^F^-dependent, σ^K^-dependent genes were σ^E^-dependent, and σ^G^-dependent genes were σ^F^-dependent but not σ^E^- or σ^K^-dependent. These results support a model where (1) σ^F^ controls the activation of both σ^E^ and σ^G^, (2) σ^E^ induces the production and activation of σ^K^, and (3) σ^E^ and σ^K^ are dispensable for σ^G^ activation. Alternative statistical models were also employed to validate these findings (see Text S1 and Figures S6 and S7).

### Quantitative RT-PCR validates the RNA-Seq Data

**Figure 6:**
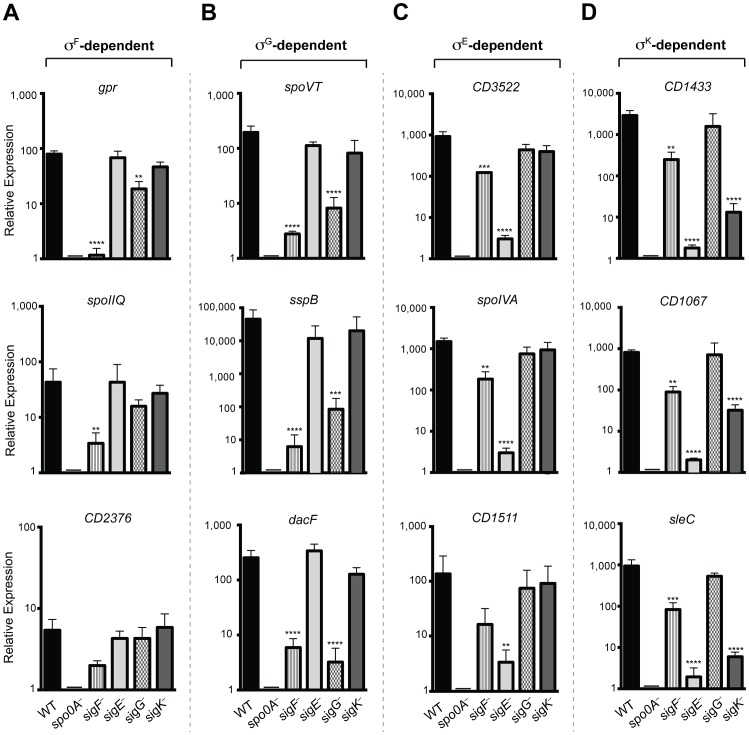
qRT-PCR validation of RNA-Seq transcriptional profiling. Transcript levels for three genes that were determined to be dependent on σ^F^, σ^E^, σ^G^, and/or σ^K^ for expression as measured by qRT-PCR on three biological replicates. Samples were distinct from those used for RNA-Seq. (A) σ^F^-dependent genes included *gpr*, *CD0125* (*spoIIQ* [13]) and *CD2376*. (B) σ^G^-dependent genes included *spoVT sspB*, and *dacF*. (C) σ^E^-dependent genes included *CD3522*, *spoIVA*, and *CD1511*. (D) σ^K^-dependent genes included *CD1433*, *CD1067* and *sleC*. cDNA was produced from RNA samples harvested from wildtype (WT), *spo0A^−^*, *sigF^−^*, *sigE^−^*, *sigG^−^*, and *sigK^−^* strains grown on sporulation media for 18 hrs. Data represent the averages of three biological replicates and at least two technical replicates. Transcripts were calculated relative to *spo0A^−^* and normalized to *rpoB* (housekeeping gene). Error bars indicate the standard error of the mean. Statistically significant changes in transcript levels were determined relative to WT and represented by adjusted p-values determined by a Dunnett's one-way ANOVA. ****p<0.0001, ***p<0.001, **p<0.01. *CD2376* transcript levels were ∼3-fold reduced in the *sigF^−^* mutant relative to wild type (p = 0.065).

To validate the RNA-Seq data, we isolated RNA from three separately prepared biological replicates of wildtype, *spo0A^−^*, *sigF^−^*, *sigE^−^*, *sigG^−^*, and *sigK^−^* strains grown on sporulation media for 18 hrs. RNA was reverse transcribed and quantitative RT-PCR (qRT-PCR) was performed using primers specific for three genes within each of the sigma factor-dependent transcriptomes. Gene expression levels in the wildtype and the sigma factor mutant strains relative to *spo0A^−^* were determined by comparative CT analysis normalized to the housekeeping gene *rpoB*. These analyses confirmed that the transcript levels of the σ^F^-dependent gene *gpr* was reduced by >50-fold (p<0.0001) in the *sigF* ^−^ mutant relative to wild type, and reduced in the *sigG* ^−^ mutant by ∼4 fold (p<0.01); *gpr* expression was not affected in the *sigE* ^−^ and *sigK* ^−^ mutants. *cd0125* (*spoIIQ*, [13]) transcription was reduced by >10-fold in the *sigF* ^−^ mutant relative to wild type (p<0.01), but no reduction in transcript levels was observed in *sigE* ^−^, *sigG* ^−^, and *sigK* ^−^ mutants (Figure 6A). Transcription of *cd2376* was reduced by 3-fold in the *sigF* ^−^ relative to wild type (Figure 6A). Although this correlation was not statistically significant, it approached statistical significance (p = 0.065) (Figure 6A); this result is likely due to the low number of overall *cd2376* transcripts present in the samples. Transcript levels of the σ^G^-dependent genes *spoVT*, *sspB*, and *dacF* showed significant reductions in the *sigG* ^−^ (p<0.0004, <0.0002 and <0.0001, respectively) and *sigF^−^* mutants (p<0.0001) compared to wild type but no significant reduction in the *sigE* ^−^ and *sigK^−^* mutants relative to wild type (Figure 6B). This observation was consistent with the RNA-Seq data indicating that σ^G^ activity depends on σ^F^, although it is likely that σ^F^ directly induces the transcription of some σ^G^-dependent genes given the predicted overlap in their promoter specificities [11]. Nevertheless, given that σ^G^ is present at wildtype levels in a *sigF^−^* strain, these observations suggest that σ^F^ regulates σ^G^ activity through a post-translational mechanism.

σ^E^-dependent genes *cd3522* and *spoIVA* were reduced by >100-fold, and *cd1511* by >50-fold, in *sigE* ^−^ relative to wild type, (p<0.0001, <0.0001, and <0.006, respectively), but not in *sigG* ^−^ and *sigK* ^−^ mutants (Figure 6C). Transcript levels of these σ^E^-dependent genes were reduced by ∼5 to 6-fold (p<0.01) in the *sigF^−^* mutant relative to wildtype, indicating that, in the absence of σ^F^, σ^E^ activity is reduced but detectable. Transcript levels of the σ^K^-dependent genes *cd1433*, *cd1067* and *sleC* were significantly reduced by >100-fold in the *sigE* ^−^ (p<0.0001 for each gene) and the *sigK* ^−^ (p<0.0001 for each gene) strains compared to wild type (Figure 6D). σ^K^-dependent gene expression was reduced in the *sigF^−^* mutant by 8 to 10-fold (p<0.01), suggesting that σ^K^ has reduced but detectable activity in the *sigF^−^* strain. Importantly, no statistically significant change for any of these σ^K^-dependent genes was observed in the *sigG* ^−^ mutant relative to wild type, consistent with the RNA-Seq results indicating that σ^K^ activity does not depend on σ^G^ (Figures 4 and 5). Altogether, the qRT-PCR data validated the RNA-Seq data identifying σ^F^, σ^E^, σ^G^, and σ^K^-dependent genes and confirmed that (1) σ^E^, σ^G^, and σ^K^ activity depend on σ^F^, (2) full σ^G^ activity requires σ^F^ but not σ^E^, and (3) σ^K^ activity requires σ^E^ but not σ^G^. It should be noted however that, although σ^F^ is required for full σ^E^ and σ^K^ activity, some degree of σ^E^- and σ^K^-dependent gene expression is observed even in the absence of σ^F^.

### Western blot analyses confirm that σ^K^ activity depends on σ^E^ but not σ^G^

**Figure 7:**
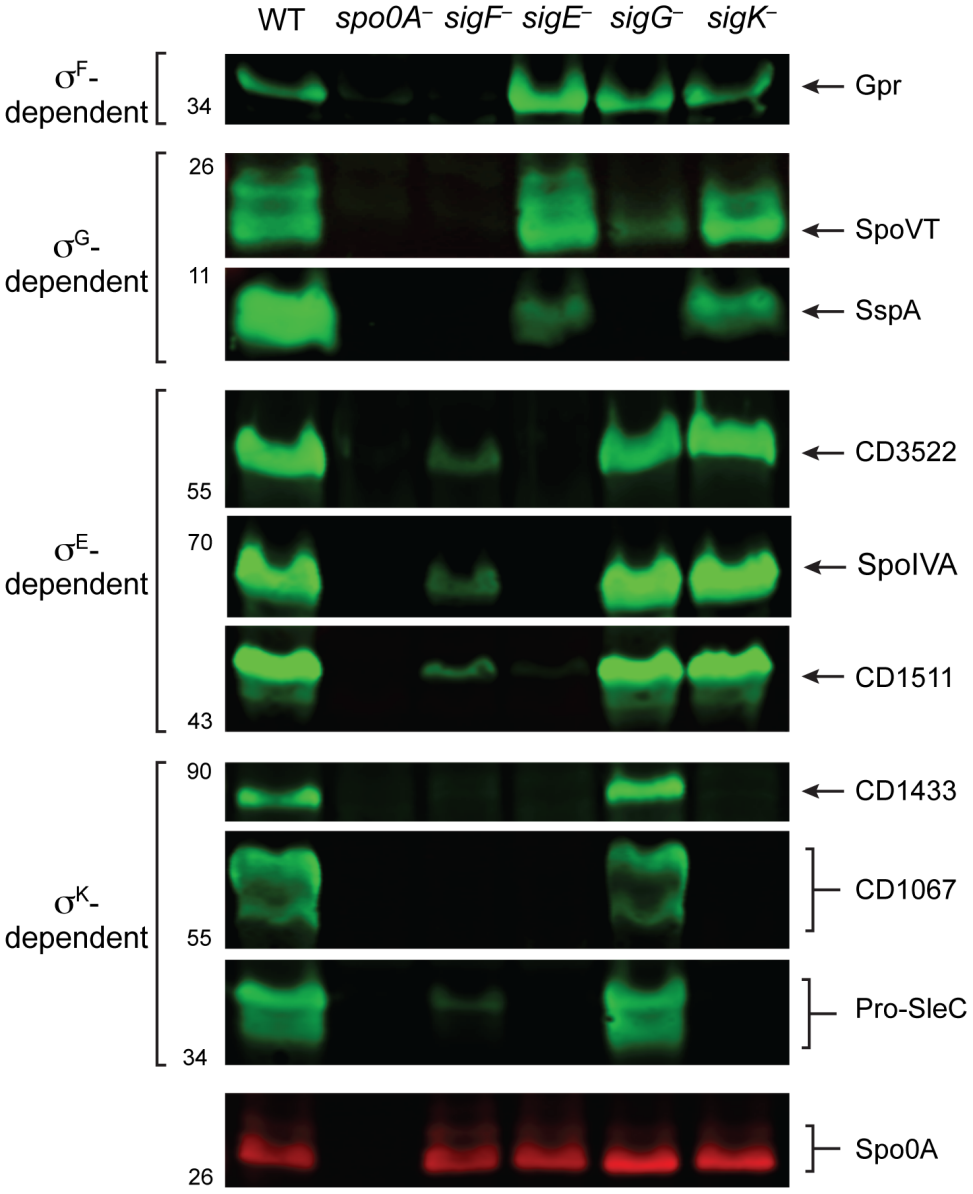
Western blot analyses of proteins encoded by genes induced by specific sigma factors during sporulation. Western blot analyses of proteins encoded by genes identified as being upregulated during sporulation by specific sigma factors. Wildtype (WT), *spo0A^−^*, *sigF^−^*, *sigE^−^*, *sigG^−^*, and *sigK^−^* strains were grown on sporulation media for 18 hrs. SleC undergoes multiple processing steps [76], [85], but only the pro-SleC form is shown. Spo0A was used as a loading control.

Having validated the RNA-Seq data at the transcript level, we next investigated whether changes in transcript levels correlated with changes in protein levels for σ^F^-, σ^E^-, σ^G^-, and σ^K^-regulated genes. To this end, we raised antibodies against proteins encoded by genes identified by RNA-Seq as being σ^F^-, σ^E^-, σ^G^-, and σ^K^-dependent. Western blot analyses of the germination protease Gpr confirmed that only σ^F^ is required for *gpr* expression, while production of the regulatory protein SpoVT and the small acid-soluble protein SspA depended on both σ^F^ and σ^G^. These results indicate that σ^G^ can directly activate the expression of *spoVT* and *sspA* (Figure 7). Western blot analyses for CD3522, SpoIVA, and CD1511 demonstrated that their production depends on σ^E^ but not σ^G^ or σ^K^; these proteins were detected, albeit at greatly reduced levels, in the *sigF^−^* mutant (Figure 7). These results were consistent with the observation that active, processed σ^E^ is present in both *sigG^−^* and *sigK^−^* strains, while only trace amounts of processed σ^E^ could be detected in the *sigF^−^* strain (Figure 2). Analysis of σ^K^-dependent protein production using antibodies specific for CD1433, CD1067 and SleC confirmed that these proteins were absent in the *sigE* ^−^ and *sigK* ^−^ mutants and present in wild type and the *sigG^−^* mutant (Figure 7). Only SleC was reliably detected in the *sigF^−^* mutant, even though *cd1433* and *cd1067* transcripts could be detected in the *sigF^−^* strain (Figure 6D). Nevertheless, taken together these observations confirm that (1) σ^F^ does not require σ^E^, σ^G^, or σ^K^ for activation, (2) full σ^E^ activation requires σ^F^, (3) full σ^G^ activation requires σ^F^ but not σ^E^ or σ^K^, and (4) σ^K^ activation requires σ^F^ and σ^E^ but not σ^G^.

## Discussion

The regulation of sporulation in the Clostridia has remained poorly characterized relative to the model spore-forming bacterium *B. subtilis* because the function and activity of all four sporulation sigma factors has not been simultaneously interrogated in a given *Clostridium* sp. to date. By constructing mutations in genes encoding for individual sporulation sigma factors in the nosocomial pathogen *C. difficile* and performing whole genome transcriptional profiling on these mutants, we identified 314 genes whose expression is activated during sporulation (Table S9) in a Spo0A, σ^F^-, σ^E^-, σ^G^-, and/or σ^K^-dependent manner (Tables S3, S5, S6, S7, S8). These experiments reveal that the sporulation pathway of *C. difficile* exhibits numerous differences relative to *B. subtilis* and other *Clostridium* spp., highlighting the diversity of mechanisms that regulate sporulation sigma factor activity in the Firmicutes.

### Diverse mechanisms regulate sporulation sigma factor activity in the Firmicutes

**Figure 8:**
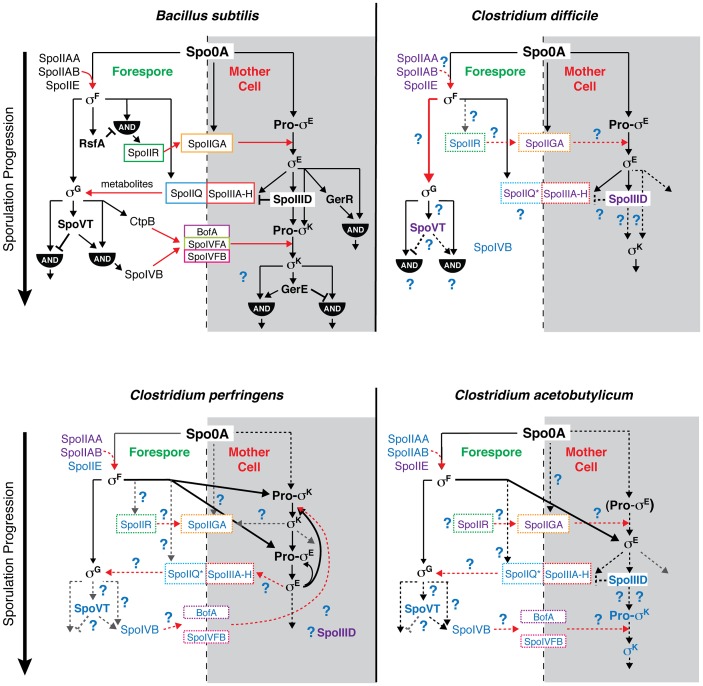
Comparison of sporulation regulatory network architecture in the Firmicutes. The temporal progression of sporulation is shown from top to bottom as determined in *B. subtilis*, *C. difficile*, *C. perfringens*, and *C. acetobutylicum*. Transcription factors and sigma factors are shown in bold, and proteins enclosed in boxes directly participate in trans-septum signaling. Dashed boxes indicate that the function of the proteins in trans-septum signaling has not been tested yet. Text color denotes whether the factor has been detected at both the transcript and protein level (black), at either the transcript or protein level (purple), or has not been tested yet at the transcript or protein level (blue), indicating the need for further experimentation. SpoIIQ* denotes the predicted clostridial homolog to *B. subtilis* SpoIIQ based on bioinformatics analyses [13]. Pro-σ^E^ in *C. acetobutylicum* is shown in parentheses to indicate that the pro-form has not been detected by Western blot [31]. Black arrows indicate transcriptional control of gene expression, red arrows indicate signaling pathways, dashed arrows indicate that the regulatory relationship between the factors has not been tested, and thick arrows demarcate notable points of divergence from the pathway defined in *B. subtilis*. AND gates are indicated. Unique features of the sporulation pathway in *C. difficile* include the post-translational activation of σ^G^ by σ^F^ and the absence of proteolytic activation of σ^K^; the σ^E^-dependent SpoIIIA-H feeding tube appears to be dispensable for σ^G^ activation.

While mutation of all four sporulation sigma factors in *C. difficile* abrogated functional spore formation as expected [11], the regulation and function of these sigma factors in *C. difficile* differed from the regulatory pathways determined for *B. subtilis* and other *Clostridium* spp. The differences between *C. difficile*, *C. perfringens*, *C. acetobutylicum*, and *B. subtilis* sporulation pathways are summarized in Figure 8, as are the similarities.

Similar to *B. subtilis*, our transcriptional and cytological analyses reveal that *C. difficile* σ^K^ functions downstream of σ^E^ to regulate late-stage sporulation events, and σ^G^ functions downstream of σ^F^ to regulate forespore maturation (Figures 2 and 6). In contrast with *B. subtilis*, *C. difficile* σ^G^ is fully active in the absence of σ^E^, and σ^K^ is fully active in the absence of σ^G^ (Figures 6 and 7). The latter observation could have been anticipated given that *C. difficile* σ^K^ lacks an N-terminal pro-peptide, in contrast to all other spore formers [43]. However, the former observation was unexpected because σ^E^-regulated gene products function to activate σ^G^ in the forespore of *B. subtilis*, initiating a positive feedback pathway that increases σ^G^ levels through auto-activation of the *sigG* promoter [44], [46], [57]. In particular, *B. subtilis* σ^G^ activation requires the formation of a σ^E^-dependent “feeding tube” [20], [21], [55], [58], [59], which maintains forespore integrity by transporting small molecules from the mother cell into the forespore [20], [21], [55]. This mode of regulation couples the activation of the forespore-specific σ^G^ to σ^E^-controlled events in the mother cell. In contrast, our results indicate that *C. difficile* σ^G^ is active in the absence of σ^E^-dependent feeding tube gene expression (Figures 5 and 6, Tables S6 and S7). Nevertheless, even though σ^G^ was active at wildtype levels in the *sigE^−^* mutant (Figures 6 and 7), it remains possible that σ^G^ activity may be mislocalized in the mother cell cytosol, similar to the premature activation of σ^G^ in Lon^−^ and anti-σ^G^ sigma factor CsfB^−^ cells [57], [60], [61].

Even though *C. difficile* σ^G^ can be fully activated in the absence of σ^E^, our results further show that σ^G^ is post-translationally activated in a σ^F^-dependent manner (Figures 2 and 6). These results raise the intriguing question as to how σ^F^ activates σ^G^ independent of σ^E^ in *C. difficile*. In *B. subtilis*, multiple post-translational mechanisms control σ^G^ activity; however, aside from the feeding tube, these mechanisms are inhibitory rather than activating. In *B. subtilis* the Lon protease reduces σ^G^ activity in the mother cell [60], while the anti-σ factors SpoIIAB [57], [62] and CsfB (also known as Gin) [61], [63], [64] prevent σ^G^ activity in the forespore until engulfment is complete. Whether these factors inhibit σ^G^ activity in *C. difficile* is unknown, although *C. difficile* does not appear to encode a CsfB homolog. In future studies, it will be interesting to determine whether σ^F^ functions to activate σ^G^ directly or alleviate its inhibition, and whether *C. difficile* sporulation sigma factors exhibit compartment-specific activity similar to *B. subtilis*.

Interestingly, the morphology of the *C. difficile sigG^−^* mutant differed considerably from a *B. subtilis sigG^−^* mutant. While *B. subtilis sigG^−^* mutant forespores are normal in appearance despite lacking both a coat and cortex [44], *C. difficile sigG^−^* mutant forespores produced layers resembling spore coat around the forespore and exhibited defects in engulfment and structural integrity (Figures 3 and S3). The forespore membrane ruffling phenotype of *C. difficile sigG^−^* mutants was reminiscent of *B. subtilis* feeding tube mutant phenotypes [21], suggesting that σ^G^ may encode proteins required to “nurture” the *C. difficile* forespore. Alternatively, σ^G^ could regulate a cytoskeletal or cortex component that confers structural integrity to the forespore. Such proteins could be represented in the σ^G^-regulated genes identified in this study (Table S7).

The phenotype of the *C. difficile sigF^−^* mutant also differed from its cognate mutant in *B. subtilis*, since the *sigF^−^* mutant produced low levels of σ^E−^ and σ^K−^ induced gene products (Figure 7) and regions that resembled mislocalized coat in the mother cell cytosol (Figure 3) [47]. In *B. subtilis*, σ^F^ is required to activate the expression of *spoIIR*, which encodes an intercellular signaling protein that activates SpoIIGA, the protease responsible for activating pro-σ^E^ [65], [66]. Whether the trace amounts of σ^E^ processing observed in the *C. difficile sigF^−^* mutant results from low-level expression of *spoIIR* or *spoIIGA*, or whether an unknown protease activates σ^E^, remains to examined.

Comparison of the sporulation pathway of *C. perfringens* relative to *C. difficile* indicates that both organisms proteolytically activate σ^E^ in a σ^F^-dependent manner (Figure 2), although it should be noted that a *C. perfringens sigF^−^* mutant does not make σ^E^, σ^G^, or σ^K^ [27] in contrast with *C. difficile* (Figure 2). Since the phenotypes of *C. perfringens sigF^−^* and *sigG^−^* mutants have not been examined by electron microscopy, the precise stage at which they are arrested remains unclear. Nevertheless, unlike *C. perfringens* (and *C. botulinum*) where σ^K^ is essential for both early and late stage sporulation events (Figure 8) [28], [29], *C. difficile* σ^K^ is needed only at late stages of sporulation. Furthermore, *C. perfringens* σ^K^ is produced at low levels in an unprocessed form in a *sigE^−^* mutant; σ^E^ is made at low levels in a *C. perfringens sigK^−^* mutant; and *sigE* and *sigK* expression appear to be auto-activated [28]. In contrast, no *sigK* expression was observed in the absence of σ^E^ in *C. difficile*.

The sporulation pathway of *C. difficile* appears to be most similar to the *C. acetobutylicum* pathway. Both *C. difficile* and *C. acetobutylicum* sigma factors σ^F^, σ^E^, and σ^G^ appear to function at similar stages of sporulation, although *C. acetobutylicum sigF^−^* and *sigE^−^* mutants exhibit more severe phenotypes than in *C. difficile* in that they fail to initiate and complete asymmetric division, respectively [30], [31], and σ^F^ is required to activate *sigG* transcription in *C. acetobutylicum* [30] unlike *C. difficile*. Similar to *C. difficile*, however, *C. acetobutylicum* σ^G^ does not require σ^E^ for auto-activation of *sigG* expression, although it is unclear whether *C. acetobutylicum* σ^G^ is active in the absence of σ^E^ [31]. Lastly, loss of *sigG* expression in *C. acetobutylicum* results in pleiotropic defects in coat and cortex formation and forespore integrity similar to *C. difficile* (Figure 3, [31]). Since a *sigK^−^* mutant in *C. acetobutylicum* has not been described, it will be interesting to determine whether *C. acetobutylicum* σ^K^ function is more similar to *C. difficile* than to *C. perfringens* and whether these differences correlate to the presence of the *skin* element, an ∼15 kb prophage-like element that interrupts the *sigK* gene in *C. difficile* but not other *Clostridium* spp. [43]. Nevertheless, our collective transcriptional and cytological analyses of sporulation sigma factor function in *C. difficile* suggest that novel mechanisms regulate σ^G^ and σ^K^ activation relative to other spore-forming organisms (Figure 8). Further studies are needed to determine the regulatory interplay between *C. difficile* sporulation sigma factors and their downstream auxiliary regulators SpoVT and SpoIIID, which modulate the expression of σ^G^- and σ^K^-regulated genes, respectively, in *B. subtilis* [24], [26], [67], [68] and are conserved in *Clostridium* spp.

### Transcriptional profiling of sporulation in *C. difficile* identifies new sporulation genes

By performing whole genome transcriptional profiling on the different sporulation sigma factor mutants, we have identified distinct subsets of genes that are σ^F^-, σ^E^-, σ^G^-, and σ^K^-dependent. The number of genes determined to be σ^G^-dependent in *C. difficile* was relatively small (34 genes) relative to *B. subtilis*, where the σ^G^ regulon comprises ∼100 genes [11], [25], [26]. Similarly, the σ^E^ and σ^K^-dependent genes (169 and 31 genes, respectively) identified by our study were smaller than their cognate regulons in *B. subtilis* (270 and 150 genes, respectively, [24]). While the parameters we used to define genes as being σ^F^-, σ^E^-, σ^G^-, and σ^K^-dependent were relatively stringent, relaxing these parameters did not result in large increases in gene numbers. One explanation for the smaller size is that *C. difficile* activates fewer genes during sporulation than *B. subtilis*. A more likely explanation is that the asynchronous population of sporulating cells (Figures 1 and S2, [37]) limits the detection of genes that are transiently expressed during discrete stages of sporulation or genes that are expressed at low levels during sporulation [24]–[26], [69]. Since the RNA samples used in the RNA-Seq analysis were harvested from a sporulation timepoint in which phase-bright forespores were produced by wildtype cells (Figure S2), fewer cells in the population are likely to be at early stages sporulation. As a result, early sporulation genes may be under-represented in our data set; for example, σ^F^-dependent early sporulation gene transcripts from *spoIIR* and *spoIIP* were almost undetectable by RNA-Seq (Table S4). In addition, genes that are regulated by more complex mechanisms beyond upregulation by a specific sigma factor are likely to be under-represented in our data set. Sporulation genes that are subject to incoherent feed forward loop regulation, in which their expression is induced by a given sigma factor and repressed by a downstream regulator such as SpoVT-mediated inhibition of *sigG* transcription in *B. subtilis* [26], may not be detected in our data set. Unraveling the complexities of sporulation gene regulation in *C. difficile* will require further characterization of the kinetics of sporulation and the analysis of mutants defective in auxiliary sporulation regulators.

Of the 51 genes proposed to comprise the core set of sporulation genes in spore-forming Firmicutes by bioinformatics analyses that are conserved in *C. difficile* [13], 34 were identified in our RNA-Seq analyses, leaving 17 that were not identified in our transcriptional analyses (Table S4). Seven of these genes do not have detectable homologs in the 630 *C. difficile* genome, and 6 were expressed at low levels with a base mean of expression less than 15 (Table S4).

Although some sporulation-induced genes were likely not detected in our analyses due to low levels of expression, the transcriptional profiling data presented here identify a promising set of genes that are likely to encode proteins with important roles in spore formation. Of the six spore coat proteins recently identified in a proteomic analysis of *C. difficile* [50], [53], all were identified in our RNA-Seq experiments. Three of these spore coat genes were determined to be σ^K^-dependent, consistent with their predicted role as components of the outer coat (Table S4). Notably, σ^K^-regulated genes were among the most abundantly expressed genes induced during sporulation, comprising 6 of the 10 most highly expressed sporulation genes (Table S9). The σ^K^-regulated *CD1067* gene was the most highly expressed gene induced during sporulation in *C. difficile*. Cysteine-rich CD1067 was also one of the most abundant spore proteins identified in proteomic analyses of purified spores and is encoded in a 7.5 kB region enriched in genes encoding spore proteins [70]. Western blot analyses of cysteine-rich CD1067 revealed that it forms higher order multimers that are highly resistant to denaturing conditions (data not shown), consistent with the proposal that CD1067 may form a rigid, disulfide-bonded structure around the spore coat upon exposure to atmospheric oxygen, for example during excretion from the host [70]. Intriguingly, CD1067 is encoded in a region enriched in highly expressed, σ^K^-regulated genes encoding hypothetical proteins unique to *C. difficile*, with 8 of the 9 genes in this region being induced during sporulation and 6 of the 9 being σ^K^-regulated. These genes may encode coat proteins that confer structural integrity and/or resistance to the *C. difficile* spore coat and thus may play important roles in disease transmission and/or represent good candidates for developing diagnostic reagents.

Although the number of σ^G^-dependent genes identified by our study was small, a number of these genes encode proteins with important functions in the forespore of *B. subtilis*, specifically *sspA*, *sspB*, *dacF*, *spoVT*, and *spoVAD* [26], [67], [71]–[73]. Since *B. subtilis* σ^G^ induces the expression of genes encoding the germinant receptors (of which there are no homologs in *C. difficile* [12], [34]), it seems likely that some of the σ^G^-dependent genes identified in our study encode proteins that transduce the germinant signal into the spore core. It will be interesting to determine whether any of the σ^G^-regulated genes identified in our study play important roles in regulating germination and thus disease transmission.

Genes encoding hypothetical proteins were the most abundant class of genes identified in our study (82 in total, Table S10). Twenty of these hypothetical proteins were detected in proteomic analyses of *C. difficile* spores [70]. Indeed, two of the hypothetical proteins were previously shown to be part of the spore coat [50], and we have validated three additional proteins as localizing to the spore coat (data not shown). BLAST searches with the hypothetical proteins identified by RNA-Seq indicate that 16 have no known homologs. These *C. difficile*-specific proteins could comprise part of the spore coat, since coat proteins are often poorly conserved, species-specific, and categorized as hypothetical proteins [10], [11].

Taken together, by examining the regulatory interplay between sporulation sigma factors in *C. difficile*, our study highlights that diverse pathways regulate sporulation in the Firmicutes and that considerable work is needed to map these pathways in the Clostridia. By using whole genome transcriptional profiling to define a large set of genes that are activated by Spo0A, σ^F^, σ^E^, σ^G^, and/or σ^K^, our study also provides a framework for identifying new proteins that are necessary for sporulation and determining the role of these proteins in forming a functional, infectious spore. Studies of this nature may lead to the identification of biomarkers for *C. difficile* spores and candidates for vaccine development.

## Materials and Methods

### Bacterial strains and growth conditions

All *C. difficile* strains are listed in Table 1 and derive from the parent strain JIR8094 [33], an erythromycin-sensitive derivative of the sequenced clinical isolate 630 [34]. *C. difficile* strains were grown on solid brain heart infusion media supplemented with yeast extract (BHIS: 37 g brain heart infusion, 5 g yeast extract, 0.1% (w/v) *L*-cysteine, 15 g agar per liter) [74]. Taurocholate (TA; 0.1% w/v), thiamphenicol (5–10 µg/mL), kanamycin (50 µg/mL), cefoxitin (16 µg/mL), FeSO_4_ (50 µM), and/or erythromycin (10 µg/mL) were used to supplement the BHIS media as indicated. Cultures were grown at 37°C, under anaerobic conditions using a gas mixture containing 85% N_2_, 5% CO_2_, and 10% H_2_.

**Table 1:** *C. difficile* strains used in this study.

Strain	*C. difficile* strain	Relevant genotype or features
11	JIR8094	*erm*-sensitive derivative of 630 [33]
13	630	Clinical isolate 630 [34]
35	*spo0A^−^*	JIR8094 *spo0A*::*ermB*
50	*sigE^−^*	JIR8094 *sigE*::*ermB*
67	*sigK^−^*	JIR8094 *sigK::ermB*
71	JIR8094/pMTL84151	JIR8094/pMTL84151
99	*sigG* ^−^	JIR8094 *sigG::ermB*
106	*sigF^−^*	JIR8094 *sigF::ermB*
110	JIR8094/pMTL83151	JIR8094/pMTL83151
127	*sigE^−^*/pMTL83151-*spoIIGA-sigE*	JIR8094 *sigE::ermB/*pMTL83151-*spoIIGA-sigE*
143	*sigG* ^−^/pMTL83151-*sigG*	JIR8094 *sigG*::*ermB*/pMTL83151-*sigG*
145	*sigK* ^−^/pMTL83151-*sigK*	JIR8094 *sigK*::*ermB*/pMTL83151-*sigK*
159	*sigK* ^−^/pMTL83151	JIR8094 *sigK*::*ermB*/pMTL83151
164	*sigG* ^−^/pMTL83151	JIR8094 *sigG*::*ermB*/pMTL83151
167	*sigE^−^*/pMTL83151	JIR8094 *sigE::ermB/*pMTL83151
214	*sigF^−^*/pMTL84151	JIR8094 *sigF::ermB/*pMTL84151
218	*spo0A^−^*/pMTL83151	JIR8094 *spo0A::ermB/*pMTL83151
222	*sigF^−^*/pMTL84151-*spoIIAA-spoIIAB-sigF*	JIR8094 *sigF::ermB*/pMTL84151-*spoIIAA-spoIIAB-sigF*
289	*spo0A^−^*/pMTL84151	JIR8094 *spo0A::ermB/*pMTL84151

Sporulation was induced on media containing BHIS and SMC (90 g BactoPeptone, 5 g protease peptone, 1 g NH_4_SO_4_, 1.5 g Tris base, 15 g agar per liter) [50], at 70% SMC and 30% BHIS (70∶30 media, 63 g BactoPeptone, 3.5 g Protease Peptone, 11.1 g BHI, 1.5 g yeast extract, 1.06 g Tris base, 0.7 g NH_4_SO_4_, 15 g agar per liter) [37]. 70∶30 agar (supplemented as appropriate with thiamphenicol at 5–10 µg/mL) was inoculated from a starter culture grown on solid media.

HB101/pK424 strains were used for conjugations and BL21(DE3) strains were used for protein expression. *E. coli* strains were routinely grown at 37°C, shaking at 225 rpm in Luria-Bertani broth (LB). Media was supplemented with chloramphenicol (20 µg/mL), ampicillin (50–100 µg/mL), or kanamycin (30 µg/mL) as indicated.

### *E. coli* strain construction

All strains are listed in Table S11; all plasmids are listed in Table S12; and all primers used are listed in Table S13. For disruption of *spo0A*, *sigE*, *sigG*, *sigK, and sigF*, a modified plasmid containing the retargeting group II intron, pCE245 (a gift from C. Ellermeier, University of Iowa), was used as the template. Primers used to amplify the targeting sequence from the template carried flanking regions specific for each gene target and are listed as follows: *spo0A* (#539, 540, 541 and 532, the EBS Universal primer as specified by the manufacturer (Sigma Aldrich), *sigE* (#653, 654, 655 and 532), *sigG* (#728, 729, 730, and 532), *sigK* (#681, 682, 683, and 532) *and sigF* (#775, 776, 777, and 532). The *spo0A* disruption mutant was constructed using the same primers as Underwood *et al.* [36]. The resulting retargeting sequences were digested with BsrGI and HindIII and cloned into pJS107 (a gift from J. Sorg, University of Texas A&M), a derivative of pJIR750ai (Sigma Aldrich) [32]. The ligations were transformed into DH5α and confirmed by sequencing. The resulting plasmids were used to transform HB101/pK424.

To construct the *sigE* complementation construct, primers #725 and 726 were used to amplify a fragment containing 252 bp upstream and 156 bp downstream of the two gene *spoIIGA-sigE* operon using 630 genomic DNA as the template. To construct the *sigG* complementation construct, primers #835 and 836 were used to amplify 288 bp upstream and 16 bp downstream of *sigG* using 630 genomic DNA as the template. The *sigK* complementation construct was made using PCR splicing by overlap extension (SOE) [75]. Primer pair #734 and 736 was used to amplify the 5′ SOE product, while primer pair #735 and 737 was used to amplify the 3′ SOE product. The resulting fragments were mixed together, and the flanking primers #734 and #737 were used to amplify an 898 bp fragment corresponding to the *sigK* gene including 256 bp region of upstream sequence. This strategy was used to clone an intact *sigK* gene with the skin element excised [43]. To construct the *sigF* complementation construct, primers #954 and #956 were used to amplify 88 bp upstream and 19 bp downstream of *spoIIAA-spoIIAB-sigF* operon, using 630 genomic DNA as the template. All complementation constructs were digested with NotI and XhoI and ligated into pMTL83151 [48] digested with the same enzymes, with the exception of the *sigF* complementation construct, which was cloned into pMTL84151 digested with the same enzymes [48].

To construct strains producing recombinant CD3522, σ^E^, σ^G^, σ^F^, Gpr, SpoVT, and SspA for antibody production, primer pairs #498 and 499; #596 and 597; #727 and 688; #723 and 724; #790 and 791; #883 and 884; #975 and 976; and #885 and 886 were used to amplify the *cd3522, sigE*, *sigG*, *sigF, gpr, spoVT, and sspA* genes lacking stop codons, respectively, using 630 genomic DNA as the template. The *sigE* expression construct deletes the sequence encoding the first 23 amino acids of σ^E^, which removes its membrane-tethering domain and improves the solubility of the protein in *E. coli*. The resulting PCR products were digested with NdeI and XhoI, (or NheI and XhoI for *gpr*) ligated to pET22b (or pET21a for *gpr* and *sspA*), and used to transform DH5α. To construct a strain producing recombinant σ^K^, PCR SOE was used to amplify the *sigK* gene lacking the skin element. Primer pair #689 and 736 was used to amplify the 5′ SOE product, while primer pair #735 and 737 was used to amplify the 3′ SOE product. The resulting fragments were mixed together, and the flanking #689 and #737 primers were used to amplify the *sigK* gene including the TAA stop codon. The resulting PCR product was digested with NcoI and XhoI, ligated to pET30a digested with the same enzymes, and used to transform DH5α. The resulting pET22b-*cd3522*, pET22b-*sigE*, pET22b-*sigG*, pET30a-*sigK*, pET22b-*sigF*, pET21a-*gpr*, pET22b-*spoVT*, and pET21a-*sspA* plasmids were used to transform BL21(DE3) for protein expression.

### *C. difficile* strain construction

*C. difficile* strains were constructed using TargeTron-based gene disruption as described previously (Figure S1, [32], [37], [76]). TargeTron constructs in pJS107 were conjugated into *C. difficile* using an *E. coli* HB101/pK424 donor strain. HB101/pK424 strains containing the appropriate pJS107 construct were grown aerobically to exponential phase in 2 mL of LB supplemented with ampicillin (50 µg/mL) and chloramphenicol (10 µg/mL). Cultures were pelleted, transferred into the anaerobic chamber, and resuspended in 1.5 mL of late-exponential phase *C. difficile* JIR8094 cultures (grown anaerobically in BHIS broth). The resulting cell mixture was plated as seven 100 µL spots onto pre-dried, pre-reduced BHIS agar plates. After overnight incubation, all growth was harvested from the BHIS plates, resuspended in 2.5 mL pre-reduced BHIS, and twenty-one 100 µL spots per strain were plated onto BHIS agar supplemented with thiamphenicol (10 µg/mL), kanamycin (50 µg/mL), and cefoxitin (16 µg/mL) to select for *C. difficile* containing the pJS107 plasmid. After 24–48 hrs of anaerobic growth, single colonies were patched onto BHIS agar supplemented with thiamphenicol (10 µg/mL), kanamycin (50 µg/mL), and FeSO_5_ (50 µM) to induce the ferredoxin promoter of the group II intron system. After overnight growth, patches were transferred to BHIS agar plates supplemented with erythromycin (10 µg/mL) for 24–72 hrs to select for cells with activated group II intron systems. Erythromycin-resistant patches were struck out for isolation onto the same media and individual colonies were screened by colony PCR for a 2 kb increase in the size of *spo0A* (primer pair #556 and 557), *sigE* (primer pair #687 and 688), *sigG* (primer pair #723 and 724), *sigK* (primer pair #689 and 690), and *sigF* (primer pair #790 and 791) (Figure S1). A minimum of two independent clones from each mutant strain was phenotypically characterized.

### *C. difficile* complementation

HB101/pK424 donor strains carrying the appropriate complementation construct were grown in LB containing ampicillin (50 µg/mL) and chloramphenicol (20 µg/mL) at 37°C, 225 rpm, under aerobic conditions, for 6 hrs. *C. difficile* recipient strains *spo0A^−^*, *sigE^−^*, *sigG^−^*, *sigK^−^*, and *sigF^−^*, containing group II intron disruptions, were grown anaerobically in BHIS broth at 37°C with gentle shaking for 6 hrs. HB101/pK424 cultures were pelleted at 2500 rpm for 5 min and the supernatant was removed. Pellets were transferred to the anaerobic chamber and gently resuspended in 1.5 mL of the appropriate *C. difficile* culture. The resulting mixture was inoculated onto pre-dried, pre-reduced BHIS agar plates, as seven 100 µL spots for 12 hrs. All spots were collected anaerobically and resuspended in 1 mL PBS. The resulting suspension was spread onto pre-dried, pre-reduced BHIS agar plates supplemented with thiamphenicol (10 µg/mL), kanamycin (50 µg/mL), and cefoxitin (10 µg/mL) at 100 µL per plate, five plates per conjugation. Plates were monitored for colony growth for 24–72 hrs. Individual colonies were struck out for isolation and analyzed for complementation by phase contrast microscopy, Western blot analysis and transmission electron microscopy. A minimum of two independent clones from each complementation strain was phenotypically characterized.

For the *sigF* complementation, a pMTL84151 plasmid backbone was used. The complementation protocol was followed as described except that after spots were collected from overnight growth on BHIS plates, the resulting PBS suspension was spotted onto three BHIS agar plates supplemented with thiamphenicol (10 µg/mL), kanamycin (50 µg/mL), and cefoxitin (16 µg/mL) with 7–100 µL spots per plate.

### Sporulation assay

*C. difficile* strains were grown from glycerol stocks on BHIS plates supplemented with TA (0.1% w/v), or with both TA and thiamphenicol (5–10 µg/mL) for strains with pMTL83151-derived or pMTL84151-derived plasmids. Cultures grown on BHIS agar plates were then used to inoculate 70∶30 agar plates (with thiamphenicol at 5–10 µg/mL as appropriate) for 18–48 hrs as previously described [37]. Sporulation induced lawns were harvested in PBS, washed once, resuspended in 0.2 mL of PBS, visualized by phase contrast microscopy, and/or further processed for analysis by transmission electron microscopy or Western blotting.

### Heat resistance assay

*C. difficile* strains grown from glycerol stocks on BHIS plates supplemented with taurocholate and thiamphenicol (described above) were inoculated on to 70∶30 media containing thiamphenicol (5–10 µg/mL). After 30 hrs of growth, cells were harvested in 1.0 mL PBS, and split into two tubes. One tube was heat shocked at 60–65°C for 25 minutes. Both heat-shocked and non-heat shocked cells were serially diluted, and cells were plated on pre-reduced BHIS-TA plates. After 20 hrs on BHIS-TA, colonies were counted, and cell counts were determined. The percent of heat-resistant spores was determined based on the ratio of heat-resistant cells to total cells, and sporulation efficiencies were determined based on the ratio of heat-resistant cells for a strain compared to wild type. Results are based on a minimum of three biological replicates. *spo0A* ^−^ containing empty vector was included as a control for all assays [77].

### Fluorescence and light microscopy

For fluorescence microscopy studies, *C. difficile* strains were harvested in PBS after 18 hours of growth on 70∶30 media, pelleted, and resuspended in 1.0 mL PBS containing 1 µg/mL FM4-64 (Molecular Probes) and 15 µg/mL Hoechst 33342 (Molecular Probes). The bacterial suspension (4 µL) was added to a freshly prepared 1% agarose pad on a microscope slide, covered with a 22×22 mm #1 coverslip and sealed with VALAB (1∶1∶1 of vaseline, lanolin, and beeswax) as previously described [78]. Phase and fluorescence microscopy were performed using a Nikon PlanApo 100× Ph3 oil immersion objective (1.4 NA) on a Nikon Eclipse TE300 epifluorescence microscope. Five fields for each sample were acquired with an iXon3 885 EMCCD camera (Andor) cooled to −70°C with frame averaging set to 4 and an EM gain setting of 3, and driven by NIS-Elements software (Nikon). Images were subsequently imported into Adobe Photoshop CS6 for minimal adjustments in brightness/contrast levels and pseudocoloring.

Phase-contrast microscopy for imaging the samples used for RNA-Seq was performed as previously described [37].

Quantification of total cells undergoing sporulation was determined by analyzing multiple fields for each strain at random. Greater than 200 cells were enumerated for each strain. For cultures analyzed by fluorescence microscopy, sporulating cells were identified as either having a polar septum with or without DNA staining in the forespore, a phase-dark forespore with or without DNA staining in the forespore compartment, a phase-bright forespore without DNA staining, or a free spore (no mother cell compartment).

### Electron microscopy

One hundred microliters of bacterial cell suspension samples from sporulation assays were prepared as previously described [37].

### Western blot analyses

Sporulation assay *C. difficile* cells (50 µL of PBS suspension) were freeze-thawed three times, diluted in 100 µL EBB buffer (8 M urea, 2 M thiourea, 4% (w/v) SDS, 2% (v/v) β-mercaptoethanol), and incubated at 95°C for 20 min with vortexing every 5 min. Samples were centrifuged for 5 min at 15,000 rpm and 7 µL of 4× sample buffer (40% (v/v) glycerol, 1 M Tris pH 6.8, 20% (v/v) β-mercaptoethanol, 8% (w/v) SDS, and 0.04% (w/v) bromophenol blue), was added. Protein samples were incubated again at 95°C for 15 minutes with vortexing followed by centrifugation for 5 min at 15,000 rpm. SDS-PAGE gels (12%–15%) were loaded with 5 µL of protein prep. Gels were transferred to Bio-Rad PVDF membrane and blocked in 50% PBS:50% Odyssey Blocking Buffer with 0.1% (v/v) Tween for 30 min at RT. Polyclonal rabbit anti-σ^E^, anti-σ^G^, anti-σ^F^, anti-SpoIVA [37], and anti-CD1433 [76], anti-CD1067, anti-Gpr, anti-SpoVT, and anti-SspA antibodies were used at a 1∶1,000 dilution and anti-σ^K^, anti-CD1511, anti-SleC [76], and anti-CD3522 at a 1∶5,000 dilution. Monoclonal mouse anti-Spo0A [37] was used at a 1∶10,000 dilution. IRDye 680CW and 800CW infrared dye-conjugated secondary antibodies were used at a 1∶20,000 dilutions. The Odyssey LiCor CLx was used to detect secondary antibody fluorescent emissions for Western blots.

### Antibody production

The anti-Δ230aa-σ^E^, anti-σ^G^, anti-σ^K^, anti-σ^F^, anti-CD3522, anti-Gpr, anti-SpoVT, and anti-SspA antibodies used in this study were raised in rabbits by Cocalico Biologicals (Reamstown, PA). The antigens Δ230aa-σ^E^-His_6_, σ^G^-His_6_, His_6_^−^σ^K^, σ^F^-His_6_, CD3522-His_6_, Gpr-His_6_, SpoVT-His_6_, and SspA-His_6_, were purified on Ni^2+^-affinity resin from *E. coli* strains #755, 743, 756, 921, 577, 853, 881, and #SspA respectively, as described above. Cultures were grown and protein expression was induced with 250 µM IPTG overnight at 19°C. *E. coli* cells were harvested, pelleted, and resuspended in 25 mL of low imidazole buffer (LIB; 500 mM NaCl, 50 mM Tris-HCl, pH 7.5, 15 mM imidazole, 10% (v/v) glycerol). Cells were flash frozen in liquid nitrogen, thawed, and lysed by sonication (45 sec burst followed by 5 min on ice for 3 cycles). For protein affinity purification, the lysate was centrifuged at 16,000× *g* for 30 min, supernatant was collected and added to pre-washed Ni^2+^-affinity resin for 4 hrs at 4°C. Bound beads were centrifuged at 2,000× *g* for 2 min at 4°C and washed once in LIB. Beads were reconstituted in 375 µL of high imidazole buffer (HIB; 500 mM NaCl, 50 mM Tris-HCl, pH 7.5, 200 mM imidazole, 10% (v/v) glycerol), incubated on a nutator for 15 min at RT, centrifuged, and eluate was collected. Beads were reconstituted with HIB for a total of five sequential elutions.

Polyclonal antibodies against CD1067 were raised in rabbits against a peptide derived from CD1067 (INSEDMRGFKKSHHC, Genscript); the polyclonal antibodies were affinity-purified using the indicated peptide (Genscript).

### RNA processing

RNA for RNA-Seq was extracted from WT, *spo0A^−^*, *sigE^−^*, *sigF^−^*, *sigG^−^*, and *sigK^−^ C. difficile* cell suspensions, from an 18 hr sporulation assay (described earlier), using a FastRNA Pro Blue Kit (MP Biomedical) and a FastPrep-24 automated homogenizer (MP Biomedical, setting 6.0, 45 seconds for 3 cycles). Contaminating genomic DNA was depleted using a column-bound DNase treatment with an RNeasy Kit (Qiagen) followed by two suspension DNase treatments (New England Biolabs), according to manufacturer's recommendations. Samples were tested for genomic DNA contamination using quantitative PCR for 16S rRNA and the *sleC* gene. DNAse-treated RNA (5 µg) was mRNA enriched using a Ribo-Zero Magnetic Kit (Epicentre).

RNA isolated for qRT-PCR was processed identically except that mRNA enrichment was done using an Ambion MICROB*Express* Bacterial mRNA Enrichment Kit (Invitrogen). Reverse transcription of enriched RNA was done using the Super Script First Strand cDNA Synthesis Kit (Invitrogen) with random hexamer primers.

### RNA-Seq library construction and sequencing

Enriched mRNA (100 ng) was submitted to the Advanced Technology Genome Center Core Lab at the University of Vermont for massively parallel sequencing on an Illumina HiSeq 1000. cDNA synthesis was carried out using the Ovation Prokaryotic RNA-Seq System (Nugen), according to manufacturer's instructions. Libraries were prepared using the Ovation ultralow multiplex kit (Nugen, 0304/0305-32) according to manufacturer's instructions. Briefly, samples were end-repaired, mono-adenylated, ligated to index/adaptors, and then amplified for 15 cycles (after a PCR titration was performed). Completed libraries were quantitated using a SYBR Fast Universal qPCR Kit (KAPA Biosystems). Paired end sequencing of samples was performed using a total of 10 pM of library in each flow cell lane. The samples were indexed and pooled in equal amounts to generate equal read coverage.

### RNA-Seq analysis

Sequence calls and quality scores were produced in BCL format from images using Illumina RTA v1.13 with default parameters. Read pairs were mapped to libraries (demultiplexed) and converted to Fastq format using Illumina CASAVA 1.8.2 with default parameters. Adapters were clipped and reads were trimmed to remove the first 12 and last 11 cycles using Trimmomatic [79], dropping read pairs for which at least one read was less than 50 bp. The *C. difficile* 630 genome (NC_009089) sequence was modified by removing the *sigK* intervening (*skin*) element. The *C. difficile* 630 genome annotation was modified by the addition of *sigK*. Read pairs were aligned to the modified *C. difficile* 630 genome (NC_009089) using BWA 0.6.1 with default parameters with one exception (−q 20). Read pairs were mapped to NC_009089 gene annotation using the countOverlaps procedure of the R/Bioconductor IRanges package [80]–[82]. Counts associated with rRNA were removed. Counts associated with the same library were pooled. Reads were of high quality (median Phred score of 39 and a first quartile of 35) as were alignments (median mapping quality score, MAPQ of 60). Median fragment lengths were between 180 and 250.

The vast majority of unmapped sequences failed to align to sequences in the NCBI non-redundant database using a blastn and blastx search. There was no indication of highly represented reads among unmapped sequences. Since the majority of reads failed to map to known natural sequences, and since sequences can arise during library preparation particularly when the input sample is small, sequences that failed to map to the *C. difficile* genome likely represent spurious sequences produced during library construction.

Differential expression statistics reflecting both effect size (fold-change) and statistical significance (p-value adjusted based on the method of Benjamini and Hochberg [83]) were calculated using DESeq [49]. Duplicate reads were excluded from these analyses. Differentially expressed genes were identified based on a minimum fold-change (higher in the reference sample than the query) and maximum p-value. Tables showing genes whose expression was downregulated by ≥4-fold with an adjusted p-value of ≤0.05 during sporulation are provided in the Supplementary Information (Tables S3, S4, S5, S6, S7, S8, S9, S10). A table showing genes whose expression was upregulated by ≥4-fold with an adjusted p-value of ≤0.05 in a Spo0A-dependent manner are shown in Table S14.

The log_2_-transformed expression of genes that were downregulated by ≥4-fold with an adjusted p-value of ≤10^−5^ in the *spo0A* ^−^ strain relative to wild type expression were represented in a heat map using the heatmap.2 procedure of the R/Bioconductor gplots package with default options [84]. Expression levels in *spo0A^−^* were not shown because the differential expression between *spo0A^−^* and wild type was biased by the method used to select genes. Expression levels in the other four strains relative to *spo0A^−^* were centered, scaled, and mapped to a red-green color scale.

### Quantitative RT-PCR

For the RNA-Seq validation, expression levels of *gpr*, *cd0125* (*spoIIQ*), *cd2376*, *cd1511*, *cd3522*, *spoIVA*, *cd1433*, *cd1067*, *sleC*, *sspB*, *spoVT*, *dacF*, and *rpoB* (housekeeping gene) were run on WT, *spo0A^−^*, *sigF^−^ sigE^−^*, *sigG^−^*, and *sigK^−^* cDNA templates in three replicate reactions using gene-specific primer pairs #1187 and 1188; #1213 and 1214; #1191 and 1192; #796 and 797; #989 and 990; #798 and 799; #792 and 793; #1030 and 1031; #575 and 576; #810 and 811; #995 and 996; #993 and 994; #1002 and 1003, respectively. Quantitative real-time PCR was performed using SYBR Green JumpStart Taq Ready Mix (Sigma), 50 nM of gene specific primers (Table S12), and an ABI PRISM 7900HT Sequence Detection System (Applied Biosystems). Mean C_T_ values were normalized to the *spo0A^−^* (negative control) sample and further normalized to *rpoB*. Relative expression values reported are representative of three biological replicates.

## Supporting Information

Figure S1 Construction of *spo0A^−^*, *sigE^−^*, *sigG^−^*, *sigK^−^* and *sigF^−^* mutants in *C. difficile*. (A) Schematic of the group II intron targeted gene disruption system. (B) Colony PCR analysis of *spo0A^−^*, *sigE^−^*, *sigG^−^*, *sigK^−^*, and *sigF^−^* strains compared to wild type (WT) using primers that flank the gene of interest. The group II intron insertion is ∼2 kb.
(TIF)

Figure S2 Phase contrast microscopy of strains used for RNA-Seq analyses. Phase-contrast microscopy of WT, *spo0A^−^*, *sigF^−^*, *sigE^−^*, *sigG^−^*, and *sigK^−^* strains grown on sporulation media for 18 hrs. White triangles mark mature phase-bright spores, and black triangles indicate immature phase-dark forespores. Phase-bright spores were not observed in the sigma factor mutants. The percentage of sporulating cells, defined as containing phase-dark forespores, phase-bright forespores, or free spores, is shown for each of the biological replicates. Scale bar represents 5 µm.
(TIF)

Figure S3 Prevalence of *sigG^−^* phenotypes. TEM of *sigG* ***^−^*** mutants during growth on sporulation media. *sigG^−^* mutant cells (n = 80) containing a forespore with a putative coat layer (black triangle) were scored for the presence of a ruffled membranes (98%), a double forespore compartment (21%), and incomplete engulfment (87%). A black arrow indicates incomplete membrane fission during engulfment, and a white arrow indicates septum-like structures in the forespore. No cortex was detected in any of the *sigG^−^* mutant cells analyzed. All wildtype forespores surrounded by a coat layer had completed engulfment (n = 60 cells, data not shown). Scale bar represents 500 nm.
(TIF)

Figure S4 Plasmid complementation rescues spore formation in *C. difficile* sigma factor mutants. (A) Phase-contrast microscopy of *sigF^−^*, *sigE^−^*, and *sigG^−^* strains grown on sporulation media for 30 hrs and the *sigK^−^* strain for 42 hrs. The strains carry either empty pMTL83151 or pMTL84151 vector [48] or pMTL8151-*sigE*, *sigG*, or *sigK* genes, respectively, or pMTL84151-*sigF*, expressed from their native promoters. White triangles mark mature phase-bright spores, and black triangles indicate immature phase-dark forespores. Phase-bright spores were not observed in the sigma factor mutants. Scale bar represents 5 µm. (B) Western blot analyses of wildtype (WT), *spo0A^−^*, *sigF^−^*, *sigE^−^*, *sigG^−^*, and *sigK^−^* carrying either empty pMTL83151 vector (EV) or a complementation construct using antibodies raised against σ^F^, σ^E^, σ^G^, and σ^K^. Spo0A levels were also measured to compare the induction of sporulation between strains [37], [86]. The asterisk demarcates a non-specific band observed in all strains tested. (C) Sporulation efficiencies determined by heat resistance assays of complementation strains *sigF^−^/*pMTL84151*-sigF^+^, sigE^−^*/pMTL83151-*sigE^+^*, *sigG^−^*/pMTL83151-*sigG^+^*, and *sigK^−^*/pMTL83151-*sigK^+^* relative to wildtype. No heat-resistant spores were detected in mutant strains carrying empty vector.
(TIF)

Figure S5 Plasmid complementation rescues coat and cortex formation in sigma factor mutants. The *sigF^−^*, *sigE^−^*, and *sigG^−^* strains were grown on sporulation media for 28 hrs, while the *sigK^−^* strains were grown for 40 hrs. The strains carry either empty pMTL83151 (or pMTL84151 vector for *sigF^−^*, [48]) or *sigF*, *sigE*, *sigG*, or *sigK* genes, respectively, expressed from their native promoters. White triangles indicate cortex and black triangles indicate coat. Scale bar represents 250 nm.
(TIF)

Figure S6 Analysis of sigma factor regulation network topology in *C. difficile*. Circles represent genes and arrows indicate activation of expression (see Text S1). *s0* = *spo0A*, *e* = *sigE*, *g* = *sigG* and *k* = *sigK*. (A) Network topology proposed for *B. subtilis*. (B) Expression profile of a query sporulation gene, *q*, among the *spo0A^−^*, *sigE* ^−^, *sigG^−^*, and *sigK^−^* mutants, illustrated in the context of the *B. subtilis* network topology. The circles of the network topology (A) represent genes whereas the columns in the heat map (Figure 5) represent strains; by coloring the circles of the topology using the expression level of *q* in the associated knockout strain (red = low; green = high), the consistency of the expression profile (B) with the network topology can be readily evaluated. More precisely, a network is consistent with the expression profile of *q* if and only if the red circles form the path between s0 (*spo0A^−^*) and *q*. The example is inconsistent with the *B. subtilis* topology because there is no way to attach *q* that will result in a consistent topology. (C and D) Expression profile for σ^G^- and σ^E^-dependent genes, respectively, in the proposed topology for *C. difficile*. Red coloring of a gene indicates that *q* is downregulated when the former is knocked out. For example, in (D) *q* is downregulated in s0 (*spo0A^−^*) and *e* (*sigE^−^*) mutants but upregulated in the *g* (*sigG^−^*) and *k* (*sigK^−^*) mutants.
(TIF)

Figure S7 Statistical analysis rejects the *B. subtilis* network topology for sporulation sigma factor regulation. Each gene was fit to models associated with the null, σ^G^-, and σ^E^-dependent transcriptome models to obtain p-values (see Text S1).
(TIF)

Table S1 Quantitation of sporulating cell phenotypes. *C. difficile* strains JIR8094 (WT), *sigF^−^*, *sigE^−^*, *sigG^−^*, and *sigK^−^* exhibit asynchronous sporulation when grown on sporulation induction media for 18 hours. Phase-contrast microscopy and fluorescence light microscopy using the membrane stain FM4-64 and the nucleic acid dye Hoechst was used to analyze sporulation in the indicated strains. A cell was deemed positive for sporulation if it fell into one of five criteria: (1) a polar septum was detected by FM4-64, but the forespore did not stain with Hoechst; (2) Polar septum was detected by FM4-64, and the forespore stained with Hoechst; (3) a phase-dark forespore stained with both FM4-64 and Hoechst; (4) A phase-dark forespore stained with FM4-64 but not Hoechst, or (5) a phase-bright forespore was visible, but it failed to stain with either FM4-64 or Hoechst. The percent of total sporulating cells reflects the number of events that fall within the stated criteria relative to the total number of cells. A total of 200 cells were counted for each strain. s*po0A^−^* cells were not evaluated for sporulation staining.
(DOCX)

Table S2 Summary of RNA-Seq data analysis. Strain.Rep refers to the strain name followed by the replicate number. Three biological replicates were processed for RNA-Seq analyses for each strain. WT refers to the parental JIR8094 strain. The total number of reads obtained and mapped to the genome is indicated. % mapped refers to the percentage of reads that mapped to the *C. difficile* genome. >90% of the unmapped reads did not map to sequences in the NCBI database and appear to derive from spurious amplification products during library construction.
(DOCX)

Table S3 Spo0A-dependent (activated) genes. ^†^ Two factors are listed in the table for genes whose expression was dependent on both σ^E^ and σ^G^ (adjusted p-value≤0.05, log_2_FC≤−2). *Dep*. indicates the most downstream sigma factor on which gene expression depends upon. *BM* refers to base mean, the mean of the counts after they were divided by the size factors to adjust for different sequencing depths. This value is the mean for the sample relative to wild type. *log_2_FC* denotes log_2_fold-change. A negative value indicates that the gene was downregulated relative to wild type. ∧ Indicates that gene product was detected in Lawley *et al.* proteomic analysis of purified spores [70]. *−Inf* indicates that no transcript was detected in the mutant relative to wild type. See Text S2 for the references.
(DOCX)

Table S4 Sporulation-related genes. ^†^ Two factors are listed in the table for genes whose expression was dependent on both σ^E^ and σ^G^ (adjusted p-value≤0.05, log_2_FC≤−2). *Dep.* indicates the most downstream sigma factor on which gene expression depends upon. *BM* refers to base mean, the mean of the counts after they were divided by the size factors to adjust for different sequencing depths. This value is the mean for the sample relative to wild type. *log_2_FC* denotes log_2_fold-change. A negative value indicates that the gene was downregulated relative to wild type. ∧ Indicates that gene product was detected in Lawley *et al.* proteomic analysis of purified spores [70]. *−Inf* indicates that no transcript was detected in the mutant relative to wild type. See Text S2 for the references.
(DOCX)

Table S5 σ^F^-dependent genes. ^†^ Two factors are listed in the table for genes whose expression was dependent on both σ^E^ and σ^G^ (adjusted p-value≤0.05, log_2_FC≤−2). *Dep.* indicates the most downstream sigma factor on which gene expression depends upon. *BM* refers to base mean, the mean of the counts after they were divided by the size factors to adjust for different sequencing depths. This value is the mean for the sample relative to wild type. *log_2_FC* denotes log_2_fold-change. A negative value indicates that the gene was downregulated relative to wild type. ∧ Indicates that gene product was detected in Lawley *et al.* proteomic analysis of purified spores [70]. *−Inf* indicates that no transcript was detected in the mutant relative to wild type. See Text S2 for the references.
(DOCX)

Table S6 σ^E^-dependent genes. ^†^ Two factors are listed in the table for genes whose expression was dependent on both σ^E^ and σ^G^ (adjusted p-value≤0.05, log_2_FC≤−2). *Dep.* indicates the most downstream sigma factor on which gene expression depends upon. *BM* refers to base mean, the mean of the counts after they were divided by the size factors to adjust for different sequencing depths. This value is the mean for the sample relative to wild type. *log_2_FC* denotes log_2_fold-change. A negative value indicates that the gene was downregulated relative to wild type. ∧ Indicates that gene product was detected in Lawley *et al.* proteomic analysis of purified spores [70]. *−Inf* indicates that no transcript was detected in the mutant relative to wild type. See Text S2 for the references.
(DOCX)

Table S7 σ^G^-dependent genes. ^†^ Two factors are listed in the table for genes whose expression was dependent on both σ^E^ and σ^G^ (adjusted p-value≤0.05, log_2_FC≤−2). *Dep.* indicates the most downstream sigma factor on which gene expression depends upon. *BM* refers to base mean, the mean of the counts after they were divided by the size factors to adjust for different sequencing depths. This value is the mean for the sample relative to wild type. *log_2_FC* denotes log_2_fold-change. A negative value indicates that the gene was downregulated relative to wild type. ∧ Indicates that gene product was detected in Lawley *et al.* proteomic analysis of purified spores [70]. *−Inf* indicates that no transcript was detected in the mutant relative to wild type. See Text S2 for the references.
(DOCX)

Table S8 σ^K^-dependent genes. *Dep.* indicates the most downstream sigma factor on which gene expression depends upon. *BM* refers to base mean, the mean of the counts after they were divided by the size factors to adjust for different sequencing depths. This value is the mean for the sample relative to wild type. *log_2_FC* denotes log_2_fold-change. A negative value indicates that the gene was downregulated relative to wild type. ∧ Indicates that gene product was detected in Lawley *et al.* proteomic analysis of purified spores [70]. *−Inf* indicates that no transcript was detected in the mutant relative to wild type. See Text S2 for the references.
(DOCX)

Table S9 Genes induced in a Spo0A-, σ^F^-, σ^E^-, σ^G^- and σ^K^-dependent manner during growth on sporulation media. ^†^ Two factors are listed in the table for genes whose expression was dependent on both σ^E^ and σ^G^ (adjusted p-value≤0.05, log_2_FC≤−2). *Dep.* indicates the most downstream sigma factor on which gene expression depends upon. *BM* refers to base mean, the mean of the counts after they were divided by the size factors to adjust for different sequencing depths. This value is the mean for the sample relative to wild type. *log_2_FC* denotes log_2_fold-change. A negative value indicates that the gene was downregulated relative to wild type. ∧ Indicates that gene product was detected in Lawley *et al.* proteomic analysis of purified spores [70]. *−Inf* indicates that no transcript was detected in the mutant relative to wild type. See Text S2 for the references.
(DOCX)

Table S10 Genes encoding hypothetical proteins induced during sporulation. ^†^ Two factors are listed in the table for genes whose expression was dependent on both σ^E^ and σ^G^ (adjusted p-value≤0.05, log_2_FC≤−2). *Dep.* indicates the most downstream sigma factor on which gene expression depends upon. *BM* refers to base mean, the mean of the counts after they were divided by the size factors to adjust for different sequencing depths. This value is the mean for the sample relative to wild type. *log_2_FC* denotes log_2_fold-change. A negative value indicates that the gene was downregulated relative to wild type. ∧ Indicates that gene product was detected in Lawley *et al.* proteomic analysis of purified spores [70]. *−Inf* indicates that no transcript was detected in the mutant relative to wild type. See Text S2 for the references.
(DOCX)

Table S11 *E. coli* strains used in this study.
(DOCX)

Table S12 Plasmids used in this study.
(DOCX)

Table S13 Primers used in this study.
(DOCX)

Table S14 Genes whose expression is increased in the absence of *spo0A*. BM refers to base mean, the mean of the counts after they were divided by the size factors to adjust for different sequencing depths. This value is the mean for the sample relative to wild type. log_2_FC denotes log_2_fold-change. A positive value indicates that the gene was upregulated in the *spo0A^−^* mutant relative to wild type. +Inf indicates that no transcript was detected in wild type relative to the *spo0A* ^−^ mutant.
(DOCX)

Text S1 Sigma factor regulation network topology in *C. difficile*.
(DOCX)

Text S2 References found in Tables S3, S4, S5, S6, S7, S8, S9 and S10.
(DOCX)
