# Supplementary figures and images for: Global Analysis of the Sporulation Pathway of Clostridium difficile
Source: PLoS Genet. 2013 Aug 8;9(8):e1003660. doi: 10.1371/journal.pgen.1003660 (PMC3738446; doi:10.1371/journal.pgen.1003660)

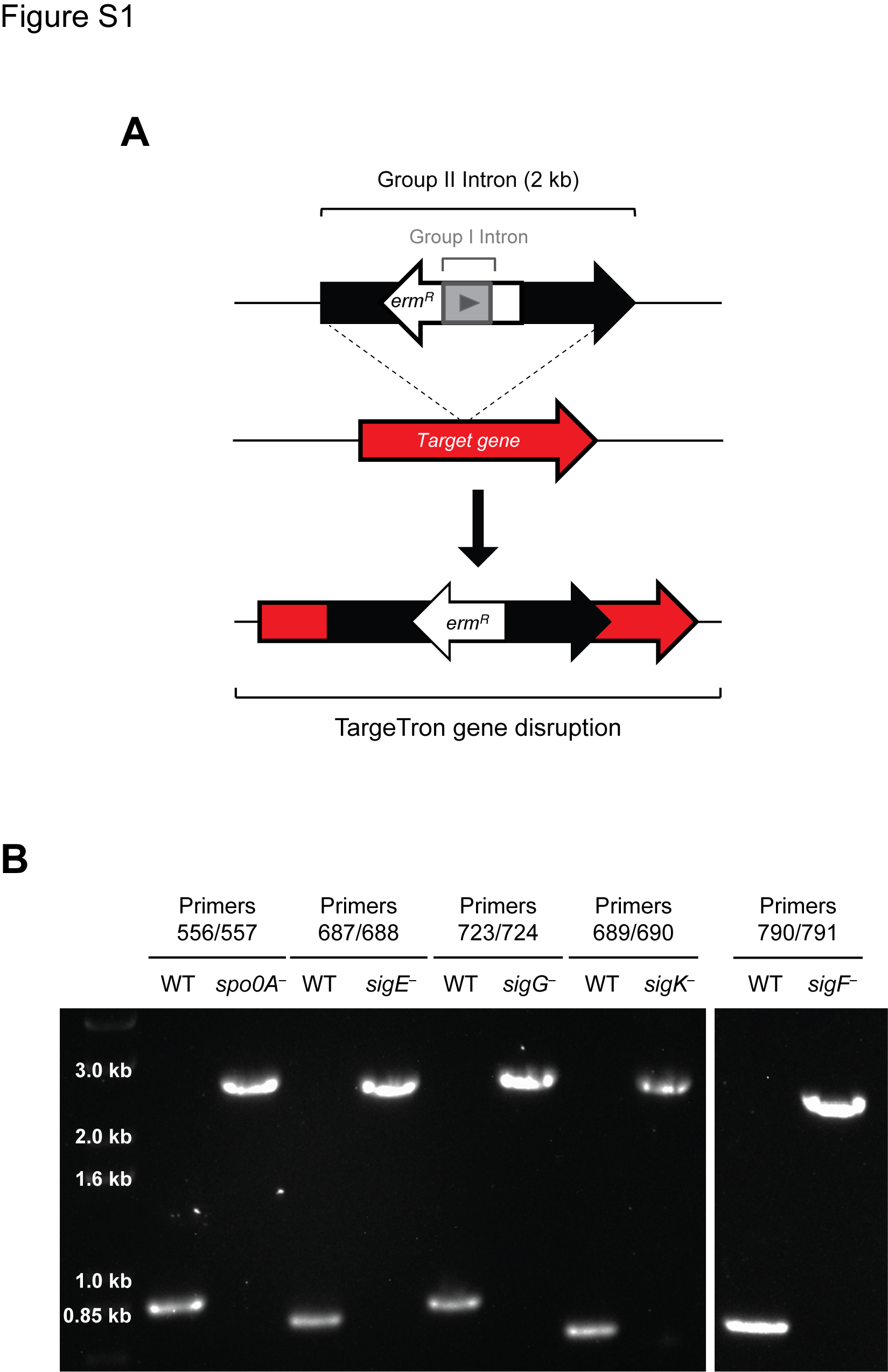

Supplement: Figure S1 — Construction of spo0A−, sigE−, sigG−, sigK− and sigF− mutants in C. difficile. (A) Schematic of the group II intron targeted gene disruption system. (B) Colony PCR analysis of spo0A−, sigE−, sigG−, sigK−, and sigF− strains compared to wild type (WT) using primers that flank the gene of interest. The group II intron insertion is ∼2 kb. (TIF) [file pgen.1003660.s001.tif]

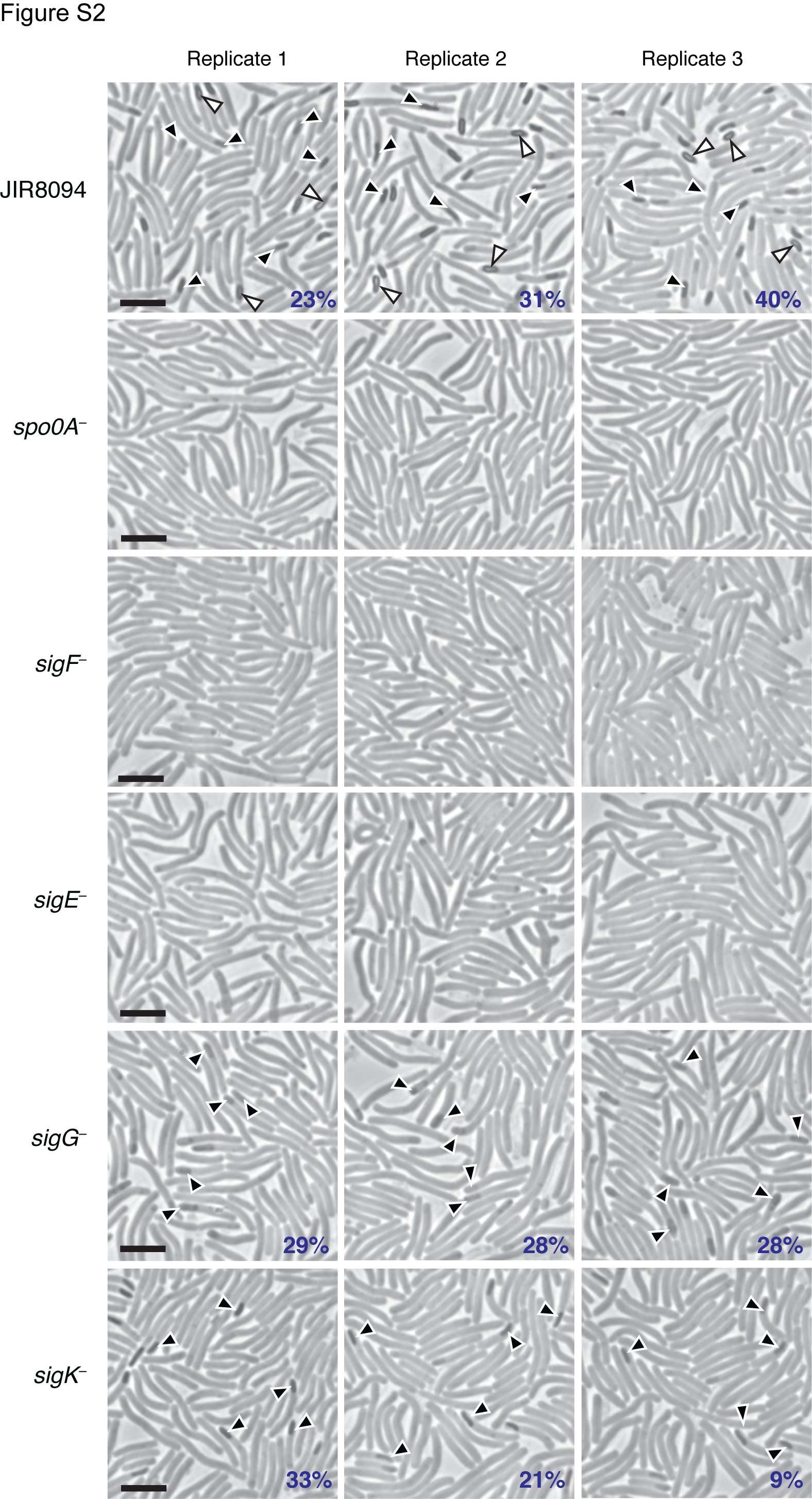

Supplement: Figure S2 — Phase contrast microscopy of strains used for RNA-Seq analyses. Phase-contrast microscopy of WT, spo0A−, sigF−, sigE−, sigG−, and sigK− strains grown on sporulation media for 18 hrs. White triangles mark mature phase-bright spores, and black triangles indicate immature phase-dark forespores. Phase-bright spores were not observed in the sigma factor mutants. The percentage of sporulating cells, defined as containing phase-dark forespores, phase-bright forespores, or free spores, is shown for each of the biological replicates. Scale bar represents 5 µm. (TIF) [file pgen.1003660.s002.tif]

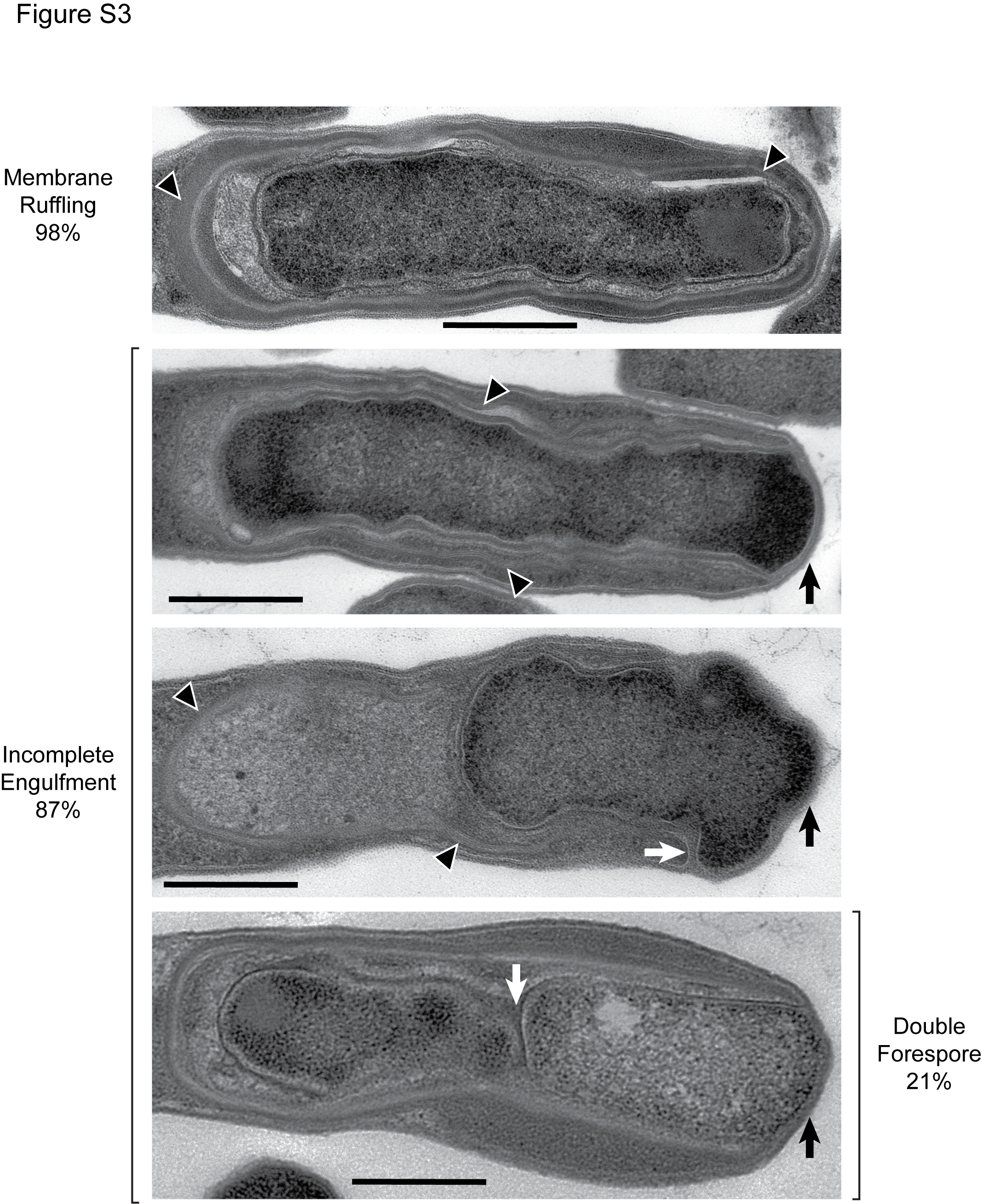

Supplement: Figure S3 — Prevalence of sigG− phenotypes. TEM of sigG − mutants during growth on sporulation media. sigG− mutant cells (n = 80) containing a forespore with a putative coat layer (black triangle) were scored for the presence of a ruffled membranes (98%), a double forespore compartment (21%), and incomplete engulfment (87%). A black arrow indicates incomplete membrane fission during engulfment, and a white arrow indicates septum-like structures in the forespore. No cortex was detected in any of the sigG− mutant cells analyzed. All wildtype forespores surrounded by a coat layer had completed engulfment (n = 60 cells, data not shown). Scale bar represents 500 nm. (TIF) [file pgen.1003660.s003.tif]

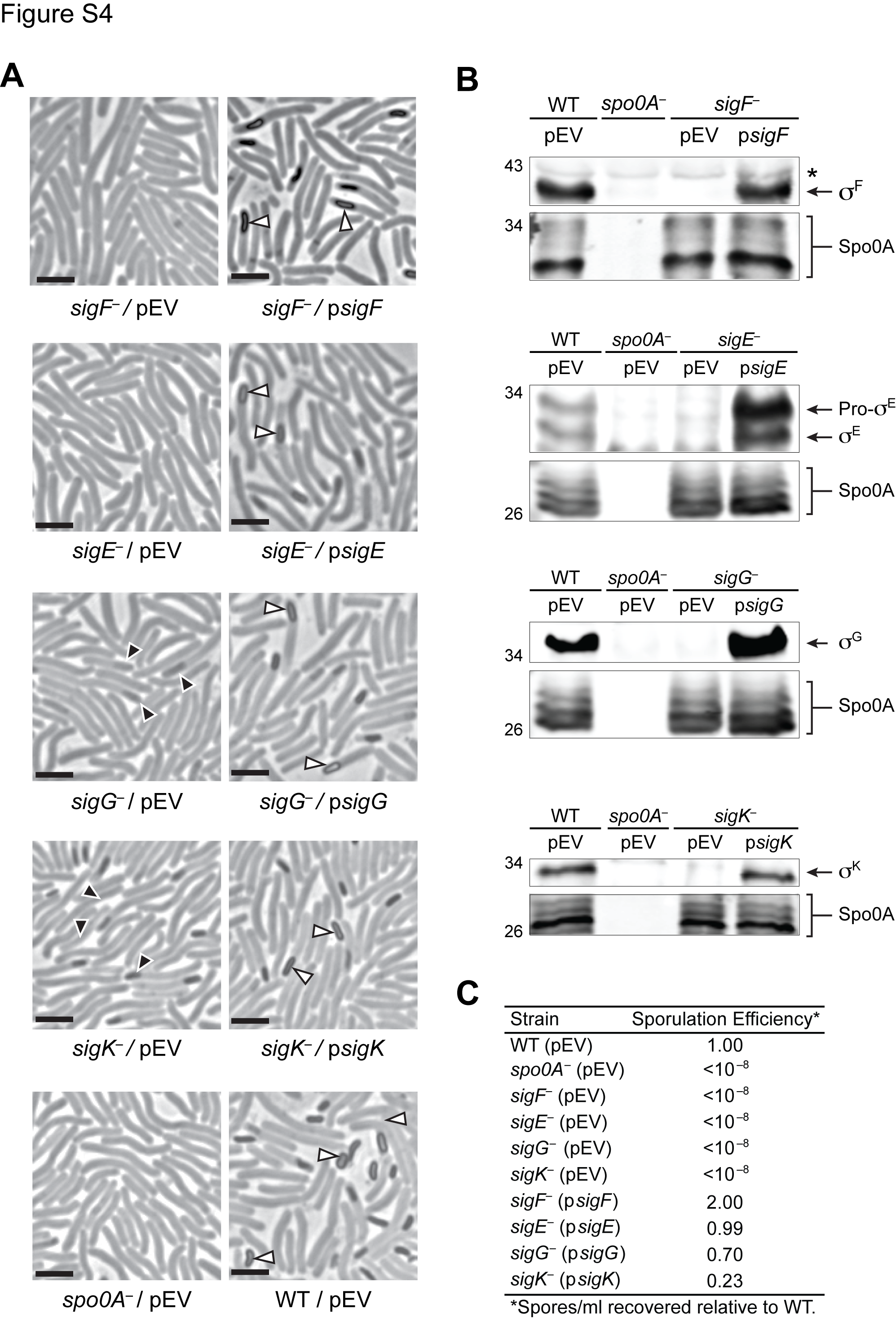

Supplement: Figure S4 — Plasmid complementation rescues spore formation in C. difficile sigma factor mutants. (A) Phase-contrast microscopy of sigF−, sigE−, and sigG− strains grown on sporulation media for 30 hrs and the sigK− strain for 42 hrs. The strains carry either empty pMTL83151 or pMTL84151 vector [48] or pMTL8151-sigE, sigG, or sigK genes, respectively, or pMTL84151-sigF, expressed from their native promoters. White triangles mark mature phase-bright spores, and black triangles indicate immature phase-dark forespores. Phase-bright spores were not observed in the sigma factor mutants. Scale bar represents 5 µm. (B) Western blot analyses of wildtype (WT), spo0A−, sigF−, sigE−, sigG−, and sigK− carrying either empty pMTL83151 vector (EV) or a complementation construct using antibodies raised against σF, σE, σG, and σK. Spo0A levels were also measured to compare the induction of sporulation between strains [37], [86]. The asterisk demarcates a non-specific band observed in all strains tested. (C) Sporulation efficiencies determined by heat resistance assays of complementation strains sigF−/pMTL84151-sigF+, sigE−/pMTL83151-sigE+, sigG−/pMTL83151-sigG+, and sigK−/pMTL83151-sigK+ relative to wildtype. No heat-resistant spores were detected in mutant strains carrying empty vector. (TIF) [file pgen.1003660.s004.tif]

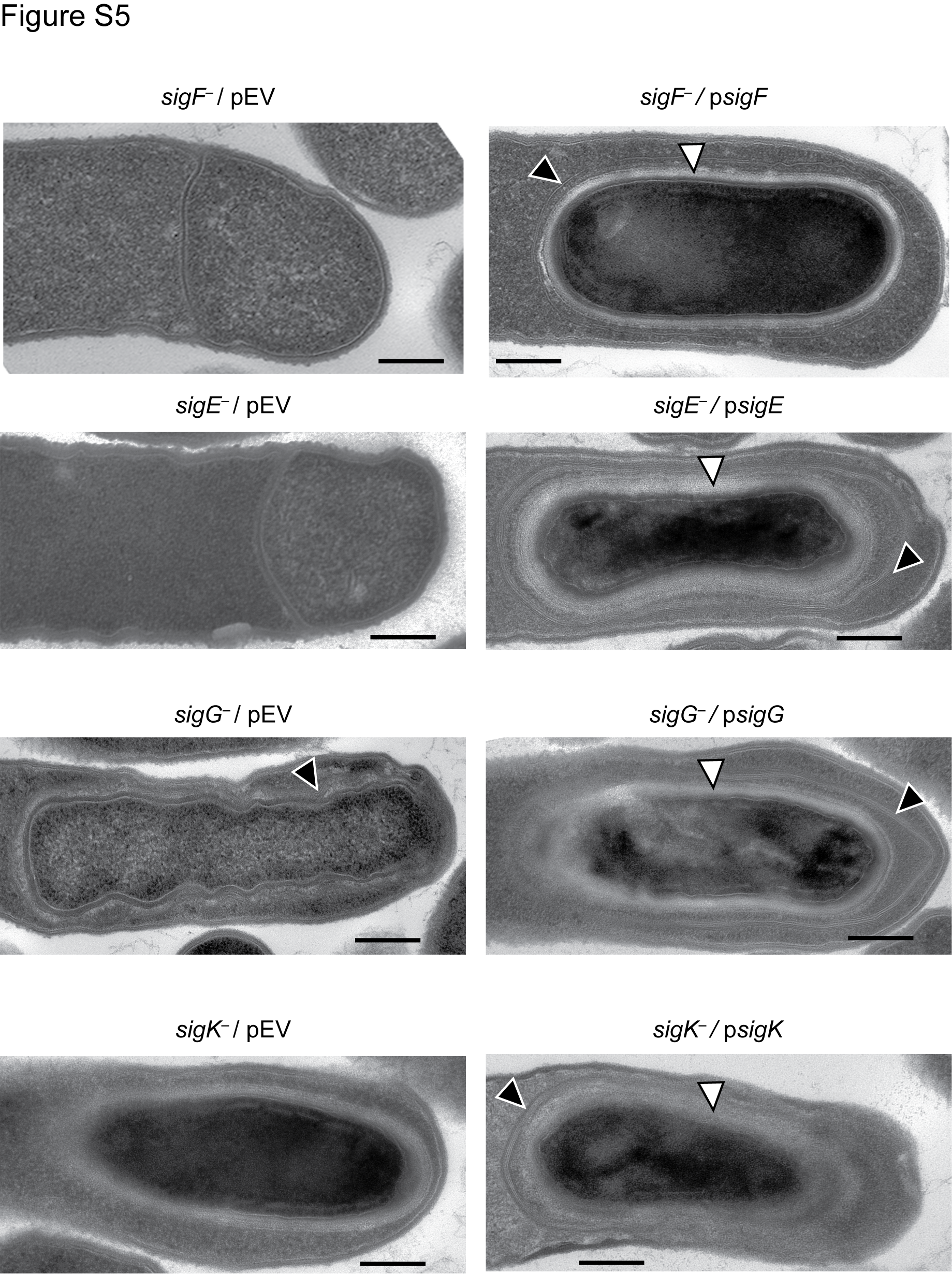

Supplement: Figure S5 — Plasmid complementation rescues coat and cortex formation in sigma factor mutants. The sigF−, sigE−, and sigG− strains were grown on sporulation media for 28 hrs, while the sigK− strains were grown for 40 hrs. The strains carry either empty pMTL83151 (or pMTL84151 vector for sigF−, [48]) or sigF, sigE, sigG, or sigK genes, respectively, expressed from their native promoters. White triangles indicate cortex and black triangles indicate coat. Scale bar represents 250 nm. (TIF) [file pgen.1003660.s005.tif]

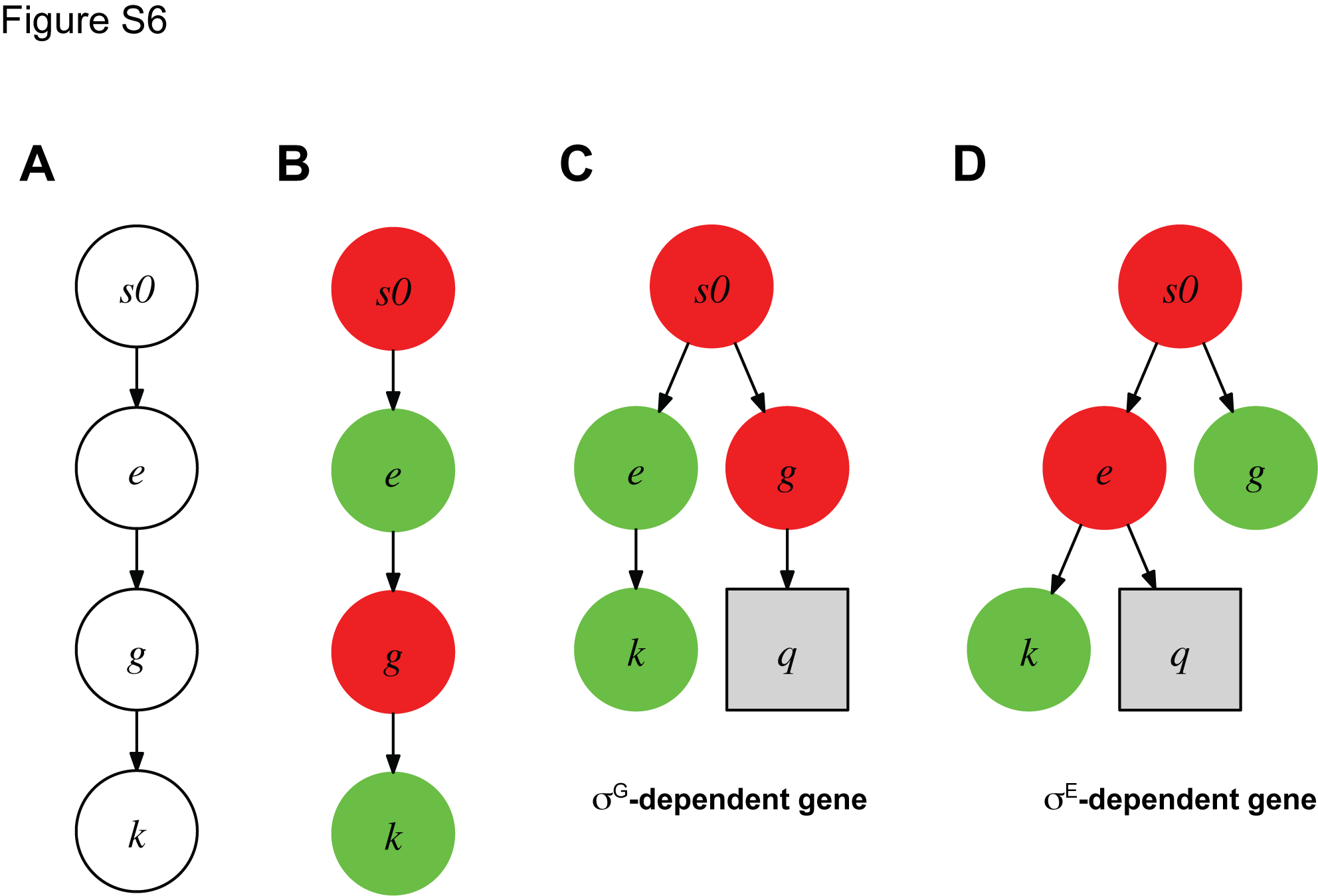

Supplement: Figure S6 — Analysis of sigma factor regulation network topology in C. difficile. Circles represent genes and arrows indicate activation of expression (see Text S1). s0 = spo0A, e = sigE, g = sigG and k = sigK. (A) Network topology proposed for B. subtilis. (B) Expression profile of a query sporulation gene, q, among the spo0A−, sigE −, sigG−, and sigK− mutants, illustrated in the context of the B. subtilis network topology. The circles of the network topology (A) represent genes whereas the columns in the heat map (Figure 5) represent strains; by coloring the circles of the topology using the expression level of q in the associated knockout strain (red = low; green = high), the consistency of the expression profile (B) with the network topology can be readily evaluated. More precisely, a network is consistent with the expression profile of q if and only if the red circles form the path between s0 (spo0A−) and q. The example is inconsistent with the B. subtilis topology because there is no way to attach q that will result in a consistent topology. (C and D) Expression profile for σG- and σE-dependent genes, respectively, in the proposed topology for C. difficile. Red coloring of a gene indicates that q is downregulated when the former is knocked out. For example, in (D) q is downregulated in s0 (spo0A−) and e (sigE−) mutants but upregulated in the g (sigG−) and k (sigK−) mutants. (TIF) [file pgen.1003660.s006.tif]

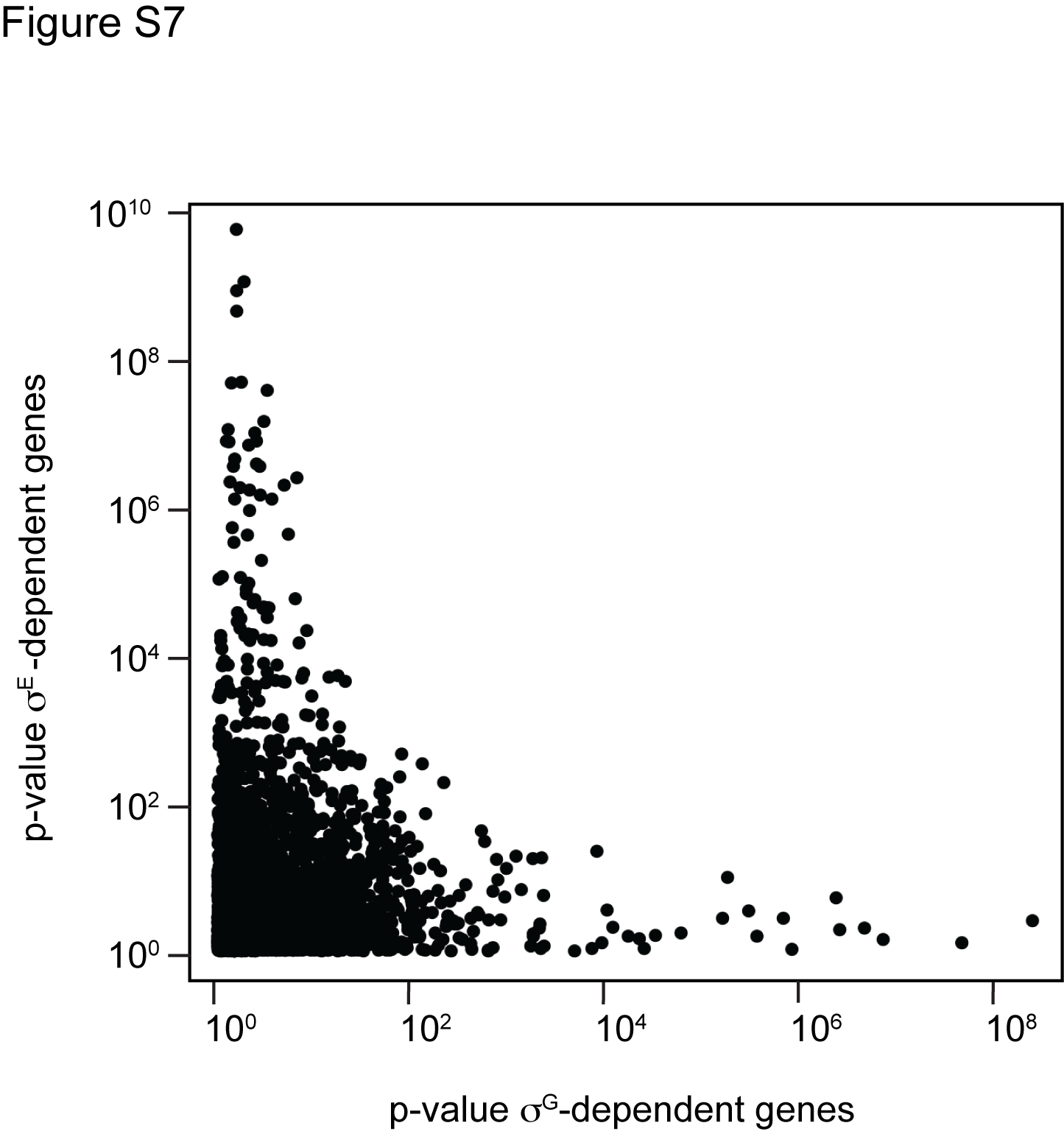

Supplement: Figure S7 — Statistical analysis rejects the B. subtilis network topology for sporulation sigma factor regulation. Each gene was fit to models associated with the null, σG-, and σE-dependent transcriptome models to obtain p-values (see Text S1). (TIF) [file pgen.1003660.s007.tif]
