# Supplementary material for: Global Analysis of the Sporulation Pathway of Clostridium difficile
Source: PLoS Genet. 2013 Aug 8;9(8):e1003660. doi: 10.1371/journal.pgen.1003660 (PMC3738446; doi:10.1371/journal.pgen.1003660)
Supplement: Table S1 — Quantitation of sporulating cell phenotypes. C. difficile strains JIR8094 (WT), sigF−, sigE−, sigG−, and sigK− exhibit asynchronous sporulation when grown on sporulation induction media for 18 hours. Phase-contrast microscopy and fluorescence light microscopy using the membrane stain FM4-64 and the nucleic acid dye Hoechst was used to analyze sporulation in the indicated strains. A cell was deemed positive for sporulation if it fell into one of five criteria: (1) a polar septum was detected by FM4-64, but the forespore did not stain with Hoechst; (2) Polar septum was detected by FM4-64, and the forespore stained with Hoechst; (3) a phase-dark forespore stained with both FM4-64 and Hoechst; (4) A phase-dark forespore stained with FM4-64 but not Hoechst, or (5) a phase-bright forespore was visible, but it failed to stain with either FM4-64 or Hoechst. The percent of total sporulating cells reflects the number of events that fall within the stated criteria relative to the total number of cells. A total of 200 cells were counted for each strain. spo0A− cells were not evaluated for sporulation staining. (DOCX) [file pgen.1003660.s008.docx]

**Table S1. Quantitation of sporulating cell phenotypes.**

| **Percentage of cells with phenotype** | **WT** | ***sigF^–^*** | ***sigE^–^*** | ***sigG^–^*** | ***sigK^–^*** |
| --- | --- | --- | --- | --- | --- |
| 1. Polar septum detected by FM4-64; forespore does not stain with Hoechst | 0 | 2 | 2 | 2 | 0 |
| 2. Polar septum detected by FM4-64; forespore stains with Hoechst | 7 | 39 | 22 | 5 | 6 |
| 3. Phase-dark forespore detected with FM4-64 and Hoechst | 7 | 0 | 0 | 18 | 4 |
| 4. Phase-dark forespore detected with FM4-64 but not Hoechst | 7 | 0 | 0 | 1 | 8 |
| 5. Phase-bright forespore: no FM4-64 or Hoechst staining | 4 | 0 | 0 | 0 | 0 |
| **% Total Sporulating Cells** | **25** | **41** | **24** | **26** | **18** |

*C. difficile* strains JIR8094 (WT), *sigF^–^*, *sigE^–^*, *sigG^–^,* and *sigK^–^* exhibit asynchronous sporulation when grown on sporulation induction media for 18 hours. Phase-contrast microscopy and fluorescence light microscopy using the membrane stain FM4-64 and the nucleic acid dye Hoechst was used to analyze sporulation in the indicated strains. A cell was deemed positive for sporulation if it fell into one of five criteria: (1) a polar septum was detected by FM4-64, but the forespore did not stain with Hoechst; **(**2) Polar septum was detected by FM4-64, and the forespore stained with Hoechst; **(**3) a phase-dark forespore stained with both FM4-64 and Hoechst; (4) A phase-dark forespore stained with FM4-64 but not Hoechst, or (5) a phase-bright forespore was visible, but it failed to stain with either FM4-64 or Hoechst. The percent of total sporulating cells reflects the number of events that fall within the stated criteria relative to the total number of cells. A total of 200 cells were counted for each strain. s*po0A^–^* cells were not evaluated for sporulation staining.
