# Supplementary material for: Global Analysis of the Sporulation Pathway of Clostridium difficile
Source: PLoS Genet. 2013 Aug 8;9(8):e1003660. doi: 10.1371/journal.pgen.1003660 (PMC3738446; doi:10.1371/journal.pgen.1003660)
Supplement: Table S2 — Summary of RNA-Seq data analysis. Strain.Rep refers to the strain name followed by the replicate number. Three biological replicates were processed for RNA-Seq analyses for each strain. WT refers to the parental JIR8094 strain. The total number of reads obtained and mapped to the genome is indicated. % mapped refers to the percentage of reads that mapped to the C. difficile genome. >90% of the unmapped reads did not map to sequences in the NCBI database and appear to derive from spurious amplification products during library construction. (DOCX) [file pgen.1003660.s009.docx]

**Table S2. Summary of RNA-Seq data analysis**

| **Strain.Rep** | **Total Reads** | **Mapped Reads** | **Unmapped Reads** | **% Mapped** | **Read Pairs Analyzed** |
| --- | --- | --- | --- | --- | --- |
| WT.1 | 113,947,550 | 14,264,583 | 99,682,967 | 12.5 | 6,852,557 |
| WT.2 | 80,537,138 | 15,040,885 | 65,496,253 | 18.7 | 7,226,927 |
| WT.3 | 16,743,541 | 1,659,364 | 15,084,177 | 9.9 | 792,548 |
| *spo0A^–^*.1 | 71,253,577 | 10,567,744 | 60,685,833 | 14.8 | 5,065,585 |
| *spo0A^–^*.2 | 112,106,468 | 16,274,770 | 95,831,698 | 14.5 | 7,847,403 |
| *spo0A^–^*.3 | 73,104,929 | 8,568,662 | 64,536,267 | 11.7 | 4,100,037 |
| *sigF–.1* | 69,900,223 | 10,521,022 | 59,379,201 | 15.1 | 5,034,154 |
| *sigF–.2* | 51,963,822 | 11,201,068 | 40,762,754 | 21.6 | 5,358,384 |
| *sigF–.3* | 143,499,590 | 16,357,171 | 127,142,419 | 11.4 | 7,804,423 |
| *sigE^–^*.1 | 102,404,015 | 14,617,494 | 87,786,521 | 14.3 | 7,000,863 |
| *sigE^–^*.2 | 120,157,280 | 17,366,729 | 102,790,551 | 14.5 | 8,332,138 |
| *sigE^–^*.3 | 96,783,023 | 11,093,658 | 85,689,365 | 11.5 | 5,301,435 |
| *sigG^–^*.1 | 98,108,051 | 15,169,368 | 82,938,683 | 15.5 | 7,278,297 |
| *sigG^–^*.2 | 83,085,195 | 18,162,565 | 64,922,630 | 21.9 | 8,711,979 |
| *sigG^–^*.3 | 81,216,196 | 16,303,158 | 64,913,038 | 20.1 | 7,852,446 |
| *sigK^–^*.1 | 96,155,400 | 12,270,298 | 83,885,102 | 12.8 | 5,730,184 |
| *sigK^–^*.2 | 101,062,343 | 10,704,279 | 90,358,064 | 10.6 | 5,029,183 |
| *sigK^–^*.3 | 118,029,725 | 11,103,107 | 106,926,618 | 9.4 | 5,176,107 |

Strain.Rep refers to the strain name followed by the replicate number. Three biological replicates were processed for RNA-Seq analyses for each strain. WT refers to the parental JIR8094 strain. The total number of reads obtained and mapped to the genome is indicated. % mapped refers to the percentage of reads that mapped to the *C. difficile* genome. The >90% of the unmapped reads did not map to sequences in the NCBI database and appear to derive from spurious amplification products during library construction.
