# Supplementary material for: Global Analysis of the Sporulation Pathway of Clostridium difficile
Source: PLoS Genet. 2013 Aug 8;9(8):e1003660. doi: 10.1371/journal.pgen.1003660 (PMC3738446; doi:10.1371/journal.pgen.1003660)
Supplement: Table S3 — Spo0A-dependent (activated) genes. † Two factors are listed in the table for genes whose expression was dependent on both σE and σG (adjusted p-value≤0.05, log2FC≤−2). Dep. indicates the most downstream sigma factor on which gene expression depends upon. BM refers to base mean, the mean of the counts after they were divided by the size factors to adjust for different sequencing depths. This value is the mean for the sample relative to wild type. log2FC denotes log2fold-change. A negative value indicates that the gene was downregulated relative to wild type. ∧ Indicates that gene product was detected in Lawley et al. proteomic analysis of purified spores [70]. −Inf indicates that no transcript was detected in the mutant relative to wild type. See Text S2 for the references. (DOCX) [file pgen.1003660.s010.docx]

**Table S3. Spo0A-dependent (activated) genes.**

|  |  |  |  | **Spo0A** | | | σ^F^ | | | **σ^E^** | | | **σ^G^** | | | **σ^K^** | | |
| --- | --- | --- | --- | --- | --- | --- | --- | --- | --- | --- | --- | --- | --- | --- | --- | --- | --- | --- |
| **Dep.**^†^ | **Name** | **locus_tag** | **description** | **BM** | **log_2_FC** | **adjP** | **BM** | **log_2_FC** | **adjP** | **BM** | **log_2_FC** | **adjP** | **BM** | **log_2_FC** | **adjP** | **BM** | **log_2_FC** | **adjP** |
| ^σ^K^ | *CD1067* | CD630_10670 | hypothetical protein | 9888 | -7.6 | 1.3x10^-60^ | 11923 | -2.7 | 3.9x10^-14^ | 10380 | -6.1 | 1.5x10^-50^ | 21003 | -0.2 | 1 | 8776 | -4.7 | 2.4x10^-32^ |
| σ^K^ | *bclA3* | CD630_33490 | exosporium glycoprotein BclA3 | 2155 | -6.1 | 5.9x10^-11^ | 2467 | -3.3 | 4.9x10^-5^ | 2247 | -5.7 | 1.3x10^-10^ | 4455 | -0.2 | 1 | 1867 | -5.5 | 2.2x10^-9^ |
| ^σ^K^ | *CD1433* | CD630_14330 | peroxiredoxin/chitinase (coat protein "CotE," [[1](#_ENREF_1)]) | 2018 | -7.7 | 1.4x10^-56^ | 2343 | -3.2 | 2.2x10^-18^ | 2112 | -6.4 | 2.8x10^-74^ | 3959 | -0.4 | 0.8 | 1753 | -5.8 | 1.2x10^-43^ |
| σ^K^ | *CD1063C* | CD630_10633 | hypothetical protein | 1552 | -7.5 | 4.2x10^-27^ | 1879 | -2.7 | 5.8x10^-7^ | 1632 | -6.0 | 2.9x10^-20^ | 3104 | -0.4 | 0.9 | 1369 | -4.9 | 3.4x10^-15^ |
| σ^K^ | *CD1063B* | CD630_10632 | hypothetical protein | 1496 | -7.5 | 4.7x10^-27^ | 1802 | -2.7 | 4.7x10^-7^ | 1569 | -6.2 | 3.8x10^-21^ | 3044 | -0.3 | 1 | 1317 | -5.1 | 2.2x10^-15^ |
| Spo0A | *CD2373* | CD630_23730 | CstA-like carbon starvation protein | 1110 | -4.2 | 7.5x10^-11^ | 2036 | -0.3 | 1 | 1833 | -0.6 | 0.5 | 2402 | 0.0 | 1 | 1584 | -0.4 | 1 |
| σ^E^ | *CD0311* | CD630_03110 | hypothetical protein | 926 | -6.8 | 2.3x10^-46^ | 1086 | -3.0 | 5.2x10^-16^ | 966 | -6.4 | 3.1x10^-68^ | 1715 | -0.6 | 0.4 | 1077 | -1.4 | 4.4x10^-3^ |
| ^σ^E^ | *sipL* | CD630_35670 | cell wall hydrolase (binds SpoIVA, [[2](#_ENREF_2)]) | 882 | -5.8 | 2.0x10^-36^ | 1036 | -2.9 | 8.3x10^-15^ | 937 | -4.6 | 9.5x10^-35^ | 1505 | -0.9 | 0.05 | 1072 | -1.2 | 0.2 |
| ^σ^K^ | *cotJB2* | CD630_24000 | spore coat peptide assembly protein CotJB2 | 797 | -8.5 | 2.2x10^-57^ | 953 | -2.9 | 9.6x10^-15^ | 833 | -6.8 | 4.6x10^-57^ | 1537 | -0.5 | 0.7 | 692 | -6.2 | 5.1x10^-44^ |
| ^Spo0A | *spo0A* | CD630_12140 | stage 0 sporulation protein A | 722 | -2.5 | 3.2x10^-10^ | 1266 | 0.0 | 1 | 1187 | -0.2 | 0.9 | 1302 | -0.2 | 1 | 1054 | 0.0 | 1 |
| ^σ^K^ | *cotJC2* | CD630_24010 | spore coat assembly protein CotJC2 ("CotD," [1]) | 720 | -7.8 | 7.4x10^-53^ | 869 | -2.7 | 1.2x10^-13^ | 753 | -6.4 | 5.3x10^-63^ | 1398 | -0.5 | 0.7 | 625 | -5.9 | 4.3x10^-42^ |
| ^σ^E^ | *spoIVA* | CD630_26290 | stage IV sporulation protein A | 665 | -7.3 | 3.6x10^-48^ | 764 | -3.4 | 3.1x10^-19^ | 695 | -6.5 | 1.1x10^-62^ | 1121 | -1.0 | 0.01 | 824 | -1.1 | 0.2 |
| ^Spo0A | *CD0514* | CD630_05140 | hemagglutinin/adhesin | 636 | -2.1 | 3.6x10^-7^ | 903 | -0.6 | 0.4 | 865 | -0.7 | 0.1 | 873 | -1.0 | 0.04 | 672 | -1.0 | 0.1 |
| σ^E^ | *alr2* | CD630_34630 | alanine racemase | 583 | -3.8 | 1.7x10^-20^ | 620 | -3.6 | 1.9x10^-21^ | 590 | -4.6 | 1.2x10^-42^ | 999 | -0.7 | 0.3 | 739 | -0.8 | 0.5 |
| σ^E^ | *CD3464* | CD630_34640 | hypothetical protein YdcC involved in sporulation [[3](#_ENREF_3)] | 557 | -4.6 | 1.5x10^-26^ | 609 | -3.6 | 2.3x10^-21^ | 574 | -5.0 | 2.3x10^-42^ | 965 | -0.7 | 0.2 | 698 | -0.9 | 0.2 |
| σ^K^ | *feoB* | CD630_15170 | ferrous iron transport protein B | 541 | -3.3 | 1.8x10^-12^ | 613 | -2.4 | 4.1x10^-8^ | 549 | -3.7 | 1.5x10^-14^ | 1125 | 0.0 | 1 | 475 | -3.0 | 3.0x10^-10^ |
| Spo0A | *CD3489* | CD630_34890 | oligoendopeptidase F, peptidase M3B family | 512 | -5.8 | 7.2x10^-37^ | 1112 | 0.1 | 1 | 1288 | 0.5 | 0.3 | 1151 | 0.0 | 1 | 760 | -0.4 | 0.9 |
| Spo0A | *spoIIE* | CD630_34900 | phosphoprotein phosphatase | 507 | -6.0 | 2.0x10^-25^ | 1224 | 0.4 | 0.7 | 1390 | 0.7 | 0.1 | 1238 | 0.3 | 1 | 751 | -0.4 | 0.9 |
| Spo0A | *spoVE* | CD630_26520 | cell division/stage V sporulation protein | 420 | -3.1 | 3.8x10^-15^ | 846 | 0.2 | 1 | 936 | 0.5 | 0.5 | 924 | 0.2 | 1 | 574 | -0.4 | 0.9 |
| ^σ^G^ | *sspA* | CD630_26880 | Small, acid-soluble spore protein alpha | 412 | -5.8 | 2.2x10^-25^ | 436 | -5.4 | 1.5x10^-24^ | 546 | -1.8 | 5.0x10^-5^ | 466 | -5.5 | 1.1x10^-24^ | 557 | -0.7 | 0.8 |
| σ^K^ | *CD1065* | CD630_10650 | hypothetical protein | 411 | -7.6 | 8.1x10^-32^ | 495 | -2.7 | 2.3x10^-10^ | 429 | -7.3 | 6.5x10^-33^ | 769 | -0.6 | 0.5 | 394 | -3.0 | 3.0x10^-11^ |
| Spo0A | *spoIIAB* | CD630_07710 | anti-σ^F^ factor | 374 | -4.4 | 8.1x10^-25^ | 863 | 0.4 | 0.8 | 881 | 0.5 | 0.6 | 874 | 0.2 | 1 | 571 | -0.2 | 1 |
| Spo0A | *murG* | CD630_26510 | UDP-NAG-NAM-(pentapeptide) pyrophosphoryl-undecaprenol NAG transferase | 370 | -3.2 | 2.7x10^-15^ | 763 | 0.2 | 1 | 788 | 0.3 | 0.8 | 803 | 0.2 | 1 | 512 | -0.3 | 1 |
| ^σ^G^ | *CD2112* | CD630_21120 | hypothetical protein | 368 | -4.9 | 3.2x10^-28^ | 391 | -4.5 | 1.6x10^-28^ | 633 | -0.5 | 0.4 | 414 | -4.9 | 1.4x10^-35^ | 411 | -1.5 | 5.2x10^-3^ |
| ^σ^K^ | *CD2399* | CD630_23990 | hypothetical protein (CotJA superfamily) | 364 | -8.9 | 4.2x10^-21^ | 427 | -3.1 | 4.0x10^-6^ | 379 | -7.6 | 3.5x10^-19^ | 702 | -0.5 | 0.9 | 314 | -7.0 | 4.0x10^-16^ |
| ^σ^K^ | *sleC* | CD630_05510 | spore cortex-lytic enzyme pre-pro-form | 356 | -7.0 | 5.9x10^-42^ | 417 | -3.1 | 6.4x10^-16^ | 372 | -6.4 | 2.4x10^-59^ | 750 | -0.2 | 1 | 307 | -6.2 | 3.4x10^-39^ |
| σ^E^ | *dapG* | CD630_13220 | aspartate kinase I | 351 | -2.2 | 7.9x10^-8^ | 378 | -2.0 | 6.3x10^-8^ | 349 | -2.6 | 7.9x10^-17^ | 483 | -1.1 | 8.8x10^-3^ | 357 | -1.2 | 0.06 |
| Spo0A | *spoVD* | CD630_26560 | stage V sporulation protein D (Sporulation-specific penicillin-binding protein) | 312 | -2.7 | 1.1x10^-9^ | 610 | 0.2 | 1 | 551 | -0.1 | 1 | 589 | -0.1 | 1 | 447 | -0.1 | 1 |
| ^Spo0A | *CD3032* | CD630_30320 | pyridoxal phosphate-dependent transferase | 306 | -3.5 | 3.0x10^-17^ | 472 | -0.7 | 0.2 | 454 | -0.8 | 0.1 | 663 | 0.1 | 1 | 347 | -1.2 | 0.03 |
| ^Spo0A | *CD1581* | CD630_15810 | hypothetical protein | 306 | -2.0 | 2.1x10^-5^ | 387 | -1.0 | 0.03 | 398 | -0.8 | 0.1 | 522 | -0.2 | 1 | 287 | -1.5 | 6.3x10^-3^ |
| Spo0A | *sigF* | CD630_07720 | sporulation factor σ^F^ | 304 | -4.9 | 1.7x10^-27^ | 763 | 0.6 | 0.5 | 740 | 0.5 | 0.5 | 731 | 0.3 | 1 | 470 | -0.2 | 1 |
| ^σ^E^ | *CD3522* | CD630_35220 | hypothetical protein | 286 | -6.1 | 2.8x10^-10^ | 317 | -4.0 | 2.1x10^-6^ | 298 | -6.0 | 8.7x10^-11^ | 449 | -1.3 | 0.2 | 312 | -1.8 | 0.2 |
| σ^F^ | *polA* | CD630_11280 | DNA polymerase I | 282 | -2.5 | 3.7x10^-9^ | 317 | -2.0 | 3.2x10^-7^ | 314 | -1.9 | 2.8x10^-9^ | 421 | -0.8 | 0.1 | 309 | -1.0 | 0.2 |
| σ^E^ | *spoIIIAA* | CD630_11920 | stage III sporulation protein AA | 261 | -6.7 | 7.3x10^-38^ | 287 | -4.2 | 2.9x10^-25^ | 271 | -6.9 | 5.9x10^-59^ | 418 | -1.2 | 1.3x10^-3^ | 326 | -1.1 | 0.1 |
| σ^K^ | *dpaA* | CD630_29680 | dipicolinate synthase subunit A | 249 | -5.7 | 1.9x10^-31^ | 286 | -3.1 | 5.7x10^-16^ | 257 | -6.0 | 3.5x10^-52^ | 506 | -0.3 | 1 | 214 | -5.4 | 1.1x10^-31^ |
| ^Spo0A | *drm* | CD630_12230 | phosphopentomutase | 240 | -2.2 | 1.6x10^-5^ | 390 | -0.2 | 1 | 403 | 0.0 | 1 | 428 | -0.1 | 1 | 301 | -0.3 | 0.9 |
| ^σ^E^ | *cspC* | CD630_22460 | subtilisin-like germination-related protease | 239 | -4.1 | 3.4x10^-18^ | 264 | -3.2 | 8.3x10^-17^ | 243 | -5.0 | 8.8x10^-41^ | 401 | -0.8 | 0.1 | 276 | -1.3 | 0.07 |
| ^Spo0A | *CD1880* | CD630_18800 | hypothetical protein (Cupin_2 superfamily) | 215 | -3.5 | 2.5x10^-16^ | 268 | -1.8 | 8.3x10^-6^ | 319 | -0.9 | 0.04 | 300 | -1.5 | 1.8x10^-5^ | 274 | -0.7 | 0.7 |
| Spo0A | *glmS* | CD630_01200 | glucosamine-fructose-6-phosphate aminotransferase | 203 | -2.3 | 1.2x10^-4^ | 236 | -1.6 | 6.9x10^-3^ | 224 | -1.8 | 1.6x10^-3^ | 362 | -0.1 | 1 | 185 | -1.8 | 0.02 |
| Spo0A | *spoIIAA* | CD630_07700 | anti-σ^F^ factor antagonist | 200 | -4.2 | 1.3x10^-20^ | 471 | 0.5 | 0.7 | 439 | 0.3 | 0.8 | 453 | 0.2 | 1 | 305 | -0.2 | 1 |
| ^σ^E^ | *CD3521* | CD630_35210 | peptidase T, M20B family | 193 | -2.4 | 9.8x10^-9^ | 216 | -1.9 | 1.5x10^-6^ | 193 | -2.8 | 6.7x10^-13^ | 292 | -0.7 | 0.2 | 210 | -1.0 | 0.3 |
| Spo0A | *CD1131* | CD630_11310 | solute-binding lipoprotein | 190 | -2.7 | 1.3x10^-10^ | 221 | -1.9 | 2.1x10^-6^ | 228 | -1.6 | 8.8x10^-7^ | 285 | -0.9 | 0.05 | 212 | -1.0 | 0.2 |
| σ^E^ | *brnQ-1* | CD630_12590 | Branched chain amino acid transport system carrier protein | 185 | -5.0 | 9.5x10^-25^ | 235 | -2.0 | 2.4x10^-7^ | 207 | -3.2 | 1.8x10^-19^ | 368 | -0.3 | 1 | 301 | -0.1 | 1 |
| Spo0A | *CD1823* | CD630_18230 | hypothetical protein (DUF328) | 185 | -2.1 | 2.9x10^-6^ | 327 | 0.1 | 1 | 273 | -0.4 | 0.7 | 298 | -0.4 | 0.9 | 201 | -0.8 | 0.4 |
| Spo0A | *CD1824* | CD630_18240 | P-type calcium transport ATPase | 179 | -2.3 | 4.5x10^-8^ | 367 | 0.4 | 0.8 | 315 | 0.1 | 1 | 349 | 0.1 | 1 | 242 | -0.2 | 1 |
| σ^K^ | *spoVFB* | CD630_29670 | dipicolinate synthase subunit B | 177 | -4.8 | 2.7x10^-24^ | 202 | -3.1 | 9.6x10^-15^ | 181 | -5.6 | 1.1x10^-44^ | 347 | -0.3 | 1 | 153 | -4.6 | 3.1x10^-24^ |
| ^σ^E^ | *cspBA* | CD630_22470 | subtilisin-like germination related protease | 173 | -3.1 | 9.5x10^-8^ | 188 | -2.7 | 6.7x10^-11^ | 173 | -3.7 | 4.2x10^-14^ | 270 | -0.9 | 0.1 | 197 | -1.0 | 0.2 |
| σ^E^ | *spoIIIAG* | CD630_11980 | stage III sporulation protein AG | 164 | -5.9 | 6.9x10^-29^ | 189 | -3.2 | 1.3x10^-15^ | 171 | -6.1 | 7.2x10^-48^ | 284 | -0.8 | 0.1 | 210 | -1.0 | 0.3 |
| σ^E^ | *spoIIIAH* | CD630_11990 | stage III sporulation protein AH | 162 | -6.2 | 3.2x10^-28^ | 187 | -3.2 | 2.1x10^-15^ | 170 | -5.4 | 7.9x10^-26^ | 286 | -0.8 | 0.2 | 208 | -0.9 | 0.6 |
| σ^E^ | *spoIIID* | CD630_01260 | stage III sporulation protein D | 160 | -7.2 | 2.0x10^-25^ | 183 | -3.5 | 7.4x10^-13^ | 167 | -6.8 | 2.2x10^-24^ | 289 | -0.7 | 0.4 | 190 | -1.4 | 0.2 |
| σ^E^ | *spoIIIAB* | CD630_11930 | stage III sporulation protein AB | 153 | -6.4 | 6.2x10^-31^ | 168 | -4.1 | 6.2x10^-23^ | 158 | -7.2 | 4.5x10^-52^ | 242 | -1.3 | 1.1x10^-3^ | 199 | -0.9 | 0.3 |
| ^σ^E^ | *CD1613* | CD630_16130 | hypothetical protein (coat protein "CotA," [1]) | 151 | -4.2 | 6.7x10^-3^ | 174 | -2.7 | 0.1 | 161 | -3.6 | 0.02 | 341 | 0.2 | 1 | 135 | -3.4 | 0.1 |
| σ^E^ | *CD1068* | CD630_10680 | polysaccharide biosynthesis/sporulation protein | 150 | -4.1 | 2.1x10^-19^ | 180 | -2.3 | 1.2x10^-8^ | 154 | -4.5 | 2.2x10^-32^ | 253 | -0.8 | 0.2 | 190 | -0.8 | 0.6 |
| ^σ^E^ | *CD2864* | CD630_28640 | hydrolase | 143 | -5.3 | 1.9x10^-25^ | 168 | -2.8 | 1.8x10^-12^ | 148 | -5.6 | 4.3x10^-41^ | 267 | -0.5 | 0.6 | 183 | -0.9 | 0.3 |
| ^Spo0A | *CD1463* | CD630_14630 | hypothetical protein | 138 | -2.3 | 9.6x10^-8^ | 185 | -0.9 | 0.1 | 212 | -0.4 | 0.7 | 189 | -1.2 | 8.8x10^-3^ | 189 | -0.1 | 1 |
| ^σ^E^ | *CD1511* | CD630_15110 | [hypothetical protein (coat protein - "CotB," [1])](#RANGE!_ENREF_1) | 137 | -5.5 | 1.3x10^-25^ | 163 | -2.7 | 1.3x10^-11^ | 146 | -4.5 | 3.5x10^-32^ | 270 | -0.3 | 1 | 200 | -0.4 | 1 |
| σ^E^ | *CD2441A* | CD630_24411 | phoH-like protein | 136 | -3.3 | 4.4x10^-13^ | 148 | -2.8 | 7.2x10^-12^ | 139 | -3.5 | 9.4x10^-23^ | 225 | -0.7 | 0.3 | 161 | -0.9 | 0.4 |
| σ^E^ | *CD1403* | CD630_14030 | synthetase | 135 | -3.1 | 3.8x10^-12^ | 159 | -2.0 | 5.5x10^-7^ | 145 | -2.8 | 9.9x10^-15^ | 230 | -0.6 | 0.5 | 177 | -0.5 | 0.9 |
| ^Spo0A | *sigG* | CD630_26420 | sporulation factor σ^G^ | 127 | -5.4 | 1.5x10^-24^ | 356 | 0.8 | 0.2 | 205 | -0.8 | 0.1 | 294 | 0.1 | 1 | 187 | -0.4 | 1 |
| σ^E^ | *CD3462* | CD630_34620 | antitoxin endoAI | 126 | -3.4 | 1.7x10^-13^ | 136 | -3.0 | 4.2x10^-13^ | 128 | -4.0 | 1.1x10^-18^ | 216 | -0.6 | 0.5 | 159 | -0.7 | 0.7 |
| Spo0A | *CD1404* | CD630_14040 | oligopeptide transporter | 126 | -3.4 | 1.1x10^-4^ | 201 | -0.6 | 0.7 | 190 | -0.8 | 0.5 | 236 | -0.3 | 1 | 160 | -0.7 | 0.9 |
| σ^E^ | *CD3181* | CD630_31810 | chlorohydrolase/aminohydrolase | 122 | -2.8 | 2.7x10^-9^ | 130 | -2.7 | 1.5x10^-8^ | 128 | -2.7 | 3.0x10^-8^ | 193 | -0.7 | 0.4 | 156 | -0.5 | 1 |
| Spo0A | *CD3290* | CD630_32900 | hypothetical protein | 120 | -2.4 | 4.4x10^-8^ | 280 | 0.7 | 0.3 | 278 | 0.7 | 0.2 | 284 | 0.6 | 0.6 | 171 | 0.0 | 1 |
| σ^F^ | *CD2375* | CD630_23750 | hypothetical protein (DUF1540) | 119 | -5.2 | 5.8x10^-24^ | 143 | -2.5 | 5.1x10^-10^ | 240 | 0.0 | 1 | 174 | -1.6 | 1.4x10^-5^ | 171 | -0.5 | 1 |
| σ^E^ | *CD1168* | CD630_11680 | membrane protein (spore coat YlbJ, [[4](#_ENREF_4)]) | 117 | -6.0 | 9.7x10^-27^ | 134 | -3.2 | 7.2x10^-15^ | 120 | -6.8 | 8.0x10^-46^ | 201 | -0.9 | 0.1 | 143 | -1.1 | 0.3 |
| σ^E^ | *CD1380* | CD630_13800 | transporter, Major Facilitator Superfamily (MFS) | 114 | -3.5 | 9.7x10^-13^ | 134 | -2.2 | 2.0x10^-8^ | 130 | -2.4 | 1.4x10^-11^ | 184 | -0.9 | 0.1 | 114 | -1.9 | 6.1x10^-5^ |
| Spo0A | *CD0145* | CD630_01450 | S1 RNA-binding domain-containing protein | 114 | -2.2 | 1.5x10^-5^ | 190 | -0.1 | 1 | 221 | 0.4 | 0.9 | 178 | -0.5 | 0.7 | 117 | -1.1 | 0.3 |
| Spo0A | *CD0622* | CD630_06220 | hypothetical protein (DUF1629) | 113 | -7.5 | 2.1x10^-10^ | 192 | -0.7 | 0.7 | 167 | -1.2 | 0.2 | 210 | -0.6 | 0.9 | 171 | -0.4 | 1 |
| Spo0A | *CD1222* | CD630_12220 | integrase site-specific recombinase XerD-like | 108 | -3.1 | 1.3x10^-12^ | 192 | -0.2 | 1 | 196 | -0.1 | 1 | 212 | -0.1 | 1 | 154 | -0.2 | 1 |
| σ^E^ | *CD0129* | CD630_01290 | hypothetical protein (YyaC, in sporulating bacteria) | 105 | -5.1 | 8.9x10^-23^ | 126 | -2.5 | 6.7x10^-10^ | 109 | -5.1 | 3.0x10^-33^ | 198 | -0.5 | 0.8 | 148 | -0.5 | 0.9 |
| ^σ^E^ | *CD1319* | CD630_13190 | polysaccharide deacetylase | 103 | -5.0 | 1.4x10^-21^ | 113 | -3.7 | 7.9x10^-18^ | 107 | -5.2 | 3.5x10^-34^ | 155 | -1.4 | 2.9x10^-4^ | 126 | -1.1 | 0.2 |
| σ^E^ | *EndoA* | CD630_34610 | endoribonuclease toxin | 103 | -3.5 | 3.9x10^-13^ | 112 | -3.0 | 9.6x10^-12^ | 104 | -4.1 | 4.8x10^-17^ | 181 | -0.5 | 0.7 | 139 | -0.5 | 0.9 |
| σ^E^ | *CD1928* | CD630_19280 | membrane protein | 100 | -7.4 | 2.0x10^-28^ | 114 | -3.4 | 7.2x10^-16^ | 105 | -6.0 | 5.7x10^-38^ | 152 | -1.5 | 1.4x10^-4^ | 119 | -1.3 | 0.1 |
| σ^E^ | *CD2833* | CD630_28330 | calcium-transporting ATPase | 100 | -4.8 | 1.7x10^-18^ | 119 | -2.7 | 1.1x10^-10^ | 102 | -6.1 | 9.7x10^-40^ | 184 | -0.6 | 0.6 | 124 | -1.0 | 0.4 |
| ^σ^G^(σ^E^) | *sspB* | CD630_32490 | Small, acid-soluble spore protein beta | 97 | -8.1 | 1.8x10^-11^ | 102 | -7.0 | 1.2x10^-11^ | 115 | -2.8 | 6.4x10^-4^ | 109 | -7.0 | 7.3x10^-11^ | 129 | -0.8 | 1 |
| ^σ^K^ | *CD1133* | CD630_11330 | hypothetical protein | 96 | -4.9 | 8.8x10^-21^ | 119 | -2.2 | 7.1x10^-7^ | 101 | -4.5 | 1.7x10^-18^ | 255 | 0.5 | 0.6 | 86 | -3.6 | 9.0x10^-13^ |
| Spo0A | *prfB* | CD630_01440 | peptide chain release factor 2 (RF-2) | 96 | -2.2 | 1.1x10^-6^ | 197 | 0.5 | 0.7 | 216 | 0.7 | 0.2 | 181 | 0.1 | 1 | 116 | -0.5 | 0.9 |
| σ^E^ | *CD0017* | CD630_00170 | DNA binding protein | 95 | -2.1 | 1.0x10^-4^ | 105 | -1.8 | 1.2x10^-3^ | 97 | -2.2 | 4.7x10^-5^ | 141 | -0.7 | 0.5 | 101 | -0.9 | 0.5 |
| Spo0A | *coaE* | CD630_11290 | dephospho-CoA kinase | 94 | -2.7 | 1.7x10^-8^ | 113 | -1.7 | 9.0x10^-5^ | 113 | -1.6 | 3.2x10^-5^ | 155 | -0.5 | 0.6 | 107 | -0.9 | 0.4 |
| σ^E^ | *CD3298* | CD630_32980 | ATP/GTP-binding protein | 92 | -6.6 | 2.6x10^-16^ | 112 | -2.6 | 1.0x10^-5^ | 97 | -5.6 | 4.7x10^-15^ | 168 | -0.7 | 0.6 | 107 | -1.4 | 0.3 |
| σ^E^ | *CD1066* | CD630_10660 | hypothetical protein | 91 | -4.0 | 1.8x10^-15^ | 105 | -2.7 | 2.4x10^-10^ | 95 | -4.1 | 4.6x10^-24^ | 149 | -0.9 | 0.1 | 96 | -1.7 | 2.3x10^-3^ |
| σ^E^ | *CD3177* | CD630_31770 | xanthine dehydrogenase | 90 | -4.5 | 1.2x10^-11^ | 103 | -3.0 | 4.8x10^-8^ | 98 | -3.5 | 3.5x10^-9^ | 153 | -0.8 | 0.3 | 125 | -0.6 | 1 |
| σ^E^ | *dnaX* | CD630_00160 | DNA polymerase III subunits gamma and tau | 85 | -3.4 | 4.2x10^-7^ | 97 | -2.4 | 1.5x10^-4^ | 90 | -3.2 | 9.3x10^-7^ | 141 | -0.7 | 0.6 | 102 | -0.9 | 0.6 |
| σ^E^ | *CD2800* | CD630_28000 | membrane protein | 84 | -4.5 | 2.4x10^-18^ | 95 | -3.2 | 1.7x10^-13^ | 86 | -5.4 | 2.5x10^-32^ | 132 | -1.1 | 0.01 | 96 | -1.4 | 0.03 |
| Spo0A | *mgsA* | CD630_11530 | methylglyoxal synthase (MGS) | 84 | -2.0 | 1.3x10^-5^ | 165 | 0.4 | 0.8 | 158 | 0.3 | 0.8 | 151 | 0.0 | 1 | 105 | -0.3 | 1 |
| Spo0A | *pdp* | CD630_12250 | pyrimidine-nucleoside phosphorylase | 83 | -2.7 | 3.6x10^-4^ | 155 | 0.1 | 1 | 150 | 0.0 | 1 | 176 | 0.2 | 1 | 107 | -0.5 | 1 |
| Spo0A | *mrdB* | CD630_11520 | Rod shape-determining protein MrdB | 83 | -2.0 | 2.5x10^-5^ | 162 | 0.4 | 0.8 | 164 | 0.4 | 0.6 | 155 | 0.1 | 1 | 103 | -0.3 | 1 |
| ^σ^E^ | *CD0761* | CD630_07610 | ATP-dependent RNA helicase | 81 | -2.8 | 9.7x10^-10^ | 89 | -2.4 | 1.3x10^-8^ | 83 | -2.9 | 1.3x10^-14^ | 111 | -1.4 | 1.1x10^-3^ | 90 | -1.0 | 0.4 |
| Spo0A | *bcp* | CD630_18220 | thiol peroxidase | 80 | -2.0 | 3.4x10^-4^ | 151 | 0.3 | 1 | 123 | -0.2 | 1 | 143 | 0.0 | 1 | 92 | -0.6 | 0.8 |
| σ^F^ | *CD2376* | CD630_23760 | [membrane protein YtvI involved in sporulation [4]](#RANGE!_ENREF_4) | 74 | -3.7 | 3.1x10^-13^ | 81 | -3.1 | 7.1x10^-13^ | 105 | -1.1 | 8.1x10^-3^ | 111 | -1.3 | 4.7x10^-3^ | 97 | -0.6 | 0.7 |
| σ^E^ | *CD1940* | CD630_19400 | membrane protein (DUF3866 superfamily) | 73 | -6.3 | 3.4x10^-23^ | 84 | -3.1 | 8.8x10^-13^ | 78 | -4.4 | 1.6x10^-24^ | 128 | -0.8 | 0.3 | 106 | -0.5 | 0.9 |
| ^σ^G^ | *CD2687A* | CD630_26871 | hypothetical protein | 73 | -4.9 | 3.0x10^-18^ | 80 | -3.6 | 1.0x10^-15^ | 118 | -0.7 | 0.1 | 98 | -2.1 | 7.0x10^-8^ | 92 | -0.9 | 0.4 |
| σ^E^ | *CD1321* | CD630_13210 | sporulation protein (YlmC) | 72 | -2.1 | 6.7x10^-6^ | 72 | -2.6 | 4.4x10^-9^ | 68 | -3.1 | 5.5x10^-13^ | 95 | -1.2 | 0.01 | 70 | -1.4 | 0.06 |
| ^σ^K^ | *CD1063A* | CD630_10631 | hypothetical protein | 71 | -7.7 | 6.8x10^-9^ | 86 | -2.7 | 3.3x10^-3^ | 74 | -8.3 | 3.1x10^-10^ | 167 | 0.1 | 1 | 63 | -4.8 | 2.5x10^-5^ |
| ^σ^G^ | *CD1486* | CD630_14860 | ribosome recycling factor | 71 | -5.8 | 4.2x10^-21^ | 75 | -5.0 | 6.4x10^-23^ | 125 | -0.4 | 0.7 | 80 | -5.5 | 1.3x10^-27^ | 86 | -1.1 | 0.3 |
| σ^K^ | *feoA* | CD630_15180 | ferrous iron transport protein | 70 | -4.7 | 2.0x10^-12^ | 79 | -3.2 | 9.2x10^-8^ | 72 | -5.2 | 3.9x10^-14^ | 149 | 0.0 | 1 | 60 | -4.7 | 3.0x10^-11^ |
| σ^E^ | *CD1398* | CD630_13980 | peptidase, M20D family | 69 | -4.9 | 2.4x10^-18^ | 82 | -2.6 | 1.7x10^-9^ | 74 | -4.1 | 2.7x10^-22^ | 119 | -0.8 | 0.2 | 83 | -1.2 | 0.2 |
| σ^K^ | *CD0749* | CD630_07490 | DNA helicase, UvrD/REP type | 69 | -4.1 | 4.7x10^-14^ | 91 | -1.6 | 5.0x10^-4^ | 77 | -2.8 | 1.5x10^-13^ | 226 | 1.1 | 0.01 | 60 | -3.5 | 2.8x10^-12^ |
| σ^E^ | *CD3465* | CD630_34650 | hypothetical protein (CBS domain) | 69 | -2.8 | 1.7x10^-8^ | 72 | -2.9 | 4.0x10^-10^ | 71 | -3.0 | 1.9x10^-11^ | 109 | -0.7 | 0.3 | 89 | -0.5 | 0.9 |
| σ^K^ | *sigK* | CD630_12300 | sporulation factor σ^K^ | 68 | -4.6 | 4.8x10^-17^ | 80 | -2.5 | 8.1x10^-9^ | 68 | -7.2 | 4.8x10^-35^ | 138 | -0.2 | 1 | 61 | -3.6 | 1.7x10^-12^ |
| ^σ^K^ | *CD0596* | CD630_05960 | hypothetical protein (CotJA homolog) | 67 | -7.0 | 1.2x10^-5^ | 75 | -3.7 | 4.7x10^-3^ | 69 | -7.5 | 7.3x10^-7^ | 159 | 0.2 | 1 | 58 | -6.1 | 1.4x10^-4^ |
| ^σ^E^ | *CD3258* | CD630_32580 | Iron hydrogenase | 66 | -4.9 | 3.3x10^-16^ | 71 | -4.1 | 1.3x10^-17^ | 69 | -5.0 | 8.4x10^-28^ | 103 | -1.2 | 8.8x10^-3^ | 71 | -1.8 | 1.5x10^-3^ |
| σ^E^ | *CD1396* | CD630_13960 | amino acid amidase | 66 | -2.0 | 5.1x10^-5^ | 75 | -1.5 | 1.2x10^-3^ | 69 | -2.0 | 7.0x10^-7^ | 104 | -0.4 | 0.9 | 75 | -0.6 | 0.8 |
| Spo0A | *ptsG-BC* | CD630_26670 | PTS system glucose-specific transporter subunit IIBC | 65 | -3.0 | 0.02 | 119 | -0.1 | 1 | 84 | -1.3 | 0.4 | 111 | -0.5 | 1 | 68 | -1.4 | 0.7 |
| Spo0A | *pupG* | CD630_12240 | Purine nucleoside phosphorylase | 65 | -2.2 | 4.8x10^-6^ | 107 | -0.1 | 1 | 110 | 0.0 | 1 | 118 | -0.1 | 1 | 86 | -0.2 | 1 |
| Spo0A | *sigE* | CD630_26430 | sporulation factor σ^E^ | 64 | -4.6 | 7.0x10^-16^ | 205 | 1.1 | 0.01 | 160 | 0.6 | 0.4 | 144 | 0.1 | 1 | 101 | -0.1 | 1 |
| Spo0A | *CD1878A* | CD630_18781 | pseudo | 64 | -2.1 | 0.03 | 91 | -0.6 | 0.8 | 101 | -0.3 | 1 | 108 | -0.3 | 1 | 70 | -0.9 | 0.7 |
| Spo0A | *CD2435* | CD630_24351 | hypothetical protein (Yqz-like) | 64 | -2.0 | 7.2x10^-5^ | 73 | -1.4 | 2.8x10^-3^ | 75 | -1.2 | 4.4x10^-3^ | 87 | -0.9 | 0.1 | 70 | -0.7 | 0.7 |
| Spo0A | *CD1221* | CD630_12210 | membrane protein (SpoIIM homolog) | 63 | -5.6 | 4.4x10^-21^ | 106 | -0.7 | 0.5 | 112 | -0.4 | 0.8 | 120 | -0.5 | 0.8 | 83 | -0.8 | 0.6 |
| ^σ^K^ | *cotJC1* | CD630_05980 | [spore coat assembly protein ("CotCB," [1])](#RANGE!_ENREF_1) | 63 | -4.8 | 0.03 | 69 | -3.7 | 0.1 | 63 | -6.7 | 2.7x10^-3^ | 151 | 0.3 | 1 | 53 | -6.2 | 0.02 |
| Spo0A | *CD2720* | CD630_27200 | transporter | 61 | -2.0 | 8.2x10^-5^ | 71 | -1.4 | 7.7x10^-3^ | 68 | -1.6 | 2.2x10^-4^ | 116 | 0.2 | 1 | 61 | -1.1 | 0.2 |
| ^σ^K^ | *CD3580* | CD630_35800 | hypothetical protein | 59 | -5.6 | 1.5x10^-19^ | 68 | -3.0 | 9.2x10^-11^ | 62 | -5.1 | 6.6x10^-24^ | 111 | -0.5 | 0.8 | 51 | -4.7 | 2.7x10^-16^ |
| Spo0A | *CD1233* | CD630_12330 | cell surface protein | 59 | -3.0 | 1.9x10^-8^ | 109 | 0.0 | 1 | 111 | 0.1 | 1 | 123 | 0.1 | 1 | 79 | -0.4 | 1 |
| ^σ^G^ | *CD0684* | CD630_06840 | ATP-dependent peptidase, M41 family | 58 | -3.7 | 6.9x10^-12^ | 60 | -4.3 | 7.9x10^-18^ | 115 | 0.1 | 1 | 65 | -3.9 | 6.9x10^-20^ | 67 | -1.2 | 0.2 |
| σ^E^ | *CD0760* | CD630_07600 | Ca^2+^/Na^+^ antiporter | 58 | -3.6 | 9.9x10^-10^ | 68 | -2.2 | 8.6x10^-5^ | 59 | -3.9 | 4.7x10^-10^ | 90 | -1.0 | 0.1 | 64 | -1.3 | 0.1 |
| ^σ^K^ | *bclA1* | CD630_03320 | exosporium glycoprotein | 57 | -5.4 | 3.3x10^-6^ | 66 | -3.2 | 4.8x10^-4^ | 59 | -7.1 | 7.2x10^-10^ | 110 | -0.4 | 1 | 49 | -5.6 | 6.6x10^-7^ |
| σ^E^ | *CD1320* | CD630_13200 | M16 family peptidase | 56 | -2.3 | 7.4x10^-4^ | 58 | -2.6 | 2.3x10^-4^ | 55 | -2.9 | 2.9x10^-5^ | 75 | -1.2 | 0.2 | 56 | -1.3 | 0.2 |
| σ^E^ | *CD2637* | CD630_26370 | two-component sensor histidine kinase | 56 | -2.2 | 5.2x10^-5^ | 59 | -2.2 | 3.2x10^-6^ | 55 | -2.6 | 1.5x10^-7^ | 75 | -1.1 | 0.04 | 56 | -1.2 | 0.4 |
| σ^E^ | *spoIIIAF* | CD630_11970 | stage III sporulation protein AF | 55 | -5.0 | 5.8x10^-17^ | 61 | -3.6 | 2.4x10^-14^ | 56 | -6.2 | 5.6x10^-30^ | 94 | -0.8 | 0.2 | 69 | -1.0 | 0.3 |
| Spo0A | *CD1229* | CD630_12290 | peptidoglycan glycosyltransferase | 55 | -3.0 | 5.5x10^-6^ | 72 | -1.3 | 0.1 | 81 | -0.7 | 0.4 | 82 | -1.0 | 0.3 | 61 | -1.1 | 0.4 |
| σ^E^ | *CD2121* | CD630_21210 | hypothetical protein | 54 | -5.4 | 2.9x10^-8^ | 64 | -2.7 | 5.1x10^-4^ | 56 | -5.5 | 2.1x10^-8^ | 96 | -0.7 | 0.8 | 62 | -1.4 | 0.4 |
| σ^E^ | *CD3178* | CD630_31780 | D-hydantoinase | 54 | -3.8 | 3.2x10^-11^ | 60 | -2.8 | 2.4x10^-9^ | 57 | -3.4 | 3.6x10^-11^ | 83 | -1.1 | 0.05 | 65 | -0.9 | 0.5 |
| Spo0A | *CD1967* | CD630_19670 | hypothetical protein | 53 | -3.7 | 4.6x10^-11^ | 159 | 1.0 | 0.04 | 125 | 0.5 | 0.6 | 137 | 0.5 | 0.7 | 90 | 0.2 | 1 |
| σ^K^ | *CD0896* | CD630_08960 | hypothetical protein | 52 | -6.8 | 5.7x10^-6^ | 59 | -3.6 | 4.5x10^-3^ | 57 | -4.4 | 6.4x10^-4^ | 100 | -0.5 | 1 | 46 | -5.6 | 4.8x10^-4^ |
| σ^F^ | *CD1130* | CD630_11300 | lytic transglycosylase-like protein | 51 | -2.7 | 1.2x10^-6^ | 58 | -2.0 | 3.3x10^-5^ | 58 | -1.8 | 8.7x10^-6^ | 76 | -0.9 | 0.2 | 53 | -1.3 | 0.1 |
| σ^E^ | *CD2799A* | CD630_27991 | hypothetical protein | 50 | -3.3 | 3.5x10^-10^ | 58 | -2.4 | 2.2x10^-7^ | 50 | -4.1 | 3.9x10^-18^ | 79 | -0.9 | 0.1 | 59 | -1.0 | 0.4 |
| ^σ^G^ | *rbr* | CD630_28450 | rubrerythrin | 48 | -4.4 | 1.6x10^-13^ | 51 | -3.8 | 2.1x10^-14^ | 96 | 0.0 | 1 | 56 | -3.5 | 8.4x10^-16^ | 66 | -0.5 | 1 |
| Spo0A | *CD1219* | CD630_12190 | hypothetical protein | 48 | -2.5 | 4.7x10^-6^ | 83 | 0.0 | 1 | 83 | 0.0 | 1 | 85 | -0.2 | 1 | 59 | -0.5 | 0.9 |
| Spo0A | *CD3287* | CD630_32870 | oxidoreductase, FAD dependent | 48 | -2.4 | 8.5x10^-6^ | 76 | -0.4 | 0.8 | 91 | 0.2 | 1 | 78 | -0.5 | 0.8 | 52 | -1.0 | 0.3 |
| Spo0A | *aroC* | CD630_18350 | chorismate synthase | 48 | -2.0 | 4.4x10^-3^ | 83 | 0.1 | 1 | 95 | 0.5 | 0.8 | 93 | 0.2 | 1 | 54 | -0.6 | 0.9 |
| σ^E^ | *spoIIIAE* | CD630_11960 | stage III sporulation protein AE | 47 | -4.9 | 1.8x10^-14^ | 52 | -3.6 | 4.4x10^-13^ | 49 | -5.1 | 7.8x10^-22^ | 76 | -1.0 | 0.1 | 63 | -0.7 | 0.8 |
| σ^K^ | *CD2144* | CD630_21440 | putative sporulation membrane protein YtaF | 46 | -6.7 | 8.8x10^-11^ | 58 | -2.3 | 1.8x10^-3^ | 49 | -4.8 | 1.4x10^-9^ | 95 | -0.2 | 1 | 40 | -6.0 | 8.1x10^-10^ |
| Spo0A | *spoIIGA* | CD630_26440 | sporulation σ^x10--^processing peptidase | 46 | -4.3 | 6.3x10^-13^ | 126 | 0.8 | 0.2 | 119 | 0.7 | 0.3 | 99 | 0.0 | 1 | 64 | -0.5 | 0.9 |
| σ^F^ | *CD0125* | CD630_01250 | cell wall endopeptidase (SpoIIQ homolog, [[5](#_ENREF_5)]) | 46 | -4.2 | 1.3x10^-12^ | 49 | -3.6 | 3.4x10^-13^ | 101 | 0.3 | 1 | 72 | -1.1 | 0.05 | 58 | -0.8 | 0.6 |
| ^σ^G^ | *spoVAD* | CD630_07740 | stage V sporulation protein AD | 46 | -4.2 | 5.7x10^-13^ | 48 | -5.1 | 1.9x10^-19^ | 66 | -1.2 | 7.7x10^-3^ | 52 | -4.7 | 3.3x10^-22^ | 58 | -0.9 | 0.6 |
| Spo0A | *ptsG-A* | CD630_26660 | PTS system glucose-specific transporter subunit IIA | 46 | -2.9 | 0.02 | 84 | -0.1 | 1 | 59 | -1.3 | 0.3 | 79 | -0.5 | 1 | 46 | -1.7 | 0.5 |
| σ^E^ | *CD1167* | CD630_11670 | integrase/recombinase | 45 | -6.2 | 2.1x10^-17^ | 52 | -3.1 | 6.3x10^-11^ | 48 | -4.8 | 5.7x10^-23^ | 82 | -0.6 | 0.5 | 61 | -0.7 | 0.9 |
| ^σ^E^ | *CD3652* | CD630_36520 | peptidase, M1 family | 45 | -4.9 | 5.6x10^-14^ | 52 | -3.0 | 5.1x10^-10^ | 46 | -8.5 | 1.1x10^-23^ | 80 | -0.7 | 0.5 | 60 | -0.7 | 0.8 |
| σ^G^ | *CD3312* | CD630_33120 | transporter, Major Facilitator Superfamily (MFS) | 45 | -4.3 | 1.9x10^-9^ | 48 | -4.0 | 6.0x10^-10^ | 62 | -1.4 | 5.5x10^-3^ | 50 | -5.2 | 4.0x10^-12^ | 61 | -0.6 | 1 |
| σ^E^ | *CD1395* | CD630_13950 | membrane protein (YrvL superfamily) | 45 | -3.2 | 4.2x10^-9^ | 54 | -1.9 | 5.6x10^-5^ | 49 | -2.5 | 2.4x10^-8^ | 79 | -0.4 | 0.9 | 59 | -0.5 | 0.9 |
| Spo0A | *CD1235* | CD630_12350 | hypothetical protein | 45 | -2.8 | 4.3x10^-3^ | 87 | 0.2 | 1 | 80 | 0.0 | 1 | 98 | 0.3 | 1 | 60 | -0.3 | 1 |
| Spo0A | *pflE* | CD630_32830 | pyruvate formate-lyase (activating enzyme) | 45 | -2.0 | 0.01 | 82 | 0.2 | 1 | 73 | -0.1 | 1 | 75 | -0.2 | 1 | 50 | -0.7 | 0.9 |
| σ^E^ | *CD0557* | CD630_05570 | uridine kinase | 44 | -3.7 | 5.1x10^-10^ | 61 | -1.2 | 0.03 | 52 | -2.2 | 7.9x10^-8^ | 80 | -0.5 | 0.9 | 54 | -0.9 | 0.5 |
| σ^G^ | *CD1430* | CD630_14300 | delta-lactam-biosynthetic de-N-acteylase | 43 | -4.7 | 3.4x10^-13^ | 45 | -4.7 | 1.2x10^-17^ | 84 | -0.1 | 1 | 49 | -4.3 | 2.2x10^-19^ | 60 | -0.5 | 0.9 |
| σ^E^ | *CD0296* | CD630_02960 | hypothetical protein | 43 | -2.5 | 1.3x10^-5^ | 51 | -1.6 | 2.0x10^-3^ | 44 | -2.8 | 2.5x10^-5^ | 81 | -0.1 | 1 | 44 | -1.3 | 0.2 |
| σ^E^ | *CD3551B* | CD630_35512 | hypothetical protein | 42 | -6.6 | 8.5x10^-13^ | 48 | -3.3 | 7.5x10^-8^ | 48 | -3.2 | 1.0x10^-6^ | 67 | -1.2 | 0.1 | 54 | -0.9 | 0.7 |
| Spo0A | *CD3569* | CD630_35690 | sporulation-specific protease (YabG) | 42 | -2.7 | 3.4x10^-7^ | 59 | -0.9 | 0.2 | 50 | -1.7 | 2.1x10^-4^ | 98 | 0.5 | 0.8 | 44 | -1.3 | 0.1 |
| Spo0A | *CD2374* | CD630_23740 | hypothetical protein | 41 | -4.6 | 4.4x10^-13^ | 58 | -1.3 | 0.02 | 53 | -1.7 | 1.2x10^-4^ | 79 | -0.3 | 1 | 58 | -0.5 | 1 |
| σ^E^ | *CD1740* | CD630_17400 | glycine/sarcosine/betaine reductase complex component B subunits alpha and beta | 41 | -3.4 | 8.8x10^-9^ | 46 | -2.7 | 3.7x10^-8^ | 43 | -3.5 | 5.9x10^-14^ | 69 | -0.7 | 0.5 | 44 | -1.5 | 0.05 |
| ^σ^E^ | *CD3007* | CD630_30070 | hypothetical protein | 41 | -3.3 | 2.5x10^-8^ | 52 | -1.6 | 1.8x10^-3^ | 48 | -2.1 | 2.1x10^-6^ | 81 | -0.1 | 1 | 41 | -1.8 | 5.3x10^-3^ |
| σ^K^ | *CD3350* | CD630_33500 | family 2 glycosyl transferase | 40 | -5.8 | 2.9x10^-6^ | 45 | -3.5 | 4.0x10^-4^ | 42 | -6.7 | 4.4x10^-8^ | 65 | -1.1 | 0.6 | 34 | –Inf | 1.9x10^-8^ |
| ^σ^G^ | *CD2635* | CD630_26350 | hypothetical protein (YIEGIA family) | 40 | -3.8 | 9.1x10^-11^ | 43 | -3.7 | 5.6x10^-13^ | 58 | -1.0 | 0.05 | 45 | -4.0 | 5.7x10^-17^ | 49 | -0.9 | 0.5 |
| σ^E^ | *CD3457* | CD630_34570 | hypothetical protein | 40 | -3.6 | 1.0x10^-9^ | 46 | -2.3 | 2.4x10^-6^ | 43 | -3.1 | 6.8x10^-11^ | 66 | -0.7 | 0.5 | 46 | -1.1 | 0.3 |
| σ^E^ | *CD3440* | CD630_34400 | glycoside hydrolase-type carbohydrate-binding protein | 40 | -3.5 | 1.2x10^-9^ | 45 | -2.7 | 3.8x10^-8^ | 41 | -3.9 | 2.5x10^-15^ | 68 | -0.7 | 0.5 | 50 | -0.8 | 0.8 |
| σ^E^ | *tepA* | CD630_13230 | protein export-enhancing factor | 40 | -3.2 | 1.5x10^-3^ | 45 | -2.4 | 9.3x10^-3^ | 44 | -2.5 | 8.6x10^-3^ | 57 | -1.3 | 0.4 | 43 | -1.3 | 0.6 |
| Spo0A | *CD1233B* | CD630_12332 | hypothetical protein | 38 | -6.4 | 8.9x10^-18^ | 66 | -0.6 | 0.7 | 69 | -0.4 | 0.8 | 71 | -0.6 | 0.7 | 51 | -0.8 | 0.7 |
| ^σ^G^ | *CD1707* | CD630_17070 | C4-dicarboxylate anaerobic carrier, DcuC family | 38 | -4.3 | 1.5x10^-11^ | 38 | -6.2 | 4.5x10^-20^ | 50 | -1.5 | 3.0x10^-4^ | 41 | -5.8 | 1.0x10^-23^ | 42 | -1.4 | 0.1 |
| Spo0A | *CD3289* | CD630_32890 | hypothetical protein | 38 | -2.8 | 5.8x10^-7^ | 85 | 0.5 | 0.7 | 97 | 0.9 | 0.1 | 99 | 0.7 | 0.4 | 52 | -0.3 | 1 |
| Spo0A | *pheA* | CD630_18360 | bifunctional P-protein, chorismate mutase/prephenate dehydratase | 38 | -2.2 | 0.03 | 71 | 0.2 | 1 | 78 | 0.5 | 0.8 | 74 | 0.2 | 1 | 42 | -0.7 | 0.9 |
| σ^E^ | *CD1397* | CD630_13970 | hypothetical protein (VPF066 superfamily) | 37 | -3.8 | 5.1x10^-10^ | 44 | -2.3 | 4.0x10^-6^ | 40 | -2.9 | 1.8x10^-10^ | 61 | -0.8 | 0.4 | 47 | -0.7 | 0.7 |
| σ^E^ | *CD3248* | CD630_32480 | polysaccharide deacetylase | 37 | -3.0 | 8.3x10^-8^ | 39 | -3.0 | 6.3x10^-9^ | 35 | -4.6 | 2.2x10^-16^ | 56 | -0.9 | 0.2 | 40 | -1.2 | 0.3 |
| σ^E^ | *CD2639* | CD630_26390 | cytotoxic factor | 37 | -2.3 | 4.9x10^-4^ | 41 | -1.9 | 1.8x10^-4^ | 38 | -2.5 | 2.3x10^-7^ | 51 | -1.1 | 0.1 | 37 | -1.3 | 0.2 |
| σ^E^ | *spoIV* | CD630_24420 | stage IV sporulation protein | 36 | -5.8 | 5.1x10^-15^ | 41 | -3.1 | 8.5x10^-10^ | 38 | -4.6 | 3.3x10^-18^ | 67 | -0.6 | 0.7 | 47 | -0.9 | 0.6 |
| ^σ^F^ | *gpr* | CD630_24700 | germination protease | 36 | -5.7 | 1.6x10^-3^ | 39 | -4.4 | 3.5x10^-3^ | 72 | 0.0 | 1 | 51 | -1.8 | 0.5 | 43 | -1.1 | 0.8 |
| σ^K^ | *CD1904* | CD630_19040 | ABC transporter permease | 36 | -5.0 | 1.6x10^-13^ | 41 | -3.4 | 7.5x10^-10^ | 38 | -5.8 | 5.1x10^-16^ | 72 | -0.3 | 1 | 32 | -3.9 | 2.9x10^-10^ |
| σ^E^ | *ssb* | CD630_32350 | single-stranded DNA-binding protein | 36 | -4.8 | 3.2x10^-12^ | 41 | -3.0 | 4.7x10^-9^ | 37 | -5.4 | 7.3x10^-21^ | 67 | -0.5 | 0.8 | 52 | -0.4 | 1 |
| ^σ^G^(σ^E^) | *CD2868* | CD630_28680 | oxidoreductase | 36 | -4.8 | 1.2x10^-12^ | 38 | -5.0 | 3.0x10^-17^ | 42 | -2.6 | 2.2x10^-8^ | 42 | -4.0 | 2.6x10^-16^ | 43 | -1.2 | 0.2 |
| ^σ^G^ | *dacF* | CD630_12910 | D-alanyl-D-alanine carboxypeptidase | 35 | -4.5 | 3.4x10^-11^ | 37 | -4.6 | 7.1x10^-13^ | 76 | 0.2 | 1 | 38 | -5.5 | 1.1x10^-14^ | 45 | -0.8 | 0.8 |
| σ^K^ | *CD0196* | CD630_01960 | pseudo | 35 | -2.7 | 1.5x10^-4^ | 53 | -0.6 | 0.8 | 43 | -1.4 | 0.03 | 128 | 1.5 | 0.1 | 32 | -2.0 | 0.03 |
| Spo0A | *CD1941* | CD630_19410 | hypothetical protein | 34 | -4.2 | 2.9x10^-7^ | 87 | 0.6 | 0.5 | 65 | -0.1 | 1 | 82 | 0.3 | 1 | 51 | -0.3 | 1 |
| Spo0A | *vanZ* | CD630_12400 | Teicoplanin resistance protein | 34 | -3.4 | 1.8x10^-8^ | 46 | -1.3 | 0.05 | 53 | -0.6 | 0.5 | 52 | -1.0 | 0.2 | 40 | -1.0 | 0.6 |
| Spo0A | *CD1232* | CD630_12320 | lipoprotein | 34 | -2.8 | 6.9x10^-6^ | 58 | -0.2 | 1 | 58 | -0.2 | 1 | 60 | -0.3 | 1 | 41 | -0.7 | 0.7 |
| Spo0A | *CD1234* | CD630_12340 | hypothetical protein | 34 | -2.4 | 4.3x10^-5^ | 57 | -0.2 | 1 | 57 | -0.1 | 1 | 65 | 0.0 | 1 | 48 | -0.1 | 1 |
| ^σ^G^ | *spoVT* | CD630_34990 | stage V sporulation protein T | 33 | -5.5 | 2.5x10^-13^ | 36 | -3.6 | 3.1x10^-11^ | 51 | -0.9 | 0.1 | 38 | -4.5 | 4.4x10^-17^ | 41 | -1.0 | 0.4 |
| ^σ^E^ | *cotJB1* | CD630_05970 | spore coat peptide assembly protein | 33 | -4.9 | 0.02 | 37 | -3.5 | 0.1 | 34 | -6.8 | 2.8x10^-3^ | 78 | 0.2 | 1 | 29 | -4.8 | 0.1 |
| σ^E^ | *spoIID* | CD630_01240 | stage II sporulation protein D | 33 | -2.7 | 6.4x10^-6^ | 39 | -1.8 | 6.7x10^-4^ | 35 | -2.6 | 7.0x10^-8^ | 54 | -0.6 | 0.7 | 38 | -1.0 | 0.5 |
| Spo0A | *CD0146* | CD630_01460 | hydrolase | 33 | -2.1 | 5.8x10^-4^ | 56 | 0.0 | 1 | 64 | 0.4 | 0.8 | 55 | -0.3 | 1 | 39 | -0.5 | 0.9 |
| Spo0A | *CD1233A* | CD630_12331 | hypothetical protein | 32 | -5.1 | 5.4x10^-14^ | 51 | -0.8 | 0.4 | 55 | -0.5 | 0.7 | 59 | -0.5 | 0.8 | 42 | -0.7 | 0.7 |
| σ^E^ | *glpQ* | CD630_14020 | glycerophosphoryl diester phosphodiesterase | 32 | -4.2 | 1.4x10^-6^ | 38 | -2.5 | 1.7x10^-3^ | 35 | -3.2 | 2.5x10^-5^ | 58 | -0.5 | 0.9 | 40 | -1.0 | 0.6 |
| ^σ^G^ | *CD2809* | CD630_28090 | hypothetical protein (DUF1540) | 32 | -4.1 | 3.6x10^-10^ | 34 | -4.1 | 2.6x10^-11^ | 62 | -0.1 | 1 | 37 | -3.5 | 1.0x10^-8^ | 43 | -0.6 | 1 |
| σ^E^ | *CD3638* | CD630_36380 | hypothetical protein | 32 | -3.0 | 1.6x10^-5^ | 33 | -2.9 | 3.7x10^-8^ | 31 | -4.2 | 4.6x10^-14^ | 46 | -1.1 | 0.1 | 34 | -1.2 | 0.2 |
| σ^E^ | *CD3456* | CD630_34560 | 5-formyltetrahydrofolate cyclo-ligase | 32 | -2.8 | 1.3x10^-5^ | 37 | -1.9 | 3.7x10^-4^ | 33 | -2.9 | 7.0x10^-9^ | 51 | -0.6 | 0.7 | 38 | -0.8 | 0.7 |
| Spo0A | *CD1404A* | CD630_14041 | hypothetical protein | 30 | -3.2 | 2.7x10^-4^ | 52 | -0.3 | 1 | 47 | -0.5 | 0.7 | 63 | 0.1 | 1 | 40 | -0.5 | 1 |
| σ^E^ | *CD0629* | CD630_06290 | Crp family transcriptional regulator | 30 | -3.1 | 8.5x10^-6^ | 33 | -2.3 | 1.5x10^-5^ | 31 | -3.2 | 1.9x10^-9^ | 45 | -1.0 | 0.2 | 34 | -1.0 | 0.5 |
| σ^E^ | *isp* | CD630_20000 | intracellular serine protease | 29 | -5.2 | 3.3x10^-11^ | 34 | -3.0 | 1.5x10^-8^ | 30 | -5.6 | 6.5x10^-19^ | 47 | -1.1 | 0.1 | 33 | -1.4 | 0.4 |
| σ^F^ | *CD2266* | CD630_22660 | oxidoreductase, FAD dependent | 29 | -3.8 | 2.0x10^-5^ | 36 | -2.0 | 7.8x10^-3^ | 38 | -1.6 | 0.01 | 45 | -1.1 | 0.3 | 37 | -0.9 | 0.8 |
| σ^E^ | *CD1555* | CD630_15550 | amino acid permease | 28 | -3.5 | 4.2x10^-7^ | 31 | -2.7 | 7.2x10^-7^ | 31 | -2.8 | 1.9x10^-8^ | 38 | -1.8 | 4.9x10^-4^ | 33 | -1.0 | 0.4 |
| σ^E^ | *pyrD* | CD630_31790 | dihydroorotate dehydrogenase, catalytic subunit | 28 | -3.4 | 8.1x10^-7^ | 32 | -2.5 | 7.6x10^-5^ | 30 | -3.1 | 7.9x10^-7^ | 49 | -0.5 | 0.9 | 41 | -0.2 | 1 |
| Spo0A | *CD1218* | CD630_12180 | glycosyl transferase family protein | 28 | -2.7 | 3.7x10^-5^ | 49 | -0.2 | 1 | 50 | 0.0 | 1 | 52 | -0.2 | 1 | 34 | -0.7 | 0.8 |
| Spo0A | *vexP3* | CD630_18750 | ABC transporter permease | 28 | -2.7 | 6.8x10^-5^ | 35 | -1.5 | 8.8x10^-3^ | 32 | -1.9 | 9.7x10^-5^ | 63 | 0.4 | 1 | 29 | -1.3 | 0.2 |
| Spo0A | *CD1233D* | CD630_12334 | hypothetical protein | 28 | -2.5 | 2.5x10^-5^ | 45 | -0.3 | 0.9 | 44 | -0.3 | 0.8 | 48 | -0.3 | 1 | 34 | -0.6 | 0.9 |
| Spo0A | *eutW* | CD630_19110 | two-component sensor histidine kinase, ethanolamine specific | 28 | -2.5 | 9.8x10^-5^ | 48 | -0.2 | 1 | 46 | -0.3 | 0.9 | 52 | -0.1 | 1 | 30 | -1.1 | 0.3 |
| Spo0A | *CD0573* | CD630_05730 | membrane protein (COG4399) | 28 | -2.4 | 2.1x10^-4^ | 34 | -1.4 | 0.02 | 37 | -1.0 | 0.1 | 41 | -0.9 | 0.4 | 30 | -1.1 | 0.4 |
| σ^F^ | *fruABC* | CD630_22690 | PTS system fructose-specific transporter subunit IIABC | 28 | -2.2 | 9.0x10^-4^ | 30 | -2.1 | 1.3x10^-4^ | 32 | -1.6 | 2.1x10^-3^ | 34 | -1.7 | 5.0x10^-3^ | 32 | -0.7 | 0.7 |
| σ^G^ | *CD0543* | CD630_05430 | hypothetical protein (DUF3298) | 27 | -2.0 | 0.04 | 28 | -2.2 | 0.04 | 42 | -0.2 | 1 | 29 | -2.6 | 0.03 | 29 | -0.8 | 0.8 |
| Spo0A | *CD0572* | CD630_05720 | sporulation protein | 26 | -3.6 | 0.01 | 34 | -1.5 | 0.3 | 34 | -1.5 | 0.3 | 38 | -1.3 | 0.7 | 29 | -1.3 | 0.6 |
| Spo0A | *CD3563* | CD630_35630 | spore cortex-lytic hydrolase | 26 | -2.6 | 2.5x10^-5^ | 33 | -1.3 | 0.05 | 37 | -0.7 | 0.3 | 37 | -1.1 | 0.2 | 35 | -0.2 | 1 |
| σ^E^ | *acpS* | CD630_34660 | 4'-phosphopantetheinyl transferase | 26 | -2.3 | 6.1x10^-4^ | 27 | -2.3 | 7.8x10^-5^ | 27 | -2.2 | 2.3x10^-5^ | 40 | -0.6 | 0.8 | 31 | -0.5 | 0.9 |
| σ^E^ | *CD3257* | CD630_32570 | polysaccharide deacetylase | 25 | -4.6 | 5.3x10^-10^ | 27 | -3.6 | 1.3x10^-9^ | 26 | -4.4 | 6.1x10^-13^ | 40 | -1.1 | 0.2 | 32 | -0.8 | 0.7 |
| σ^G^ | *CD2808* | CD630_28080 | hypothetical protein | 25 | -4.2 | 3.5x10^-9^ | 25 | -7.4 | 2.9x10^-17^ | 55 | 0.3 | 1 | 30 | -2.8 | 4.4x10^-8^ | 30 | -1.0 | 0.5 |
| σ^E^ | *CD1884* | CD630_18840 | hypothetical protein | 25 | -3.9 | 2.0x10^-7^ | 27 | -3.2 | 2.1x10^-8^ | 25 | -6.3 | 1.4x10^-13^ | 42 | -0.7 | 0.7 | 30 | -1.0 | 0.6 |
| Spo0A | *nudF* | CD630_12200 | NUDIX family hydrolase | 25 | -3.2 | 5.1x10^-6^ | 55 | 0.4 | 0.8 | 57 | 0.5 | 0.5 | 61 | 0.5 | 0.8 | 36 | -0.2 | 1 |
| σ^E^ | *CD1846* | CD630_18460 | conjugative transposon protein | 25 | -3.1 | 1.8x10^-3^ | 28 | -2.5 | 7.7x10^-3^ | 27 | -2.7 | 2.0x10^-3^ | 54 | 0.1 | 1 | 25 | -2.0 | 0.1 |
| σ^E^ | *mviN* | CD630_27810 | transmembrane virulence factor, MviN family protein | 25 | -2.6 | 2.5x10^-4^ | 26 | -2.7 | 1.5x10^-6^ | 25 | -3.0 | 4.2x10^-8^ | 34 | -1.3 | 0.1 | 29 | -0.8 | 0.7 |
| σ^K^ | *CD2346* | CD630_23460 | membrane protein | 24 | -2.9 | 1.9x10^-5^ | 29 | -1.5 | 0.01 | 26 | -2.3 | 7.6x10^-6^ | 53 | 0.4 | 1 | 22 | -2.1 | 0.01 |
| σ^E^ | *CD1741* | CD630_17410 | pseudo | 24 | -2.6 | 1.7x10^-4^ | 26 | -2.1 | 2.9x10^-4^ | 23 | -3.3 | 8.2x10^-8^ | 39 | -0.5 | 0.9 | 25 | -1.3 | 0.3 |
| ^σ^G^ | *sodA* | CD630_16310 | superoxide dismutase (Mn) | 23 | -5.7 | 2.3x10^-6^ | 24 | –Inf | 1.6x10^-9^ | 35 | -1.0 | 0.4 | 26 | –Inf | 6.1x10^-9^ | 31 | -0.7 | 0.9 |
| σ^G^ | *CD2636* | CD630_26360 | membrane protein (YIEGIA family) | 23 | -4.6 | 7.2x10^-9^ | 23 | -6.3 | 1.9x10^-15^ | 32 | -1.3 | 0.02 | 25 | -5.3 | 4.1x10^-16^ | 27 | -1.2 | 0.4 |
| σ^E^ | *nrdR* | CD630_26400 | NrdR family transcriptional regulator | 23 | -2.8 | 2.8x10^-4^ | 23 | -3.1 | 4.7x10^-7^ | 21 | -5.2 | 4.4x10^-13^ | 31 | -1.3 | 0.1 | 22 | -1.7 | 0.1 |
| Spo0A | *aroB* | CD630_18330 | 3-dehydroquinate synthase | 23 | -2.2 | 0.01 | 42 | 0.1 | 1 | 49 | 0.6 | 0.6 | 45 | 0.1 | 1 | 25 | -0.9 | 0.6 |
| σ^E^ | *CD1845* | CD630_18450 | membrane protein | 22 | -4.9 | 5.0x10^-3^ | 26 | -2.9 | 0.1 | 24 | -4.3 | 6.6x10^-3^ | 46 | -0.1 | 1 | 23 | -2.1 | 0.5 |
| ^σ^F^ | *CD1132* | CD630_11320 | heavy-metal transport/detoxification protein | 22 | -2.6 | 8.4x10^-5^ | 23 | -2.8 | 3.6x10^-6^ | 25 | -1.8 | 1.2x10^-3^ | 29 | -1.5 | 0.01 | 21 | -1.9 | 0.02 |
| Spo0A | *CD1233C* | CD630_12333 | regulator | 22 | -2.2 | 7.8x10^-4^ | 37 | -0.2 | 1 | 36 | -0.1 | 1 | 39 | -0.2 | 1 | 29 | -0.2 | 1 |
| Spo0A | *CD1819* | CD630_18190 | membrane protein | 22 | -2.1 | 0.03 | 39 | 0.2 | 1 | 37 | 0.1 | 1 | 42 | 0.2 | 1 | 26 | -0.5 | 1 |
| σ^G^ | *CD2315* | CD630_23150 | hypothetical protein (PIG-L superfamily) | 21 | -3.2 | 5.8x10^-5^ | 24 | -2.3 | 6.8x10^-5^ | 27 | -1.5 | 0.01 | 26 | -2.2 | 1.9x10^-4^ | 25 | -0.9 | 0.7 |
| Spo0A | *CD0549* | CD630_05490 | hypothetical protein (FH2 domain) | 21 | -3.0 | 5.6x10^-5^ | 54 | 0.8 | 0.4 | 65 | 1.3 | 0.1 | 41 | 0.0 | 1 | 29 | -0.3 | 1 |
| σ^E^ | *CD3234* | CD630_32340 | hypothetical protein (methyltransferase domain) | 21 | -2.2 | 5.0x10^-3^ | 22 | -2.3 | 2.0x10^-4^ | 19 | -4.1 | 8.2x10^-10^ | 32 | -0.6 | 0.8 | 25 | -0.5 | 0.9 |
| Spo0A | *CD1965* | CD630_19650 | permease | 21 | -2.0 | 7.1x10^-3^ | 34 | -0.1 | 1 | 34 | 0.0 | 1 | 37 | -0.1 | 1 | 21 | -1.1 | 0.5 |
| Spo0A | *CD0621* | CD630_06210 | membrane protein | 20 | -5.0 | 6.5x10^-3^ | 36 | -0.4 | 1 | 30 | -1.1 | 0.6 | 38 | -0.5 | 1 | 28 | -0.5 | 1 |
| σ^E^ | *spoIIIAC* | CD630_11940 | stage III sporulation protein AC | 20 | -4.3 | 3.8x10^-7^ | 20 | -4.4 | 2.0x10^-10^ | 19 | -6.7 | 1.8x10^-15^ | 32 | -0.9 | 0.4 | 26 | -0.7 | 0.8 |
| σ^E^ | *CD0556* | CD630_05560 | sugar isomerase / endonuclease | 20 | -2.9 | 1.3x10^-4^ | 27 | -1.2 | 0.1 | 23 | -2.3 | 9.9x10^-5^ | 33 | -0.7 | 0.8 | 25 | -0.6 | 0.9 |
| Spo0A | *eutV* | CD630_19100 | two-component response regulator, Ethanolamine specific | 20 | -2.7 | 6.8x10^-5^ | 33 | -0.4 | 0.9 | 32 | -0.4 | 0.9 | 36 | -0.3 | 1 | 24 | -0.8 | 0.7 |
| Spo0A | *CD1423* | CD630_14230 | hypothetical protein | 20 | -2.6 | 5.5x10^-4^ | 50 | 0.8 | 0.4 | 42 | 0.4 | 0.7 | 48 | 0.5 | 0.9 | 37 | 0.5 | 0.9 |
| σ^E^ | *CD1301* | CD630_13010 | membrane protein (TP0381 superfamily) | 19 | -3.7 | 2.5x10^-3^ | 20 | -3.2 | 3.3x10^-3^ | 19 | -4.3 | 7.7x10^-5^ | 30 | -0.9 | 0.7 | 20 | -1.5 | 0.5 |
| σ^E^ | *CD3150A* | CD630_31501 | hypothetical protein | 19 | -3.4 | 1.6x10^-4^ | 21 | -3.1 | 5.2x10^-4^ | 19 | -4.1 | 5.7x10^-6^ | 29 | -1.1 | 0.4 | 22 | -1.0 | 0.8 |
| σ^E^ | *CD2641* | CD630_26410 | sporulation protein | 19 | -3.3 | 5.2x10^-3^ | 20 | -3.3 | 8.7x10^-4^ | 18 | -5.5 | 4.7x10^-6^ | 26 | -1.6 | 0.3 | 19 | -1.9 | 0.3 |
| σ^E^ | *CD1724* | CD630_17240 | hypothetical protein (DUF3795) | 18 | -4.1 | 4.2x10^-4^ | 20 | -2.9 | 3.2x10^-3^ | 18 | -4.0 | 4.1x10^-4^ | 28 | -1.0 | 0.8 | 20 | -1.4 | 0.6 |
| σ^F^ | *CD3180* | CD630_31800 | purine permease | 18 | -3.1 | 0.03 | 19 | -3.2 | 0.03 | 21 | -2.3 | 0.1 | 30 | -0.6 | 1 | 26 | -0.2 | 1 |
| Spo0A | *CD2211* | CD630_22110 | ABC transporter multidrug-family ATP-binding/permease | 18 | -2.1 | 0.02 | 40 | 0.8 | 0.9 | 36 | 0.5 | 0.8 | 36 | 0.3 | 1 | 22 | -0.3 | 1 |
| σ^E^ | *spoIIIAD* | CD630_11950 | stage III sporulation protein AD | 17 | -4.4 | 1.4x10^-6^ | 18 | -5.4 | 1.6x10^-11^ | 18 | -4.9 | 1.5x10^-11^ | 29 | -0.9 | 0.4 | 22 | -0.9 | 0.7 |
| ^σ^G^ | *CD2841* | CD630_28410 | amidohydrolase | 17 | -3.5 | 1.1x10^-4^ | 18 | -3.4 | 7.1x10^-7^ | 27 | -0.7 | 0.5 | 20 | -3.1 | 1.6x10^-6^ | 18 | -1.7 | 0.2 |
| Spo0A | *CD0571* | CD630_05710 | hypothetical protein | 17 | -2.3 | 5.8x10^-3^ | 23 | -1.0 | 0.4 | 24 | -0.8 | 0.5 | 28 | -0.4 | 1 | 22 | -0.3 | 1 |
| σ^E^ | *CD2638* | CD630_26380 | two-component response regulator | 17 | -2.3 | 7.4x10^-3^ | 18 | -2.0 | 3.1x10^-3^ | 17 | -2.2 | 1.3x10^-3^ | 22 | -1.2 | 0.2 | 15 | -1.9 | 0.1 |
| σ^E^ | *CD1844A* | CD630_18441 | pseudo | 16 | -6.5 | 7.4x10^-7^ | 20 | -2.5 | 4.9x10^-3^ | 17 | -4.4 | 3.7x10^-5^ | 35 | 0.0 | 1 | 16 | -2.4 | 0.06 |
| σ^E^ | *CD1575* | CD630_15750 | hypothetical protein (DUF348, COG3584) | 16 | -3.7 | 2.4x10^-5^ | 19 | -2.0 | 3.3x10^-3^ | 16 | -3.8 | 4.9x10^-8^ | 30 | -0.2 | 1 | 20 | -0.8 | 0.8 |
| Spo0A | *CD1966* | CD630_19660 | acyl-CoA thioesterase | 15 | -4.1 | 4.6x10^-3^ | 31 | 0.1 | 1 | 28 | -0.1 | 1 | 26 | -0.7 | 1 | 17 | -1.2 | 0.6 |
| Spo0A | *spoIIR* | CD630_35640 | pro-σ^E^ endopeptidase (stage II sporulation) | 15 | -2.0 | 0.01 | 18 | -1.1 | 0.5 | 20 | -0.7 | 0.5 | 19 | -1.2 | 0.5 | 20 | 0.0 | 1 |
| σ^K^ | *CD0902* | CD630_09020 | cation efflux protein | 14 | -3.6 | 2.1x10^-3^ | 15 | -3.3 | 2.0x10^-3^ | 13 | -7.0 | 3.8x10^-6^ | 36 | 0.6 | 0.9 | 12 | -3.8 | 7.2x10^-3^ |
| σ^G^ | *CD1354* | CD630_13540 | hypothetical protein | 14 | -3.3 | 6.1x10^-4^ | 14 | -5.0 | 3.1x10^-9^ | 29 | 0.2 | 1 | 15 | -4.3 | 1.0x10^-8^ | 19 | -0.5 | 1 |
| ^σ^G^ | *CD24310* | CD630_24310 | nitrite/sulfite reductase | 13 | -3.8 | 1.6x10^-4^ | 15 | -2.4 | 1.2x10^-3^ | 18 | -1.2 | 0.2 | 16 | -2.3 | 1.4x10^-3^ | 17 | -0.6 | 0.9 |
| Spo0A | *CD1678* | CD630_16780 | membrane protein (DUF969) | 13 | -3.3 | 4.4x10^-3^ | 16 | -1.9 | 0.1 | 16 | -1.5 | 0.2 | 16 | -2.0 | 0.1 | 18 | -0.4 | 1 |
| σ^E^ | *CD2316* | CD630_23160 | two-component response regulator | 13 | -3.1 | 1.8x10^-3^ | 16 | -1.7 | 0.03 | 15 | -2.2 | 3.2x10^-3^ | 20 | -0.9 | 0.6 | 15 | -0.8 | 0.8 |
| Spo0A | *CD2142* | CD630_21420 | transporter | 13 | -3.1 | 0.02 | 15 | -1.8 | 0.2 | 17 | -1.2 | 0.3 | 19 | -0.9 | 0.8 | 14 | -1.1 | 0.8 |
| σ^E^ | *CD2445* | CD630_24450 | transmembrane signaling protein,TspO/MBR family | 13 | -2.2 | 0.03 | 16 | -1.1 | 0.4 | 13 | -2.6 | 6.4x10^-4^ | 24 | 0.0 | 1 | 16 | -0.3 | 1 |
| σ^E^ | *CD1726* | CD630_17260 | hypothetical protein | 12 | -7.1 | 8.5x10^-8^ | 15 | -2.6 | 8.3x10^-4^ | 13 | -4.5 | 1.1x10^-5^ | 20 | -1.0 | 0.5 | 16 | -1.0 | 0.7 |
| σ^E^ | *CD1929* | CD630_19290 | membrane protein | 12 | -4.4 | 5.2x10^-5^ | 13 | -3.5 | 1.1x10^-5^ | 13 | -3.5 | 1.1x10^-6^ | 19 | -0.9 | 0.7 | 14 | -1.1 | 0.6 |
| σ^G^ | *CD3551A* | CD630_35511 | membrane protein (DUF37) [[6](#_ENREF_6)] | 12 | -4.2 | 0.04 | 12 | -5.6 | 4.7x10^-3^ | 16 | -1.4 | 0.6 | 13 | -5.6 | 0.01 | 14 | -1.3 | 0.8 |
| σ^E^ | *CD0131* | CD630_01310 | membrane protein | 12 | -3.4 | 1.8x10^-3^ | 13 | -3.0 | 1.2x10^-4^ | 12 | -5.3 | 1.1x10^-8^ | 18 | -1.1 | 0.4 | 12 | -1.8 | 0.2 |
| Spo0A | *CD2657* | CD630_26570 | hypothetical protein | 12 | -3.2 | 1.5x10^-3^ | 21 | -0.1 | 1 | 16 | -1.1 | 0.3 | 25 | 0.1 | 1 | 14 | -0.9 | 0.8 |
| Spo0A | *CD1239* | CD630_12390 | beta-lactams repressor | 12 | -2.9 | 2.5x10^-3^ | 15 | -1.3 | 0.2 | 17 | -0.8 | 0.4 | 17 | -1.1 | 0.5 | 12 | -1.8 | 0.2 |
| σ^G^ | *CD1028* | CD630_10280 | signaling protein | 12 | -2.8 | 3.6x10^-3^ | 17 | -1.1 | 0.3 | 19 | -0.6 | 0.9 | 14 | -2.6 | 1.5x10^-3^ | 23 | 0.5 | 0.9 |
| σ^K^ | *CD2409* | CD630_24090 | hypothetical protein | 11 | -3.8 | 8.5x10^-4^ | 14 | -1.7 | 0.1 | 14 | -1.6 | 0.03 | 24 | 0.2 | 1 | 10 | -2.7 | 0.02 |
| σ^F^ | *CD1297* | CD630_12970 | hypothetical protein (DUF2953) | 11 | -3.4 | 9.6x10^-4^ | 11 | -5.3 | 6.6x10^-8^ | 19 | -0.3 | 1 | 15 | -1.7 | 0.05 | 14 | -0.6 | 0.9 |
| ^σ^G^(σ^E^) | *CD2598* | CD630_25980 | oligosaccharide deacetylase | 11 | -3.2 | 1.8x10^-3^ | 11 | -5.2 | 9.2x10^-8^ | 12 | -2.4 | 5.0x10^-4^ | 12 | -3.9 | 3.0x10^-6^ | 13 | -1.0 | 0.7 |
| σ^G^ | *CD1298* | CD630_12980 | hypothetical protein (YtfJ sporulation protein, [[7](#_ENREF_7)]) | 11 | -3.0 | 3.4x10^-3^ | 11 | -6.2 | 2.1x10^-8^ | 22 | 0.2 | 1 | 14 | -2.2 | 8.8x10^-3^ | 13 | -1.0 | 0.8 |
| ^σ^G^ | *CD1595A* | CD630_15951 | ferredoxin | 11 | -2.9 | 3.8x10^-3^ | 12 | -2.3 | 4.4x10^-3^ | 15 | -1.1 | 0.3 | 13 | -2.4 | 6.0x10^-3^ | 15 | -0.3 | 1 |
| σ^E^ | *CD1185* | CD630_11850 | diguanylate kinase signaling protein | 11 | -2.5 | 0.02 | 12 | -2.0 | 0.02 | 11 | -2.6 | 2.3x10^-3^ | 16 | -1.0 | 0.7 | 10 | -2.0 | 0.2 |
| Spo0A | *mnaA* | CD630_10330 | UDP-N-acetylglucosamine 2-epimerase | 11 | -2.5 | 5.8x10^-3^ | 14 | -1.5 | 0.1 | 13 | -1.6 | 0.1 | 18 | -0.6 | 0.9 | 13 | -0.9 | 0.8 |
| Spo0A | *CD1170* | CD630_11700 | hypothetical protein | 11 | -2.3 | 0.03 | 17 | -0.2 | 1 | 13 | -1.3 | 0.2 | 16 | -0.7 | 0.9 | 14 | -0.2 | 1 |
| σ^E^ | *cwlD* | CD630_01060 | Germination-specific N-acetylmuramoyl-L-alanine amidase, Autolysin | 10 | -4.9 | 4.1x10^-5^ | 13 | -2.3 | 4.2x10^-3^ | 11 | -4.2 | 3.0x10^-6^ | 18 | -0.8 | 0.8 | 12 | -1.1 | 0.7 |
| ^σ^G^(σ^E^) | *CD0214* | CD630_02140 | hypothetical protein | 10 | -3.3 | 1.6x10^-3^ | 11 | -2.9 | 7.3x10^-4^ | 10 | -3.6 | 2.7x10^-5^ | 11 | -3.1 | 3.9x10^-4^ | 11 | -1.4 | 0.6 |
| Spo0A | *CD1045* | CD630_10450 | sporulation integral membrane protein | 9 | -3.0 | 4.5x10^-3^ | 11 | -1.8 | 0.1 | 11 | -1.6 | 0.1 | 13 | -1.2 | 0.5 | 10 | -1.0 | 0.8 |
| ^σ^E^ | *CD2865* | CD630_28650 | bacterioferritin | 9 | -2.9 | 0.03 | 11 | -1.6 | 0.3 | 10 | -2.7 | 0.02 | 16 | -0.3 | 1 | 12 | -0.5 | 1 |
| σ^E^ | *ddl* | CD630_14080 | D-Ala-D-Ala ligase | 9 | -2.3 | 0.04 | 11 | -1.5 | 0.2 | 10 | -2.0 | 0.03 | 16 | -0.2 | 1 | 9 | -1.4 | 0.6 |
| ^σ^F^ | *CD2245A* | CD630_22451 | hypothetical protein (Yqz-like) | 8 | -6.5 | 2.2x10^-5^ | 8 | -5.9 | 1.7x10^-5^ | 11 | -1.5 | 0.1 | 11 | -2.3 | 0.1 | 10 | -1.3 | 0.7 |
| σ^E^ | *CD1063* | CD630_10630 | hypothetical protein | 8 | -4.5 | 0.01 | 10 | -2.0 | 0.2 | 8 | –Inf | 5.0x10^-4^ | 17 | -0.1 | 1 | 9 | -1.8 | 0.7 |
| σ^E^ | *spmA* | CD630_35420 | spore maturation protein A | 8 | -3.5 | 7.6x10^-3^ | 9 | -3.6 | 1.7x10^-4^ | 8 | –Inf | 1.1x10^-7^ | 14 | -0.8 | 0.8 | 9 | -1.4 | 0.6 |
| σ^E^ | *CD2055* | CD630_20550 | hypothetical protein | 7 | -3.7 | 0.02 | 10 | -1.2 | 0.5 | 8 | -3.1 | 0.01 | 28 | 1.4 | 0.2 | 6 | -2.7 | 0.1 |
| σ^E^ | *CD3636* | CD630_36360 | membrane protein | 7 | -3.3 | 0.05 | 7 | -4.5 | 2.7x10^-3^ | 6 | -5.9 | 1.0x10^-3^ | 10 | -1.0 | 0.8 | 6 | -2.0 | 0.4 |
| σ^E^ | *CD3368* | CD630_33680 | ribosome biogenesis GTPase RsgA; putative EngC-like GTPase | 7 | -2.9 | 0.05 | 8 | -2.2 | 0.1 | 8 | -3.2 | 0.02 | 15 | 0.0 | 1 | 6 | -2.7 | 0.1 |
| Spo0A | *CD0620* | CD630_06200 | hypothetical protein | 6 | -5.1 | 1.6x10^-3^ | 11 | -0.3 | 1 | 9 | -1.4 | 0.4 | 10 | -1.0 | 0.8 | 8 | -0.8 | 1 |
| σ^G^ | *CD0793* | CD630_07930 | hypothetical protein | 6 | -4.3 | 3.6x10^-3^ | 6 | -5.6 | 1.6x10^-4^ | 7 | -1.8 | 0.1 | 7 | -3.5 | 4.7x10^-3^ | 6 | -2.3 | 0.3 |
| ^σ^G^ | *CD2599* | CD630_25990 | transcriptional regulator | 5 | -3.4 | 0.04 | 5 | -4.1 | 2.7x10^-3^ | 7 | -1.7 | 0.2 | 6 | -3.0 | 0.03 | 6 | -1.0 | 0.9 |
| σ^E^ | *CD0213* | CD630_02130 | spore coat protein | 5 | -3.4 | 0.05 | 6 | -2.1 | 0.3 | 5 | -3.6 | 0.04 | 6 | -1.9 | 0.5 | 6 | -1.1 | 0.9 |
| Spo0A | *CD0619* | CD630_06190 | hypothetical protein | 4 | -5.5 | 0.01 | 8 | 0.0 | 1 | 6 | -1.3 | 0.6 | 9 | 0.0 | 1 | 8 | 0.3 | 1 |
| σ^G^ | *spoVAC* | CD630_07730 | stage V sporulation protein AC | 4 | -4.0 | 0.05 | 4 | -5.2 | 8.8x10^-3^ | 6 | -1.0 | 0.7 | 5 | -5.5 | 0.02 | 4 | -1.9 | 0.6 |

^†^ Two factors are listed in the table for genes whose expression was dependent on both σ^E^ and σ^G^ (adjusted p-value ≤ 0.05, log_2_FC ≤ -2). *Dep.* indicates the most downstream sigma factor on which gene expression depends upon. *BM* refers to base mean, the mean of the counts after they were divided by the size factors to adjust for different sequencing depths. This value is the mean for the sample relative to wild type. *log_2_FC* denotes log_2_fold-change. A negative value indicates that the gene was downregulated relative to wild type. ^ Indicates that gene product was detected in Lawley *et al*. proteomic analysis of purified spores [[8](#_ENREF_8)]. *–Inf* indicates that no transcript was detected in the mutant relative to wild type. See Text S2 for the references.
