# Supplementary material for: Global Analysis of the Sporulation Pathway of Clostridium difficile
Source: PLoS Genet. 2013 Aug 8;9(8):e1003660. doi: 10.1371/journal.pgen.1003660 (PMC3738446; doi:10.1371/journal.pgen.1003660)
Supplement: Table S4 — Sporulation-related genes. † Two factors are listed in the table for genes whose expression was dependent on both σE and σG (adjusted p-value≤0.05, log2FC≤−2). Dep. indicates the most downstream sigma factor on which gene expression depends upon. BM refers to base mean, the mean of the counts after they were divided by the size factors to adjust for different sequencing depths. This value is the mean for the sample relative to wild type. log2FC denotes log2fold-change. A negative value indicates that the gene was downregulated relative to wild type. ∧ Indicates that gene product was detected in Lawley et al. proteomic analysis of purified spores [70]. −Inf indicates that no transcript was detected in the mutant relative to wild type. See Text S2 for the references. (DOCX) [file pgen.1003660.s011.docx]

**Table S4. Genes induced during sporulation in a Spo0A-, σ^F^-, σ^E^-, σ^G^-, and/or σ^K^-dependent manner.**

|  |  |  |  | **Spo0A** | | | **σ^F^** | | | **σ^E^** | | | **σ^G^** | | | **σ^K^** | | |
| --- | --- | --- | --- | --- | --- | --- | --- | --- | --- | --- | --- | --- | --- | --- | --- | --- | --- | --- |
| **Dep.^†^** | **Name** | **locus_tag** | **description** | **BM** | **log_2_FC** | **adjP** | **BM** | **log_2_FC** | **adjP** | **BM** | **log_2_FC** | **adjP** | **BM** | **log_2_FC** | **adjP** | **BM** | **log_2_FC** | **adjP** |
| σ^E^ | *alr2* | CD630_34630 | alanine racemase | 583 | -3.8 | 1.7x10^-20^ | 620 | -3.6 | 1.9x10^-21^ | 590 | -4.6 | 1.2x10^-42^ | 999 | -0.7 | 0.3 | 739 | -0.8 | 0.5 |
| ^σ^K^ | *bclA1* | CD630_03320 | exosporium glycoprotein | 57 | -5.4 | 3.3x10^-6^ | 66 | -3.2 | 4.8x10^-4^ | 59 | -7.1 | 7.2x10^-10^ | 110 | -0.4 | 1 | 49 | -5.6 | 6.6x10^-7^ |
| σ^E^ | *bclA2* | CD630_32300 | exosporium glycoprotein | 42 | -4.2 | 0.1 | 48 | -2.9 | 0.2 | 42 | -5.9 | 0.01 | 92 | 0.0 | 1 | 36 | -4.8 | 0.1 |
| σ^K^ | *bclA3* | CD630_33490 | exosporium glycoprotein BclA3 | 2155 | -6.1 | 5.9x10^-11^ | 2467 | -3.3 | 4.9x10^-5^ | 2247 | -5.7 | 1.3x10^-10^ | 4455 | -0.2 | 1 | 1867 | -5.5 | 2.2x10^-9^ |
| σ^F^ | *CD0125* | CD630_01250 | [cell wall endopeptidase (SpoIIQ homolog, [5])](#RANGE!_ENREF_5) | 46 | -4.2 | 1.3x10^-12^ | 49 | -3.6 | 3.4x10^-13^ | 101 | 0.3 | 1 | 72 | -1.1 | 0.05 | 58 | -0.8 | 0.6 |
| σ^E^ | *CD0129* | CD630_01290 | hypothetical protein (YyaC, in sporulating bacteria) | 105 | -5.1 | 8.9x10^-23^ | 126 | -2.5 | 6.7x10^-10^ | 109 | -5.1 | 3.0x10^-33^ | 198 | -0.5 | 0.8 | 148 | -0.5 | 0.9 |
| ^σ^K^ | *CD0596* | CD630_05960 | hypothetical protein (CotJA homolog) | 67 | -7.0 | 1.2x10^-5^ | 75 | -3.7 | 4.7x10^-3^ | 69 | -7.5 | 7.3x10^-7^ | 159 | 0.2 | 1 | 58 | -6.1 | 1.4x10^-4^ |
| Spo0A | *CD1045* | CD630_10450 | sporulation integral membrane protein | 9 | -3.0 | 4.5x10^-3^ | 11 | -1.8 | 0.1 | 11 | -1.6 | 0.1 | 13 | -1.2 | 0.5 | 10 | -1.0 | 0.8 |
| σ^E^ | *CD1168* | CD630_11680 | [membrane protein (spore coat, YlbJ [4])](#RANGE!_ENREF_4) | 117 | -6.0 | 9.7x10^-27^ | 134 | -3.2 | 7.2x10^-15^ | 120 | -6.8 | 8.0x10^-46^ | 201 | -0.9 | 0.1 | 143 | -1.1 | 0.3 |
| Spo0A | *CD1221* | CD630_12210 | membrane protein (SpoIIM homolog) | 63 | -5.6 | 4.4x10^-21^ | 106 | -0.7 | 0.5 | 112 | -0.4 | 0.8 | 120 | -0.5 | 0.8 | 83 | -0.8 | 0.6 |
| σ^F^ | *CD1290* | CD630_12900 | small acid-soluble spore protein SASP | 3 | -4.1 | 0.1 | 3 | –Inf | 6.5x10^-3^ | 5 | -1.0 | 0.7 | 4 | -2.5 | 0.2 | 4 | -1.3 | 0.9 |
| σ^G^ | *CD1298* | CD630_12980 | [hypothetical protein (YtfJ sporulation protein [7])](#RANGE!_ENREF_8) | 11 | -3.0 | 3.4x10^-3^ | 11 | -6.2 | 2.1x10^-8^ | 22 | 0.2 | 1 | 14 | -2.2 | 8.8x10^-3^ | 13 | -1.0 | 0.8 |
| σ^E^ | *CD1321* | CD630_13210 | sporulation protein (YlmC) | 72 | -2.1 | 6.7x10^-6^ | 72 | -2.6 | 4.4x10^-9^ | 68 | -3.1 | 5.5x10^-13^ | 95 | -1.2 | 0.01 | 70 | -1.4 | 0.06 |
| ^σ^K^ | *CD1433* | CD630_14330 | peroxiredoxin/chitinase (coat protein "CotE," [1]) | 2018 | -7.7 | 1.4x10^-56^ | 2343 | -3.2 | 2.2x10^-18^ | 2112 | -6.4 | 2.8x10^-74^ | 3959 | -0.4 | 0.8 | 1753 | -5.8 | 1.2x10^-43^ |
| ^σ^E^ | *CD1511* | CD630_15110 | [hypothetical protein (coat protein - "CotB," [1])](#RANGE!_ENREF_1) | 137 | -5.5 | 1.3x10^-25^ | 163 | -2.7 | 1.3x10^-11^ | 146 | -4.5 | 3.5x10^-32^ | 270 | -0.3 | 1 | 200 | -0.4 | 1 |
| ^σ^E^ | *CD1613* | CD630_16130 | hypothetical protein (coat protein "CotA," [1]) | 151 | -4.2 | 6.7x10^-3^ | 174 | -2.7 | 0.1 | 161 | -3.6 | 0.02 | 341 | 0.2 | 1 | 135 | -3.4 | 0.1 |
| σ^K^ | *CD2144* | CD630_21440 | putative sporulation membrane protein YtaF | 46 | -6.7 | 8.8x10^-11^ | 58 | -2.3 | 1.8x10^-3^ | 49 | -4.8 | 1.4x10^-9^ | 95 | -0.2 | 1 | 40 | -6.0 | 8.1x10^-10^ |
| σ^F^ | *CD2376* | CD630_23760 | [membrane protein YtvI involved in sporulation [4]](#RANGE!_ENREF_4) | 74 | -3.7 | 3.1x10^-13^ | 81 | -3.1 | 7.1x10^-13^ | 105 | -1.1 | 8.1x10^-3^ | 111 | -1.3 | 4.7x10^-3^ | 97 | -0.6 | 0.7 |
| σ^E^ | *CD2641* | CD630_26410 | sporulation protein | 19 | -3.3 | 5.2x10^-3^ | 20 | -3.3 | 8.7x10^-4^ | 18 | -5.5 | 4.7x10^-6^ | 26 | -1.6 | 0.3 | 19 | -1.9 | 0.3 |
| σ^F^ | *CD2685* | CD630_26850 | sporulation stage II, protein E | 16 | -0.6 | 0.8 | 13 | -2.0 | 0.02 | 15 | -1.1 | 0.3 | 15 | -1.5 | 0.2 | 12 | -1.2 | 0.6 |
| σ^E^ | *CD3464* | CD630_34640 | hypothetical protein YdcC involved in sporulation {Feucht, 2003 #284} | 557 | -4.6 | 1.5x10^-26^ | 609 | -3.6 | 2.3x10^-21^ | 574 | -5.0 | 2.3x10^-42^ | 965 | -0.7 | 0.2 | 698 | -0.9 | 0.2 |
| Spo0A | *CD3563* | CD630_35630 | spore cortex-lytic hydrolase | 26 | -2.6 | 2.5x10^-5^ | 33 | -1.3 | 0.05 | 37 | -0.7 | 0.3 | 37 | -1.1 | 0.2 | 35 | -0.2 | 1 |
| Spo0A | *CD3569* | CD630_35690 | sporulation-specific protease (YabG) | 42 | -2.7 | 3.4x10^-7^ | 59 | -0.9 | 0.2 | 50 | -1.7 | 2.1x10^-4^ | 98 | 0.5 | 0.8 | 44 | -1.3 | 0.1 |
| ^σ^E^ | *cotJB1* | CD630_05970 | spore coat peptide assembly protein | 33 | -4.9 | 0.02 | 37 | -3.5 | 0.1 | 34 | -6.8 | 2.8x10^-3^ | 78 | 0.2 | 1 | 29 | -4.8 | 0.1 |
| ^σ^K^ | *cotJB2* | CD630_24000 | spore coat peptide assembly protein CotJB2 | 797 | -8.5 | 2.2x10^-57^ | 953 | -2.9 | 9.6x10^-15^ | 833 | -6.8 | 4.6x10^-57^ | 1537 | -0.5 | 0.7 | 692 | -6.2 | 5.1x10^-44^ |
| ^σ^K^ | *cotJC1* | CD630_05980 | [spore coat assembly protein ("CotCB," [1])](#RANGE!_ENREF_1) | 63 | -4.8 | 0.03 | 69 | -3.7 | 0.1 | 63 | -6.7 | 2.7x10^-3^ | 151 | 0.3 | 1 | 53 | -6.2 | 0.02 |
| ^σ^K^ | *cotJC2* | CD630_24010 | spore coat assembly protein CotJC2 ("CotD," [1]) | 720 | -7.8 | 7.4x10^-53^ | 869 | -2.7 | 1.2x10^-13^ | 753 | -6.4 | 5.3x10^-63^ | 1398 | -0.5 | 0.7 | 625 | -5.9 | 4.3x10^-42^ |
| ^σ^E^ | *cspBA* | CD630_22470 | subtilisin-like germination related protease | 173 | -3.1 | 9.5x10^-8^ | 188 | -2.7 | 6.7x10^-11^ | 173 | -3.7 | 4.2x10^-14^ | 270 | -0.9 | 0.1 | 197 | -1.0 | 0.2 |
| ^σ^E^ | *cspC* | CD630_22460 | subtilisin-like germination-related protease | 239 | -4.1 | 3.4x10^-18^ | 264 | -3.2 | 8.3x10^-17^ | 243 | -5.0 | 8.8x10^-41^ | 401 | -0.8 | 0.1 | 276 | -1.3 | 0.07 |
| σ^E^ | *cwlD* | CD630_01060 | Germination-specific N-acetylmuramoyl-L-alanine amidase, Autolysin | 10 | -4.9 | 4.1x10^-5^ | 13 | -2.3 | 4.2x10^-3^ | 11 | -4.2 | 3.0x10^-6^ | 18 | -0.8 | 0.8 | 12 | -1.1 | 0.7 |
| ^σ^G^ | *dacF* | CD630_12910 | D-alanyl-D-alanine carboxypeptidase | 35 | -4.5 | 3.4x10^-11^ | 37 | -4.6 | 7.1x10^-13^ | 76 | 0.2 | 1 | 38 | -5.5 | 1.1x10^-14^ | 45 | -0.8 | 0.8 |
| σ^K^ | *dpaA* | CD630_29680 | dipicolinate synthase subunit A | 249 | -5.7 | 1.9x10^-31^ | 286 | -3.1 | 5.7x10^-16^ | 257 | -6.0 | 3.5x10^-52^ | 506 | -0.3 | 1 | 214 | -5.4 | 1.1x10^-31^ |
| ^σ^F^ | *gpr* | CD630_24700 | germination protease | 36 | -5.7 | 1.6x10^-3^ | 39 | -4.4 | 3.5x10^-3^ | 72 | 0.0 | 1 | 51 | -1.8 | 0.5 | 43 | -1.1 | 0.8 |
| Spo0A | *sigE* | CD630_26430 | sporulation factor σ^E^ | 64 | -4.6 | 7.0x10^-16^ | 205 | 1.1 | 0.01 | 160 | 0.6 | 0.4 | 144 | 0.1 | 1 | 101 | -0.1 | 1 |
| Spo0A | *sigF* | CD630_07720 | sporulation factor σ^F^ | 304 | -4.9 | 1.7x10^-27^ | 763 | 0.6 | 0.5 | 740 | 0.5 | 0.5 | 731 | 0.3 | 1 | 470 | -0.2 | 1 |
| ^Spo0A | *sigG* | CD630_26420 | sporulation factor σ^G^ | 127 | -5.4 | 1.5x10^-24^ | 356 | 0.8 | 0.2 | 205 | -0.8 | 0.1 | 294 | 0.1 | 1 | 187 | -0.4 | 1 |
| σ^K^ | *sigK* | CD630_12300 | sporulation factor σ^K^ | 68 | -4.6 | 4.8x10^-17^ | 80 | -2.5 | 8.1x10^-9^ | 68 | -7.2 | 4.8x10^-35^ | 138 | -0.2 | 1 | 61 | -3.6 | 1.7x10^-12^ |
| ^σ^E^ | *sipL* | CD630_35670 | cell wall hydrolase (binds SpoIVA, [2]) | 882 | -5.8 | 2.0x10^-36^ | 1036 | -2.9 | 8.3x10^-15^ | 937 | -4.6 | 9.5x10^-35^ | 1505 | -0.9 | 0.05 | 1072 | -1.2 | 0.2 |
| ^σ^K^ | *sleC* | CD630_05510 | spore cortex-lytic enzyme prx10-pro-form | 356 | -7.0 | 5.9x10^-42^ | 417 | -3.1 | 6.4x10^-16^ | 372 | -6.4 | 2.4x10^-59^ | 750 | -0.2 | 1 | 307 | -6.2 | 3.4x10^-39^ |
| ^σ^G^ | *sodA* | CD630_16310 | superoxide dismutase (Mn) | 23 | -5.7 | 2.3x10^-6^ | 24 | –Inf | 1.6x10^-9^ | 35 | -1.0 | 0.4 | 26 | –Inf | 6.1x10^-9^ | 31 | -0.7 | 0.9 |
| σ^E^ | *spmA* | CD630_35420 | spore maturation protein A | 8 | -3.5 | 7.6x10^-3^ | 9 | -3.6 | 1.7x10^-4^ | 8 | –Inf | 1.1x10^-7^ | 14 | -0.8 | 0.8 | 9 | -1.4 | 0.6 |
| ^Spo0A | *spo0A* | CD630_12140 | stage 0 sporulation protein A | 722 | -2.5 | 3.2x10^-10^ | 1266 | 0.0 | 1 | 1187 | -0.2 | 0.9 | 1302 | -0.2 | 1 | 1054 | 0.0 | 1 |
| Spo0A | *spoIIAA* | CD630_07700 | anti-σ^F^ factor antagonist | 200 | -4.2 | 1.3x10^-20^ | 471 | 0.5 | 0.7 | 439 | 0.3 | 0.8 | 453 | 0.2 | 1 | 305 | -0.2 | 1 |
| Spo0A | *spoIIAB* | CD630_07710 | anti-σ^F^ factor | 374 | -4.4 | 8.1x10^-25^ | 863 | 0.4 | 0.8 | 881 | 0.5 | 0.6 | 874 | 0.2 | 1 | 571 | -0.2 | 1 |
| σ^E^ | *spoIID* | CD630_01240 | stage II sporulation protein D | 33 | -2.7 | 6.4x10^-6^ | 39 | -1.8 | 6.7x10^-4^ | 35 | -2.6 | 7.0x10^-8^ | 54 | -0.6 | 0.7 | 38 | -1.0 | 0.5 |
| Spo0A | *spoIIE* | CD630_34900 | phosphoprotein phosphatase | 507 | -6.0 | 2.0x10^-25^ | 1224 | 0.4 | 0.7 | 1390 | 0.7 | 0.1 | 1238 | 0.3 | 1 | 751 | -0.4 | 0.9 |
| Spo0A | *spoIIGA* | CD630_26440 | sporulation σ^E^-processing peptidase | 46 | -4.3 | 6.3x10^-13^ | 126 | 0.8 | 0.2 | 119 | 0.7 | 0.3 | 99 | 0.0 | 1 | 64 | -0.5 | 0.9 |
| σ^E^ | *spoIIIAA* | CD630_11920 | stage III sporulation protein AA | 261 | -6.7 | 7.3x10^-38^ | 287 | -4.2 | 2.9x10^-25^ | 271 | -6.9 | 5.9x10^-59^ | 418 | -1.2 | 1.3x10^-3^ | 326 | -1.1 | 0.1 |
| σ^E^ | *spoIIIAB* | CD630_11930 | stage III sporulation protein AB | 153 | -6.4 | 6.2x10^-31^ | 168 | -4.1 | 6.2x10^-23^ | 158 | -7.2 | 4.5x10^-52^ | 242 | -1.3 | 1.1x10^-3^ | 199 | -0.9 | 0.3 |
| σ^E^ | *spoIIIAC* | CD630_11940 | stage III sporulation protein AC | 20 | -4.3 | 3.8x10^-7^ | 20 | -4.4 | 2.0x10^-10^ | 19 | -6.7 | 1.8x10^-15^ | 32 | -0.9 | 0.4 | 26 | -0.7 | 0.8 |
| σ^E^ | *spoIIIAD* | CD630_11950 | stage III sporulation protein AD | 17 | -4.4 | 1.4x10^-6^ | 18 | -5.4 | 1.6x10^-11^ | 18 | -4.9 | 1.5x10^-11^ | 29 | -0.9 | 0.4 | 22 | -0.9 | 0.7 |
| σ^E^ | *spoIIIAE* | CD630_11960 | stage III sporulation protein AE | 47 | -4.9 | 1.8x10^-14^ | 52 | -3.6 | 4.4x10^-13^ | 49 | -5.1 | 7.8x10^-22^ | 76 | -1.0 | 0.1 | 63 | -0.7 | 0.8 |
| σ^E^ | *spoIIIAF* | CD630_11970 | stage III sporulation protein AF | 55 | -5.0 | 5.8x10^-17^ | 61 | -3.6 | 2.4x10^-14^ | 56 | -6.2 | 5.6x10^-30^ | 94 | -0.8 | 0.2 | 69 | -1.0 | 0.3 |
| σ^E^ | *spoIIIAG* | CD630_11980 | stage III sporulation protein AG | 164 | -5.9 | 6.9x10^-29^ | 189 | -3.2 | 1.3x10^-15^ | 171 | -6.1 | 7.2x10^-48^ | 284 | -0.8 | 0.1 | 210 | -1.0 | 0.3 |
| σ^E^ | *spoIIIAH* | CD630_11990 | stage III sporulation protein AH | 162 | -6.2 | 3.2x10^-28^ | 187 | -3.2 | 2.1x10^-15^ | 170 | -5.4 | 7.9x10^-26^ | 286 | -0.8 | 0.2 | 208 | -0.9 | 0.6 |
| σ^E^ | *spoIIID* | CD630_01260 | stage III sporulation protein D | 160 | -7.2 | 2.0x10^-25^ | 183 | -3.5 | 7.4x10^-13^ | 167 | -6.8 | 2.2x10^-24^ | 289 | -0.7 | 0.4 | 190 | -1.4 | 0.2 |
| σ^F^ | *spoIIP* | CD630_24690 | stage II sporulation protein P | 7 | -2.4 | 0.1 | 8 | -2.2 | 0.03 | 14 | 0.1 | 1 | 11 | -1.0 | 0.7 | 9 | -0.5 | 1 |
| Spo0A | *spoIIR* | CD630_35640 | pro-σ^E^ endopeptidase (stage II sporulation) | 15 | -2.0 | 0.01 | 18 | -1.1 | 0.5 | 20 | -0.7 | 0.5 | 19 | -1.2 | 0.5 | 20 | 0.0 | 1 |
| σ^E^ | *spoIV* | CD630_24420 | stage IV sporulation protein | 36 | -5.8 | 5.1x10^-15^ | 41 | -3.1 | 8.5x10^-10^ | 38 | -4.6 | 3.3x10^-18^ | 67 | -0.6 | 0.7 | 47 | -0.9 | 0.6 |
| ^σ^E^ | *spoIVA* | CD630_26290 | stage IV sporulation protein A | 665 | -7.3 | 3.6x10^-48^ | 764 | -3.4 | 3.1x10^-19^ | 695 | -6.5 | 1.1x10^-62^ | 1121 | -1.0 | 0.01 | 824 | -1.1 | 0.2 |
| σ^G^ | *spoVAC* | CD630_07730 | stage V sporulation protein AC | 4 | -4.0 | 0.05 | 4 | -5.2 | 8.8x10^-3^ | 6 | -1.0 | 0.7 | 5 | -5.5 | 0.02 | 4 | -1.9 | 0.6 |
| ^σ^G^ | *spoVAD* | CD630_07740 | stage V sporulation protein AD | 46 | -4.2 | 5.7x10^-13^ | 48 | -5.1 | 1.9x10^-19^ | 66 | -1.2 | 7.7x10^-3^ | 52 | -4.7 | 3.3x10^-22^ | 58 | -0.9 | 0.6 |
| Spo0A | *spoVD* | CD630_26560 | stage V sporulation protein D (Sporulation-specific penicillin-binding protein) | 312 | -2.7 | 1.1x10^-9^ | 610 | 0.2 | 1 | 551 | -0.1 | 1 | 589 | -0.1 | 1 | 447 | -0.1 | 1 |
| Spo0A | *spoVE* | CD630_26520 | cell division/stage V sporulation protein | 420 | -3.1 | 3.8x10^-15^ | 846 | 0.2 | 1 | 936 | 0.5 | 0.5 | 924 | 0.2 | 1 | 574 | -0.4 | 0.9 |
| σ^K^ | *spoVFB* | CD630_29670 | dipicolinate synthase subunit B | 177 | -4.8 | 2.7x10^-24^ | 202 | -3.1 | 9.6x10^-15^ | 181 | -5.6 | 1.1x10^-44^ | 347 | -0.3 | 1 | 153 | -4.6 | 3.1x10^-24^ |
| ^σ^G^ | *spoVT* | CD630_34990 | stage V sporulation protein T | 33 | -5.5 | 2.5x10^-13^ | 36 | -3.6 | 3.1x10^-11^ | 51 | -0.9 | 0.1 | 38 | -4.5 | 4.4x10^-17^ | 41 | -1.0 | 0.4 |
| σ^E^ | *ssb* | CD630_32350 | single-stranded DNA-binding protein | 36 | -4.8 | 3.2x10^-12^ | 41 | -3.0 | 4.7x10^-9^ | 37 | -5.4 | 7.3x10^-21^ | 67 | -0.5 | 0.8 | 52 | -0.4 | 1 |
| ^σ^G^ | *sspA* | CD630_26880 | Small, acid-soluble spore protein alpha | 412 | -5.8 | 2.2x10^-25^ | 436 | -5.4 | 1.5x10^-24^ | 546 | -1.8 | 5.0x10^-5^ | 466 | -5.5 | 1.1x10^-24^ | 557 | -0.7 | 0.8 |
| ^σ^G^(σ^E^) | *sspB* | CD630_32490 | Small, acid-soluble spore protein beta | 97 | -8.1 | 1.8x10^-11^ | 102 | -7.0 | 1.2x10^-11^ | 115 | -2.8 | 6.4x10^-4^ | 109 | -7.0 | 7.3x10^-11^ | 129 | -0.8 | 1 |
|  | *dapA* | CD630_30000 | dihydrodipicolinate synthase 1 | 13 | 1.9 | 0.04 | 6 | 0.1 | 1 | 8 | 1.0 | 0.7 | 5 | 0.0 | 1 | 8 | 0.6 | 1 |
|  | *oxaA1* | CD630_36780 | Stage III sporulation protein SpoIIIJ | 235 | 0.5 | 0.6 | 207 | 0.1 | 1 | 188 | -0.2 | 1 | 164 | 0.0 | 1 | 223 | 0.1 | 1 |
|  | *spmB* | CD630_35410 | Spore maturation protein SpmB | 3 | -1.8 | 0.7 | 4 | -1.6 | 0.6 | 3 | -3.1 | 0.1 | 4 | -0.8 | 1 | 5 | -0.4 | 1 |
|  | *spoIVB* | CD630_12130 | Stage IV sporulation protein SpoIVB | 100 | -1.1 | 0.3 | 129 | -0.3 | 1 | 125 | -0.4 | 0.9 | 105 | -0.3 | 1 | 138 | -0.4 | 1 |
|  | *spoIVCA* | CD630_19050 | Site-specific recombinase (putative SpoIVCA homolog) | 6 | -1.6 | 0.6 | 6 | -1.9 | 0.2 | 6 | -2.1 | 0.1 | 7 | -0.4 | 1 | 9 | -0.3 | 1 |
|  | *spoVB* | CD630_34980 | Stage V sporulation protein SpoVB | 38 | -1.8 | 5.5x10^-3^ | 50 | -0.7 | 0.5 | 47 | -0.8 | 0.2 | 43 | -0.5 | 0.9 | 53 | -0.7 | 0.6 |
|  | *spoVC* | CD630_35200 | Pth, Peptidyl-tRNA hydrolase SpoVC homolog | 11 | -0.2 | 1 | 10 | -0.8 | 0.8 | 10 | -0.7 | 0.8 | 8 | -0.9 | 0.9 | 11 | -0.6 | 1 |
|  | *spoVM* | Not annotated | stage V sporulation protein M (SpoVM homolog, [5]) | 12 | -0.6 | 0.9 | 15 | -0.1 | 1 | 12 | -0.8 | 0.7527 | 9 | -1.0 | 0.8 | 14 | -0.4 | 1 |
|  | *yabP* | CD630_34940 | Spore cortex protein YabP | 34 | -0.6 | 0.4 | 36 | -0.6 | 0.7 | 36 | -0.5 | 0.6 | 30 | -0.5 | 0.9 | 45 | 0.0 | 1 |
|  | *yqfC* | CD630_24430 | Spore cortex protein YqfC, YabP family | 2 | -4.6 | 0.4 | 2 | -3.9 | 0.5 | 2 | –Inf | 0.3 | 2 | -2.0 | 1.0 | 5 | 0.0 | 1 |
|  | *yqfD* | Unk | Stage IV sporulation protein YqfD |  |  |  |  |  |  |  |  |  |  |  |  |  |  |  |
|  | *yabQ* | Unk | Spore cortex formation membrane protein YabQ | |  |  |  |  |  |  |  |  |  |  |  |  |  |  |
|  | *spoIIIE* | Unk | Stage III sporulation protein SpoIIIE |  |  |  |  |  |  |  |  |  |  |  |  |  |  |  |
|  | *yabQ* | Unk | Spore cortex formation membrane protein YabQ | |  |  |  |  |  |  |  |  |  |  |  |  |  |  |
|  | *gerM* | Unk | Spore germination and sporulation regulator GerM | |  |  |  |  |  |  |  |  |  |  |  |  |  |  |
|  | *obgE* | Unk | GTPase |  |  |  |  |  |  |  |  |  |  |  |  |  |  |  |
|  | *stoA* | Unk | Thiol-disulfide oxidoreductase |  |  |  |  |  |  |  |  |  |  |  |  |  |  |  |

^†^ Two factors are listed in the table for genes whose expression was dependent on both σ^E^ and σ^G^ (adjusted p-value ≤ 0.05, log_2_FC ≤ -2). *Dep.* indicates the most downstream sigma factor on which gene expression depends upon. *BM* refers to base mean, the mean of the counts after they were divided by the size factors to adjust for different sequencing depths. This value is the mean for the sample relative to wild type. *log_2_FC* denotes log_2_fold-change. A negative value indicates that the gene was downregulated relative to wild type. ^ Indicates that gene product was detected in Lawley *et al*. proteomic analysis of purified spores [[8](#_ENREF_7)]. *–Inf* indicates that no transcript was detected in the mutant relative to wild type. See Text S2 for the references.
