# Supplementary material for: Global Analysis of the Sporulation Pathway of Clostridium difficile
Source: PLoS Genet. 2013 Aug 8;9(8):e1003660. doi: 10.1371/journal.pgen.1003660 (PMC3738446; doi:10.1371/journal.pgen.1003660)
Supplement: Table S5 — σF-dependent genes. † Two factors are listed in the table for genes whose expression was dependent on both σE and σG (adjusted p-value≤0.05, log2FC≤−2). Dep. indicates the most downstream sigma factor on which gene expression depends upon. BM refers to base mean, the mean of the counts after they were divided by the size factors to adjust for different sequencing depths. This value is the mean for the sample relative to wild type. log2FC denotes log2fold-change. A negative value indicates that the gene was downregulated relative to wild type. ∧ Indicates that gene product was detected in Lawley et al. proteomic analysis of purified spores [70]. −Inf indicates that no transcript was detected in the mutant relative to wild type. See Text S2 for the references. (DOCX) [file pgen.1003660.s012.docx]

**Table S5. σ^F^-dependent genes.**

|  |  |  |  | σ^F^ | | | **Spo0A** | | | σ^E^ | | | **σ^G^** | | | **σ^K^** | | |
| --- | --- | --- | --- | --- | --- | --- | --- | --- | --- | --- | --- | --- | --- | --- | --- | --- | --- | --- |
| **Dep.**^†^ | **Name** | **locus_tag** | **description** | **BM** | **log_2_FC** | **adjP** | **BM** | **log_2_FC** | **adjP** | **BM** | **log_2_FC** | **adjP** | **BM** | **log_2_FC** | **adjP** | **BM** | **log_2_FC** | **adjP** |
| ^σ^K^ | *CD1067* | CD630_10670 | hypothetical protein | 11923 | -2.7 | 3.9x10^-14^ | 9888 | -7.6 | 1.3x10^-60^ | 10380 | -6.1 | 1.5x10^-50^ | 21003 | -0.2 | 1 | 8776 | -4.7 | 2.4x10^-32^ |
| σ^K^ | *bclA3* | CD630_33490 | exosporium glycoprotein BclA3 | 2467 | -3.3 | 4.9x10^-5^ | 2155 | -6.1 | 5.9x10^-11^ | 2247 | -5.7 | 1.3x10^-10^ | 4455 | -0.2 | 1 | 1867 | -5.5 | 2.2x10^-9^ |
| ^σ^K^ | *CD1433* | CD630_14330 | peroxiredoxin/chitinase (coat protein "CotE," [1]) | 2343 | -3.2 | 2.2x10^-18^ | 2018 | -7.7 | 1.4x10^-56^ | 2112 | -6.4 | 2.8x10^-74^ | 3959 | -0.4 | 0.8 | 1753 | -5.8 | 1.2x10^-43^ |
| σ^K^ | *CD1063C* | CD630_10633 | hypothetical protein | 1879 | -2.7 | 5.8x10^-7^ | 1552 | -7.5 | 4.2x10^-27^ | 1632 | -6.0 | 2.9x10^-20^ | 3104 | -0.4 | 0.9 | 1369 | -4.9 | 3.4x10^-15^ |
| σ^K^ | *CD1063B* | CD630_10632 | hypothetical protein | 1802 | -2.7 | 4.7x10^-7^ | 1496 | -7.5 | 4.7x10^-27^ | 1569 | -6.2 | 3.8x10^-21^ | 3044 | -0.3 | 1 | 1317 | -5.1 | 2.2x10^-15^ |
| σ^E^ | *CD0311* | CD630_03110 | hypothetical protein | 1086 | -3.0 | 5.2x10^-16^ | 926 | -6.8 | 2.3x10^-46^ | 966 | -6.4 | 3.1x10^-68^ | 1715 | -0.6 | 0.4 | 1077 | -1.4 | 4.4x10^-3^ |
| ^σ^E^ | *sipL* | CD630_35670 | cell wall hydrolase (binds SpoIVA, [2]) | 1036 | -2.9 | 8.3x10^-15^ | 882 | -5.8 | 2.0x10^-36^ | 937 | -4.6 | 9.5x10^-35^ | 1505 | -0.9 | 0.05 | 1072 | -1.2 | 0.2 |
| ^σ^K^ | *cotJB2* | CD630_24000 | spore coat peptide assembly protein CotJB2 | 953 | -2.9 | 9.6x10^-15^ | 797 | -8.5 | 2.2x10^-57^ | 833 | -6.8 | 4.6x10^-57^ | 1537 | -0.5 | 0.7 | 692 | -6.2 | 5.1x10^-44^ |
| ^σ^K^ | *cotJC2* | CD630_24010 | spore coat assembly protein CotJC2 ("CotD," [1]) | 869 | -2.7 | 1.2x10^-13^ | 720 | -7.8 | 7.4x10^-53^ | 753 | -6.4 | 5.3x10^-63^ | 1398 | -0.5 | 0.7 | 625 | -5.9 | 4.3x10^-42^ |
| ^σ^E^ | *spoIVA* | CD630_26290 | stage IV sporulation protein A | 764 | -3.4 | 3.1x10^-19^ | 665 | -7.3 | 3.6x10^-48^ | 695 | -6.5 | 1.1x10^-62^ | 1121 | -1.0 | 0.01 | 824 | -1.1 | 0.2 |
| σ^E^ | *alr2* | CD630_34630 | alanine racemase | 620 | -3.6 | 1.9x10^-21^ | 583 | -3.8 | 1.7x10^-20^ | 590 | -4.6 | 1.2x10^-42^ | 999 | -0.7 | 0.3 | 739 | -0.8 | 0.5 |
| σ^K^ | *feoB* | CD630_15170 | ferrous iron transport protein B | 613 | -2.4 | 4.1x10^-8^ | 541 | -3.3 | 1.8x10^-12^ | 549 | -3.7 | 1.5x10^-14^ | 1125 | 0.0 | 1 | 475 | -3.0 | 3.0x10^-10^ |
| σ^E^ | *CD3464* | CD630_34640 | hypothetical protein YdcC involved in sporulation [3] | 609 | -3.6 | 2.3x10^-21^ | 557 | -4.6 | 1.5x10^-26^ | 574 | -5.0 | 2.3x10^-42^ | 965 | -0.7 | 0.2 | 698 | -0.9 | 0.2 |
| σ^K^ | *CD1065* | CD630_10650 | hypothetical protein | 495 | -2.7 | 2.3x10^-10^ | 411 | -7.6 | 8.1x10^-32^ | 429 | -7.3 | 6.5x10^-33^ | 769 | -0.6 | 0.5 | 394 | -3.0 | 3.0x10^-11^ |
| ^σ^G^ | *sspA* | CD630_26880 | Small, acid-soluble spore protein alpha | 436 | -5.4 | 1.5x10^-24^ | 412 | -5.8 | 2.2x10^-25^ | 546 | -1.8 | 5.0x10^-5^ | 466 | -5.5 | 1.1x10^-24^ | 557 | -0.7 | 0.8 |
| ^σ^K^ | *CD2399* | CD630_23990 | hypothetical protein (CotJA superfamily) | 427 | -3.1 | 4.0x10^-6^ | 364 | -8.9 | 4.2x10^-21^ | 379 | -7.6 | 3.5x10^-19^ | 702 | -0.5 | 0.9 | 314 | -7.0 | 4.0x10^-16^ |
| σ^E^ | *CD3183* | CD630_31830 | peptidase | 417 | -2.3 | 1.3x10^-9^ | 516 | -0.8 | 0.1 | 397 | -2.6 | 6.2x10^-16^ | 536 | -1.1 | 3.0x10^-3^ | 460 | -0.7 | 0.6 |
| ^σ^K^ | *sleC* | CD630_05510 | spore cortex-lytic enzyme prx10-pro-form | 417 | -3.1 | 6.4x10^-16^ | 356 | -7.0 | 5.9x10^-42^ | 372 | -6.4 | 2.4x10^-59^ | 750 | -0.2 | 1 | 307 | -6.2 | 3.4x10^-39^ |
| ^σ^G^ | *CD2112* | CD630_21120 | hypothetical protein | 391 | -4.5 | 1.6x10^-28^ | 368 | -4.9 | 3.2x10^-28^ | 633 | -0.5 | 0.4 | 414 | -4.9 | 1.4x10^-35^ | 411 | -1.5 | 5.2x10^-3^ |
| σ^E^ | *dapG* | CD630_13220 | aspartate kinase I | 378 | -2.0 | 6.3x10^-8^ | 351 | -2.2 | 7.9x10^-8^ | 349 | -2.6 | 7.9x10^-17^ | 483 | -1.1 | 8.8x10^-3^ | 357 | -1.2 | 0.06 |
| σ^K^ | *CD0564* | CD630_05640 | ATP-dependent protease | 343 | -2.1 | 1.8x10^-8^ | 343 | -1.8 | 5.6x10^-6^ | 328 | -2.4 | 2.1x10^-13^ | 487 | -0.7 | 0.3 | 276 | -2.2 | 4.0x10^-8^ |
| σ^F^ | *polA* | CD630_11280 | DNA polymerase I | 317 | -2.0 | 3.2x10^-7^ | 282 | -2.5 | 3.7x10^-9^ | 314 | -1.9 | 2.8x10^-9^ | 421 | -0.8 | 0.1 | 309 | -1.0 | 0.2 |
| ^σ^E^ | *CD3522* | CD630_35220 | hypothetical protein | 317 | -4.0 | 2.1x10^-6^ | 286 | -6.1 | 2.8x10^-10^ | 298 | -6.0 | 8.7x10^-11^ | 449 | -1.3 | 0.2 | 312 | -1.8 | 0.2 |
| σ^E^ | *spoIIIAA* | CD630_11920 | stage III sporulation protein AA | 287 | -4.2 | 2.9x10^-25^ | 261 | -6.7 | 7.3x10^-38^ | 271 | -6.9 | 5.9x10^-59^ | 418 | -1.2 | 1.3x10^-3^ | 326 | -1.1 | 0.1 |
| σ^K^ | *dpaA* | CD630_29680 | dipicolinate synthase subunit A | 286 | -3.1 | 5.7x10^-16^ | 249 | -5.7 | 1.9x10^-31^ | 257 | -6.0 | 3.5x10^-52^ | 506 | -0.3 | 1 | 214 | -5.4 | 1.1x10^-31^ |
| ^σ^E^ | *cspC* | CD630_22460 | subtilisin-like germination-related protease | 264 | -3.2 | 8.3x10^-17^ | 239 | -4.1 | 3.4x10^-18^ | 243 | -5.0 | 8.8x10^-41^ | 401 | -0.8 | 0.1 | 276 | -1.3 | 0.07 |
| σ^E^ | *brnQ-1* | CD630_12590 | Branched chain amino acid transport system carrier protein | 235 | -2.0 | 2.4x10^-7^ | 185 | -5.0 | 9.5x10^-25^ | 207 | -3.2 | 1.8x10^-19^ | 368 | -0.3 | 1 | 301 | -0.1 | 1 |
| σ^K^ | *spoVFB* | CD630_29670 | dipicolinate synthase subunit B | 202 | -3.1 | 9.6x10^-15^ | 177 | -4.8 | 2.7x10^-24^ | 181 | -5.6 | 1.1x10^-44^ | 347 | -0.3 | 1 | 153 | -4.6 | 3.1x10^-24^ |
| σ^E^ | *spoIIIAG* | CD630_11980 | stage III sporulation protein AG | 189 | -3.2 | 1.3x10^-15^ | 164 | -5.9 | 6.9x10^-29^ | 171 | -6.1 | 7.2x10^-48^ | 284 | -0.8 | 0.1 | 210 | -1.0 | 0.3 |
| ^σ^E^ | *cspBA* | CD630_22470 | subtilisin-like germination related protease | 188 | -2.7 | 6.7x10^-11^ | 173 | -3.1 | 9.5x10^-8^ | 173 | -3.7 | 4.2x10^-14^ | 270 | -0.9 | 0.1 | 197 | -1.0 | 0.2 |
| σ^E^ | *spoIIIAH* | CD630_11990 | stage III sporulation protein AH | 187 | -3.2 | 2.1x10^-15^ | 162 | -6.2 | 3.2x10^-28^ | 170 | -5.4 | 7.9x10^-26^ | 286 | -0.8 | 0.2 | 208 | -0.9 | 0.6 |
| σ^E^ | *spoIIID* | CD630_01260 | stage III sporulation protein D | 183 | -3.5 | 7.4x10^-13^ | 160 | -7.2 | 2.0x10^-25^ | 167 | -6.8 | 2.2x10^-24^ | 289 | -0.7 | 0.4 | 190 | -1.4 | 0.2 |
| σ^E^ | *CD1068* | CD630_10680 | polysaccharide biosynthesis/sporulation protein | 180 | -2.3 | 1.2x10^-8^ | 150 | -4.1 | 2.1x10^-19^ | 154 | -4.5 | 2.2x10^-32^ | 253 | -0.8 | 0.2 | 190 | -0.8 | 0.6 |
| σ^E^ | *CD3182* | CD630_31820 | D-aminoacylase | 177 | -2.1 | 9.1x10^-4^ | 200 | -1.1 | 0.1 | 169 | -2.4 | 2.0x10^-4^ | 248 | -0.7 | 0.7 | 200 | -0.5 | 0.9 |
| σ^E^ | *spoIIIAB* | CD630_11930 | stage III sporulation protein AB | 168 | -4.1 | 6.2x10^-23^ | 153 | -6.4 | 6.2x10^-31^ | 158 | -7.2 | 4.5x10^-52^ | 242 | -1.3 | 1.1x10^-3^ | 199 | -0.9 | 0.3 |
| ^σ^E^ | *CD2864* | CD630_28640 | hydrolase | 168 | -2.8 | 1.8x10^-12^ | 143 | -5.3 | 1.9x10^-25^ | 148 | -5.6 | 4.3x10^-41^ | 267 | -0.5 | 0.6 | 183 | -0.9 | 0.3 |
| ^σ^E^ | *CD1511* | CD630_15110 | [hypothetical protein (coat protein - "CotB," [1])](#RANGE!_ENREF_1) | 163 | -2.7 | 1.3x10^-11^ | 137 | -5.5 | 1.3x10^-25^ | 146 | -4.5 | 3.5x10^-32^ | 270 | -0.3 | 1 | 200 | -0.4 | 1 |
| σ^E^ | *CD1403* | CD630_14030 | synthetase | 159 | -2.0 | 5.5x10^-7^ | 135 | -3.1 | 3.8x10^-12^ | 145 | -2.8 | 9.9x10^-15^ | 230 | -0.6 | 0.5 | 177 | -0.5 | 0.9 |
| σ^E^ | *CD2441A* | CD630_24411 | phoH-like protein | 148 | -2.8 | 7.2x10^-12^ | 136 | -3.3 | 4.4x10^-13^ | 139 | -3.5 | 9.4x10^-23^ | 225 | -0.7 | 0.3 | 161 | -0.9 | 0.4 |
| σ^F^ | *CD2375* | CD630_23750 | hypothetical protein (DUF1540) | 143 | -2.5 | 5.1x10^-10^ | 119 | -5.2 | 5.8x10^-24^ | 240 | 0.0 | 1 | 174 | -1.6 | 1.4x10^-5^ | 171 | -0.5 | 1 |
| σ^E^ | *CD3462* | CD630_34620 | antitoxin endoAI | 136 | -3.0 | 4.2x10^-13^ | 126 | -3.4 | 1.7x10^-13^ | 128 | -4.0 | 1.1x10^-18^ | 216 | -0.6 | 0.5 | 159 | -0.7 | 0.7 |
| σ^E^ | *CD1380* | CD630_13800 | transporter, Major Facilitator Superfamily (MFS) | 134 | -2.2 | 2.0x10^-8^ | 114 | -3.5 | 9.7x10^-13^ | 130 | -2.4 | 1.4x10^-11^ | 184 | -0.9 | 0.1 | 114 | -1.9 | 6.1x10^-5^ |
| σ^E^ | *CD1168* | CD630_11680 | [membrane protein (spore coat, YlbJ [4])](#RANGE!_ENREF_4) | 134 | -3.2 | 7.2x10^-15^ | 117 | -6.0 | 9.7x10^-27^ | 120 | -6.8 | 8.0x10^-46^ | 201 | -0.9 | 0.1 | 143 | -1.1 | 0.3 |
| σ^E^ | *CD3181* | CD630_31810 | chlorohydrolase/aminohydrolase | 130 | -2.7 | 1.5x10^-8^ | 122 | -2.8 | 2.7x10^-9^ | 128 | -2.7 | 3.0x10^-8^ | 193 | -0.7 | 0.4 | 156 | -0.5 | 1 |
| σ^E^ | *dpaL* | CD630_31840 | diaminopropionate ammonia-lyase | 127 | -2.1 | 2.0x10^-3^ | 184 | -0.2 | 1 | 118 | -2.6 | 2.7x10^-4^ | 163 | -1.0 | 0.5 | 139 | -0.6 | 0.9 |
| σ^E^ | *CD0129* | CD630_01290 | hypothetical protein (YyaC, in sporulating bacteria) | 126 | -2.5 | 6.7x10^-10^ | 105 | -5.1 | 8.9x10^-23^ | 109 | -5.1 | 3.0x10^-33^ | 198 | -0.5 | 0.8 | 148 | -0.5 | 0.9 |
| ^σ^K^ | *CD1133* | CD630_11330 | hypothetical protein | 119 | -2.2 | 7.1x10^-7^ | 96 | -4.9 | 8.8x10^-21^ | 101 | -4.5 | 1.7x10^-18^ | 255 | 0.5 | 0.6 | 86 | -3.6 | 9.0x10^-13^ |
| σ^E^ | *CD2833* | CD630_28330 | calcium-transporting ATPase | 119 | -2.7 | 1.1x10^-10^ | 100 | -4.8 | 1.7x10^-18^ | 102 | -6.1 | 9.7x10^-40^ | 184 | -0.6 | 0.6 | 124 | -1.0 | 0.4 |
| σ^E^ | *CD1928* | CD630_19280 | membrane protein | 114 | -3.4 | 7.2x10^-16^ | 100 | -7.4 | 2.0x10^-28^ | 105 | -6.0 | 5.7x10^-38^ | 152 | -1.5 | 1.4x10^-4^ | 119 | -1.3 | 0.1 |
| ^σ^E^ | *CD1319* | CD630_13190 | polysaccharide deacetylase | 113 | -3.7 | 7.9x10^-18^ | 103 | -5.0 | 1.4x10^-21^ | 107 | -5.2 | 3.5x10^-34^ | 155 | -1.4 | 2.9x10^-4^ | 126 | -1.1 | 0.2 |
| σ^E^ | *CD2084* | CD630_20840 | peptidase | 113 | -2.1 | 3.0x10^-7^ | 137 | -0.8 | 0.1 | 109 | -2.3 | 2.8x10^-10^ | 138 | -1.3 | 2.8x10^-3^ | 125 | -0.6 | 0.8 |
| σ^E^ | *CD3298* | CD630_32980 | ATP/GTP-binding protein | 112 | -2.6 | 1.0x10^-5^ | 92 | -6.6 | 2.6x10^-16^ | 97 | -5.6 | 4.7x10^-15^ | 168 | -0.7 | 0.6 | 107 | -1.4 | 0.3 |
| σ^E^ | *EndoA* | CD630_34610 | endoribonuclease toxin | 112 | -3.0 | 9.6x10^-12^ | 103 | -3.5 | 3.9x10^-13^ | 104 | -4.1 | 4.8x10^-17^ | 181 | -0.5 | 0.7 | 139 | -0.5 | 0.9 |
| σ^E^ | *CD1066* | CD630_10660 | hypothetical protein | 105 | -2.7 | 2.4x10^-10^ | 91 | -4.0 | 1.8x10^-15^ | 95 | -4.1 | 4.6x10^-24^ | 149 | -0.9 | 0.1 | 96 | -1.7 | 2.3x10^-3^ |
| σ^E^ | *CD3177* | CD630_31770 | xanthine dehydrogenase | 103 | -3.0 | 4.8x10^-8^ | 90 | -4.5 | 1.2x10^-11^ | 98 | -3.5 | 3.5x10^-9^ | 153 | -0.8 | 0.3 | 125 | -0.6 | 1 |
| ^σ^G^(σ^E^) | *sspB* | CD630_32490 | Small, acid-soluble spore protein beta | 102 | -7.0 | 1.2x10^-11^ | 97 | -8.1 | 1.8x10^-11^ | 115 | -2.8 | 6.4x10^-4^ | 109 | -7.0 | 7.3x10^-11^ | 129 | -0.8 | 1 |
| ^σ^E^ | *CD3269* | CD630_32690 | oligoendopeptidase F, M3B family | 100 | -2.1 | 7.1x10^-7^ | 130 | -0.5 | 0.4 | 97 | -2.1 | 4.6x10^-9^ | 149 | -0.4 | 0.9 | 90 | -1.4 | 0.01 |
| σ^E^ | *dnaX* | CD630_00160 | DNA polymerase III subunits gamma and tau | 97 | -2.4 | 1.5x10^-4^ | 85 | -3.4 | 4.2x10^-7^ | 90 | -3.2 | 9.3x10^-7^ | 141 | -0.7 | 0.6 | 102 | -0.9 | 0.6 |
| σ^E^ | *CD2800* | CD630_28000 | membrane protein | 95 | -3.2 | 1.7x10^-13^ | 84 | -4.5 | 2.4x10^-18^ | 86 | -5.4 | 2.5x10^-32^ | 132 | -1.1 | 0.01 | 96 | -1.4 | 0.03 |
| σ^E^ | *CD1930* | CD630_19300 | hypothetical protein (ComEC-related) | 94 | -2.7 | 1.5x10^-10^ | 99 | -1.8 | 4.8x10^-5^ | 85 | -4.1 | 1.6x10^-24^ | 149 | -0.5 | 0.8 | 102 | -0.9 | 0.6 |
| ^σ^E^ | *CD0761* | CD630_07610 | ATP-dependent RNA helicase | 89 | -2.4 | 1.3x10^-8^ | 81 | -2.8 | 9.7x10^-10^ | 83 | -2.9 | 1.3x10^-14^ | 111 | -1.4 | 1.1x10^-3^ | 90 | -1.0 | 0.4 |
| ^σ^K^ | *CD1063A* | CD630_10631 | hypothetical protein | 86 | -2.7 | 3.3x10^-3^ | 71 | -7.7 | 6.8x10^-9^ | 74 | -8.3 | 3.1x10^-10^ | 167 | 0.1 | 1 | 63 | -4.8 | 2.5x10^-5^ |
| σ^E^ | *CD1940* | CD630_19400 | membrane protein (DUF3866 superfamily) | 84 | -3.1 | 8.8x10^-13^ | 73 | -6.3 | 3.4x10^-23^ | 78 | -4.4 | 1.6x10^-24^ | 128 | -0.8 | 0.3 | 106 | -0.5 | 0.9 |
| σ^E^ | *CD1398* | CD630_13980 | peptidase, M20D family | 82 | -2.6 | 1.7x10^-9^ | 69 | -4.9 | 2.4x10^-18^ | 74 | -4.1 | 2.7x10^-22^ | 119 | -0.8 | 0.2 | 83 | -1.2 | 0.2 |
| σ^F^ | *CD2376* | CD630_23760 | [membrane protein YtvI involved in sporulation [4]](#RANGE!_ENREF_4) | 81 | -3.1 | 7.1x10^-13^ | 74 | -3.7 | 3.1x10^-13^ | 105 | -1.1 | 8.1x10^-3^ | 111 | -1.3 | 4.7x10^-3^ | 97 | -0.6 | 0.7 |
| σ^K^ | *sigK* | CD630_12300 | sporulation factor σ^K^ | 80 | -2.5 | 8.1x10^-9^ | 68 | -4.6 | 4.8x10^-17^ | 68 | -7.2 | 4.8x10^-35^ | 138 | -0.2 | 1 | 61 | -3.6 | 1.7x10^-12^ |
| ^σ^G^ | *CD2687A* | CD630_26871 | hypothetical protein | 80 | -3.6 | 1.0x10^-15^ | 73 | -4.9 | 3.0x10^-18^ | 118 | -0.7 | 0.1 | 98 | -2.1 | 7.0x10^-8^ | 92 | -0.9 | 0.4 |
| σ^K^ | *feoA* | CD630_15180 | ferrous iron transport protein | 79 | -3.2 | 9.2x10^-8^ | 70 | -4.7 | 2.0x10^-12^ | 72 | -5.2 | 3.9x10^-14^ | 149 | 0.0 | 1 | 60 | -4.7 | 3.0x10^-11^ |
| ^σ^G^ | *CD1486* | CD630_14860 | ribosome recycling factor | 75 | -5.0 | 6.4x10^-23^ | 71 | -5.8 | 4.2x10^-21^ | 125 | -0.4 | 0.7 | 80 | -5.5 | 1.3x10^-27^ | 86 | -1.1 | 0.3 |
| ^σ^K^ | *CD0596* | CD630_05960 | hypothetical protein (CotJA homolog) | 75 | -3.7 | 4.7x10^-3^ | 67 | -7.0 | 1.2x10^-5^ | 69 | -7.5 | 7.3x10^-7^ | 159 | 0.2 | 1 | 58 | -6.1 | 1.4x10^-4^ |
| σ^E^ | *CD3465* | CD630_34650 | hypothetical protein (CBS domain) | 72 | -2.9 | 4.0x10^-10^ | 69 | -2.8 | 1.7x10^-8^ | 71 | -3.0 | 1.9x10^-11^ | 109 | -0.7 | 0.3 | 89 | -0.5 | 0.9 |
| σ^E^ | *CD1321* | CD630_13210 | sporulation protein (YlmC) | 72 | -2.6 | 4.4x10^-9^ | 72 | -2.1 | 6.7x10^-6^ | 68 | -3.1 | 5.5x10^-13^ | 95 | -1.2 | 0.01 | 70 | -1.4 | 0.06 |
| ^σ^E^ | *CD3258* | CD630_32580 | Iron hydrogenase | 71 | -4.1 | 1.3x10^-17^ | 66 | -4.9 | 3.3x10^-16^ | 69 | -5.0 | 8.4x10^-28^ | 103 | -1.2 | 8.8x10^-3^ | 71 | -1.8 | 1.5x10^-3^ |
| ^σ^K^ | *CD3580* | CD630_35800 | hypothetical protein | 68 | -3.0 | 9.2x10^-11^ | 59 | -5.6 | 1.5x10^-19^ | 62 | -5.1 | 6.6x10^-24^ | 111 | -0.5 | 0.8 | 51 | -4.7 | 2.7x10^-16^ |
| σ^E^ | *CD0760* | CD630_07600 | Ca^2+^/Na^+^ antiporter | 68 | -2.2 | 8.6x10^-5^ | 58 | -3.6 | 9.9x10^-10^ | 59 | -3.9 | 4.7x10^-10^ | 90 | -1.0 | 0.1 | 64 | -1.3 | 0.1 |
| ^σ^K^ | *bclA1* | CD630_03320 | exosporium glycoprotein | 66 | -3.2 | 4.8x10^-4^ | 57 | -5.4 | 3.3x10^-6^ | 59 | -7.1 | 7.2x10^-10^ | 110 | -0.4 | 1 | 49 | -5.6 | 6.6x10^-7^ |
| σ^E^ | *CD2121* | CD630_21210 | hypothetical protein | 64 | -2.7 | 5.1x10^-4^ | 54 | -5.4 | 2.9x10^-8^ | 56 | -5.5 | 2.1x10^-8^ | 96 | -0.7 | 0.8 | 62 | -1.4 | 0.4 |
| σ^E^ | *spoIIIAF* | CD630_11970 | stage III sporulation protein AF | 61 | -3.6 | 2.4x10^-14^ | 55 | -5.0 | 5.8x10^-17^ | 56 | -6.2 | 5.6x10^-30^ | 94 | -0.8 | 0.2 | 69 | -1.0 | 0.3 |
| σ^E^ | *CD3178* | CD630_31780 | D-hydantoinase | 60 | -2.8 | 2.4x10^-9^ | 54 | -3.8 | 3.2x10^-11^ | 57 | -3.4 | 3.6x10^-11^ | 83 | -1.1 | 0.05 | 65 | -0.9 | 0.5 |
| ^σ^G^ | *CD0684* | CD630_06840 | ATP-dependent peptidase, M41 family | 60 | -4.3 | 7.9x10^-18^ | 58 | -3.7 | 6.9x10^-12^ | 115 | 0.1 | 1 | 65 | -3.9 | 6.9x10^-20^ | 67 | -1.2 | 0.2 |
| σ^K^ | *CD0896* | CD630_08960 | hypothetical protein | 59 | -3.6 | 4.5x10^-3^ | 52 | -6.8 | 5.7x10^-6^ | 57 | -4.4 | 6.4x10^-4^ | 100 | -0.5 | 1 | 46 | -5.6 | 4.8x10^-4^ |
| σ^E^ | *CD2637* | CD630_26370 | two-component sensor histidine kinase | 59 | -2.2 | 3.2x10^-6^ | 56 | -2.2 | 5.2x10^-5^ | 55 | -2.6 | 1.5x10^-7^ | 75 | -1.1 | 0.04 | 56 | -1.2 | 0.4 |
| σ^K^ | *CD2144* | CD630_21440 | putative sporulation membrane protein YtaF | 58 | -2.3 | 1.8x10^-3^ | 46 | -6.7 | 8.8x10^-11^ | 49 | -4.8 | 1.4x10^-9^ | 95 | -0.2 | 1 | 40 | -6.0 | 8.1x10^-10^ |
| σ^F^ | *CD1130* | CD630_11300 | lytic transglycosylase-like protein | 58 | -2.0 | 3.3x10^-5^ | 51 | -2.7 | 1.2x10^-6^ | 58 | -1.8 | 8.7x10^-6^ | 76 | -0.9 | 0.2 | 53 | -1.3 | 0.1 |
| σ^E^ | *CD1320* | CD630_13200 | M16 family peptidase | 58 | -2.6 | 2.3x10^-4^ | 56 | -2.3 | 7.4x10^-4^ | 55 | -2.9 | 2.9x10^-5^ | 75 | -1.2 | 0.2 | 56 | -1.3 | 0.2 |
| σ^E^ | *CD2799A* | CD630_27991 | hypothetical protein | 58 | -2.4 | 2.2x10^-7^ | 50 | -3.3 | 3.5x10^-10^ | 50 | -4.1 | 3.9x10^-18^ | 79 | -0.9 | 0.1 | 59 | -1.0 | 0.4 |
| σ^F^ | *CD1285* | CD630_12850 | Holliday junction resolvase-like protein | 56 | -2.9 | 0.02 | 57 | -2.2 | 0.1 | 60 | -2.1 | 0.1 | 84 | -0.8 | 0.9 | 55 | -1.4 | 0.6 |
| σ^F^ | *uppS* | CD630_27620 | undecaprenyl pyrophosphate synthetase | 56 | -2.4 | 1.3x10^-6^ | 60 | -1.5 | 8.4x10^-3^ | 114 | 0.6 | 0.5 | 73 | -1.2 | 0.1 | 57 | -1.0 | 0.3 |
| σ^E^ | *CD1167* | CD630_11670 | integrase/recombinase | 52 | -3.1 | 6.3x10^-11^ | 45 | -6.2 | 2.1x10^-17^ | 48 | -4.8 | 5.7x10^-23^ | 82 | -0.6 | 0.5 | 61 | -0.7 | 0.9 |
| ^σ^E^ | *CD3652* | CD630_36520 | peptidase, M1 family | 52 | -3.0 | 5.1x10^-10^ | 45 | -4.9 | 5.6x10^-14^ | 46 | -8.5 | 1.1x10^-23^ | 80 | -0.7 | 0.5 | 60 | -0.7 | 0.8 |
| σ^E^ | *spoIIIAE* | CD630_11960 | stage III sporulation protein AE | 52 | -3.6 | 4.4x10^-13^ | 47 | -4.9 | 1.8x10^-14^ | 49 | -5.1 | 7.8x10^-22^ | 76 | -1.0 | 0.1 | 63 | -0.7 | 0.8 |
| ^σ^G^ | *rbr* | CD630_28450 | rubrerythrin | 51 | -3.8 | 2.1x10^-14^ | 48 | -4.4 | 1.6x10^-13^ | 96 | 0.0 | 1 | 56 | -3.5 | 8.4x10^-16^ | 66 | -0.5 | 1 |
| σ^F^ | *CD0125* | CD630_01250 | [cell wall endopeptidase (SpoIIQ homolog, [5])](#RANGE!_ENREF_5) | 49 | -3.6 | 3.4x10^-13^ | 46 | -4.2 | 1.3x10^-12^ | 101 | 0.3 | 1 | 72 | -1.1 | 0.05 | 58 | -0.8 | 0.6 |
| σ^E^ | *CD3551B* | CD630_35512 | hypothetical protein | 48 | -3.3 | 7.5x10^-8^ | 42 | -6.6 | 8.5x10^-13^ | 48 | -3.2 | 1.0x10^-6^ | 67 | -1.2 | 0.1 | 54 | -0.9 | 0.7 |
| σ^G^ | *CD3312* | CD630_33120 | transporter, Major Facilitator Superfamily (MFS) | 48 | -4.0 | 6.0x10^-10^ | 45 | -4.3 | 1.9x10^-9^ | 62 | -1.4 | 5.5x10^-3^ | 50 | -5.2 | 4.0x10^-12^ | 61 | -0.6 | 1 |
| ^σ^G^ | *spoVAD* | CD630_07740 | stage V sporulation protein AD | 48 | -5.1 | 1.9x10^-19^ | 46 | -4.2 | 5.7x10^-13^ | 66 | -1.2 | 7.7x10^-3^ | 52 | -4.7 | 3.3x10^-22^ | 58 | -0.9 | 0.6 |
| σ^E^ | *CD3457* | CD630_34570 | hypothetical protein | 46 | -2.3 | 2.4x10^-6^ | 40 | -3.6 | 1.0x10^-9^ | 43 | -3.1 | 6.8x10^-11^ | 66 | -0.7 | 0.5 | 46 | -1.1 | 0.3 |
| σ^E^ | *CD1740* | CD630_17400 | glycine/sarcosine/betaine reductase complex component B subunits alpha and beta | 46 | -2.7 | 3.7x10^-8^ | 41 | -3.4 | 8.8x10^-9^ | 43 | -3.5 | 5.9x10^-14^ | 69 | -0.7 | 0.5 | 44 | -1.5 | 0.05 |
| σ^K^ | *CD3350* | CD630_33500 | family 2 glycosyl transferase | 45 | -3.5 | 4.0x10^-4^ | 40 | -5.8 | 2.9x10^-6^ | 42 | -6.7 | 4.4x10^-8^ | 65 | -1.1 | 0.6 | 34 | –Inf | 1.9x10^-8^ |
| σ^G^ | *CD1430* | CD630_14300 | delta-lactam-biosynthetic de-N-acteylase | 45 | -4.7 | 1.2x10^-17^ | 43 | -4.7 | 3.4x10^-13^ | 84 | -0.1 | 1 | 49 | -4.3 | 2.2x10^-19^ | 60 | -0.5 | 0.9 |
| σ^E^ | *CD1085* | CD630_10850 | membrane protein | 45 | -2.5 | 2.7x10^-7^ | 49 | -1.5 | 0.02 | 45 | -2.4 | 1.5x10^-7^ | 61 | -1.0 | 0.1 | 49 | -0.8 | 0.5 |
| σ^E^ | *CD3440* | CD630_34400 | glycoside hydrolase-type carbohydrate-binding protein | 45 | -2.7 | 3.8x10^-8^ | 40 | -3.5 | 1.2x10^-9^ | 41 | -3.9 | 2.5x10^-15^ | 68 | -0.7 | 0.5 | 50 | -0.8 | 0.8 |
| σ^E^ | *tepA* | CD630_13230 | protein export-enhancing factor | 45 | -2.4 | 9.3x10^-3^ | 40 | -3.2 | 1.5x10^-3^ | 44 | -2.5 | 8.6x10^-3^ | 57 | -1.3 | 0.4 | 43 | -1.3 | 0.6 |
| σ^E^ | *CD1397* | CD630_13970 | hypothetical protein (VPF066 superfamily) | 44 | -2.3 | 4.0x10^-6^ | 37 | -3.8 | 5.1x10^-10^ | 40 | -2.9 | 1.8x10^-10^ | 61 | -0.8 | 0.4 | 47 | -0.7 | 0.7 |
| ^σ^G^ | *CD2635* | CD630_26350 | hypothetical protein (YIEGIA family) | 43 | -3.7 | 5.6x10^-13^ | 40 | -3.8 | 9.1x10^-11^ | 58 | -1.0 | 0.05 | 45 | -4.0 | 5.7x10^-17^ | 49 | -0.9 | 0.5 |
| σ^E^ | *ssb* | CD630_32350 | single-stranded DNA-binding protein | 41 | -3.0 | 4.7x10^-9^ | 36 | -4.8 | 3.2x10^-12^ | 37 | -5.4 | 7.3x10^-21^ | 67 | -0.5 | 0.8 | 52 | -0.4 | 1 |
| σ^E^ | *spoIV* | CD630_24420 | stage IV sporulation protein | 41 | -3.1 | 8.5x10^-10^ | 36 | -5.8 | 5.1x10^-15^ | 38 | -4.6 | 3.3x10^-18^ | 67 | -0.6 | 0.7 | 47 | -0.9 | 0.6 |
| σ^K^ | *CD1904* | CD630_19040 | ABC transporter permease | 41 | -3.4 | 7.5x10^-10^ | 36 | -5.0 | 1.6x10^-13^ | 38 | -5.8 | 5.1x10^-16^ | 72 | -0.3 | 1 | 32 | -3.9 | 2.9x10^-10^ |
| ^σ^F^ | *gpr* | CD630_24700 | germination protease | 39 | -4.4 | 3.5x10^-3^ | 36 | -5.7 | 1.6x10^-3^ | 72 | 0.0 | 1 | 51 | -1.8 | 0.5 | 43 | -1.1 | 0.8 |
| σ^E^ | *CD3248* | CD630_32480 | polysaccharide deacetylase | 39 | -3.0 | 6.3x10^-9^ | 37 | -3.0 | 8.3x10^-8^ | 35 | -4.6 | 2.2x10^-16^ | 56 | -0.9 | 0.2 | 40 | -1.2 | 0.3 |
| ^σ^G^ | *CD1707* | CD630_17070 | C4-dicarboxylate anaerobic carrier, DcuC family | 38 | -6.2 | 4.5x10^-20^ | 38 | -4.3 | 1.5x10^-11^ | 50 | -1.5 | 3.0x10^-4^ | 41 | -5.8 | 1.0x10^-23^ | 42 | -1.4 | 0.1 |
| σ^E^ | *glpQ* | CD630_14020 | glycerophosphoryl diester phosphodiesterase | 38 | -2.5 | 1.7x10^-3^ | 32 | -4.2 | 1.4x10^-6^ | 35 | -3.2 | 2.5x10^-5^ | 58 | -0.5 | 0.9 | 40 | -1.0 | 0.6 |
| ^σ^G^(σ^E^) | *CD2868* | CD630_28680 | oxidoreductase | 38 | -5.0 | 3.0x10^-17^ | 36 | -4.8 | 1.2x10^-12^ | 42 | -2.6 | 2.2x10^-8^ | 42 | -4.0 | 2.6x10^-16^ | 43 | -1.2 | 0.2 |
| ^σ^G^ | *dacF* | CD630_12910 | D-alanyl-D-alanine carboxypeptidase | 37 | -4.6 | 7.1x10^-13^ | 35 | -4.5 | 3.4x10^-11^ | 76 | 0.2 | 1 | 38 | -5.5 | 1.1x10^-14^ | 45 | -0.8 | 0.8 |
| ^σ^G^ | *spoVT* | CD630_34990 | stage V sporulation protein T | 36 | -3.6 | 3.1x10^-11^ | 33 | -5.5 | 2.5x10^-13^ | 51 | -0.9 | 0.1 | 38 | -4.5 | 4.4x10^-17^ | 41 | -1.0 | 0.4 |
| σ^F^ | *CD2266* | CD630_22660 | oxidoreductase, FAD dependent | 36 | -2.0 | 7.8x10^-3^ | 29 | -3.8 | 2.0x10^-5^ | 38 | -1.6 | 0.01 | 45 | -1.1 | 0.3 | 37 | -0.9 | 0.8 |
| σ^E^ | *CD3270* | CD630_32700 | magnesium transport ATPase, MgtC/SapB family | 36 | -2.0 | 0.04 | 43 | -0.7 | 0.4 | 34 | -2.3 | 0.01 | 65 | 0.2 | 1 | 30 | -1.8 | 0.1 |
| ^σ^G^ | *CD2809* | CD630_28090 | hypothetical protein (DUF1540) | 34 | -4.1 | 2.6x10^-11^ | 32 | -4.1 | 3.6x10^-10^ | 62 | -0.1 | 1 | 37 | -3.5 | 1.0x10^-8^ | 43 | -0.6 | 1 |
| σ^E^ | *isp* | CD630_20000 | intracellular serine protease | 34 | -3.0 | 1.5x10^-8^ | 29 | -5.2 | 3.3x10^-11^ | 30 | -5.6 | 6.5x10^-19^ | 47 | -1.1 | 0.1 | 33 | -1.4 | 0.4 |
| σ^E^ | *CD0629* | CD630_06290 | Crp family transcriptional regulator | 33 | -2.3 | 1.5x10^-5^ | 30 | -3.1 | 8.5x10^-6^ | 31 | -3.2 | 1.9x10^-9^ | 45 | -1.0 | 0.2 | 34 | -1.0 | 0.5 |
| σ^E^ | *CD3638* | CD630_36380 | hypothetical protein | 33 | -2.9 | 3.7x10^-8^ | 32 | -3.0 | 1.6x10^-5^ | 31 | -4.2 | 4.6x10^-14^ | 46 | -1.1 | 0.1 | 34 | -1.2 | 0.2 |
| σ^E^ | *pyrD* | CD630_31790 | dihydroorotate dehydrogenase, catalytic subunit | 32 | -2.5 | 7.6x10^-5^ | 28 | -3.4 | 8.1x10^-7^ | 30 | -3.1 | 7.9x10^-7^ | 49 | -0.5 | 0.9 | 41 | -0.2 | 1 |
| σ^E^ | *CD1555* | CD630_15550 | amino acid permease | 31 | -2.7 | 7.2x10^-7^ | 28 | -3.5 | 4.2x10^-7^ | 31 | -2.8 | 1.9x10^-8^ | 38 | -1.8 | 4.9x10^-4^ | 33 | -1.0 | 0.4 |
| σ^F^ | *fruABC* | CD630_22690 | PTS system fructose-specific transporter subunit IIABC | 30 | -2.1 | 1.3x10^-4^ | 28 | -2.2 | 9.0x10^-4^ | 32 | -1.6 | 2.1x10^-3^ | 34 | -1.7 | 5.0x10^-3^ | 32 | -0.7 | 0.7 |
| σ^E^ | *CD1846* | CD630_18460 | conjugative transposon protein | 28 | -2.5 | 7.7x10^-3^ | 25 | -3.1 | 1.8x10^-3^ | 27 | -2.7 | 2.0x10^-3^ | 54 | 0.1 | 1 | 25 | -2.0 | 0.1 |
| σ^G^ | *CD0543* | CD630_05430 | hypothetical protein (DUF3298) | 28 | -2.2 | 0.04 | 27 | -2.0 | 0.04 | 42 | -0.2 | 1 | 29 | -2.6 | 0.03 | 29 | -0.8 | 0.8 |
| σ^E^ | *CD3257* | CD630_32570 | polysaccharide deacetylase | 27 | -3.6 | 1.3x10^-9^ | 25 | -4.6 | 5.3x10^-10^ | 26 | -4.4 | 6.1x10^-13^ | 40 | -1.1 | 0.2 | 32 | -0.8 | 0.7 |
| σ^E^ | *CD1884* | CD630_18840 | hypothetical protein | 27 | -3.2 | 2.1x10^-8^ | 25 | -3.9 | 2.0x10^-7^ | 25 | -6.3 | 1.4x10^-13^ | 42 | -0.7 | 0.7 | 30 | -1.0 | 0.6 |
| σ^E^ | *acpS* | CD630_34660 | 4'-phosphopantetheinyl transferase | 27 | -2.3 | 7.8x10^-5^ | 26 | -2.3 | 6.1x10^-4^ | 27 | -2.2 | 2.3x10^-5^ | 40 | -0.6 | 0.8 | 31 | -0.5 | 0.9 |
| σ^E^ | *CD1741* | CD630_17410 | pseudo | 26 | -2.1 | 2.9x10^-4^ | 24 | -2.6 | 1.7x10^-4^ | 23 | -3.3 | 8.2x10^-8^ | 39 | -0.5 | 0.9 | 25 | -1.3 | 0.3 |
| σ^E^ | *mviN* | CD630_27810 | transmembrane virulence factor, MviN family protein | 26 | -2.7 | 1.5x10^-6^ | 25 | -2.6 | 2.5x10^-4^ | 25 | -3.0 | 4.2x10^-8^ | 34 | -1.3 | 0.1 | 29 | -0.8 | 0.7 |
| σ^G^ | *CD2808* | CD630_28080 | hypothetical protein | 25 | -7.4 | 2.9x10^-17^ | 25 | -4.2 | 3.5x10^-9^ | 55 | 0.3 | 1 | 30 | -2.8 | 4.4x10^-8^ | 30 | -1.0 | 0.5 |
| σ^G^ | *CD2315* | CD630_23150 | hypothetical protein (PIG-L superfamily) | 24 | -2.3 | 6.8x10^-5^ | 21 | -3.2 | 5.8x10^-5^ | 27 | -1.5 | 0.01 | 26 | -2.2 | 1.9x10^-4^ | 25 | -0.9 | 0.7 |
| ^σ^G^ | *sodA* | CD630_16310 | superoxide dismutase (Mn) | 24 | –Inf | 1.6x10^-9^ | 23 | -5.7 | 2.3x10^-6^ | 35 | -1.0 | 0.4 | 26 | –Inf | 6.1x10^-9^ | 31 | -0.7 | 0.9 |
| σ^E^ | *nrdR* | CD630_26400 | NrdR family transcriptional regulator | 23 | -3.1 | 4.7x10^-7^ | 23 | -2.8 | 2.8x10^-4^ | 21 | -5.2 | 4.4x10^-13^ | 31 | -1.3 | 0.1 | 22 | -1.7 | 0.1 |
| σ^G^ | *CD2636* | CD630_26360 | membrane protein (YIEGIA family) | 23 | -6.3 | 1.9x10^-15^ | 23 | -4.6 | 7.2x10^-9^ | 32 | -1.3 | 0.02 | 25 | -5.3 | 4.1x10^-16^ | 27 | -1.2 | 0.4 |
| ^σ^F^ | *CD1132* | CD630_11320 | heavy-metal transport/detoxification protein | 23 | -2.8 | 3.6x10^-6^ | 22 | -2.6 | 8.4x10^-5^ | 25 | -1.8 | 1.2x10^-3^ | 29 | -1.5 | 0.01 | 21 | -1.9 | 0.02 |
| σ^E^ | *CD3234* | CD630_32340 | hypothetical protein (methyltransferase domain) | 22 | -2.3 | 2.0x10^-4^ | 21 | -2.2 | 5.0x10^-3^ | 19 | -4.1 | 8.2x10^-10^ | 32 | -0.6 | 0.8 | 25 | -0.5 | 0.9 |
| σ^E^ | *CD3150A* | CD630_31501 | hypothetical protein | 21 | -3.1 | 5.2x10^-4^ | 19 | -3.4 | 1.6x10^-4^ | 19 | -4.1 | 5.7x10^-6^ | 29 | -1.1 | 0.4 | 22 | -1.0 | 0.8 |
| σ^E^ | *spoIIIAC* | CD630_11940 | stage III sporulation protein AC | 20 | -4.4 | 2.0x10^-10^ | 20 | -4.3 | 3.8x10^-7^ | 19 | -6.7 | 1.8x10^-15^ | 32 | -0.9 | 0.4 | 26 | -0.7 | 0.8 |
| σ^E^ | *CD1301* | CD630_13010 | membrane protein (TP0381 superfamily) | 20 | -3.2 | 3.3x10^-3^ | 19 | -3.7 | 2.5x10^-3^ | 19 | -4.3 | 7.7x10^-5^ | 30 | -0.9 | 0.7 | 20 | -1.5 | 0.5 |
| σ^E^ | *CD2641* | CD630_26410 | sporulation protein | 20 | -3.3 | 8.7x10^-4^ | 19 | -3.3 | 5.2x10^-3^ | 18 | -5.5 | 4.7x10^-6^ | 26 | -1.6 | 0.3 | 19 | -1.9 | 0.3 |
| σ^E^ | *CD1724* | CD630_17240 | hypothetical protein (DUF3795) | 20 | -2.9 | 3.2x10^-3^ | 18 | -4.1 | 4.2x10^-4^ | 18 | -4.0 | 4.1x10^-4^ | 28 | -1.0 | 0.8 | 20 | -1.4 | 0.6 |
| σ^E^ | *CD1844A* | CD630_18441 | pseudo | 20 | -2.5 | 4.9x10^-3^ | 16 | -6.5 | 7.4x10^-7^ | 17 | -4.4 | 3.7x10^-5^ | 35 | 0.0 | 1 | 16 | -2.4 | 0.06 |
| σ^F^ | *CD3180* | CD630_31800 | purine permease | 19 | -3.2 | 0.03 | 18 | -3.1 | 0.03 | 21 | -2.3 | 0.1 | 30 | -0.6 | 1 | 26 | -0.2 | 1 |
| σ^E^ | *CD1575* | CD630_15750 | hypothetical protein (DUF348, COG3584) | 19 | -2.0 | 3.3x10^-3^ | 16 | -3.7 | 2.4x10^-5^ | 16 | -3.8 | 4.9x10^-8^ | 30 | -0.2 | 1 | 20 | -0.8 | 0.8 |
| σ^E^ | *CD2638* | CD630_26380 | two-component response regulator | 18 | -2.0 | 3.1x10^-3^ | 17 | -2.3 | 7.4x10^-3^ | 17 | -2.2 | 1.3x10^-3^ | 22 | -1.2 | 0.2 | 15 | -1.9 | 0.1 |
| ^σ^G^ | *CD2841* | CD630_28410 | amidohydrolase | 18 | -3.4 | 7.1x10^-7^ | 17 | -3.5 | 1.1x10^-4^ | 27 | -0.7 | 0.5 | 20 | -3.1 | 1.6x10^-6^ | 18 | -1.7 | 0.2 |
| σ^E^ | *spoIIIAD* | CD630_11950 | stage III sporulation protein AD | 18 | -5.4 | 1.6x10^-11^ | 17 | -4.4 | 1.4x10^-6^ | 18 | -4.9 | 1.5x10^-11^ | 29 | -0.9 | 0.4 | 22 | -0.9 | 0.7 |
| σ^E^ | *CD3637* | CD630_36370 | NADPH-dependent FMN reductase | 16 | -3.0 | 0.02 | 17 | -1.9 | 0.1 | 15 | -3.6 | 8.6x10^-3^ | 23 | -0.9 | 0.9 | 18 | -0.8 | 0.9 |
| ^σ^G^ | *CD24310* | CD630_24310 | nitrite/sulfite reductase | 15 | -2.4 | 1.2x10^-3^ | 13 | -3.8 | 1.6x10^-4^ | 18 | -1.2 | 0.2 | 16 | -2.3 | 1.4x10^-3^ | 17 | -0.6 | 0.9 |
| σ^E^ | *CD1726* | CD630_17260 | hypothetical protein | 15 | -2.6 | 8.3x10^-4^ | 12 | -7.1 | 8.5x10^-8^ | 13 | -4.5 | 1.1x10^-5^ | 20 | -1.0 | 0.5 | 16 | -1.0 | 0.7 |
| σ^K^ | *CD0902* | CD630_09020 | cation efflux protein | 15 | -3.3 | 2.0x10^-3^ | 14 | -3.6 | 2.1x10^-3^ | 13 | -7.0 | 3.8x10^-6^ | 36 | 0.6 | 0.9 | 12 | -3.8 | 7.2x10^-3^ |
| σ^G^ | *CD1354* | CD630_13540 | hypothetical protein | 14 | -5.0 | 3.1x10^-9^ | 14 | -3.3 | 6.1x10^-4^ | 29 | 0.2 | 1 | 15 | -4.3 | 1.0x10^-8^ | 19 | -0.5 | 1 |
| σ^F^ | *CD0906* | CD630_09060 | DNA-binding protein | 13 | -3.1 | 0.03 | 17 | -1.1 | 0.5 | 14 | -2.6 | 0.1 | 19 | -1.1 | 0.9 | 21 | 0.2 | 1 |
| σ^F^ | *CD1031* | CD630_10310 | cell wall anchored protein | 13 | -2.4 | 1.9x10^-3^ | 14 | -1.8 | 0.1 | 15 | -1.6 | 0.03 | 15 | -1.9 | 0.02 | 14 | -1.0 | 0.7 |
| σ^E^ | *CD0131* | CD630_01310 | membrane protein | 13 | -3.0 | 1.2x10^-4^ | 12 | -3.4 | 1.8x10^-3^ | 12 | -5.3 | 1.1x10^-8^ | 18 | -1.1 | 0.4 | 12 | -1.8 | 0.2 |
| σ^E^ | *CD1929* | CD630_19290 | membrane protein | 13 | -3.5 | 1.1x10^-5^ | 12 | -4.4 | 5.2x10^-5^ | 13 | -3.5 | 1.1x10^-6^ | 19 | -0.9 | 0.7 | 14 | -1.1 | 0.6 |
| σ^E^ | *cwlD* | CD630_01060 | Germination-specific N-acetylmuramoyl-L-alanine amidase, Autolysin | 13 | -2.3 | 4.2x10^-3^ | 10 | -4.9 | 4.1x10^-5^ | 11 | -4.2 | 3.0x10^-6^ | 18 | -0.8 | 0.8 | 12 | -1.1 | 0.7 |
| σ^F^ | *CD2685* | CD630_26850 | sporulation stage II, protein E | 13 | -2.0 | 0.02 | 16 | -0.6 | 0.8 | 15 | -1.1 | 0.3 | 15 | -1.5 | 0.2 | 12 | -1.2 | 0.6 |
| σ^E^ | *CD1185* | CD630_11850 | diguanylate kinase signaling protein | 12 | -2.0 | 0.02 | 11 | -2.5 | 0.02 | 11 | -2.6 | 2.3x10^-3^ | 16 | -1.0 | 0.7 | 10 | -2.0 | 0.2 |
| σ^G^ | *CD3551A* | CD630_35511 | [membrane protein (DUF37) [6]](#RANGE!_ENREF_6) | 12 | -5.6 | 4.7x10^-3^ | 12 | -4.2 | 0.04 | 16 | -1.4 | 0.6 | 13 | -5.6 | 0.01 | 14 | -1.3 | 0.8 |
| ^σ^G^ | *CD1595A* | CD630_15951 | ferredoxin | 12 | -2.3 | 4.4x10^-3^ | 11 | -2.9 | 3.8x10^-3^ | 15 | -1.1 | 0.3 | 13 | -2.4 | 6.0x10^-3^ | 15 | -0.3 | 1 |
| σ^F^ | *CD1297* | CD630_12970 | hypothetical protein (DUF2953) | 11 | -5.3 | 6.6x10^-8^ | 11 | -3.4 | 9.6x10^-4^ | 19 | -0.3 | 1 | 15 | -1.7 | 0.05 | 14 | -0.6 | 0.9 |
| ^σ^G^(σ^E^) | *CD2598* | CD630_25980 | oligosaccharide deacetylase | 11 | -5.2 | 9.2x10^-8^ | 11 | -3.2 | 1.8x10^-3^ | 12 | -2.4 | 5.0x10^-4^ | 12 | -3.9 | 3.0x10^-6^ | 13 | -1.0 | 0.7 |
| σ^E^ | *CD3635* | CD630_36350 | hypothetical protein | 11 | -3.9 | 0.04 | 10 | -3.5 | 0.1 | 11 | -3.9 | 0.03 | 17 | -0.9 | 1 | 12 | -1.3 | 0.9 |
| ^σ^G^(σ^E^) | *CD0214* | CD630_02140 | hypothetical protein | 11 | -2.9 | 7.3x10^-4^ | 10 | -3.3 | 1.6x10^-3^ | 10 | -3.6 | 2.7x10^-5^ | 11 | -3.1 | 3.9x10^-4^ | 11 | -1.4 | 0.6 |
| σ^G^ | *CD1298* | CD630_12980 | [hypothetical protein (YtfJ sporulation protein [7])](#RANGE!_ENREF_8) | 11 | -6.2 | 2.1x10^-8^ | 11 | -3.0 | 3.4x10^-3^ | 22 | 0.2 | 1 | 14 | -2.2 | 8.8x10^-3^ | 13 | -1.0 | 0.8 |
| σ^F^ | *CD2377* | CD630_23770 | NUDIX family hydrolase | 10 | -2.4 | 5.6x10^-3^ | 11 | -1.8 | 0.2 | 11 | -1.9 | 0.04 | 14 | -1.0 | 0.6 | 10 | -1.2 | 0.6 |
| σ^E^ | *spmA* | CD630_35420 | spore maturation protein A | 9 | -3.6 | 1.7x10^-4^ | 8 | -3.5 | 7.6x10^-3^ | 8 | –Inf | 1.1x10^-7^ | 14 | -0.8 | 0.8 | 9 | -1.4 | 0.6 |
| ^σ^F^ | *CD2245A* | CD630_22451 | hypothetical protein (Yqz-like) | 8 | -5.9 | 1.7x10^-5^ | 8 | -6.5 | 2.2x10^-5^ | 11 | -1.5 | 0.1 | 11 | -2.3 | 0.1 | 10 | -1.3 | 0.7 |
| σ^F^ | *spoIIP* | CD630_24690 | stage II sporulation protein P | 8 | -2.2 | 0.03 | 7 | -2.4 | 0.1 | 14 | 0.1 | 1 | 11 | -1.0 | 0.7 | 9 | -0.5 | 1 |
| σ^F^ | *CD2863* | CD630_28630 | sigma-54 dependent transcriptional regulator | 7 | -2.8 | 9.3x10^-3^ | 8 | -1.5 | 0.4 | 8 | -1.7 | 0.2 | 10 | -1.1 | 0.7 | 8 | -1.1 | 0.8 |
| σ^G^ | *fruK* | CD630_22700 | fructose 1-phosphate kinase | 7 | -3.2 | 2.7x10^-3^ | 8 | -1.9 | 0.2 | 9 | -1.4 | 0.2 | 8 | -2.5 | 0.04 | 7 | -2.0 | 0.3 |
| σ^E^ | *CD3636* | CD630_36360 | membrane protein | 7 | -4.5 | 2.7x10^-3^ | 7 | -3.3 | 0.05 | 6 | -5.9 | 1.0x10^-3^ | 10 | -1.0 | 0.8 | 6 | -2.0 | 0.4 |
| σ^G^ | *CD0793* | CD630_07930 | hypothetical protein | 6 | -5.6 | 1.6x10^-4^ | 6 | -4.3 | 3.6x10^-3^ | 7 | -1.8 | 0.1 | 7 | -3.5 | 4.7x10^-3^ | 6 | -2.3 | 0.3 |
| ^σ^G^ | *CD2599* | CD630_25990 | transcriptional regulator | 5 | -4.1 | 2.7x10^-3^ | 5 | -3.4 | 0.04 | 7 | -1.7 | 0.2 | 6 | -3.0 | 0.03 | 6 | -1.0 | 0.9 |
| σ^E^ | *CD2395* | CD630_23950 | hypothetical protein | 5 | -4.2 | 5.5x10^-3^ | 6 | -1.7 | 0.5 | 5 | –Inf | 3.7x10^-4^ | 7 | -1.2 | 0.7 | 5 | -2.7 | 0.3 |
| σ^G^ | *spoVAC* | CD630_07730 | stage V sporulation protein AC | 4 | -5.2 | 8.8x10^-3^ | 4 | -4.0 | 0.05 | 6 | -1.0 | 0.7 | 5 | -5.5 | 0.02 | 4 | -1.9 | 0.6 |
| σ^E^ | *CD3636A* | CD630_36361 | hypothetical protein | 4 | -4.9 | 0.01 | 4 | -2.4 | 0.3 | 4 | -3.5 | 0.04 | 5 | -1.8 | 0.6 | 4 | -2.1 | 0.6 |
| σ^E^ | *CD2687* | CD630_26870 | hypothetical protein | 3 | –Inf | 2.8x10^-3^ | 4 | -1.6 | 0.6 | 5 | -0.7 | 1 | 5 | -1.8 | 0.5 | 5 | -0.3 | 1 |
| σ^F^ | *CD1290* | CD630_12900 | small acid-soluble spore protein SASP | 3 | –Inf | 6.5x10^-3^ | 3 | -4.1 | 0.1 | 5 | -1.0 | 0.7 | 4 | -2.5 | 0.2 | 4 | -1.3 | 0.9 |
| ^σ^E^ | *CD2634* | CD630_26340 | hypothetical protein | 2 | –Inf | 0.04 | 3 | -2.6 | 0.4 | 3 | -1.2 | 0.8 | 3 | -2.8 | 0.5 | 3 | -1.3 | 1 |

^†^ Two factors are listed in the table for genes whose expression was dependent on both σ^E^ and σ^G^ (adjusted p-value ≤ 0.05, log_2_FC ≤ -2). *Dep.* indicates the most downstream sigma factor on which gene expression depends upon. *BM* refers to base mean, the mean of the counts after they were divided by the size factors to adjust for different sequencing depths. This value is the mean for the sample relative to wild type. *log_2_FC* denotes log_2_fold-change. A negative value indicates that the gene was downregulated relative to wild type. ^ Indicates that gene product was detected in Lawley *et al*. proteomic analysis of purified spores [[8](#_ENREF_7)]. *–Inf* indicates that no transcript was detected in the mutant relative to wild type. See Text S2 for the references.
