# Supplementary material for: Global Analysis of the Sporulation Pathway of Clostridium difficile
Source: PLoS Genet. 2013 Aug 8;9(8):e1003660. doi: 10.1371/journal.pgen.1003660 (PMC3738446; doi:10.1371/journal.pgen.1003660)
Supplement: Table S6 — σE-dependent genes. † Two factors are listed in the table for genes whose expression was dependent on both σE and σG (adjusted p-value≤0.05, log2FC≤−2). Dep. indicates the most downstream sigma factor on which gene expression depends upon. BM refers to base mean, the mean of the counts after they were divided by the size factors to adjust for different sequencing depths. This value is the mean for the sample relative to wild type. log2FC denotes log2fold-change. A negative value indicates that the gene was downregulated relative to wild type. ∧ Indicates that gene product was detected in Lawley et al. proteomic analysis of purified spores [70]. −Inf indicates that no transcript was detected in the mutant relative to wild type. See Text S2 for the references. (DOCX) [file pgen.1003660.s013.docx]

**Table S6. σ^E^-dependent genes.**

|  |  |  |  | **σ^E^** | | | **Spo0A** | | | **σ^F^** | | | **σ^G^** | | | **σ^K^** | | |
| --- | --- | --- | --- | --- | --- | --- | --- | --- | --- | --- | --- | --- | --- | --- | --- | --- | --- | --- |
| **Dep.^†^** | **Name** | **locus_tag** | **description** | **BM** | **log_2_FC** | **adjP** | **BM** | **log_2_FC** | **adjP** | **BM** | **log_2_FC** | **adjP** | **BM** | **log_2_FC** | **adjP** | **BM** | **log_2_FC** | **adjP** |
| ^σ^K^ | *CD1067* | CD630_10670 | hypothetical protein | 10380 | -6.1 | 1.5x10^-50^ | 9888 | -7.6 | 1.3x10^-60^ | 11923 | -2.7 | 3.9x10^-14^ | 21003 | -0.2 | 1 | 8776 | -4.7 | 2.4x10^-32^ |
| σ^K^ | *bclA3* | CD630_33490 | exosporium glycoprotein BclA3 | 2247 | -5.7 | 1.3x10^-10^ | 2155 | -6.1 | 5.9x10^-11^ | 2467 | -3.3 | 4.9x10^-5^ | 4455 | -0.2 | 1 | 1867 | -5.5 | 2.2x10^-9^ |
| ^σ^K^ | *CD1433* | CD630_14330 | peroxiredoxin/chitinase (coat protein "CotE," [1]) | 2112 | -6.4 | 2.8x10^-74^ | 2018 | -7.7 | 1.4x10^-56^ | 2343 | -3.2 | 2.2x10^-18^ | 3959 | -0.4 | 0.8 | 1753 | -5.8 | 1.2x10^-43^ |
| σ^K^ | *CD1063C* | CD630_10633 | hypothetical protein | 1632 | -6.0 | 2.9x10^-20^ | 1552 | -7.5 | 4.2x10^-27^ | 1879 | -2.7 | 5.8x10^-7^ | 3104 | -0.4 | 0.9 | 1369 | -4.9 | 3.4x10^-15^ |
| σ^K^ | *CD1063B* | CD630_10632 | hypothetical protein | 1569 | -6.2 | 3.8x10^-21^ | 1496 | -7.5 | 4.7x10^-27^ | 1802 | -2.7 | 4.7x10^-7^ | 3044 | -0.3 | 1 | 1317 | -5.1 | 2.2x10^-15^ |
| σ^E^ | *CD0311* | CD630_03110 | hypothetical protein | 966 | -6.4 | 3.1x10^-68^ | 926 | -6.8 | 2.3x10^-46^ | 1086 | -3.0 | 5.2x10^-16^ | 1715 | -0.6 | 0.4 | 1077 | -1.4 | 4.4x10^-3^ |
| ^σ^E^ | *sipL* | CD630_35670 | cell wall hydrolase (binds SpoIVA, [2]) | 937 | -4.6 | 9.5x10^-35^ | 882 | -5.8 | 2.0x10^-36^ | 1036 | -2.9 | 8.3x10^-15^ | 1505 | -0.9 | 0.05 | 1072 | -1.2 | 0.2 |
| ^σ^K^ | *cotJB2* | CD630_24000 | spore coat peptide assembly protein CotJB2 | 833 | -6.8 | 4.6x10^-57^ | 797 | -8.5 | 2.2x10^-57^ | 953 | -2.9 | 9.6x10^-15^ | 1537 | -0.5 | 0.7 | 692 | -6.2 | 5.1x10^-44^ |
| ^σ^K^ | *cotJC2* | CD630_24010 | spore coat assembly protein CotJC2 ("CotD," [1]) | 753 | -6.4 | 5.3x10^-63^ | 720 | -7.8 | 7.4x10^-53^ | 869 | -2.7 | 1.2x10^-13^ | 1398 | -0.5 | 0.7 | 625 | -5.9 | 4.3x10^-42^ |
| ^σ^E^ | *spoIVA* | CD630_26290 | stage IV sporulation protein A | 695 | -6.5 | 1.1x10^-62^ | 665 | -7.3 | 3.6x10^-48^ | 764 | -3.4 | 3.1x10^-19^ | 1121 | -1.0 | 0.01 | 824 | -1.1 | 0.2 |
| σ^E^ | *alr2* | CD630_34630 | alanine racemase | 590 | -4.6 | 1.2x10^-42^ | 583 | -3.8 | 1.7x10^-20^ | 620 | -3.6 | 1.9x10^-21^ | 999 | -0.7 | 0.3 | 739 | -0.8 | 0.5 |
| σ^E^ | *CD3464* | CD630_34640 | hypothetical protein YdcC involved in sporulation [3] | 574 | -5.0 | 2.3x10^-42^ | 557 | -4.6 | 1.5x10^-26^ | 609 | -3.6 | 2.3x10^-21^ | 965 | -0.7 | 0.2 | 698 | -0.9 | 0.2 |
| σ^K^ | *feoB* | CD630_15170 | ferrous iron transport protein B | 549 | -3.7 | 1.5x10^-14^ | 541 | -3.3 | 1.8x10^-12^ | 613 | -2.4 | 4.1x10^-8^ | 1125 | 0.0 | 1 | 475 | -3.0 | 3.0x10^-10^ |
| σ^K^ | *CD1065* | CD630_10650 | hypothetical protein | 429 | -7.3 | 6.5x10^-33^ | 411 | -7.6 | 8.1x10^-32^ | 495 | -2.7 | 2.3x10^-10^ | 769 | -0.6 | 0.5 | 394 | -3.0 | 3.0x10^-11^ |
| σ^E^ | *CD3183* | CD630_31830 | peptidase | 397 | -2.6 | 6.2x10^-16^ | 516 | -0.8 | 0.1 | 417 | -2.3 | 1.3x10^-9^ | 536 | -1.1 | 3.0x10^-3^ | 460 | -0.7 | 0.6 |
| ^σ^K^ | *CD2399* | CD630_23990 | hypothetical protein (CotJA superfamily) | 379 | -7.6 | 3.5x10^-19^ | 364 | -8.9 | 4.2x10^-21^ | 427 | -3.1 | 4.0x10^-6^ | 702 | -0.5 | 0.9 | 314 | -7.0 | 4.0x10^-16^ |
| ^σ^K^ | *sleC* | CD630_05510 | spore cortex-lytic enzyme prx10-pro-form | 372 | -6.4 | 2.4x10^-59^ | 356 | -7.0 | 5.9x10^-42^ | 417 | -3.1 | 6.4x10^-16^ | 750 | -0.2 | 1 | 307 | -6.2 | 3.4x10^-39^ |
| σ^E^ | *dapG* | CD630_13220 | aspartate kinase I | 349 | -2.6 | 7.9x10^-17^ | 351 | -2.2 | 7.9x10^-8^ | 378 | -2.0 | 6.3x10^-8^ | 483 | -1.1 | 8.8x10^-3^ | 357 | -1.2 | 0.06 |
| σ^K^ | *CD0564* | CD630_05640 | ATP-dependent protease | 328 | -2.4 | 2.1x10^-13^ | 343 | -1.8 | 5.6x10^-6^ | 343 | -2.1 | 1.8x10^-8^ | 487 | -0.7 | 0.3 | 276 | -2.2 | 4.0x10^-8^ |
| ^σ^E^ | *CD3522* | CD630_35220 | hypothetical protein | 298 | -6.0 | 8.7x10^-11^ | 286 | -6.1 | 2.8x10^-10^ | 317 | -4.0 | 2.1x10^-6^ | 449 | -1.3 | 0.2 | 312 | -1.8 | 0.2 |
| σ^E^ | *spoIIIAA* | CD630_11920 | stage III sporulation protein AA | 271 | -6.9 | 5.9x10^-59^ | 261 | -6.7 | 7.3x10^-38^ | 287 | -4.2 | 2.9x10^-25^ | 418 | -1.2 | 1.3x10^-3^ | 326 | -1.1 | 0.1 |
| σ^K^ | *dpaA* | CD630_29680 | dipicolinate synthase subunit A | 257 | -6.0 | 3.5x10^-52^ | 249 | -5.7 | 1.9x10^-31^ | 286 | -3.1 | 5.7x10^-16^ | 506 | -0.3 | 1 | 214 | -5.4 | 1.1x10^-31^ |
| ^σ^E^ | *cspC* | CD630_22460 | subtilisin-like germination-related protease | 243 | -5.0 | 8.8x10^-41^ | 239 | -4.1 | 3.4x10^-18^ | 264 | -3.2 | 8.3x10^-17^ | 401 | -0.8 | 0.1 | 276 | -1.3 | 0.07 |
| σ^E^ | *brnQ-1* | CD630_12590 | Branched chain amino acid transport system carrier protein | 207 | -3.2 | 1.8x10^-19^ | 185 | -5.0 | 9.5x10^-25^ | 235 | -2.0 | 2.4x10^-7^ | 368 | -0.3 | 1 | 301 | -0.1 | 1 |
| ^σ^E^ | *CD3521* | CD630_35210 | peptidase T, M20B family | 193 | -2.8 | 6.7x10^-13^ | 193 | -2.4 | 9.8x10^-9^ | 216 | -1.9 | 1.5x10^-6^ | 292 | -0.7 | 0.2 | 210 | -1.0 | 0.3 |
| σ^K^ | *spoVFB* | CD630_29670 | dipicolinate synthase subunit B | 181 | -5.6 | 1.1x10^-44^ | 177 | -4.8 | 2.7x10^-24^ | 202 | -3.1 | 9.6x10^-15^ | 347 | -0.3 | 1 | 153 | -4.6 | 3.1x10^-24^ |
| ^σ^E^ | *cspBA* | CD630_22470 | subtilisin-like germination related protease | 173 | -3.7 | 4.2x10^-14^ | 173 | -3.1 | 9.5x10^-8^ | 188 | -2.7 | 6.7x10^-11^ | 270 | -0.9 | 0.1 | 197 | -1.0 | 0.2 |
| σ^E^ | *spoIIIAG* | CD630_11980 | stage III sporulation protein AG | 171 | -6.1 | 7.2x10^-48^ | 164 | -5.9 | 6.9x10^-29^ | 189 | -3.2 | 1.3x10^-15^ | 284 | -0.8 | 0.1 | 210 | -1.0 | 0.3 |
| σ^E^ | *spoIIIAH* | CD630_11990 | stage III sporulation protein AH | 170 | -5.4 | 7.9x10^-26^ | 162 | -6.2 | 3.2x10^-28^ | 187 | -3.2 | 2.1x10^-15^ | 286 | -0.8 | 0.2 | 208 | -0.9 | 0.6 |
| σ^E^ | *CD3182* | CD630_31820 | D-aminoacylase | 169 | -2.4 | 2.0x10^-4^ | 200 | -1.1 | 0.1 | 177 | -2.1 | 9.1x10^-4^ | 248 | -0.7 | 0.7 | 200 | -0.5 | 0.9 |
| σ^E^ | *spoIIID* | CD630_01260 | stage III sporulation protein D | 167 | -6.8 | 2.2x10^-24^ | 160 | -7.2 | 2.0x10^-25^ | 183 | -3.5 | 7.4x10^-13^ | 289 | -0.7 | 0.4 | 190 | -1.4 | 0.2 |
| ^σ^E^ | *CD1613* | CD630_16130 | hypothetical protein (coat protein "CotA," [1]) | 161 | -3.6 | 0.02 | 151 | -4.2 | 6.7x10^-3^ | 174 | -2.7 | 0.1 | 341 | 0.2 | 1 | 135 | -3.4 | 0.1 |
| σ^E^ | *spoIIIAB* | CD630_11930 | stage III sporulation protein AB | 158 | -7.2 | 4.5x10^-52^ | 153 | -6.4 | 6.2x10^-31^ | 168 | -4.1 | 6.2x10^-23^ | 242 | -1.3 | 1.1x10^-3^ | 199 | -0.9 | 0.3 |
| σ^E^ | *CD1068* | CD630_10680 | polysaccharide biosynthesis/sporulation protein | 154 | -4.5 | 2.2x10^-32^ | 150 | -4.1 | 2.1x10^-19^ | 180 | -2.3 | 1.2x10^-8^ | 253 | -0.8 | 0.2 | 190 | -0.8 | 0.6 |
| ^σ^E^ | *CD2864* | CD630_28640 | hydrolase | 148 | -5.6 | 4.3x10^-41^ | 143 | -5.3 | 1.9x10^-25^ | 168 | -2.8 | 1.8x10^-12^ | 267 | -0.5 | 0.6 | 183 | -0.9 | 0.3 |
| ^σ^E^ | *CD1511* | CD630_15110 | [hypothetical protein (coat protein - "CotB," [1])](#RANGE!_ENREF_1) | 146 | -4.5 | 3.5x10^-32^ | 137 | -5.5 | 1.3x10^-25^ | 163 | -2.7 | 1.3x10^-11^ | 270 | -0.3 | 1 | 200 | -0.4 | 1 |
| σ^E^ | *CD1403* | CD630_14030 | synthetase | 145 | -2.8 | 9.9x10^-15^ | 135 | -3.1 | 3.8x10^-12^ | 159 | -2.0 | 5.5x10^-7^ | 230 | -0.6 | 0.5 | 177 | -0.5 | 0.9 |
| σ^E^ | *CD2441A* | CD630_24411 | phoH-like protein | 139 | -3.5 | 9.4x10^-23^ | 136 | -3.3 | 4.4x10^-13^ | 148 | -2.8 | 7.2x10^-12^ | 225 | -0.7 | 0.3 | 161 | -0.9 | 0.4 |
| σ^E^ | *CD1380* | CD630_13800 | transporter, Major Facilitator Superfamily (MFS) | 130 | -2.4 | 1.4x10^-11^ | 114 | -3.5 | 9.7x10^-13^ | 134 | -2.2 | 2.0x10^-8^ | 184 | -0.9 | 0.1 | 114 | -1.9 | 6.1x10^-5^ |
| σ^E^ | *CD3462* | CD630_34620 | antitoxin endoAI | 128 | -4.0 | 1.1x10^-18^ | 126 | -3.4 | 1.7x10^-13^ | 136 | -3.0 | 4.2x10^-13^ | 216 | -0.6 | 0.5 | 159 | -0.7 | 0.7 |
| σ^E^ | *CD3181* | CD630_31810 | chlorohydrolase/aminohydrolase | 128 | -2.7 | 3.0x10^-8^ | 122 | -2.8 | 2.7x10^-9^ | 130 | -2.7 | 1.5x10^-8^ | 193 | -0.7 | 0.4 | 156 | -0.5 | 1 |
| σ^E^ | *CD1168* | CD630_11680 | [membrane protein (spore coat, YlbJ [4])](#RANGE!_ENREF_4) | 120 | -6.8 | 8.0x10^-46^ | 117 | -6.0 | 9.7x10^-27^ | 134 | -3.2 | 7.2x10^-15^ | 201 | -0.9 | 0.1 | 143 | -1.1 | 0.3 |
| σ^E^ | *dpaL* | CD630_31840 | diaminopropionate ammonia-lyase | 118 | -2.6 | 2.7x10^-4^ | 184 | -0.2 | 1 | 127 | -2.1 | 2.0x10^-3^ | 163 | -1.0 | 0.5 | 139 | -0.6 | 0.9 |
| ^σ^G^(σ^E^) | *sspB* | CD630_32490 | Small, acid-soluble spore protein beta | 115 | -2.8 | 6.4x10^-4^ | 97 | -8.1 | 1.8x10^-11^ | 102 | -7.0 | 1.2x10^-11^ | 109 | -7.0 | 7.3x10^-11^ | 129 | -0.8 | 1 |
| σ^E^ | *CD0129* | CD630_01290 | hypothetical protein (YyaC, in sporulating bacteria) | 109 | -5.1 | 3.0x10^-33^ | 105 | -5.1 | 8.9x10^-23^ | 126 | -2.5 | 6.7x10^-10^ | 198 | -0.5 | 0.8 | 148 | -0.5 | 0.9 |
| σ^E^ | *CD2084* | CD630_20840 | peptidase | 109 | -2.3 | 2.8x10^-10^ | 137 | -0.8 | 0.1 | 113 | -2.1 | 3.0x10^-7^ | 138 | -1.3 | 2.8x10^-3^ | 125 | -0.6 | 0.8 |
| ^σ^E^ | *CD1319* | CD630_13190 | polysaccharide deacetylase | 107 | -5.2 | 3.5x10^-34^ | 103 | -5.0 | 1.4x10^-21^ | 113 | -3.7 | 7.9x10^-18^ | 155 | -1.4 | 2.9x10^-4^ | 126 | -1.1 | 0.2 |
| σ^E^ | *CD1928* | CD630_19280 | membrane protein | 105 | -6.0 | 5.7x10^-38^ | 100 | -7.4 | 2.0x10^-28^ | 114 | -3.4 | 7.2x10^-16^ | 152 | -1.5 | 1.4x10^-4^ | 119 | -1.3 | 0.1 |
| σ^E^ | *EndoA* | CD630_34610 | endoribonuclease toxin | 104 | -4.1 | 4.8x10^-17^ | 103 | -3.5 | 3.9x10^-13^ | 112 | -3.0 | 9.6x10^-12^ | 181 | -0.5 | 0.7 | 139 | -0.5 | 0.9 |
| σ^E^ | *CD2833* | CD630_28330 | calcium-transporting ATPase | 102 | -6.1 | 9.7x10^-40^ | 100 | -4.8 | 1.7x10^-18^ | 119 | -2.7 | 1.1x10^-10^ | 184 | -0.6 | 0.6 | 124 | -1.0 | 0.4 |
| ^σ^K^ | *CD1133* | CD630_11330 | hypothetical protein | 101 | -4.5 | 1.7x10^-18^ | 96 | -4.9 | 8.8x10^-21^ | 119 | -2.2 | 7.1x10^-7^ | 255 | 0.5 | 0.6 | 86 | -3.6 | 9.0x10^-13^ |
| σ^E^ | *CD3177* | CD630_31770 | xanthine dehydrogenase | 98 | -3.5 | 3.5x10^-9^ | 90 | -4.5 | 1.2x10^-11^ | 103 | -3.0 | 4.8x10^-8^ | 153 | -0.8 | 0.3 | 125 | -0.6 | 1 |
| σ^E^ | *CD3298* | CD630_32980 | ATP/GTP-binding protein | 97 | -5.6 | 4.7x10^-15^ | 92 | -6.6 | 2.6x10^-16^ | 112 | -2.6 | 1.0x10^-5^ | 168 | -0.7 | 0.6 | 107 | -1.4 | 0.3 |
| σ^E^ | *CD0017* | CD630_00170 | DNA binding protein | 97 | -2.2 | 4.7x10^-5^ | 95 | -2.1 | 1.0x10^-4^ | 105 | -1.8 | 1.2x10^-3^ | 141 | -0.7 | 0.5 | 101 | -0.9 | 0.5 |
| ^σ^E^ | *CD3269* | CD630_32690 | oligoendopeptidase F, M3B family | 97 | -2.1 | 4.6x10^-9^ | 130 | -0.5 | 0.4 | 100 | -2.1 | 7.1x10^-7^ | 149 | -0.4 | 0.9 | 90 | -1.4 | 0.01 |
| σ^E^ | *CD1066* | CD630_10660 | hypothetical protein | 95 | -4.1 | 4.6x10^-24^ | 91 | -4.0 | 1.8x10^-15^ | 105 | -2.7 | 2.4x10^-10^ | 149 | -0.9 | 0.1 | 96 | -1.7 | 2.3x10^-3^ |
| σ^E^ | *dnaX* | CD630_00160 | DNA polymerase III subunits gamma and tau | 90 | -3.2 | 9.3x10^-7^ | 85 | -3.4 | 4.2x10^-7^ | 97 | -2.4 | 1.5x10^-4^ | 141 | -0.7 | 0.6 | 102 | -0.9 | 0.6 |
| σ^E^ | *CD2800* | CD630_28000 | membrane protein | 86 | -5.4 | 2.5x10^-32^ | 84 | -4.5 | 2.4x10^-18^ | 95 | -3.2 | 1.7x10^-13^ | 132 | -1.1 | 0.01 | 96 | -1.4 | 0.03 |
| σ^E^ | *CD1930* | CD630_19300 | hypothetical protein (ComEC-related) | 85 | -4.1 | 1.6x10^-24^ | 99 | -1.8 | 4.8x10^-5^ | 94 | -2.7 | 1.5x10^-10^ | 149 | -0.5 | 0.8 | 102 | -0.9 | 0.6 |
| ^σ^E^ | *CD0761* | CD630_07610 | ATP-dependent RNA helicase | 83 | -2.9 | 1.3x10^-14^ | 81 | -2.8 | 9.7x10^-10^ | 89 | -2.4 | 1.3x10^-8^ | 111 | -1.4 | 1.1x10^-3^ | 90 | -1.0 | 0.4 |
| σ^E^ | *CD1940* | CD630_19400 | membrane protein (DUF3866 superfamily) | 78 | -4.4 | 1.6x10^-24^ | 73 | -6.3 | 3.4x10^-23^ | 84 | -3.1 | 8.8x10^-13^ | 128 | -0.8 | 0.3 | 106 | -0.5 | 0.9 |
| σ^K^ | *CD0749* | CD630_07490 | DNA helicase, UvrD/REP type | 77 | -2.8 | 1.5x10^-13^ | 69 | -4.1 | 4.7x10^-14^ | 91 | -1.6 | 5.0x10^-4^ | 226 | 1.1 | 0.01 | 60 | -3.5 | 2.8x10^-12^ |
| ^σ^K^ | *CD1063A* | CD630_10631 | hypothetical protein | 74 | -8.3 | 3.1x10^-10^ | 71 | -7.7 | 6.8x10^-9^ | 86 | -2.7 | 3.3x10^-3^ | 167 | 0.1 | 1 | 63 | -4.8 | 2.5x10^-5^ |
| σ^E^ | *CD1398* | CD630_13980 | peptidase, M20D family | 74 | -4.1 | 2.7x10^-22^ | 69 | -4.9 | 2.4x10^-18^ | 82 | -2.6 | 1.7x10^-9^ | 119 | -0.8 | 0.2 | 83 | -1.2 | 0.2 |
| σ^K^ | *feoA* | CD630_15180 | ferrous iron transport protein | 72 | -5.2 | 3.9x10^-14^ | 70 | -4.7 | 2.0x10^-12^ | 79 | -3.2 | 9.2x10^-8^ | 149 | 0.0 | 1 | 60 | -4.7 | 3.0x10^-11^ |
| σ^E^ | *era* | CD630_24370 | GTPase Era | 72 | -2.2 | 3.2x10^-8^ | 79 | -1.4 | 5.7x10^-3^ | 75 | -1.9 | 1.1x10^-5^ | 101 | -0.8 | 0.3 | 81 | -0.6 | 0.8 |
| σ^E^ | *CD3465* | CD630_34650 | hypothetical protein (CBS domain) | 71 | -3.0 | 1.9x10^-11^ | 69 | -2.8 | 1.7x10^-8^ | 72 | -2.9 | 4.0x10^-10^ | 109 | -0.7 | 0.3 | 89 | -0.5 | 0.9 |
| ^σ^K^ | *CD0596* | CD630_05960 | hypothetical protein (CotJA homolog) | 69 | -7.5 | 7.3x10^-7^ | 67 | -7.0 | 1.2x10^-5^ | 75 | -3.7 | 4.7x10^-3^ | 159 | 0.2 | 1 | 58 | -6.1 | 1.4x10^-4^ |
| ^σ^E^ | *CD3258* | CD630_32580 | Iron hydrogenase | 69 | -5.0 | 8.4x10^-28^ | 66 | -4.9 | 3.3x10^-16^ | 71 | -4.1 | 1.3x10^-17^ | 103 | -1.2 | 8.8x10^-3^ | 71 | -1.8 | 1.5x10^-3^ |
| σ^E^ | *CD1396* | CD630_13960 | amino acid amidase | 69 | -2.0 | 7.0x10^-7^ | 66 | -2.0 | 5.1x10^-5^ | 75 | -1.5 | 1.2x10^-3^ | 104 | -0.4 | 0.9 | 75 | -0.6 | 0.8 |
| σ^K^ | *sigK* | CD630_12300 | sporulation factor σ^K^ | 68 | -7.2 | 4.8x10^-35^ | 68 | -4.6 | 4.8x10^-17^ | 80 | -2.5 | 8.1x10^-9^ | 138 | -0.2 | 1 | 61 | -3.6 | 1.7x10^-12^ |
| σ^E^ | *CD1321* | CD630_13210 | sporulation protein (YlmC) | 68 | -3.1 | 5.5x10^-13^ | 72 | -2.1 | 6.7x10^-6^ | 72 | -2.6 | 4.4x10^-9^ | 95 | -1.2 | 0.01 | 70 | -1.4 | 0.06 |
| σ^E^ | *CD3251* | CD630_32510 | dehydrogenase | 64 | -2.5 | 5.0x10^-9^ | 71 | -1.5 | 8.1x10^-3^ | 73 | -1.6 | 7.5x10^-4^ | 95 | -0.7 | 0.5 | 77 | -0.5 | 0.9 |
| ^σ^K^ | *cotJC1* | CD630_05980 | [spore coat assembly protein ("CotCB," [1])](#RANGE!_ENREF_1) | 63 | -6.7 | 2.7x10^-3^ | 63 | -4.8 | 0.03 | 69 | -3.7 | 0.1 | 151 | 0.3 | 1 | 53 | -6.2 | 0.02 |
| ^σ^K^ | *CD3580* | CD630_35800 | hypothetical protein | 62 | -5.1 | 6.6x10^-24^ | 59 | -5.6 | 1.5x10^-19^ | 68 | -3.0 | 9.2x10^-11^ | 111 | -0.5 | 0.8 | 51 | -4.7 | 2.7x10^-16^ |
| ^σ^K^ | *bclA1* | CD630_03320 | exosporium glycoprotein | 59 | -7.1 | 7.2x10^-10^ | 57 | -5.4 | 3.3x10^-6^ | 66 | -3.2 | 4.8x10^-4^ | 110 | -0.4 | 1 | 49 | -5.6 | 6.6x10^-7^ |
| σ^E^ | *CD0760* | CD630_07600 | Ca^2+^/Na^+^ antiporter | 59 | -3.9 | 4.7x10^-10^ | 58 | -3.6 | 9.9x10^-10^ | 68 | -2.2 | 8.6x10^-5^ | 90 | -1.0 | 0.1 | 64 | -1.3 | 0.1 |
| σ^K^ | *CD0896* | CD630_08960 | hypothetical protein | 57 | -4.4 | 6.4x10^-4^ | 52 | -6.8 | 5.7x10^-6^ | 59 | -3.6 | 4.5x10^-3^ | 100 | -0.5 | 1 | 46 | -5.6 | 4.8x10^-4^ |
| σ^E^ | *CD3178* | CD630_31780 | D-hydantoinase | 57 | -3.4 | 3.6x10^-11^ | 54 | -3.8 | 3.2x10^-11^ | 60 | -2.8 | 2.4x10^-9^ | 83 | -1.1 | 0.05 | 65 | -0.9 | 0.5 |
| σ^E^ | *spoIIIAF* | CD630_11970 | stage III sporulation protein AF | 56 | -6.2 | 5.6x10^-30^ | 55 | -5.0 | 5.8x10^-17^ | 61 | -3.6 | 2.4x10^-14^ | 94 | -0.8 | 0.2 | 69 | -1.0 | 0.3 |
| σ^E^ | *CD2121* | CD630_21210 | hypothetical protein | 56 | -5.5 | 2.1x10^-8^ | 54 | -5.4 | 2.9x10^-8^ | 64 | -2.7 | 5.1x10^-4^ | 96 | -0.7 | 0.8 | 62 | -1.4 | 0.4 |
| σ^E^ | *CD1320* | CD630_13200 | M16 family peptidase | 55 | -2.9 | 2.9x10^-5^ | 56 | -2.3 | 7.4x10^-4^ | 58 | -2.6 | 2.3x10^-4^ | 75 | -1.2 | 0.2 | 56 | -1.3 | 0.2 |
| σ^E^ | *CD2637* | CD630_26370 | two-component sensor histidine kinase | 55 | -2.6 | 1.5x10^-7^ | 56 | -2.2 | 5.2x10^-5^ | 59 | -2.2 | 3.2x10^-6^ | 75 | -1.1 | 0.04 | 56 | -1.2 | 0.4 |
| σ^E^ | *CD0557* | CD630_05570 | uridine kinase | 52 | -2.2 | 7.9x10^-8^ | 44 | -3.7 | 5.1x10^-10^ | 61 | -1.2 | 0.03 | 80 | -0.5 | 0.9 | 54 | -0.9 | 0.5 |
| σ^E^ | *CD2799A* | CD630_27991 | hypothetical protein | 50 | -4.1 | 3.9x10^-18^ | 50 | -3.3 | 3.5x10^-10^ | 58 | -2.4 | 2.2x10^-7^ | 79 | -0.9 | 0.1 | 59 | -1.0 | 0.4 |
| σ^E^ | *spoIIIAE* | CD630_11960 | stage III sporulation protein AE | 49 | -5.1 | 7.8x10^-22^ | 47 | -4.9 | 1.8x10^-14^ | 52 | -3.6 | 4.4x10^-13^ | 76 | -1.0 | 0.1 | 63 | -0.7 | 0.8 |
| σ^K^ | *CD2144* | CD630_21440 | putative sporulation membrane protein YtaF | 49 | -4.8 | 1.4x10^-9^ | 46 | -6.7 | 8.8x10^-11^ | 58 | -2.3 | 1.8x10^-3^ | 95 | -0.2 | 1 | 40 | -6.0 | 8.1x10^-10^ |
| σ^E^ | *CD1395* | CD630_13950 | membrane protein (YrvL superfamily) | 49 | -2.5 | 2.4x10^-8^ | 45 | -3.2 | 4.2x10^-9^ | 54 | -1.9 | 5.6x10^-5^ | 79 | -0.4 | 0.9 | 59 | -0.5 | 0.9 |
| σ^E^ | *CD1167* | CD630_11670 | integrase/recombinase | 48 | -4.8 | 5.7x10^-23^ | 45 | -6.2 | 2.1x10^-17^ | 52 | -3.1 | 6.3x10^-11^ | 82 | -0.6 | 0.5 | 61 | -0.7 | 0.9 |
| σ^E^ | *CD3551B* | CD630_35512 | hypothetical protein | 48 | -3.2 | 1.0x10^-6^ | 42 | -6.6 | 8.5x10^-13^ | 48 | -3.3 | 7.5x10^-8^ | 67 | -1.2 | 0.1 | 54 | -0.9 | 0.7 |
| ^σ^E^ | *CD3007* | CD630_30070 | hypothetical protein | 48 | -2.1 | 2.1x10^-6^ | 41 | -3.3 | 2.5x10^-8^ | 52 | -1.6 | 1.8x10^-3^ | 81 | -0.1 | 1 | 41 | -1.8 | 5.3x10^-3^ |
| ^σ^E^ | *CD3652* | CD630_36520 | peptidase, M1 family | 46 | -8.5 | 1.1x10^-23^ | 45 | -4.9 | 5.6x10^-14^ | 52 | -3.0 | 5.1x10^-10^ | 80 | -0.7 | 0.5 | 60 | -0.7 | 0.8 |
| σ^E^ | *CD1085* | CD630_10850 | membrane protein | 45 | -2.4 | 1.5x10^-7^ | 49 | -1.5 | 0.02 | 45 | -2.5 | 2.7x10^-7^ | 61 | -1.0 | 0.1 | 49 | -0.8 | 0.5 |
| σ^E^ | *CD0296* | CD630_02960 | hypothetical protein | 44 | -2.8 | 2.5x10^-5^ | 43 | -2.5 | 1.3x10^-5^ | 51 | -1.6 | 2.0x10^-3^ | 81 | -0.1 | 1 | 44 | -1.3 | 0.2 |
| σ^E^ | *tepA* | CD630_13230 | protein export-enhancing factor | 44 | -2.5 | 8.6x10^-3^ | 40 | -3.2 | 1.5x10^-3^ | 45 | -2.4 | 9.3x10^-3^ | 57 | -1.3 | 0.4 | 43 | -1.3 | 0.6 |
| σ^E^ | *CD1740* | CD630_17400 | glycine/sarcosine/betaine reductase complex component B subunits alpha and beta | 43 | -3.5 | 5.9x10^-14^ | 41 | -3.4 | 8.8x10^-9^ | 46 | -2.7 | 3.7x10^-8^ | 69 | -0.7 | 0.5 | 44 | -1.5 | 0.05 |
| σ^E^ | *CD3457* | CD630_34570 | hypothetical protein | 43 | -3.1 | 6.8x10^-11^ | 40 | -3.6 | 1.0x10^-9^ | 46 | -2.3 | 2.4x10^-6^ | 66 | -0.7 | 0.5 | 46 | -1.1 | 0.3 |
| σ^K^ | *CD3350* | CD630_33500 | family 2 glycosyl transferase | 42 | -6.7 | 4.4x10^-8^ | 40 | -5.8 | 2.9x10^-6^ | 45 | -3.5 | 4.0x10^-4^ | 65 | -1.1 | 0.6 | 34 | –Inf | 1.9x10^-8^ |
| σ^E^ | *bclA2* | CD630_32300 | exosporium glycoprotein | 42 | -5.9 | 0.01 | 42 | -4.2 | 0.1 | 48 | -2.9 | 0.2 | 92 | 0.0 | 1 | 36 | -4.8 | 0.1 |
| ^σ^G^(σ^E^) | *CD2868* | CD630_28680 | oxidoreductase | 42 | -2.6 | 2.2x10^-8^ | 36 | -4.8 | 1.2x10^-12^ | 38 | -5.0 | 3.0x10^-17^ | 42 | -4.0 | 2.6x10^-16^ | 43 | -1.2 | 0.2 |
| σ^E^ | *CD3440* | CD630_34400 | glycoside hydrolase-type carbohydrate-binding protein | 41 | -3.9 | 2.5x10^-15^ | 40 | -3.5 | 1.2x10^-9^ | 45 | -2.7 | 3.8x10^-8^ | 68 | -0.7 | 0.5 | 50 | -0.8 | 0.8 |
| σ^E^ | *CD1397* | CD630_13970 | hypothetical protein (VPF066 superfamily) | 40 | -2.9 | 1.8x10^-10^ | 37 | -3.8 | 5.1x10^-10^ | 44 | -2.3 | 4.0x10^-6^ | 61 | -0.8 | 0.4 | 47 | -0.7 | 0.7 |
| σ^K^ | *CD1904* | CD630_19040 | ABC transporter permease | 38 | -5.8 | 5.1x10^-16^ | 36 | -5.0 | 1.6x10^-13^ | 41 | -3.4 | 7.5x10^-10^ | 72 | -0.3 | 1 | 32 | -3.9 | 2.9x10^-10^ |
| σ^E^ | *spoIV* | CD630_24420 | stage IV sporulation protein | 38 | -4.6 | 3.3x10^-18^ | 36 | -5.8 | 5.1x10^-15^ | 41 | -3.1 | 8.5x10^-10^ | 67 | -0.6 | 0.7 | 47 | -0.9 | 0.6 |
| σ^E^ | *CD2639* | CD630_26390 | cytotoxic factor | 38 | -2.5 | 2.3x10^-7^ | 37 | -2.3 | 4.9x10^-4^ | 41 | -1.9 | 1.8x10^-4^ | 51 | -1.1 | 0.1 | 37 | -1.3 | 0.2 |
| σ^E^ | *ssb* | CD630_32350 | single-stranded DNA-binding protein | 37 | -5.4 | 7.3x10^-21^ | 36 | -4.8 | 3.2x10^-12^ | 41 | -3.0 | 4.7x10^-9^ | 67 | -0.5 | 0.8 | 52 | -0.4 | 1 |
| σ^E^ | *CD1086* | CD630_10860 | peptidase, M20D family | 37 | -2.6 | 4.3x10^-8^ | 42 | -1.4 | 0.03 | 41 | -1.9 | 1.4x10^-4^ | 51 | -1.1 | 0.1 | 45 | -0.5 | 0.9 |
| σ^E^ | *CD3248* | CD630_32480 | polysaccharide deacetylase | 35 | -4.6 | 2.2x10^-16^ | 37 | -3.0 | 8.3x10^-8^ | 39 | -3.0 | 6.3x10^-9^ | 56 | -0.9 | 0.2 | 40 | -1.2 | 0.3 |
| σ^E^ | *glpQ* | CD630_14020 | glycerophosphoryl diester phosphodiesterase | 35 | -3.2 | 2.5x10^-5^ | 32 | -4.2 | 1.4x10^-6^ | 38 | -2.5 | 1.7x10^-3^ | 58 | -0.5 | 0.9 | 40 | -1.0 | 0.6 |
| σ^E^ | *spoIID* | CD630_01240 | stage II sporulation protein D | 35 | -2.6 | 7.0x10^-8^ | 33 | -2.7 | 6.4x10^-6^ | 39 | -1.8 | 6.7x10^-4^ | 54 | -0.6 | 0.7 | 38 | -1.0 | 0.5 |
| ^σ^E^ | *cotJB1* | CD630_05970 | spore coat peptide assembly protein | 34 | -6.8 | 2.8x10^-3^ | 33 | -4.9 | 0.02 | 37 | -3.5 | 0.1 | 78 | 0.2 | 1 | 29 | -4.8 | 0.1 |
| σ^E^ | *CD3270* | CD630_32700 | magnesium transport ATPase, MgtC/SapB family | 34 | -2.3 | 0.01 | 43 | -0.7 | 0.4 | 36 | -2.0 | 0.04 | 65 | 0.2 | 1 | 30 | -1.8 | 0.1 |
| σ^E^ | *CD3456* | CD630_34560 | 5-formyltetrahydrofolate cyclo-ligase | 33 | -2.9 | 7.0x10^-9^ | 32 | -2.8 | 1.3x10^-5^ | 37 | -1.9 | 3.7x10^-4^ | 51 | -0.6 | 0.7 | 38 | -0.8 | 0.7 |
| σ^E^ | *CD3638* | CD630_36380 | hypothetical protein | 31 | -4.2 | 4.6x10^-14^ | 32 | -3.0 | 1.6x10^-5^ | 33 | -2.9 | 3.7x10^-8^ | 46 | -1.1 | 0.1 | 34 | -1.2 | 0.2 |
| σ^E^ | *CD0629* | CD630_06290 | Crp family transcriptional regulator | 31 | -3.2 | 1.9x10^-9^ | 30 | -3.1 | 8.5x10^-6^ | 33 | -2.3 | 1.5x10^-5^ | 45 | -1.0 | 0.2 | 34 | -1.0 | 0.5 |
| σ^E^ | *CD1555* | CD630_15550 | amino acid permease | 31 | -2.8 | 1.9x10^-8^ | 28 | -3.5 | 4.2x10^-7^ | 31 | -2.7 | 7.2x10^-7^ | 38 | -1.8 | 4.9x10^-4^ | 33 | -1.0 | 0.4 |
| σ^E^ | *isp* | CD630_20000 | intracellular serine protease | 30 | -5.6 | 6.5x10^-19^ | 29 | -5.2 | 3.3x10^-11^ | 34 | -3.0 | 1.5x10^-8^ | 47 | -1.1 | 0.1 | 33 | -1.4 | 0.4 |
| σ^E^ | *pyrD* | CD630_31790 | dihydroorotate dehydrogenase, catalytic subunit | 30 | -3.1 | 7.9x10^-7^ | 28 | -3.4 | 8.1x10^-7^ | 32 | -2.5 | 7.6x10^-5^ | 49 | -0.5 | 0.9 | 41 | -0.2 | 1 |
| σ^E^ | *CD1846* | CD630_18460 | conjugative transposon protein | 27 | -2.7 | 2.0x10^-3^ | 25 | -3.1 | 1.8x10^-3^ | 28 | -2.5 | 7.7x10^-3^ | 54 | 0.1 | 1 | 25 | -2.0 | 0.1 |
| σ^E^ | *acpS* | CD630_34660 | 4'-phosphopantetheinyl transferase | 27 | -2.2 | 2.3x10^-5^ | 26 | -2.3 | 6.1x10^-4^ | 27 | -2.3 | 7.8x10^-5^ | 40 | -0.6 | 0.8 | 31 | -0.5 | 0.9 |
| σ^E^ | *CD3257* | CD630_32570 | polysaccharide deacetylase | 26 | -4.4 | 6.1x10^-13^ | 25 | -4.6 | 5.3x10^-10^ | 27 | -3.6 | 1.3x10^-9^ | 40 | -1.1 | 0.2 | 32 | -0.8 | 0.7 |
| σ^K^ | *CD2346* | CD630_23460 | membrane protein | 26 | -2.3 | 7.6x10^-6^ | 24 | -2.9 | 1.9x10^-5^ | 29 | -1.5 | 0.01 | 53 | 0.4 | 1 | 22 | -2.1 | 0.01 |
| σ^E^ | *CD1884* | CD630_18840 | hypothetical protein | 25 | -6.3 | 1.4x10^-13^ | 25 | -3.9 | 2.0x10^-7^ | 27 | -3.2 | 2.1x10^-8^ | 42 | -0.7 | 0.7 | 30 | -1.0 | 0.6 |
| σ^E^ | *mviN* | CD630_27810 | transmembrane virulence factor, MviN family protein | 25 | -3.0 | 4.2x10^-8^ | 25 | -2.6 | 2.5x10^-4^ | 26 | -2.7 | 1.5x10^-6^ | 34 | -1.3 | 0.1 | 29 | -0.8 | 0.7 |
| σ^E^ | *CD1845* | CD630_18450 | membrane protein | 24 | -4.3 | 6.6x10^-3^ | 22 | -4.9 | 5.0x10^-3^ | 26 | -2.9 | 0.1 | 46 | -0.1 | 1 | 23 | -2.1 | 0.5 |
| σ^E^ | *CD1741* | CD630_17410 | pseudo | 23 | -3.3 | 8.2x10^-8^ | 24 | -2.6 | 1.7x10^-4^ | 26 | -2.1 | 2.9x10^-4^ | 39 | -0.5 | 0.9 | 25 | -1.3 | 0.3 |
| σ^E^ | *CD0556* | CD630_05560 | sugar isomerase / endonuclease | 23 | -2.3 | 9.9x10^-5^ | 20 | -2.9 | 1.3x10^-4^ | 27 | -1.2 | 0.1 | 33 | -0.7 | 0.8 | 25 | -0.6 | 0.9 |
| σ^E^ | *nrdR* | CD630_26400 | NrdR family transcriptional regulator | 21 | -5.2 | 4.4x10^-13^ | 23 | -2.8 | 2.8x10^-4^ | 23 | -3.1 | 4.7x10^-7^ | 31 | -1.3 | 0.1 | 22 | -1.7 | 0.1 |
| σ^E^ | *CD2440* | CD630_24400 | metal-dependent hydrolase | 21 | -2.5 | 2.5x10^-5^ | 24 | -1.4 | 0.1 | 23 | -1.8 | 5.6x10^-3^ | 33 | -0.5 | 0.9 | 23 | -0.8 | 0.8 |
| σ^E^ | *spoIIIAC* | CD630_11940 | stage III sporulation protein AC | 19 | -6.7 | 1.8x10^-15^ | 20 | -4.3 | 3.8x10^-7^ | 20 | -4.4 | 2.0x10^-10^ | 32 | -0.9 | 0.4 | 26 | -0.7 | 0.8 |
| σ^E^ | *CD1301* | CD630_13010 | membrane protein (TP0381 superfamily) | 19 | -4.3 | 7.7x10^-5^ | 19 | -3.7 | 2.5x10^-3^ | 20 | -3.2 | 3.3x10^-3^ | 30 | -0.9 | 0.7 | 20 | -1.5 | 0.5 |
| σ^E^ | *CD3234* | CD630_32340 | hypothetical protein (methyltransferase domain) | 19 | -4.1 | 8.2x10^-10^ | 21 | -2.2 | 5.0x10^-3^ | 22 | -2.3 | 2.0x10^-4^ | 32 | -0.6 | 0.8 | 25 | -0.5 | 0.9 |
| σ^E^ | *CD3150A* | CD630_31501 | hypothetical protein | 19 | -4.1 | 5.7x10^-6^ | 19 | -3.4 | 1.6x10^-4^ | 21 | -3.1 | 5.2x10^-4^ | 29 | -1.1 | 0.4 | 22 | -1.0 | 0.8 |
| σ^E^ | *CD2641* | CD630_26410 | sporulation protein | 18 | -5.5 | 4.7x10^-6^ | 19 | -3.3 | 5.2x10^-3^ | 20 | -3.3 | 8.7x10^-4^ | 26 | -1.6 | 0.3 | 19 | -1.9 | 0.3 |
| σ^E^ | *spoIIIAD* | CD630_11950 | stage III sporulation protein AD | 18 | -4.9 | 1.5x10^-11^ | 17 | -4.4 | 1.4x10^-6^ | 18 | -5.4 | 1.6x10^-11^ | 29 | -0.9 | 0.4 | 22 | -0.9 | 0.7 |
| σ^E^ | *CD1724* | CD630_17240 | hypothetical protein (DUF3795) | 18 | -4.0 | 4.1x10^-4^ | 18 | -4.1 | 4.2x10^-4^ | 20 | -2.9 | 3.2x10^-3^ | 28 | -1.0 | 0.8 | 20 | -1.4 | 0.6 |
| σ^E^ | *CD1844A* | CD630_18441 | pseudo | 17 | -4.4 | 3.7x10^-5^ | 16 | -6.5 | 7.4x10^-7^ | 20 | -2.5 | 4.9x10^-3^ | 35 | 0.0 | 1 | 16 | -2.4 | 0.06 |
| σ^E^ | *gltC* | CD630_17460 | Sodium/glutamate symporter | 17 | -2.6 | 1.3x10^-4^ | 18 | -1.8 | 0.05 | 20 | -1.8 | 0.01 | 27 | -0.5 | 0.9 | 25 | 0.0 | 1 |
| σ^E^ | *CD2638* | CD630_26380 | two-component response regulator | 17 | -2.2 | 1.3x10^-3^ | 17 | -2.3 | 7.4x10^-3^ | 18 | -2.0 | 3.1x10^-3^ | 22 | -1.2 | 0.2 | 15 | -1.9 | 0.1 |
| ^σ^E^ | *CD24390* | CD630_24390 | diacylglycerol kinase | 17 | -2.1 | 2.2x10^-3^ | 19 | -1.4 | 0.2 | 19 | -1.6 | 0.03 | 29 | -0.2 | 1 | 19 | -0.7 | 0.9 |
| σ^E^ | *CD1575* | CD630_15750 | hypothetical protein (DUF348, COG3584) | 16 | -3.8 | 4.9x10^-8^ | 16 | -3.7 | 2.4x10^-5^ | 19 | -2.0 | 3.3x10^-3^ | 30 | -0.2 | 1 | 20 | -0.8 | 0.8 |
| σ^E^ | *CD1124A* | CD630_11241 | hypothetical protein | 16 | -2.0 | 3.0x10^-3^ | 17 | -1.5 | 0.1 | 17 | -1.9 | 0.01 | 22 | -0.9 | 0.6 | 18 | -0.6 | 0.9 |
| σ^E^ | *CD3637* | CD630_36370 | NADPH-dependent FMN reductase | 15 | -3.6 | 8.6x10^-3^ | 17 | -1.9 | 0.1 | 16 | -3.0 | 0.02 | 23 | -0.9 | 0.9 | 18 | -0.8 | 0.9 |
| σ^E^ | *CD2316* | CD630_23160 | two-component response regulator | 15 | -2.2 | 3.2x10^-3^ | 13 | -3.1 | 1.8x10^-3^ | 16 | -1.7 | 0.03 | 20 | -0.9 | 0.6 | 15 | -0.8 | 0.8 |
| σ^E^ | *CD2427* | CD630_24270 | flavodoxin/ferredoxin oxidoreductase subunit gamma | 14 | -2.0 | 0.03 | 14 | -1.8 | 0.05 | 16 | -1.3 | 0.3 | 19 | -0.7 | 0.9 | 16 | -0.4 | 1 |
| σ^K^ | *CD0902* | CD630_09020 | cation efflux protein | 13 | -7.0 | 3.8x10^-6^ | 14 | -3.6 | 2.1x10^-3^ | 15 | -3.3 | 2.0x10^-3^ | 36 | 0.6 | 0.9 | 12 | -3.8 | 7.2x10^-3^ |
| σ^E^ | *CD1726* | CD630_17260 | hypothetical protein | 13 | -4.5 | 1.1x10^-5^ | 12 | -7.1 | 8.5x10^-8^ | 15 | -2.6 | 8.3x10^-4^ | 20 | -1.0 | 0.5 | 16 | -1.0 | 0.7 |
| σ^E^ | *CD1929* | CD630_19290 | membrane protein | 13 | -3.5 | 1.1x10^-6^ | 12 | -4.4 | 5.2x10^-5^ | 13 | -3.5 | 1.1x10^-5^ | 19 | -0.9 | 0.7 | 14 | -1.1 | 0.6 |
| σ^E^ | *CD2445* | CD630_24450 | transmembrane signaling protein,TspO/MBR family | 13 | -2.6 | 6.4x10^-4^ | 13 | -2.2 | 0.03 | 16 | -1.1 | 0.4 | 24 | 0.0 | 1 | 16 | -0.3 | 1 |
| σ^E^ | *CD0131* | CD630_01310 | membrane protein | 12 | -5.3 | 1.1x10^-8^ | 12 | -3.4 | 1.8x10^-3^ | 13 | -3.0 | 1.2x10^-4^ | 18 | -1.1 | 0.4 | 12 | -1.8 | 0.2 |
| ^σ^G^(σ^E^) | *CD2598* | CD630_25980 | oligosaccharide deacetylase | 12 | -2.4 | 5.0x10^-4^ | 11 | -3.2 | 1.8x10^-3^ | 11 | -5.2 | 9.2x10^-8^ | 12 | -3.9 | 3.0x10^-6^ | 13 | -1.0 | 0.7 |
| σ^E^ | *cwlD* | CD630_01060 | Germination-specific N-acetylmuramoyl-L-alanine amidase, Autolysin | 11 | -4.2 | 3.0x10^-6^ | 10 | -4.9 | 4.1x10^-5^ | 13 | -2.3 | 4.2x10^-3^ | 18 | -0.8 | 0.8 | 12 | -1.1 | 0.7 |
| σ^E^ | *CD3635* | CD630_36350 | hypothetical protein | 11 | -3.9 | 0.03 | 10 | -3.5 | 0.1 | 11 | -3.9 | 0.04 | 17 | -0.9 | 1 | 12 | -1.3 | 0.9 |
| σ^E^ | *CD1185* | CD630_11850 | diguanylate kinase signaling protein | 11 | -2.6 | 2.3x10^-3^ | 11 | -2.5 | 0.02 | 12 | -2.0 | 0.02 | 16 | -1.0 | 0.7 | 10 | -2.0 | 0.2 |
| ^σ^G^(σ^E^) | *CD0214* | CD630_02140 | hypothetical protein | 10 | -3.6 | 2.7x10^-5^ | 10 | -3.3 | 1.6x10^-3^ | 11 | -2.9 | 7.3x10^-4^ | 11 | -3.1 | 3.9x10^-4^ | 11 | -1.4 | 0.6 |
| ^σ^E^ | *CD2865* | CD630_28650 | bacterioferritin | 10 | -2.7 | 0.02 | 9 | -2.9 | 0.03 | 11 | -1.6 | 0.3 | 16 | -0.3 | 1 | 12 | -0.5 | 1 |
| σ^E^ | *ddl* | CD630_14080 | D-Ala-D-Ala ligase | 10 | -2.0 | 0.03 | 9 | -2.3 | 0.04 | 11 | -1.5 | 0.2 | 16 | -0.2 | 1 | 9 | -1.4 | 0.6 |
| σ^E^ | *CD3368* | CD630_33680 | ribosome biogenesis GTPase RsgA; putative EngC-like GTPase | 8 | -3.2 | 0.02 | 7 | -2.9 | 0.05 | 8 | -2.2 | 0.1 | 15 | 0.0 | 1 | 6 | -2.7 | 0.1 |
| σ^E^ | *CD2055* | CD630_20550 | hypothetical protein | 8 | -3.1 | 0.01 | 7 | -3.7 | 0.02 | 10 | -1.2 | 0.5 | 28 | 1.4 | 0.2 | 6 | -2.7 | 0.1 |
| σ^E^ | *spmA* | CD630_35420 | spore maturation protein A | 8 | –Inf | 1.1x10^-7^ | 8 | -3.5 | 7.6x10^-3^ | 9 | -3.6 | 1.7x10^-4^ | 14 | -0.8 | 0.8 | 9 | -1.4 | 0.6 |
| σ^E^ | *CD1063* | CD630_10630 | hypothetical protein | 8 | –Inf | 5.0x10^-4^ | 8 | -4.5 | 0.01 | 10 | -2.0 | 0.2 | 17 | -0.1 | 1 | 9 | -1.8 | 0.7 |
| σ^E^ | *CD3636* | CD630_36360 | membrane protein | 6 | -5.9 | 1.0x10^-3^ | 7 | -3.3 | 0.05 | 7 | -4.5 | 2.7x10^-3^ | 10 | -1.0 | 0.8 | 6 | -2.0 | 0.4 |
| σ^E^ | *CD1189* | CD630_11890 | amino acid/polyamine transporter I | 5 | -3.7 | 0.03 | 6 | -1.2 | 0.7 | 5 | -2.5 | 0.2 | 6 | -1.9 | 0.6 | 5 | -1.8 | 0.6 |
| σ^E^ | *CD0213* | CD630_02130 | spore coat protein | 5 | -3.6 | 0.04 | 5 | -3.4 | 0.05 | 6 | -2.1 | 0.3 | 6 | -1.9 | 0.5 | 6 | -1.1 | 0.9 |
| σ^E^ | *CD2395* | CD630_23950 | hypothetical protein | 5 | –Inf | 3.7x10^-4^ | 6 | -1.7 | 0.5 | 5 | -4.2 | 5.5x10^-3^ | 7 | -1.2 | 0.7 | 5 | -2.7 | 0.3 |
| σ^E^ | *CD3151* | CD630_31510 | hypothetical protein | 4 | -5.3 | 4.7x10^-3^ | 5 | -1.6 | 0.7 | 5 | -2.6 | 0.1 | 6 | -1.5 | 0.7 | 5 | -1.3 | 0.8 |
| σ^E^ | *fliI* | CD630_02510 | ATP synthase subunit beta FliI | 4 | -4.3 | 0.01 | 7 | -0.3 | 1 | 6 | -1.4 | 0.6 | 6 | -1.7 | 0.5 | 5 | -1.1 | 0.9 |
| σ^E^ | *CD3636A* | CD630_36361 | hypothetical protein | 4 | -3.5 | 0.04 | 4 | -2.4 | 0.3 | 4 | -4.9 | 0.01 | 5 | -1.8 | 0.6 | 4 | -2.1 | 0.6 |
| σ^E^ | *CD1926* | CD630_19260 | pseudo | 3 | –Inf | 0.02 | 3 | -4.1 | 0.1 | 3 | -4.4 | 0.1 | 5 | -1.2 | 0.9 | 3 | -1.6 | 0.8 |

^†^ Two factors are listed in the table for genes whose expression was dependent on both σ^E^ and σ^G^ (adjusted p-value ≤ 0.05, log_2_FC ≤ -2). *Dep.* indicates the most downstream sigma factor on which gene expression depends upon. *BM* refers to base mean, the mean of the counts after they were divided by the size factors to adjust for different sequencing depths. This value is the mean for the sample relative to wild type. *log_2_FC* denotes log_2_fold-change. A negative value indicates that the gene was downregulated relative to wild type. ^ Indicates that gene product was detected in Lawley *et al*. proteomic analysis of purified spores [[8](#_ENREF_7)]. *–Inf* indicates that no transcript was detected in the mutant relative to wild type. See Text S2 for the references.
