# Supplementary material for: Global Analysis of the Sporulation Pathway of Clostridium difficile
Source: PLoS Genet. 2013 Aug 8;9(8):e1003660. doi: 10.1371/journal.pgen.1003660 (PMC3738446; doi:10.1371/journal.pgen.1003660)
Supplement: Table S7 — σG-dependent genes. † Two factors are listed in the table for genes whose expression was dependent on both σE and σG (adjusted p-value≤0.05, log2FC≤−2). Dep. indicates the most downstream sigma factor on which gene expression depends upon. BM refers to base mean, the mean of the counts after they were divided by the size factors to adjust for different sequencing depths. This value is the mean for the sample relative to wild type. log2FC denotes log2fold-change. A negative value indicates that the gene was downregulated relative to wild type. ∧ Indicates that gene product was detected in Lawley et al. proteomic analysis of purified spores [70]. −Inf indicates that no transcript was detected in the mutant relative to wild type. See Text S2 for the references. (DOCX) [file pgen.1003660.s014.docx]

**Table S7. σ^G^-dependent genes.**

|  |  |  |  | **σ^G^** | | | **Spo0A** | | | **σ^F^** | | | | **σ^E^** | | | | **σ^K^** | | | |
| --- | --- | --- | --- | --- | --- | --- | --- | --- | --- | --- | --- | --- | --- | --- | --- | --- | --- | --- | --- | --- | --- |
| **Dep.^†^** | **Name** | **locus_tag** | **description** | **BM** | **log_2_FC** | **adjP** | **BM** | **log_2_FC** | **adjP** | **BM** | **log_2_FC** | **adjP** | **BM** | | **log_2_FC** | **adjP** | **BM** | | **log_2_FC** | **adjP** |  |
| ^σ^G^ | *sspA* | CD630_26880 | Small, acid-soluble spore protein alpha | 466 | -5.5 | 1.1x10^-24^ | 412 | -5.8 | 2.2x10^-25^ | 436 | -5.4 | 1.5x10^-24^ | 546 | | -1.8 | 5.0x10^-5^ | 557 | | -0.7 | 0.8 |  |
| ^σ^G^ | *CD2112* | CD630_21120 | hypothetical protein | 414 | -4.9 | 1.4x10^-35^ | 368 | -4.9 | 3.2x10^-28^ | 391 | -4.5 | 1.6x10^-28^ | 633 | | -0.5 | 0.4 | 411 | | -1.5 | 5.2x10^-3^ |  |
| ^σ^G^(σ^E^) | *sspB* | CD630_32490 | Small, acid-soluble spore protein beta | 109 | -7.0 | 7.3x10^-11^ | 97 | -8.1 | 1.8x10^-11^ | 102 | -7.0 | 1.2x10^-11^ | 115 | | -2.8 | 6.4x10^-4^ | 129 | | -0.8 | 1 |  |
| ^σ^G^ | *CD2687A* | CD630_26871 | hypothetical protein | 98 | -2.1 | 7.0x10^-8^ | 73 | -4.9 | 3.0x10^-18^ | 80 | -3.6 | 1.0x10^-15^ | 118 | | -0.7 | 0.1 | 92 | | -0.9 | 0.4 |  |
| ^σ^G^ | *CD1486* | CD630_14860 | ribosome recycling factor | 80 | -5.5 | 1.3x10^-27^ | 71 | -5.8 | 4.2x10^-21^ | 75 | -5.0 | 6.4x10^-23^ | 125 | | -0.4 | 0.7 | 86 | | -1.1 | 0.3 |  |
| ^σ^G^ | *CD0684* | CD630_06840 | ATP-dependent peptidase, M41 family | 65 | -3.9 | 6.9x10^-20^ | 58 | -3.7 | 6.9x10^-12^ | 60 | -4.3 | 7.9x10^-18^ | 115 | | 0.1 | 1 | 67 | | -1.2 | 0.2 |  |
| ^σ^G^ | *rbr* | CD630_28450 | rubrerythrin | 56 | -3.5 | 8.4x10^-16^ | 48 | -4.4 | 1.6x10^-13^ | 51 | -3.8 | 2.1x10^-14^ | 96 | | 0.0 | 1 | 66 | | -0.5 | 1 |  |
| ^σ^G^ | *spoVAD* | CD630_07740 | stage V sporulation protein AD | 52 | -4.7 | 3.3x10^-22^ | 46 | -4.2 | 5.7x10^-13^ | 48 | -5.1 | 1.9x10^-19^ | 66 | | -1.2 | 7.7x10^-3^ | 58 | | -0.9 | 0.6 |  |
| σ^G^ | *CD3312* | CD630_33120 | transporter, Major Facilitator Superfamily (MFS) | 50 | -5.2 | 4.0x10^-12^ | 45 | -4.3 | 1.9x10^-9^ | 48 | -4.0 | 6.0x10^-10^ | 62 | | -1.4 | 5.5x10^-3^ | 61 | | -0.6 | 1 |  |
| σ^G^ | *CD1430* | CD630_14300 | delta-lactam-biosynthetic de-N-acteylase | 49 | -4.3 | 2.2x10^-19^ | 43 | -4.7 | 3.4x10^-13^ | 45 | -4.7 | 1.2x10^-17^ | 84 | | -0.1 | 1 | 60 | | -0.5 | 0.9 |  |
| ^σ^G^ | *CD2635* | CD630_26350 | hypothetical protein (YIEGIA family) | 45 | -4.0 | 5.7x10^-17^ | 40 | -3.8 | 9.1x10^-11^ | 43 | -3.7 | 5.6x10^-13^ | 58 | | -1.0 | 0.05 | 49 | | -0.9 | 0.5 |  |
| ^σ^G^(σ^E^) | *CD2868* | CD630_28680 | oxidoreductase | 42 | -4.0 | 2.6x10^-16^ | 36 | -4.8 | 1.2x10^-12^ | 38 | -5.0 | 3.0x10^-17^ | 42 | | -2.6 | 2.2x10^-8^ | 43 | | -1.2 | 0.2 |  |
| ^σ^G^ | *CD1707* | CD630_17070 | C4-dicarboxylate anaerobic carrier, DcuC family | 41 | -5.8 | 1.0x10^-23^ | 38 | -4.3 | 1.5x10^-11^ | 38 | -6.2 | 4.5x10^-20^ | 50 | | -1.5 | 3.0x10^-4^ | 42 | | -1.4 | 0.1 |  |
| ^σ^G^ | *dacF* | CD630_12910 | D-alanyl-D-alanine carboxypeptidase | 38 | -5.5 | 1.1x10^-14^ | 35 | -4.5 | 3.4x10^-11^ | 37 | -4.6 | 7.1x10^-13^ | 76 | | 0.2 | 1 | 45 | | -0.8 | 0.8 |  |
| ^σ^G^ | *spoVT* | CD630_34990 | stage V sporulation protein T | 38 | -4.5 | 4.4x10^-17^ | 33 | -5.5 | 2.5x10^-13^ | 36 | -3.6 | 3.1x10^-11^ | 51 | | -0.9 | 0.1 | 41 | | -1.0 | 0.4 |  |
| ^σ^G^ | *CD2809* | CD630_28090 | hypothetical protein (DUF1540) | 37 | -3.5 | 1.0x10^-8^ | 32 | -4.1 | 3.6x10^-10^ | 34 | -4.1 | 2.6x10^-11^ | 62 | | -0.1 | 1 | 43 | | -0.6 | 1 |  |
| σ^G^ | *CD2808* | CD630_28080 | hypothetical protein | 30 | -2.8 | 4.4x10^-8^ | 25 | -4.2 | 3.5x10^-9^ | 25 | -7.4 | 2.9x10^-17^ | 55 | | 0.3 | 1 | 30 | | -1.0 | 0.5 |  |
| σ^G^ | *CD0543* | CD630_05430 | hypothetical protein (DUF3298) | 29 | -2.6 | 0.03 | 27 | -2.0 | 0.04 | 28 | -2.2 | 0.04 | 42 | | -0.2 | 1 | 29 | | -0.8 | 0.8 |  |
| σ^G^ | *CD2315* | CD630_23150 | hypothetical protein (PIG-L superfamily) | 26 | -2.2 | 1.9x10^-4^ | 21 | -3.2 | 5.8x10^-5^ | 24 | -2.3 | 6.8x10^-5^ | 27 | | -1.5 | 0.01 | 25 | | -0.9 | 0.7 |  |
| ^σ^G^ | *sodA* | CD630_16310 | superoxide dismutase (Mn) | 26 | –Inf | 6.1x10^-9^ | 23 | -5.7 | 2.3x10^-6^ | 24 | –Inf | 1.6x10^-9^ | 35 | | -1.0 | 0.4 | 31 | | -0.7 | 0.9 |  |
| σ^G^ | *CD2636* | CD630_26360 | membrane protein (YIEGIA family) | 25 | -5.3 | 4.1x10^-16^ | 23 | -4.6 | 7.2x10^-9^ | 23 | -6.3 | 1.9x10^-15^ | 32 | | -1.3 | 0.02 | 27 | | -1.2 | 0.4 |  |
| ^σ^G^ | *CD2841* | CD630_28410 | amidohydrolase | 20 | -3.1 | 1.6x10^-6^ | 17 | -3.5 | 1.1x10^-4^ | 18 | -3.4 | 7.1x10^-7^ | 27 | | -0.7 | 0.5 | 18 | | -1.7 | 0.2 |  |
| ^σ^G^ | *CD24310* | CD630_24310 | nitrite/sulfite reductase | 16 | -2.3 | 1.4x10^-3^ | 13 | -3.8 | 1.6x10^-4^ | 15 | -2.4 | 1.2x10^-3^ | 18 | | -1.2 | 0.2 | 17 | | -0.6 | 0.9 |  |
| σ^G^ | *CD1354* | CD630_13540 | hypothetical protein | 15 | -4.3 | 1.0x10^-8^ | 14 | -3.3 | 6.1x10^-4^ | 14 | -5.0 | 3.1x10^-9^ | 29 | | 0.2 | 1 | 19 | | -0.5 | 1 |  |
| σ^G^ | *CD1028* | CD630_10280 | signaling protein | 14 | -2.6 | 1.5x10^-3^ | 12 | -2.8 | 3.6x10^-3^ | 17 | -1.1 | 0.3 | 19 | | -0.6 | 0.9 | 23 | | 0.5 | 0.9 |  |
| σ^G^ | *CD1298* | CD630_12980 | [hypothetical protein (YtfJ sporulation protein [7])](#RANGE!_ENREF_8) | 14 | -2.2 | 8.8x10^-3^ | 11 | -3.0 | 3.4x10^-3^ | 11 | -6.2 | 2.1x10^-8^ | 22 | | 0.2 | 1 | 13 | | -1.0 | 0.8 |  |
| σ^G^ | *CD3551A* | CD630_35511 | [membrane protein (DUF37) [6]](#RANGE!_ENREF_6) | 13 | -5.6 | 0.01 | 12 | -4.2 | 0.04 | 12 | -5.6 | 4.7x10^-3^ | 16 | | -1.4 | 0.6 | 14 | | -1.3 | 0.8 |  |
| ^σ^G^ | *CD1595A* | CD630_15951 | ferredoxin | 13 | -2.4 | 6.0x10^-3^ | 11 | -2.9 | 3.8x10^-3^ | 12 | -2.3 | 4.4x10^-3^ | 15 | | -1.1 | 0.3 | 15 | | -0.3 | 1 |  |
| ^σ^G^(σ^E^) | *CD2598* | CD630_25980 | oligosaccharide deacetylase | 12 | -3.9 | 3.0x10^-6^ | 11 | -3.2 | 1.8x10^-3^ | 11 | -5.2 | 9.2x10^-8^ | 12 | | -2.4 | 5.0x10^-4^ | 13 | | -1.0 | 0.7 |  |
| ^σ^G^(σ^E^) | *CD0214* | CD630_02140 | hypothetical protein | 11 | -3.1 | 3.9x10^-4^ | 10 | -3.3 | 1.6x10^-3^ | 11 | -2.9 | 7.3x10^-4^ | 10 | | -3.6 | 2.7x10^-5^ | 11 | | -1.4 | 0.6 |  |
| σ^G^ | *fruK* | CD630_22700 | fructose 1-phosphate kinase | 8 | -2.5 | 0.04 | 8 | -1.9 | 0.2 | 7 | -3.2 | 2.7x10^-3^ | 9 | | -1.4 | 0.2 | 7 | | -2.0 | 0.3 |  |
| σ^G^ | *CD0793* | CD630_07930 | hypothetical protein | 7 | -3.5 | 4.7x10^-3^ | 6 | -4.3 | 3.6x10^-3^ | 6 | -5.6 | 1.6x10^-4^ | 7 | | -1.8 | 0.1 | 6 | | -2.3 | 0.3 |  |
| ^σ^G^ | *CD2599* | CD630_25990 | transcriptional regulator | 6 | -3.0 | 0.03 | 5 | -3.4 | 0.04 | 5 | -4.1 | 2.7x10^-3^ | 7 | | -1.7 | 0.2 | 6 | | -1.0 | 0.9 |  |
| σ^G^ | *spoVAC* | CD630_07730 | stage V sporulation protein AC | 5 | -5.5 | 0.02 | 4 | -4.0 | 0.05 | 4 | -5.2 | 8.8x10^-3^ | 6 | | -1.0 | 0.7 | 4 | | -1.9 | 0.6 |  |

^†^ Two factors are listed in the table for genes whose expression was dependent on both σ^E^ and σ^G^ (adjusted p-value ≤ 0.05, log_2_FC ≤ -2). *Dep.* indicates the most downstream sigma factor on which gene expression depends upon. *BM* refers to base mean, the mean of the counts after they were divided by the size factors to adjust for different sequencing depths. This value is the mean for the sample relative to wild type. *log_2_FC* denotes log_2_fold-change. A negative value indicates that the gene was downregulated relative to wild type. ^ Indicates that gene product was detected in Lawley *et al*. proteomic analysis of purified spores [[8](#_ENREF_7)]. *–Inf* indicates that no transcript was detected in the mutant relative to wild type. See Text S2 for the references.
