# Supplementary material for: Global Analysis of the Sporulation Pathway of Clostridium difficile
Source: PLoS Genet. 2013 Aug 8;9(8):e1003660. doi: 10.1371/journal.pgen.1003660 (PMC3738446; doi:10.1371/journal.pgen.1003660)
Supplement: Table S8 — σK-dependent genes. Dep. indicates the most downstream sigma factor on which gene expression depends upon. BM refers to base mean, the mean of the counts after they were divided by the size factors to adjust for different sequencing depths. This value is the mean for the sample relative to wild type. log2FC denotes log2fold-change. A negative value indicates that the gene was downregulated relative to wild type. ∧ Indicates that gene product was detected in Lawley et al. proteomic analysis of purified spores [70]. −Inf indicates that no transcript was detected in the mutant relative to wild type. See Text S2 for the references. (DOCX) [file pgen.1003660.s015.docx]

**Table S8. σ^K^-dependent genes.**

|  |  |  |  | **σ^K^** | | | **Spo0A** | | | **σ^F^** | | | **σ^E^** | | | **σ^G^** | | |
| --- | --- | --- | --- | --- | --- | --- | --- | --- | --- | --- | --- | --- | --- | --- | --- | --- | --- | --- |
| **Dep.** | **Name** | **locus_tag** | **description** | **BM** | **log_2_FC** | **adjP** | **BM** | **log_2_FC** | **adjP** | **BM** | **log_2_FC** | **adjP** | **BM** | **log_2_FC** | **adjP** | **BM** | **log_2_FC** | **adjP** |
| ^σ^K^ | *CD1067* | CD630_10670 | hypothetical protein | 8776 | -4.7 | 2.4x10^-32^ | 9888 | -7.6 | 1.3x10^-60^ | 11923 | -2.7 | 3.9x10^-14^ | 10380 | -6.1 | 1.5x10-^50^ | 21003 | -0.2 | 1 |
| σ^K^ | *bclA3* | CD630_33490 | exosporium glycoprotein BclA3 | 1867 | -5.5 | 2.2x10^-9^ | 2155 | -6.1 | 5.9x10^-11^ | 2467 | -3.3 | 4.9x10^-5^ | 2247 | -5.7 | 1.3x10^-10^ | 4455 | -0.2 | 1 |
| ^σ^K^ | *CD1433* | CD630_14330 | peroxiredoxin/chitinase (coat protein "CotE," [1]) | 1753 | -5.8 | 1.2x10^-43^ | 2018 | -7.7 | 1.4x10^-56^ | 2343 | -3.2 | 2.2x10^-18^ | 2112 | -6.4 | 2.8x10^-7^4 | 3959 | -0.4 | 0.8 |
| σ^K^ | *CD1063C* | CD630_10633 | hypothetical protein | 1369 | -4.9 | 3.4x10^-15^ | 1552 | -7.5 | 4.2x10^-27^ | 1879 | -2.7 | 5.8x10^-7^ | 1632 | -6.0 | 2.9x10^-20^ | 3104 | -0.4 | 0.9 |
| σ^K^ | *CD1063B* | CD630_10632 | hypothetical protein | 1317 | -5.1 | 2.2x10^-15^ | 1496 | -7.5 | 4.7x10^-27^ | 1802 | -2.7 | 4.7x10^-7^ | 1569 | -6.2 | 3.8x10^-21^ | 3044 | -0.3 | 1 |
| ^σ^K^ | *cotJB2* | CD630_24000 | spore coat peptide assembly protein CotJB2 | 692 | -6.2 | 5.1x10^-44^ | 797 | -8.5 | 2.2x10^-57^ | 953 | -2.9 | 9.6x10^-15^ | 833 | -6.8 | 4.6x10^-57^ | 1537 | -0.5 | 0.7 |
| ^σ^K^ | *cotJC2* | CD630_24010 | spore coat assembly protein CotJC2 ("CotD," [1]) | 625 | -5.9 | 4.3x10^-42^ | 720 | -7.8 | 7.4x10^-53^ | 869 | -2.7 | 1.2x10^-13^ | 753 | -6.4 | 5.3x10^-63^ | 1398 | -0.5 | 0.7 |
| σ^K^ | *feoB* | CD630_15170 | ferrous iron transport protein B | 475 | -3.0 | 3.0x10^-10^ | 541 | -3.3 | 1.8x10^-12^ | 613 | -2.4 | 4.1x10^-8^ | 549 | -3.7 | 1.5x10^-14^ | 1125 | 0.0 | 1 |
| σ^K^ | *CD1065* | CD630_10650 | hypothetical protein | 394 | -3.0 | 3.0x10^-11^ | 411 | -7.6 | 8.1x10^-32^ | 495 | -2.7 | 2.3x10^-10^ | 429 | -7.3 | 6.5x10^-33^ | 769 | -0.6 | 0.5 |
| ^σ^K^ | *CD2399* | CD630_23990 | hypothetical protein (CotJA superfamily) | 314 | -7.0 | 4.0x10^-16^ | 364 | -8.9 | 4.2x10^-21^ | 427 | -3.1 | 4.0x10^-6^ | 379 | -7.6 | 3.5x10^-19^ | 702 | -0.5 | 0.9 |
| ^σ^K^ | *sleC* | CD630_05510 | spore cortex-lytic enzyme pre-pro-form | 307 | -6.2 | 3.4x10^-39^ | 356 | -7.0 | 5.9x10^-42^ | 417 | -3.1 | 6.4x10^-16^ | 372 | -6.4 | 2.4x10^-59^ | 750 | -0.2 | 1 |
| σ^K^ | *CD0564* | CD630_05640 | ATP-dependent protease | 276 | -2.2 | 4.0x10^-8^ | 343 | -1.8 | 5.6x10^-6^ | 343 | -2.1 | 1.8x10^-8^ | 328 | -2.4 | 2.1x10^-13^ | 487 | -0.7 | 0.3 |
| σ^K^ | *dpaA* | CD630_29680 | dipicolinate synthase subunit A | 214 | -5.4 | 1.1x10^-31^ | 249 | -5.7 | 1.9x10^-31^ | 286 | -3.1 | 5.7x10^-16^ | 257 | -6.0 | 3.5x10^-52^ | 506 | -0.3 | 1 |
| σ^K^ | *spoVFB* | CD630_29670 | dipicolinate synthase subunit B | 153 | -4.6 | 3.1x10^-24^ | 177 | -4.8 | 2.7x10^-24^ | 202 | -3.1 | 9.6x10^-15^ | 181 | -5.6 | 1.1x10^-44^ | 347 | -0.3 | 1 |
| ^σ^K^ | *CD1133* | CD630_11330 | hypothetical protein | 86 | -3.6 | 9.0x10^-13^ | 96 | -4.9 | 8.8x10^-21^ | 119 | -2.2 | 7.1x10^-7^ | 101 | -4.5 | 1.7x10^-18^ | 255 | 0.5 | 0.6 |
| ^σ^K^ | *CD1063A* | CD630_10631 | hypothetical protein | 63 | -4.8 | 2.5x10^-5^ | 71 | -7.7 | 6.8x10^-9^ | 86 | -2.7 | 3.3x10^-3^ | 74 | -8.3 | 3.1x10^-10^ | 167 | 0.1 | 1 |
| σ^K^ | *sigK* | CD630_12300 | sporulation factor σ^K^ | 61 | -3.6 | 1.7x10^-12^ | 68 | -4.6 | 4.8x10^-17^ | 80 | -2.5 | 8.1x10^-9^ | 68 | -7.2 | 4.8x10^-35^ | 138 | -0.2 | 1 |
| σ^K^ | *feoA* | CD630_15180 | ferrous iron transport protein | 60 | -4.7 | 3.0x10^-11^ | 70 | -4.7 | 2.0x10^-12^ | 79 | -3.2 | 9.2x10^-8^ | 72 | -5.2 | 3.9x10^-14^ | 149 | 0.0 | 1 |
| σ^K^ | *CD0749* | CD630_07490 | DNA helicase, UvrD/REP type | 60 | -3.5 | 2.8x10^-12^ | 69 | -4.1 | 4.7x10^-14^ | 91 | -1.6 | 5.0x10^-4^ | 77 | -2.8 | 1.5x10^-13^ | 226 | 1.1 | 0.01 |
| ^σ^K^ | *CD0596* | CD630_05960 | hypothetical protein (CotJA homolog) | 58 | -6.1 | 1.4x10^-4^ | 67 | -7.0 | 1.2x10^-5^ | 75 | -3.7 | 4.7x10^-3^ | 69 | -7.5 | 7.3x10^-7^ | 159 | 0.2 | 1 |
| ^σ^K^ | *cotJC1* | CD630_05980 | [spore coat assembly protein ("CotCB," [1])](#RANGE!_ENREF_1) | 53 | -6.2 | 0.02 | 63 | -4.8 | 0.03 | 69 | -3.7 | 0.1 | 63 | -6.7 | 2.7x10^-3^ | 151 | 0.3 | 1 |
| ^σ^K^ | *CD3580* | CD630_35800 | hypothetical protein | 51 | -4.7 | 2.7x10^-16^ | 59 | -5.6 | 1.5x10^-19^ | 68 | -3.0 | 9.2x10^-11^ | 62 | -5.1 | 6.6x10^-24^ | 111 | -0.5 | 0.8 |
| ^σ^K^ | *bclA1* | CD630_03320 | exosporium glycoprotein | 49 | -5.6 | 6.6x10^-7^ | 57 | -5.4 | 3.3x10^-6^ | 66 | -3.2 | 4.8x10^-4^ | 59 | -7.1 | 7.2x10^-10^ | 110 | -0.4 | 1 |
| σ^K^ | *CD0896* | CD630_08960 | hypothetical protein | 46 | -5.6 | 4.8x10^-4^ | 52 | -6.8 | 5.7x10-6 | 59 | -3.6 | 4.5x10^-3^ | 57 | -4.4 | 6.4x10^-4^ | 100 | -0.5 | 1 |
| σ^K^ | *CD2144* | CD630_21440 | putative sporulation membrane protein YtaF | 40 | -6.0 | 8.1x10^-10^ | 46 | -6.7 | 8.8x10^-11^ | 58 | -2.3 | 1.8x10^-3^ | 49 | -4.8 | 1.4x10^-9^ | 95 | -0.2 | 1 |
| σ^K^ | *CD3350* | CD630_33500 | family 2 glycosyl transferase | 34 | –Inf | 1.9x10^-8^ | 40 | -5.8 | 2.9x10^-6^ | 45 | -3.5 | 4.0x10^-4^ | 42 | -6.7 | 4.4x10^-8^ | 65 | -1.1 | 0.6 |
| σ^K^ | *CD1904* | CD630_19040 | ABC transporter permease | 32 | -3.9 | 2.9x10^-10^ | 36 | -5.0 | 1.6x10^-13^ | 41 | -3.4 | 7.5x10^-10^ | 38 | -5.8 | 5.1x10^-16^ | 72 | -0.3 | 1 |
| σ^K^ | *CD0196* | CD630_01960 | pseudo | 32 | -2.0 | 0.03 | 35 | -2.7 | 1.5x10^-4^ | 53 | -0.6 | 0.8 | 43 | -1.4 | 0.03 | 128 | 1.5 | 0.1 |
| σ^K^ | *CD2346* | CD630_23460 | membrane protein | 22 | -2.1 | 0.01 | 24 | -2.9 | 1.9x10^-5^ | 29 | -1.5 | 0.01 | 26 | -2.3 | 7.6x10^-6^ | 53 | 0.4 | 1 |
| σ^K^ | *CD0902* | CD630_09020 | cation efflux protein | 12 | -3.8 | 7.2x10^-3^ | 14 | -3.6 | 2.1x10^-3^ | 15 | -3.3 | 2.0x10^-3^ | 13 | -7.0 | 3.8x10^-6^ | 36 | 0.6 | 0.9 |
| σ^K^ | *CD2409* | CD630_24090 | hypothetical protein | 10 | -2.7 | 0.02 | 11 | -3.8 | 8.5x10^-4^ | 14 | -1.7 | 0.1 | 14 | -1.6 | 0.03 | 24 | 0.2 | 1 |

*Dep.* indicates the most downstream sigma factor on which gene expression depends upon. *BM* refers to base mean, the mean of the counts after they were divided by the size factors to adjust for different sequencing depths. This value is the mean for the sample relative to wild type. *log_2_FC* denotes log_2_fold-change. A negative value indicates that the gene was downregulated relative to wild type. ^ Indicates that gene product was detected in Lawley *et al*. proteomic analysis of purified spores [[8](#_ENREF_7)]. *–Inf* indicates that no transcript was detected in the mutant relative to wild type. See Text S2 for the references.
