# Supplementary material for: Global Analysis of the Sporulation Pathway of Clostridium difficile
Source: PLoS Genet. 2013 Aug 8;9(8):e1003660. doi: 10.1371/journal.pgen.1003660 (PMC3738446; doi:10.1371/journal.pgen.1003660)
Supplement: Table S9 — Genes induced in a Spo0A-, σF-, σE-, σG- and σK-dependent manner during growth on sporulation media. † Two factors are listed in the table for genes whose expression was dependent on both σE and σG (adjusted p-value≤0.05, log2FC≤−2). Dep. indicates the most downstream sigma factor on which gene expression depends upon. BM refers to base mean, the mean of the counts after they were divided by the size factors to adjust for different sequencing depths. This value is the mean for the sample relative to wild type. log2FC denotes log2fold-change. A negative value indicates that the gene was downregulated relative to wild type. ∧ Indicates that gene product was detected in Lawley et al. proteomic analysis of purified spores [70]. −Inf indicates that no transcript was detected in the mutant relative to wild type. See Text S2 for the references. (DOCX) [file pgen.1003660.s016.docx]

**Table S9. Genes induced in a Spo0A-, σ^F^-, σ^E^-, σ^G^-, and σ^K^-dependent manner during growth on sporulation media.**

|  |  |  |  | **Spo0A** | | | | | | **σ^F^** | | | | | | **σ^E^** | | | | | | **σ^G^** | | | | | | **σ^K^** | | | | | |  |
| --- | --- | --- | --- | --- | --- | --- | --- | --- | --- | --- | --- | --- | --- | --- | --- | --- | --- | --- | --- | --- | --- | --- | --- | --- | --- | --- | --- | --- | --- | --- | --- | --- | --- | --- |
| **Dep.** ^†^ | **Name** | **locus_tag** | **description** | **BM** | | **log_2_FC** | | **adjP** | | **BM** | | **log_2_FC** | | **adjP** | | **BM** | | **log_2_FC** | | **adjP** | | **BM** | | **log_2_FC** | | **adjP** | | **BM** | | **log_2_FC** | | **adjP** | |  |
| ^σ^K^ | *CD1067* | CD630_10670 | hypothetical protein | 9888 | | -7.6 | | 1.3x10^-60^ | | 11923 | | -2.7 | | 3.9x10^-14^ | | 10380 | | -6.1 | | 1.5x10^-50^ | | 21003 | | -0.2 | | 1 | | 8776 | | -4.7 | | 2.4x10^-32^ | |  |
| σ^K^ | *bclA3* | CD630_33490 | exosporium glycoprotein BclA3 | 2155 | | -6.1 | | 5.9x10^-11^ | | 2467 | | -3.3 | | 4.9x10^-5^ | | 2247 | | -5.7 | | 1.3x10^-10^ | | 4455 | | -0.2 | | 1 | | 1867 | | -5.5 | | 2.2x10^-9^ | |  |
| ^σ^K^ | *CD1433* | CD630_14330 | peroxiredoxin/chitinase (coat protein "CotE," [1]) | 2018 | | -7.7 | | 1.4x10^-56^ | | 2343 | | -3.2 | | 2.2x10^-18^ | | 2112 | | -6.4 | | 2.8x10^-74^ | | 3959 | | -0.4 | | 0.8 | | 1753 | | -5.8 | | 1.2x10^-43^ | |  |
| σ^K^ | *CD1063C* | CD630_10633 | hypothetical protein | 1552 | | -7.5 | | 4.2x10^-27^ | | 1879 | | -2.7 | | 5.8x10^-7^ | | 1632 | | -6.0 | | 2.9x10^-20^ | | 3104 | | -0.4 | | 0.9 | | 1369 | | -4.9 | | 3.4x10^-15^ | |  |
| σ^K^ | *CD1063B* | CD630_10632 | hypothetical protein | 1496 | | -7.5 | | 4.7x10^-27^ | | 1802 | | -2.7 | | 4.7x10^-7^ | | 1569 | | -6.2 | | 3.8x10^-21^ | | 3044 | | -0.3 | | 1 | | 1317 | | -5.1 | | 2.2x10^-15^ | |  |
| Spo0A | *CD2373* | CD630_23730 | CstA-like carbon starvation protein | 1110 | | -4.2 | | 7.5x10^-11^ | | 2036 | | -0.3 | | 1 | | 1833 | | -0.6 | | 0.5 | | 2402 | | 0.0 | | 1 | | 1584 | | -0.4 | | 1 | |  |
| σ^E^ | *CD0311* | CD630_03110 | hypothetical protein | 926 | | -6.8 | | 2.3x10^-46^ | | 1086 | | -3.0 | | 5.2x10^-16^ | | 966 | | -6.4 | | 3.1x10^-68^ | | 1715 | | -0.6 | | 0.4 | | 1077 | | -1.4 | | 4.4x10^-3^ | |  |
| ^σ^E^ | *sipL* | CD630_35670 | cell wall hydrolase (binds SpoIVA, [2]) | 882 | | -5.8 | | 2.0x10^-36^ | | 1036 | | -2.9 | | 8.3x10^-15^ | | 937 | | -4.6 | | 9.5x10^-35^ | | 1505 | | -0.9 | | 0.05 | | 1072 | | -1.2 | | 0.2 | |  |
| ^σ^K^ | *cotJB2* | CD630_24000 | spore coat peptide assembly protein CotJB2 | 797 | | -8.5 | | 2.2x10^-57^ | | 953 | | -2.9 | | 9.6x10^-15^ | | 833 | | -6.8 | | 4.6x10^-57^ | | 1537 | | -0.5 | | 0.7 | | 692 | | -6.2 | | 5.1x10^-44^ | |  |
| ^Spo0A | *spo0A* | CD630_12140 | stage 0 sporulation protein A | 722 | | -2.5 | | 3.2x10^-10^ | | 1266 | | 0.0 | | 1 | | 1187 | | -0.2 | | 0.9 | | 1302 | | -0.2 | | 1 | | 1054 | | 0.0 | | 1 | |  |
| ^σ^K^ | *cotJC2* | CD630_24010 | spore coat assembly protein CotJC2 ("CotD," [1]) | 720 | | -7.8 | | 7.4x10^-53^ | | 869 | | -2.7 | | 1.2x10^-13^ | | 753 | | -6.4 | | 5.3x10^-63^ | | 1398 | | -0.5 | | 0.7 | | 625 | | -5.9 | | 4.3x10^-42^ | |  |
| ^σ^E^ | *spoIVA* | CD630_26290 | stage IV sporulation protein A | 665 | | -7.3 | | 3.6x10^-48^ | | 764 | | -3.4 | | 3.1x10^-19^ | | 695 | | -6.5 | | 1.1x10^-62^ | | 1121 | | -1.0 | | 0.01 | | 824 | | -1.1 | | 0.2 | |  |
| ^Spo0A | *CD0514* | CD630_05140 | hemagglutinin/adhesin | 636 | | -2.1 | | 3.6x10^-7^ | | 903 | | -0.6 | | 0.4 | | 865 | | -0.7 | | 0.1 | | 873 | | -1.0 | | 0.04 | | 672 | | -1.0 | | 0.1 | |  |
| σ^E^ | *alr2* | CD630_34630 | alanine racemase | 583 | | -3.8 | | 1.7x10^-20^ | | 620 | | -3.6 | | 1.9x10^-21^ | | 590 | | -4.6 | | 1.2x10^-42^ | | 999 | | -0.7 | | 0.3 | | 739 | | -0.8 | | 0.5 | |  |
| σ^E^ | *CD3464* | CD630_34640 | hypothetical protein YdcC involved in sporulation [3] | 557 | | -4.6 | | 1.5x10^-26^ | | 609 | | -3.6 | | 2.3x10^-21^ | | 574 | | -5.0 | | 2.3x10^-42^ | | 965 | | -0.7 | | 0.2 | | 698 | | -0.9 | | 0.2 | |  |
| σ^K^ | *feoB* | CD630_15170 | ferrous iron transport protein B | 541 | | -3.3 | | 1.8x10^-12^ | | 613 | | -2.4 | | 4.1x10^-8^ | | 549 | | -3.7 | | 1.5x10^-14^ | | 1125 | | 0.0 | | 1 | | 475 | | -3.0 | | 3.0x10^-10^ | |  |
| σ^E^ | *CD3183* | CD630_31830 | peptidase | 516 | | -0.8 | | 0.1 | | 417 | | -2.3 | | 1.3x10^-9^ | | 397 | | -2.6 | | 6.2x10^-16^ | | 536 | | -1.1 | | 3.0x10^-3^ | | 460 | | -0.7 | | 0.6 | |  |
| Spo0A | *CD3489* | CD630_34890 | oligoendopeptidase F, peptidase M3B family | 512 | | -5.8 | | 7.2x10^-37^ | | 1112 | | 0.1 | | 1 | | 1288 | | 0.5 | | 0.3 | | 1151 | | 0.0 | | 1 | | 760 | | -0.4 | | 0.9 | |  |
| Spo0A | *spoIIE* | CD630_34900 | phosphoprotein phosphatase | 507 | | -6.0 | | 2.0x10^-25^ | | 1224 | | 0.4 | | 0.7 | | 1390 | | 0.7 | | 0.1 | | 1238 | | 0.3 | | 1 | | 751 | | -0.4 | | 0.9 | |  |
| Spo0A | *spoVE* | CD630_26520 | cell division/stage V sporulation protein | 420 | | -3.1 | | 3.8x10^-15^ | | 846 | | 0.2 | | 1 | | 936 | | 0.5 | | 0.5 | | 924 | | 0.2 | | 1 | | 574 | | -0.4 | | 0.9 | |  |
| ^σ^G^ | *sspA* | CD630_26880 | Small, acid-soluble spore protein alpha | 412 | | -5.8 | | 2.2x10^-25^ | | 436 | | -5.4 | | 1.5x10^-24^ | | 546 | | -1.8 | | 5.0x10^-5^ | | 466 | | -5.5 | | 1.1x10^-24^ | | 557 | | -0.7 | | 0.8 | |  |
| σ^K^ | *CD1065* | CD630_10650 | hypothetical protein | 411 | | -7.6 | | 8.1x10^-32^ | | 495 | | -2.7 | | 2.3x10^-10^ | | 429 | | -7.3 | | 6.5x10^-33^ | | 769 | | -0.6 | | 0.5 | | 394 | | -3.0 | | 3.0x10^-11^ | |  |
| Spo0A | *spoIIAB* | CD630_07710 | anti-σ^F^ factor | 374 | | -4.4 | | 8.1x10^-25^ | | 863 | | 0.4 | | 0.8 | | 881 | | 0.5 | | 0.6 | | 874 | | 0.2 | | 1 | | 571 | | -0.2 | | 1 | |  |
| Spo0A | *murG* | CD630_26510 | UDP-NAG-NAM-(pentapeptide) pyrophosphoryl-undecaprenol NAG transferase | 370 | | -3.2 | | 2.7x10^-15^ | | 763 | | 0.2 | | 1 | | 788 | | 0.3 | | 0.8 | | 803 | | 0.2 | | 1 | | 512 | | -0.3 | | 1 | |  |
| ^σ^G^ | *CD2112* | CD630_21120 | hypothetical protein | 368 | | -4.9 | | 3.2x10^-28^ | | 391 | | -4.5 | | 1.6x10^-28^ | | 633 | | -0.5 | | 0.4 | | 414 | | -4.9 | | 1.4x10^-35^ | | 411 | | -1.5 | | 5.2x10^-3^ | |  |
| ^σ^K^ | *CD2399* | CD630_23990 | hypothetical protein (CotJA superfamily) | 364 | | -8.9 | | 4.2x10^-21^ | | 427 | | -3.1 | | 4.0x10^-6^ | | 379 | | -7.6 | | 3.5x10^-19^ | | 702 | | -0.5 | | 0.9 | | 314 | | -7.0 | | 4.0x10^-16^ | |  |
| ^σ^K^ | *sleC* | CD630_05510 | spore cortex-lytic enzyme pre-pro-form | 356 | | -7.0 | | 5.9x10^-42^ | | 417 | | -3.1 | | 6.4x10^-16^ | | 372 | | -6.4 | | 2.4x10^-59^ | | 750 | | -0.2 | | 1 | | 307 | | -6.2 | | 3.4x10^-39^ | |  |
| σ^E^ | *dapG* | CD630_13220 | aspartate kinase I | 351 | | -2.2 | | 7.9x10^-8^ | | 378 | | -2.0 | | 6.3x10^-8^ | | 349 | | -2.6 | | 7.9x10^-17^ | | 483 | | -1.1 | | 8.8x10^-3^ | | 357 | | -1.2 | | 0.06 | |  |
| σ^K^ | *CD0564* | CD630_05640 | ATP-dependent protease | 343 | | -1.8 | | 5.6x10^-6^ | | 343 | | -2.1 | | 1.8x10^-8^ | | 328 | | -2.4 | | 2.1x10^-13^ | | 487 | | -0.7 | | 0.3 | | 276 | | -2.2 | | 4.0x10^-8^ | |  |
| Spo0A | *spoVD* | CD630_26560 | stage V sporulation protein D (Sporulation-specific penicillin-binding protein) | 312 | | -2.7 | | 1.1x10^-9^ | | 610 | | 0.2 | | 1 | | 551 | | -0.1 | | 1 | | 589 | | -0.1 | | 1 | | 447 | | -0.1 | | 1 | |  |
| ^Spo0A | *CD1581* | CD630_15810 | hypothetical protein | 306 | | -2.0 | | 2.1x10^-5^ | | 387 | | -1.0 | | 0.03 | | 398 | | -0.8 | | 0.1 | | 522 | | -0.2 | | 1 | | 287 | | -1.5 | | 6.3x10^-3^ | |  |
| ^Spo0A | *CD3032* | CD630_30320 | pyridoxal phosphate-dependent transferase | 306 | | -3.5 | | 3.0x10^-17^ | | 472 | | -0.7 | | 0.2 | | 454 | | -0.8 | | 0.1 | | 663 | | 0.1 | | 1 | | 347 | | -1.2 | | 0.03 | |  |
| Spo0A | *sigF* | CD630_07720 | sporulation factor σ^F^ | 304 | | -4.9 | | 1.7x10^-27^ | | 763 | | 0.6 | | 0.5 | | 740 | | 0.5 | | 0.5 | | 731 | | 0.3 | | 1 | | 470 | | -0.2 | | 1 | |  |
| ^σ^E^ | *CD3522* | CD630_35220 | hypothetical protein | 286 | | -6.1 | | 2.8x10^-10^ | | 317 | | -4.0 | | 2.1x10^-6^ | | 298 | | -6.0 | | 8.7x10^-11^ | | 449 | | -1.3 | | 0.2 | | 312 | | -1.8 | | 0.2 | |  |
| σ^F^ | *polA* | CD630_11280 | DNA polymerase I | 282 | | -2.5 | | 3.7x10^-9^ | | 317 | | -2.0 | | 3.2x10^-7^ | | 314 | | -1.9 | | 2.8x10^-9^ | | 421 | | -0.8 | | 0.1 | | 309 | | -1.0 | | 0.2 | |  |
| σ^E^ | *spoIIIAA* | CD630_11920 | stage III sporulation protein AA | 261 | | -6.7 | | 7.3x10^-38^ | | 287 | | -4.2 | | 2.9x10^-25^ | | 271 | | -6.9 | | 5.9x10^-59^ | | 418 | | -1.2 | | 1.3x10^-3^ | | 326 | | -1.1 | | 0.1 | |  |
| σ^K^ | *dpaA* | CD630_29680 | dipicolinate synthase subunit A | 249 | | -5.7 | | 1.9x10^-31^ | | 286 | | -3.1 | | 5.7x10^-16^ | | 257 | | -6.0 | | 3.5x10^-52^ | | 506 | | -0.3 | | 1 | | 214 | | -5.4 | | 1.1x10^-31^ | |  |
| ^Spo0A | *drm* | CD630_12230 | phosphopentomutase | 240 | | -2.2 | | 1.6x10^-5^ | | 390 | | -0.2 | | 1 | | 403 | | 0.0 | | 1 | | 428 | | -0.1 | | 1 | | 301 | | -0.3 | | 0.9 | |  |
| ^σ^E^ | *cspC* | CD630_22460 | subtilisin-like germination-related protease | 239 | | -4.1 | | 3.4x10^-18^ | | 264 | | -3.2 | | 8.3x10^-17^ | | 243 | | -5.0 | | 8.8x10^-41^ | | 401 | | -0.8 | | 0.1 | | 276 | | -1.3 | | 0.07 | |  |
| ^Spo0A | *CD1880* | CD630_18800 | hypothetical protein (Cupin_2 superfamily) | 215 | | -3.5 | | 2.5x10^-16^ | | 268 | | -1.8 | | 8.3x10^-6^ | | 319 | | -0.9 | | 0.04 | | 300 | | -1.5 | | 1.8x10^-5^ | | 274 | | -0.7 | | 0.7 | |  |
| Spo0A | *glmS* | CD630_01200 | glucosamine-fructose-6-phosphate aminotransferase | 203 | | -2.3 | | 1.2x10^-4^ | | 236 | | -1.6 | | 6.9x10^-3^ | | 224 | | -1.8 | | 1.6x10^-3^ | | 362 | | -0.1 | | 1 | | 185 | | -1.8 | | 0.02 | |  |
| σ^E^ | *CD3182* | CD630_31820 | D-aminoacylase | 200 | | -1.1 | | 0.1 | | 177 | | -2.1 | | 9.1x10^-4^ | | 169 | | -2.4 | | 2.0x10^-4^ | | 248 | | -0.7 | | 0.7 | | 200 | | -0.5 | | 0.9 | |  |
| Spo0A | *spoIIAA* | CD630_07700 | anti-σ^F^ factor antagonist | 200 | | -4.2 | | 1.3x10^-20^ | | 471 | | 0.5 | | 0.7 | | 439 | | 0.3 | | 0.8 | | 453 | | 0.2 | | 1 | | 305 | | -0.2 | | 1 | |  |
| ^σ^E^ | *CD3521* | CD630_35210 | peptidase T, M20B family | 193 | | -2.4 | | 9.8x10^-9^ | | 216 | | -1.9 | | 1.5x10^-6^ | | 193 | | -2.8 | | 6.7x10^-13^ | | 292 | | -0.7 | | 0.2 | | 210 | | -1.0 | | 0.3 | |  |
| Spo0A | *CD1131* | CD630_11310 | solute-binding lipoprotein | 190 | | -2.7 | | 1.3x10^-10^ | | 221 | | -1.9 | | 2.1x10^-6^ | | 228 | | -1.6 | | 8.8x10^-7^ | | 285 | | -0.9 | | 0.05 | | 212 | | -1.0 | | 0.2 | |  |
| Spo0A | *CD1823* | CD630_18230 | hypothetical protein (DUF328) | 185 | | -2.1 | | 2.9x10^-6^ | | 327 | | 0.1 | | 1 | | 273 | | -0.4 | | 0.7 | | 298 | | -0.4 | | 0.9 | | 201 | | -0.8 | | 0.4 | |  |
| σ^E^ | *brnQ-1* | CD630_12590 | Branched chain amino acid transport system carrier protein | 185 | | -5.0 | | 9.5x10^-25^ | | 235 | | -2.0 | | 2.4x10^-7^ | | 207 | | -3.2 | | 1.8x10^-19^ | | 368 | | -0.3 | | 1 | | 301 | | -0.1 | | 1 | |  |
| σ^E^ | *dpaL* | CD630_31840 | diaminopropionate ammonia-lyase | 184 | | -0.2 | | 1 | | 127 | | -2.1 | | 2.0x10^-3^ | | 118 | | -2.6 | | 2.7x10^-4^ | | 163 | | -1.0 | | 0.5 | | 139 | | -0.6 | | 0.9 | |  |
| Spo0A | *CD1824* | CD630_18240 | P-type calcium transport ATPase | 179 | | -2.3 | | 4.5x10^-8^ | | 367 | | 0.4 | | 0.8 | | 315 | | 0.1 | | 1 | | 349 | | 0.1 | | 1 | | 242 | | -0.2 | | 1 | |  |
| σ^K^ | *spoVFB* | CD630_29670 | dipicolinate synthase subunit B | 177 | | -4.8 | | 2.7x10^-24^ | | 202 | | -3.1 | | 9.6x10^-15^ | | 181 | | -5.6 | | 1.1x10^-44^ | | 347 | | -0.3 | | 1 | | 153 | | -4.6 | | 3.1x10^-24^ | |  |
| ^σ^E^ | *cspBA* | CD630_22470 | subtilisin-like germination related protease | 173 | | -3.1 | | 9.5x10^-8^ | | 188 | | -2.7 | | 6.7x10^-11^ | | 173 | | -3.7 | | 4.2x10^-14^ | | 270 | | -0.9 | | 0.1 | | 197 | | -1.0 | | 0.2 | |  |
| σ^E^ | *spoIIIAG* | CD630_11980 | stage III sporulation protein AG | 164 | | -5.9 | | 6.9x10^-29^ | | 189 | | -3.2 | | 1.3x10^-15^ | | 171 | | -6.1 | | 7.2x10^-48^ | | 284 | | -0.8 | | 0.1 | | 210 | | -1.0 | | 0.3 | |  |
| σ^E^ | *spoIIIAH* | CD630_11990 | stage III sporulation protein AH | 162 | | -6.2 | | 3.2x10^-28^ | | 187 | | -3.2 | | 2.1x10^-15^ | | 170 | | -5.4 | | 7.9x10^-26^ | | 286 | | -0.8 | | 0.2 | | 208 | | -0.9 | | 0.6 | |  |
| σ^E^ | *spoIIID* | CD630_01260 | stage III sporulation protein D | 160 | | -7.2 | | 2.0x10^-25^ | | 183 | | -3.5 | | 7.4x10^-13^ | | 167 | | -6.8 | | 2.2x10^-24^ | | 289 | | -0.7 | | 0.4 | | 190 | | -1.4 | | 0.2 | |  |
| σ^E^ | *spoIIIAB* | CD630_11930 | stage III sporulation protein AB | 153 | | -6.4 | | 6.2x10^-31^ | | 168 | | -4.1 | | 6.2x10^-23^ | | 158 | | -7.2 | | 4.5x10^-52^ | | 242 | | -1.3 | | 1.1x10^-3^ | | 199 | | -0.9 | | 0.3 | |  |
| ^σ^E^ | *CD1613* | CD630_16130 | hypothetical protein (coat protein "CotA," [1]) | 151 | | -4.2 | | 6.7x10^-3^ | | 174 | | -2.7 | | 0.1 | | 161 | | -3.6 | | 0.02 | | 341 | | 0.2 | | 1 | | 135 | | -3.4 | | 0.1 | |  |
| σ^E^ | *CD1068* | CD630_10680 | polysaccharide biosynthesis/sporulation protein | 150 | | -4.1 | | 2.1x10^-19^ | | 180 | | -2.3 | | 1.2x10^-8^ | | 154 | | -4.5 | | 2.2x10^-32^ | | 253 | | -0.8 | | 0.2 | | 190 | | -0.8 | | 0.6 | |  |
| ^σ^E^ | *CD2864* | CD630_28640 | hydrolase | 143 | | -5.3 | | 1.9x10^-25^ | | 168 | | -2.8 | | 1.8x10^-12^ | | 148 | | -5.6 | | 4.3x10^-41^ | | 267 | | -0.5 | | 0.6 | | 183 | | -0.9 | | 0.3 | |  |
| ^Spo0A | *CD1463* | CD630_14630 | hypothetical protein | 138 | | -2.3 | | 9.6x10^-8^ | | 185 | | -0.9 | | 0.1 | | 212 | | -0.4 | | 0.7 | | 189 | | -1.2 | | 8.8x10^-3^ | | 189 | | -0.1 | | 1 | |  |
| σ^E^ | *CD2084* | CD630_20840 | peptidase | 137 | | -0.8 | | 0.1 | | 113 | | -2.1 | | 3.0x10^-7^ | | 109 | | -2.3 | | 2.8x10^-10^ | | 138 | | -1.3 | | 2.8x10^-3^ | | 125 | | -0.6 | | 0.8 | |  |
| ^σ^E^ | *CD1511* | CD630_15110 | hypothetical protein (coat protein - "CotB," [[1](#_ENREF_1)]) | 137 | | -5.5 | | 1.3x10^-25^ | | 163 | | -2.7 | | 1.3x10^-11^ | | 146 | | -4.5 | | 3.5x10-32 | | 270 | | -0.3 | | 1 | | 200 | | -0.4 | | 1 | |  |
| σ^E^ | *CD2441A* | CD630_24411 | phoH-like protein | 136 | | -3.3 | | 4.4x10^-13^ | | 148 | | -2.8 | | 7.2x10^-12^ | | 139 | | -3.5 | | 9.4x10^-23^ | | 225 | | -0.7 | | 0.3 | | 161 | | -0.9 | | 0.4 | |  |
| σ^E^ | *CD1403* | CD630_14030 | synthetase | 135 | | -3.1 | | 3.8x10^-12^ | | 159 | | -2.0 | | 5.5x10^-7^ | | 145 | | -2.8 | | 9.9x10^-15^ | | 230 | | -0.6 | | 0.5 | | 177 | | -0.5 | | 0.9 | |  |
| ^σ^E^ | *CD3269* | CD630_32690 | oligoendopeptidase F, M3B family | 130 | | -0.5 | | 0.4 | | 100 | | -2.1 | | 7.1x10^-7^ | | 97 | | -2.1 | | 4.6x10^-9^ | | 149 | | -0.4 | | 0.9 | | 90 | | -1.4 | | 0.01 | |  |
| ^Spo0A | *sigG* | CD630_26420 | sporulation factor σ^G^ | 127 | | -5.4 | | 1.5x10^-24^ | | 356 | | 0.8 | | 0.2 | | 205 | | -0.8 | | 0.1 | | 294 | | 0.1 | | 1 | | 187 | | -0.4 | | 1 | |  |
| Spo0A | *CD1404* | CD630_14040 | oligopeptide transporter | 126 | | -3.4 | | 1.1x10^-4^ | | 201 | | -0.6 | | 0.7 | | 190 | | -0.8 | | 0.5 | | 236 | | -0.3 | | 1 | | 160 | | -0.7 | | 0.9 | |  |
| σ^E^ | *CD3462* | CD630_34620 | antitoxin endoAI | 126 | | -3.4 | | 1.7x10^-13^ | | 136 | | -3.0 | | 4.2x10^-13^ | | 128 | | -4.0 | | 1.1x10^-18^ | | 216 | | -0.6 | | 0.5 | | 159 | | -0.7 | | 0.7 | |  |
| σ^E^ | *CD3181* | CD630_31810 | chlorohydrolase/aminohydrolase | 122 | | -2.8 | | 2.7x10^-9^ | | 130 | | -2.7 | | 1.5x10^-8^ | | 128 | | -2.7 | | 3.0x10-8 | | 193 | | -0.7 | | 0.4 | | 156 | | -0.5 | | 1 | |  |
| Spo0A | *CD3290* | CD630_32900 | hypothetical protein | 120 | | -2.4 | | 4.4x10^-8^ | | 280 | | 0.7 | | 0.3 | | 278 | | 0.7 | | 0.2 | | 284 | | 0.6 | | 0.6 | | 171 | | 0.0 | | 1 | |  |
| σ^F^ | *CD2375* | CD630_23750 | hypothetical protein (DUF1540) | 119 | | -5.2 | | 5.8x10^-24^ | | 143 | | -2.5 | | 5.1x10^-10^ | | 240 | | 0.0 | | 1 | | 174 | | -1.6 | | 1.4x10^-5^ | | 171 | | -0.5 | | 1 | |  |
| σ^E^ | *CD1168* | CD630_11680 | membrane protein (spore coat, YlbJ [[4](#_ENREF_4)]) | 117 | | -6.0 | | 9.7x10^-27^ | | 134 | | -3.2 | | 7.2x10^-15^ | | 120 | | -6.8 | | 8.0x10^-46^ | | 201 | | -0.9 | | 0.1 | | 143 | | -1.1 | | 0.3 | |  |
| σ^E^ | *CD1380* | CD630_13800 | transporter, Major Facilitator Superfamily (MFS) | 114 | | -3.5 | | 9.7x10^-13^ | | 134 | | -2.2 | | 2.0x10^-8^ | | 130 | | -2.4 | | 1.4x10^-11^ | | 184 | | -0.9 | | 0.1 | | 114 | | -1.9 | | 6.1x10^-5^ | |  |
| Spo0A | *CD0145* | CD630_01450 | S1 RNA-binding domain-containing protein | 114 | | -2.2 | | 1.5x10^-5^ | | 190 | | -0.1 | | 1 | | 221 | | 0.4 | | 0.9 | | 178 | | -0.5 | | 0.7 | | 117 | | -1.1 | | 0.3 | |  |
| Spo0A | *CD0622* | CD630_06220 | hypothetical protein (DUF1629) | 113 | | -7.5 | | 2.1x10^-10^ | | 192 | | -0.7 | | 0.7 | | 167 | | -1.2 | | 0.2 | | 210 | | -0.6 | | 0.9 | | 171 | | -0.4 | | 1 | |  |
| Spo0A | *CD1222* | CD630_12220 | integrase site-specific recombinase XerD-like | | 108 | | -3.1 | | 1.3x10^-12^ | | 192 | | -0.2 | | 1 | | 196 | | -0.1 | | 1 | | 212 | | -0.1 | | 1 | | 154 | | -0.2 | | 1 | |
| σ^E^ | *CD0129* | CD630_01290 | hypothetical protein (YyaC, in sporulating bacteria) | 105 | | -5.1 | | 8.9x10^-23^ | | 126 | | -2.5 | | 6.7x10^-10^ | | 109 | | -5.1 | | 3.0x10^-33^ | | 198 | | -0.5 | | 0.8 | | 148 | | -0.5 | | 0.9 | |  |
| ^σ^E^ | *CD1319* | CD630_13190 | polysaccharide deacetylase | 103 | | -5.0 | | 1.4x10^-21^ | | 113 | | -3.7 | | 7.9x10^-18^ | | 107 | | -5.2 | | 3.5x10^-34^ | | 155 | | -1.4 | | 2.9x10^-4^ | | 126 | | -1.1 | | 0.2 | |  |
| σ^E^ | *EndoA* | CD630_34610 | endoribonuclease toxin | 103 | | -3.5 | | 3.9x10^-13^ | | 112 | | -3.0 | | 9.6x10^-12^ | | 104 | | -4.1 | | 4.8x10^-17^ | | 181 | | -0.5 | | 0.7 | | 139 | | -0.5 | | 0.9 | |  |
| σ^E^ | *CD2833* | CD630_28330 | calcium-transporting ATPase | 100 | | -4.8 | | 1.7x10^-18^ | | 119 | | -2.7 | | 1.1x10^-10^ | | 102 | | -6.1 | | 9.7x10^-40^ | | 184 | | -0.6 | | 0.6 | | 124 | | -1.0 | | 0.4 | |  |
| σ^E^ | *CD1928* | CD630_19280 | membrane protein | 100 | | -7.4 | | 2.0x10^-28^ | | 114 | | -3.4 | | 7.2x10^-16^ | | 105 | | -6.0 | | 5.7x10^-38^ | | 152 | | -1.5 | | 1.4x10^-4^ | | 119 | | -1.3 | | 0.1 | |  |
| σ^E^ | *CD1930* | CD630_19300 | hypothetical protein (ComEC-related) | 99 | | -1.8 | | 4.8x10^-5^ | | 94 | | -2.7 | | 1.5x10^-10^ | | 85 | | -4.1 | | 1.6x10^-24^ | | 149 | | -0.5 | | 0.8 | | 102 | | -0.9 | | 0.6 | |  |
| ^σ^G^(σ^E^) | *sspB* | CD630_32490 | Small, acid-soluble spore protein beta | 97 | | -8.1 | | 1.8x10^-11^ | | 102 | | -7.0 | | 1.2x10^-11^ | | 115 | | -2.8 | | 6.4x10^-4^ | | 109 | | -7.0 | | 7.3x10^-11^ | | 129 | | -0.8 | | 1 | |  |
| ^σ^K^ | *CD1133* | CD630_11330 | hypothetical protein | 96 | | -4.9 | | 8.8x10^-21^ | | 119 | | -2.2 | | 7.1x10^-7^ | | 101 | | -4.5 | | 1.7x10^-18^ | | 255 | | 0.5 | | 0.6 | | 86 | | -3.6 | | 9.0x10^-13^ | |  |
| Spo0A | *prfB* | CD630_01440 | peptide chain release factor 2 (RF-2) | 96 | | -2.2 | | 1.1x10^-6^ | | 197 | | 0.5 | | 0.7 | | 216 | | 0.7 | | 0.2 | | 181 | | 0.1 | | 1 | | 116 | | -0.5 | | 0.9 | |  |
| σ^E^ | *CD0017* | CD630_00170 | DNA binding protein | 95 | | -2.1 | | 1.0x10^-4^ | | 105 | | -1.8 | | 1.2x10^-3^ | | 97 | | -2.2 | | 4.7x10^-5^ | | 141 | | -0.7 | | 0.5 | | 101 | | -0.9 | | 0.5 | |  |
| Spo0A | *coaE* | CD630_11290 | dephospho-CoA kinase | 94 | | -2.7 | | 1.7x10^-8^ | | 113 | | -1.7 | | 9.0x10^-5^ | | 113 | | -1.6 | | 3.2x10^-5^ | | 155 | | -0.5 | | 0.6 | | 107 | | -0.9 | | 0.4 | |  |
| σ^E^ | *CD3298* | CD630_32980 | ATP/GTP-binding protein | 92 | | -6.6 | | 2.6x10^-16^ | | 112 | | -2.6 | | 1.0x10^-5^ | | 97 | | -5.6 | | 4.7x10^-15^ | | 168 | | -0.7 | | 0.6 | | 107 | | -1.4 | | 0.3 | |  |
| σ^E^ | *CD1066* | CD630_10660 | hypothetical protein | 91 | | -4.0 | | 1.8x10^-15^ | | 105 | | -2.7 | | 2.4x10^-10^ | | 95 | | -4.1 | | 4.6x10^-24^ | | 149 | | -0.9 | | 0.1 | | 96 | | -1.7 | | 2.3x10^-3^ | |  |
| σ^E^ | *CD3177* | CD630_31770 | xanthine dehydrogenase | 90 | | -4.5 | | 1.2x10^-11^ | | 103 | | -3.0 | | 4.8x10^-8^ | | 98 | | -3.5 | | 3.5x10^-9^ | | 153 | | -0.8 | | 0.3 | | 125 | | -0.6 | | 1 | |  |
| σ^E^ | *dnaX* | CD630_00160 | DNA polymerase III subunits gamma and tau | 85 | | -3.4 | | 4.2x10^-7^ | | 97 | | -2.4 | | 1.5x10^-4^ | | 90 | | -3.2 | | 9.3x10^-7^ | | 141 | | -0.7 | | 0.6 | | 102 | | -0.9 | | 0.6 | |  |
| σ^E^ | *CD2800* | CD630_28000 | membrane protein | 84 | | -4.5 | | 2.4x10^-18^ | | 95 | | -3.2 | | 1.7x10^-13^ | | 86 | | -5.4 | | 2.5x10^-32^ | | 132 | | -1.1 | | 0.01 | | 96 | | -1.4 | | 0.03 | |  |
| Spo0A | *mgsA* | CD630_11530 | methylglyoxal synthase (MGS) | 84 | | -2.0 | | 1.3x10^-5^ | | 165 | | 0.4 | | 0.8 | | 158 | | 0.3 | | 0.8 | | 151 | | 0.0 | | 1 | | 105 | | -0.3 | | 1 | |  |
| Spo0A | *mrdB* | CD630_11520 | Rod shape-determining protein MrdB | 83 | | -2.0 | | 2.5x10^-5^ | | 162 | | 0.4 | | 0.8 | | 164 | | 0.4 | | 0.6 | | 155 | | 0.1 | | 1 | | 103 | | -0.3 | | 1 | |  |
| Spo0A | *pdp* | CD630_12250 | pyrimidine-nucleoside phosphorylase | 83 | | -2.7 | | 3.6x10^-4^ | | 155 | | 0.1 | | 1 | | 150 | | 0.0 | | 1 | | 176 | | 0.2 | | 1 | | 107 | | -0.5 | | 1 | |  |
| ^σ^E^ | *CD0761* | CD630_07610 | ATP-dependent RNA helicase | 81 | | -2.8 | | 9.7x10^-10^ | | 89 | | -2.4 | | 1.3x10^-8^ | | 83 | | -2.9 | | 1.3x10^-14^ | | 111 | | -1.4 | | 1.1x10^-3^ | | 90 | | -1.0 | | 0.4 | |  |
| Spo0A | *bcp* | CD630_18220 | thiol peroxidase | 80 | | -2.0 | | 3.4x10^-4^ | | 151 | | 0.3 | | 1 | | 123 | | -0.2 | | 1 | | 143 | | 0.0 | | 1 | | 92 | | -0.6 | | 0.8 | |  |
| σ^E^ | *era* | CD630_24370 | GTPase Era | 79 | | -1.4 | | 5.7x10^-3^ | | 75 | | -1.9 | | 1.1x10^-5^ | | 72 | | -2.2 | | 3.2x10^-8^ | | 101 | | -0.8 | | 0.3 | | 81 | | -0.6 | | 0.8 | |  |
| σ^F^ | *CD2376* | CD630_23760 | membrane protein YtvI involved in sporulation [[4](#_ENREF_4)] | 74 | | -3.7 | | 3.1x10^-13^ | | 81 | | -3.1 | | 7.1x10^-13^ | | 105 | | -1.1 | | 8.1x10^-3^ | | 111 | | -1.3 | | 4.7x10^-3^ | | 97 | | -0.6 | | 0.7 | |  |
| σ^E^ | *CD1940* | CD630_19400 | membrane protein (DUF3866 superfamily) | 73 | | -6.3 | | 3.4x10^-23^ | | 84 | | -3.1 | | 8.8x10^-13^ | | 78 | | -4.4 | | 1.6x10^-24^ | | 128 | | -0.8 | | 0.3 | | 106 | | -0.5 | | 0.9 | |  |
| ^σ^G^ | *CD2687A* | CD630_26871 | hypothetical protein | 73 | | -4.9 | | 3.0x10^-18^ | | 80 | | -3.6 | | 1.0x10^-15^ | | 118 | | -0.7 | | 0.1 | | 98 | | -2.1 | | 7.0x10^-8^ | | 92 | | -0.9 | | 0.4 | |  |
| σ^E^ | *CD1321* | CD630_13210 | sporulation protein (YlmC) | 72 | | -2.1 | | 6.7x10^-6^ | | 72 | | -2.6 | | 4.4x10^-9^ | | 68 | | -3.1 | | 5.5x10^-13^ | | 95 | | -1.2 | | 0.01 | | 70 | | -1.4 | | 0.06 | |  |
| σ^E^ | *CD3251* | CD630_32510 | dehydrogenase | 71 | | -1.5 | | 8.1x10^-3^ | | 73 | | -1.6 | | 7.5x10^-4^ | | 64 | | -2.5 | | 5.0x10^-9^ | | 95 | | -0.7 | | 0.5 | | 77 | | -0.5 | | 0.9 | |  |
| ^σ^K^ | *CD1063A* | CD630_10631 | hypothetical protein | 71 | | -7.7 | | 6.8x10^-9^ | | 86 | | -2.7 | | 3.3x10^-3^ | | 74 | | -8.3 | | 3.1x10^-10^ | | 167 | | 0.1 | | 1 | | 63 | | -4.8 | | 2.5x10^-5^ | |  |
| ^σ^G^ | *CD1486* | CD630_14860 | ribosome recycling factor | 71 | | -5.8 | | 4.2x10^-21^ | | 75 | | -5.0 | | 6.4x10^-23^ | | 125 | | -0.4 | | 0.7 | | 80 | | -5.5 | | 1.3x10^-27^ | | 86 | | -1.1 | | 0.3 | |  |
| σ^K^ | *feoA* | CD630_15180 | ferrous iron transport protein | 70 | | -4.7 | | 2.0x10^-12^ | | 79 | | -3.2 | | 9.2x10^-8^ | | 72 | | -5.2 | | 3.9x10^-14^ | | 149 | | 0.0 | | 1 | | 60 | | -4.7 | | 3.0x10^-11^ | |  |
| σ^E^ | *CD1398* | CD630_13980 | peptidase, M20D family | 69 | | -4.9 | | 2.4x10^-18^ | | 82 | | -2.6 | | 1.7x10^-9^ | | 74 | | -4.1 | | 2.7x10^-22^ | | 119 | | -0.8 | | 0.2 | | 83 | | -1.2 | | 0.2 | |  |
| σ^E^ | *CD3465* | CD630_34650 | hypothetical protein (CBS domain) | 69 | | -2.8 | | 1.7x10^-8^ | | 72 | | -2.9 | | 4.0x10^-10^ | | 71 | | -3.0 | | 1.9x10^-11^ | | 109 | | -0.7 | | 0.3 | | 89 | | -0.5 | | 0.9 | |  |
| σ^K^ | *CD0749* | CD630_07490 | DNA helicase, UvrD/REP type | 69 | | -4.1 | | 4.7x10^-14^ | | 91 | | -1.6 | | 5.0x10^-4^ | | 77 | | -2.8 | | 1.5x10^-13^ | | 226 | | 1.1 | | 0.01 | | 60 | | -3.5 | | 2.8x10^-12^ | |  |
| σ^K^ | *sigK* | CD630_12300 | sporulation factor σ^K^ | 68 | | -4.6 | | 4.8x10^-17^ | | 80 | | -2.5 | | 8.1x10^-9^ | | 68 | | -7.2 | | 4.8x10^-35^ | | 138 | | -0.2 | | 1 | | 61 | | -3.6 | | 1.7x10^-12^ | |  |
| ^σ^K^ | *CD0596* | CD630_05960 | hypothetical protein (CotJA homolog) | 67 | | -7.0 | | 1.2x10^-5^ | | 75 | | -3.7 | | 4.7x10^-3^ | | 69 | | -7.5 | | 7.3x10^-7^ | | 159 | | 0.2 | | 1 | | 58 | | -6.1 | | 1.4x10^-4^ | |  |
| σ^E^ | *CD1396* | CD630_13960 | amino acid amidase | 66 | | -2.0 | | 5.1x10^-5^ | | 75 | | -1.5 | | 1.2x10^-3^ | | 69 | | -2.0 | | 7.0x10^-7^ | | 104 | | -0.4 | | 0.9 | | 75 | | -0.6 | | 0.8 | |  |
| ^σ^E^ | *CD3258* | CD630_32580 | Iron hydrogenase | 66 | | -4.9 | | 3.3x10^-16^ | | 71 | | -4.1 | | 1.3x10^-17^ | | 69 | | -5.0 | | 8.4x10^-28^ | | 103 | | -1.2 | | 8.8x10^-3^ | | 71 | | -1.8 | | 1.5x10^-3^ | |  |
| Spo0A | *ptsG-BC* | CD630_26670 | PTS system glucose-specific transporter subunit IIBC | 65 | | -3.0 | | 0.02 | | 119 | | -0.1 | | 1 | | 84 | | -1.3 | | 0.4 | | 111 | | -0.5 | | 1 | | 68 | | -1.4 | | 0.7 | |  |
| Spo0A | *pupG* | CD630_12240 | Purine nucleoside phosphorylase | 65 | | -2.2 | | 4.8x10^-6^ | | 107 | | -0.1 | | 1 | | 110 | | 0.0 | | 1 | | 118 | | -0.1 | | 1 | | 86 | | -0.2 | | 1 | |  |
| Spo0A | *CD1878A* | CD630_18781 | pseudo | 64 | | -2.1 | | 0.03 | | 91 | | -0.6 | | 0.8 | | 101 | | -0.3 | | 1 | | 108 | | -0.3 | | 1 | | 70 | | -0.9 | | 0.7 | |  |
| Spo0A | *CD2435A* | CD630_24351 | hypothetical protein (Yqz-like) | 64 | | -2.0 | | 7.2x10^-5^ | | 73 | | -1.4 | | 2.8x10^-3^ | | 75 | | -1.2 | | 4.4x10^-3^ | | 87 | | -0.9 | | 0.1 | | 70 | | -0.7 | | 0.7 | |  |
| Spo0A | *sigE* | CD630_26430 | sporulation factor σ^E^ | 64 | | -4.6 | | 7.0x10^-16^ | | 205 | | 1.1 | | 0.01 | | 160 | | 0.6 | | 0.4 | | 144 | | 0.1 | | 1 | | 101 | | -0.1 | | 1 | |  |
| Spo0A | *CD1221* | CD630_12210 | membrane protein (SpoIIM homolog) | 63 | | -5.6 | | 4.4x10^-21^ | | 106 | | -0.7 | | 0.5 | | 112 | | -0.4 | | 0.8 | | 120 | | -0.5 | | 0.8 | | 83 | | -0.8 | | 0.6 | |  |
| ^σ^K^ | *cotJC1* | CD630_05980 | spore coat assembly protein ("CotCB," [[1](#_ENREF_1)]) | 63 | | -4.8 | | 0.03 | | 69 | | -3.7 | | 0.1 | | 63 | | -6.7 | | 2.7x10^-3^ | | 151 | | 0.3 | | 1 | | 53 | | -6.2 | | 0.02 | |  |
| Spo0A | *CD2720* | CD630_27200 | transporter | 61 | | -2.0 | | 8.2x10^-5^ | | 71 | | -1.4 | | 7.7x10^-3^ | | 68 | | -1.6 | | 2.2x10^-4^ | | 116 | | 0.2 | | 1 | | 61 | | -1.1 | | 0.2 | |  |
| σ^F^ | *uppS* | CD630_27620 | undecaprenyl pyrophosphate synthetase | 60 | | -1.5 | | 8.4x10^-3^ | | 56 | | -2.4 | | 1.3x10^-6^ | | 114 | | 0.6 | | 0.5 | | 73 | | -1.2 | | 0.1 | | 57 | | -1.0 | | 0.3 | |  |
| Spo0A | *CD1233* | CD630_12330 | cell surface protein | 59 | | -3.0 | | 1.9x10^-8^ | | 109 | | 0.0 | | 1 | | 111 | | 0.1 | | 1 | | 123 | | 0.1 | | 1 | | 79 | | -0.4 | | 1 | |  |
| ^σ^K^ | *CD3580* | CD630_35800 | hypothetical protein | 59 | | -5.6 | | 1.5x10^-19^ | | 68 | | -3.0 | | 9.2x10^-11^ | | 62 | | -5.1 | | 6.6x10^-24^ | | 111 | | -0.5 | | 0.8 | | 51 | | -4.7 | | 2.7x10^-16^ | |  |
| ^σ^G^ | *CD0684* | CD630_06840 | ATP-dependent peptidase, M41 family | 58 | | -3.7 | | 6.9x10^-12^ | | 60 | | -4.3 | | 7.9x10^-18^ | | 115 | | 0.1 | | 1 | | 65 | | -3.9 | | 6.9x10^-20^ | | 67 | | -1.2 | | 0.2 | |  |
| σ^E^ | *CD0760* | CD630_07600 | Ca^2+^/Na^+^ antiporter | 58 | | -3.6 | | 9.9x10^-10^ | | 68 | | -2.2 | | 8.6x10^-5^ | | 59 | | -3.9 | | 4.7x10^-10^ | | 90 | | -1.0 | | 0.1 | | 64 | | -1.3 | | 0.1 | |  |
| ^σ^K^ | *bclA1* | CD630_03320 | exosporium glycoprotein | 57 | | -5.4 | | 3.3x10^-6^ | | 66 | | -3.2 | | 4.8x10^-4^ | | 59 | | -7.1 | | 7.2x10^-10^ | | 110 | | -0.4 | | 1 | | 49 | | -5.6 | | 6.6x10^-7^ | |  |
| σ^F^ | *CD1285* | CD630_12850 | Holliday junction resolvase-like protein | 57 | | -2.2 | | 0.1 | | 56 | | -2.9 | | 0.02 | | 60 | | -2.1 | | 0.1 | | 84 | | -0.8 | | 0.9 | | 55 | | -1.4 | | 0.6 | |  |
| σ^E^ | *CD1320* | CD630_13200 | M16 family peptidase | 56 | | -2.3 | | 7.4x10^-4^ | | 58 | | -2.6 | | 2.3x10^-4^ | | 55 | | -2.9 | | 2.9x10^-5^ | | 75 | | -1.2 | | 0.2 | | 56 | | -1.3 | | 0.2 | |  |
| σ^E^ | *CD2637* | CD630_26370 | two-component sensor histidine kinase | 56 | | -2.2 | | 5.2x10^-5^ | | 59 | | -2.2 | | 3.2x10^-6^ | | 55 | | -2.6 | | 1.5x10^-7^ | | 75 | | -1.1 | | 0.04 | | 56 | | -1.2 | | 0.4 | |  |
| Spo0A | *CD1229* | CD630_12290 | peptidoglycan glycosyltransferase | 55 | | -3.0 | | 5.5x10^-6^ | | 72 | | -1.3 | | 0.1 | | 81 | | -0.7 | | 0.4 | | 82 | | -1.0 | | 0.3 | | 61 | | -1.1 | | 0.4 | |  |
| σ^E^ | *spoIIIAF* | CD630_11970 | stage III sporulation protein AF | 55 | | -5.0 | | 5.8x10^-17^ | | 61 | | -3.6 | | 2.4x10^-14^ | | 56 | | -6.2 | | 5.6x10^-30^ | | 94 | | -0.8 | | 0.2 | | 69 | | -1.0 | | 0.3 | |  |
| σ^E^ | *CD2121* | CD630_21210 | hypothetical protein | 54 | | -5.4 | | 2.9x10^-8^ | | 64 | | -2.7 | | 5.1x10^-4^ | | 56 | | -5.5 | | 2.1x10^-8^ | | 96 | | -0.7 | | 0.8 | | 62 | | -1.4 | | 0.4 | |  |
| σ^E^ | *CD3178* | CD630_31780 | D-hydantoinase | 54 | | -3.8 | | 3.2x10^-11^ | | 60 | | -2.8 | | 2.4x10^-9^ | | 57 | | -3.4 | | 3.6x10^-11^ | | 83 | | -1.1 | | 0.05 | | 65 | | -0.9 | | 0.5 | |  |
| Spo0A | *CD1967* | CD630_19670 | hypothetical protein | 53 | | -3.7 | | 4.6x10^-11^ | | 159 | | 1.0 | | 0.04 | | 125 | | 0.5 | | 0.6 | | 137 | | 0.5 | | 0.7 | | 90 | | 0.2 | | 1 | |  |
| σ^K^ | *CD0896* | CD630_08960 | hypothetical protein | 52 | | -6.8 | | 5.7x10^-6^ | | 59 | | -3.6 | | 4.5x10^-3^ | | 57 | | -4.4 | | 6.4x10^-4^ | | 100 | | -0.5 | | 1 | | 46 | | -5.6 | | 4.8x10^-4^ | |  |
| σ^F^ | *CD1130* | CD630_11300 | lytic transglycosylase-like protein | 51 | | -2.7 | | 1.2x10^-6^ | | 58 | | -2.0 | | 3.3x10^-5^ | | 58 | | -1.8 | | 8.7x10^-6^ | | 76 | | -0.9 | | 0.2 | | 53 | | -1.3 | | 0.1 | |  |
| σ^E^ | *CD2799A* | CD630_27991 | hypothetical protein | 50 | | -3.3 | | 3.5x10^-10^ | | 58 | | -2.4 | | 2.2x10^-7^ | | 50 | | -4.1 | | 3.9x10^-18^ | | 79 | | -0.9 | | 0.1 | | 59 | | -1.0 | | 0.4 | |  |
| σ^E^ | *CD1085* | CD630_10850 | membrane protein | 49 | | -1.5 | | 0.02 | | 45 | | -2.5 | | 2.7x10^-7^ | | 45 | | -2.4 | | 1.5x10^-7^ | | 61 | | -1.0 | | 0.1 | | 49 | | -0.8 | | 0.5 | |  |
| Spo0A | *CD3287* | CD630_32870 | oxidoreductase, FAD dependent | 48 | | -2.4 | | 8.5x10^-6^ | | 76 | | -0.4 | | 0.8 | | 91 | | 0.2 | | 1 | | 78 | | -0.5 | | 0.8 | | 52 | | -1.0 | | 0.3 | |  |
| ^σ^G^ | *rbr* | CD630_28450 | rubrerythrin | 48 | | -4.4 | | 1.6x10^-13^ | | 51 | | -3.8 | | 2.1x10^-14^ | | 96 | | 0.0 | | 1 | | 56 | | -3.5 | | 8.4x10^-16^ | | 66 | | -0.5 | | 1 | |  |
| Spo0A | *aroC* | CD630_18350 | chorismate synthase | 48 | | -2.0 | | 4.4x10^-3^ | | 83 | | 0.1 | | 1 | | 95 | | 0.5 | | 0.8 | | 93 | | 0.2 | | 1 | | 54 | | -0.6 | | 0.9 | |  |
| Spo0A | *CD1219* | CD630_12190 | hypothetical protein | 48 | | -2.5 | | 4.7x10^-6^ | | 83 | | 0.0 | | 1 | | 83 | | 0.0 | | 1 | | 85 | | -0.2 | | 1 | | 59 | | -0.5 | | 0.9 | |  |
| σ^E^ | *spoIIIAE* | CD630_11960 | stage III sporulation protein AE | 47 | | -4.9 | | 1.8x10^-14^ | | 52 | | -3.6 | | 4.4x10^-13^ | | 49 | | -5.1 | | 7.8x10^-22^ | | 76 | | -1.0 | | 0.1 | | 63 | | -0.7 | | 0.8 | |  |
| Spo0A | *ptsG-A* | CD630_26660 | PTS system glucose-specific transporter subunit IIA | 46 | | -2.9 | | 0.02 | | 84 | | -0.1 | | 1 | | 59 | | -1.3 | | 0.3 | | 79 | | -0.5 | | 1 | | 46 | | -1.7 | | 0.5 | |  |
| ^σ^G^ | *spoVAD* | CD630_07740 | stage V sporulation protein AD | 46 | | -4.2 | | 5.7x10^-13^ | | 48 | | -5.1 | | 1.9x10^-19^ | | 66 | | -1.2 | | 7.7x10^-3^ | | 52 | | -4.7 | | 3.3x10^-22^ | | 58 | | -0.9 | | 0.6 | |  |
| Spo0A | *spoIIGA* | CD630_26440 | sporulation σ^E^-processing peptidase | 46 | | -4.3 | | 6.3x10^-13^ | | 126 | | 0.8 | | 0.2 | | 119 | | 0.7 | | 0.3 | | 99 | | 0.0 | | 1 | | 64 | | -0.5 | | 0.9 | |  |
| σ^K^ | *CD2144* | CD630_21440 | putative sporulation membrane protein YtaF | 46 | | -6.7 | | 8.8x10^-11^ | | 58 | | -2.3 | | 1.8x10^-3^ | | 49 | | -4.8 | | 1.4x10^-9^ | | 95 | | -0.2 | | 1 | | 40 | | -6.0 | | 8.1x10^-10^ | |  |
| σ^F^ | *CD0125* | CD630_01250 | cell wall endopeptidase (SpoIIQ homolog, [[5](#_ENREF_5)]) | 46 | | -4.2 | | 1.3x10^-12^ | | 49 | | -3.6 | | 3.4x10^-13^ | | 101 | | 0.3 | | 1 | | 72 | | -1.1 | | 0.05 | | 58 | | -0.8 | | 0.6 | |  |
| Spo0A | *pflE* | CD630_32830 | pyruvate formate-lyase (activating enzyme) | 45 | | -2.0 | | 0.01 | | 82 | | 0.2 | | 1 | | 73 | | -0.1 | | 1 | | 75 | | -0.2 | | 1 | | 50 | | -0.7 | | 0.9 | |  |
| ^σ^E^ | *CD3652* | CD630_36520 | peptidase, M1 family | 45 | | -4.9 | | 5.6x10^-14^ | | 52 | | -3.0 | | 5.1x10^-10^ | | 46 | | -8.5 | | 1.1x10^-23^ | | 80 | | -0.7 | | 0.5 | | 60 | | -0.7 | | 0.8 | |  |
| σ^E^ | *CD1167* | CD630_11670 | integrase/recombinase | 45 | | -6.2 | | 2.1x10^-17^ | | 52 | | -3.1 | | 6.3x10^-11^ | | 48 | | -4.8 | | 5.7x10^-23^ | | 82 | | -0.6 | | 0.5 | | 61 | | -0.7 | | 0.9 | |  |
| σ^G^ | *CD3312* | CD630_33120 | transporter, Major Facilitator Superfamily (MFS) | 45 | | -4.3 | | 1.9x10^-9^ | | 48 | | -4.0 | | 6.0x10^-10^ | | 62 | | -1.4 | | 5.5x10^-3^ | | 50 | | -5.2 | | 4.0x10^-12^ | | 61 | | -0.6 | | 1 | |  |
| σ^E^ | *CD1395* | CD630_13950 | membrane protein (YrvL superfamily) | 45 | | -3.2 | | 4.2x10^-9^ | | 54 | | -1.9 | | 5.6x10^-5^ | | 49 | | -2.5 | | 2.4x10^-8^ | | 79 | | -0.4 | | 0.9 | | 59 | | -0.5 | | 0.9 | |  |
| Spo0A | *CD1235* | CD630_12350 | hypothetical protein | 45 | | -2.8 | | 4.3x10^-3^ | | 87 | | 0.2 | | 1 | | 80 | | 0.0 | | 1 | | 98 | | 0.3 | | 1 | | 60 | | -0.3 | | 1 | |  |
| σ^E^ | *CD0557* | CD630_05570 | uridine kinase | 44 | | -3.7 | | 5.1x10^-10^ | | 61 | | -1.2 | | 0.03 | | 52 | | -2.2 | | 7.9x10^-8^ | | 80 | | -0.5 | | 0.9 | | 54 | | -0.9 | | 0.5 | |  |
| σ^E^ | *CD0296* | CD630_02960 | hypothetical protein | 43 | | -2.5 | | 1.3x10^-5^ | | 51 | | -1.6 | | 2.0x10^-3^ | | 44 | | -2.8 | | 2.5x10-5 | | 81 | | -0.1 | | 1 | | 44 | | -1.3 | | 0.2 | |  |
| σ^G^ | *CD1430* | CD630_14300 | delta-lactam-biosynthetic de-N-acteylase | 43 | | -4.7 | | 3.4x10^-13^ | | 45 | | -4.7 | | 1.2x10^-17^ | | 84 | | -0.1 | | 1 | | 49 | | -4.3 | | 2.2x10^-19^ | | 60 | | -0.5 | | 0.9 | |  |
| σ^E^ | *CD3270* | CD630_32700 | magnesium transport ATPase, MgtC/SapB family | 43 | | -0.7 | | 0.4 | | 36 | | -2.0 | | 0.04 | | 34 | | -2.3 | | 0.01 | | 65 | | 0.2 | | 1 | | 30 | | -1.8 | | 0.1 | |  |
| σ^E^ | *CD1086* | CD630_10860 | peptidase, M20D family | 42 | | -1.4 | | 0.03 | | 41 | | -1.9 | | 1.4x10^-4^ | | 37 | | -2.6 | | 4.3x10^-8^ | | 51 | | -1.1 | | 0.1 | | 45 | | -0.5 | | 0.9 | |  |
| σ^E^ | *bclA2* | CD630_32300 | exosporium glycoprotein | 42 | | -4.2 | | 0.1 | | 48 | | -2.9 | | 0.2 | | 42 | | -5.9 | | 0.01 | | 92 | | 0.0 | | 1 | | 36 | | -4.8 | | 0.1 | |  |
| Spo0A | *CD3569* | CD630_35690 | sporulation-specific protease (YabG) | 42 | | -2.7 | | 3.4x10^-7^ | | 59 | | -0.9 | | 0.2 | | 50 | | -1.7 | | 2.1x10-^4^ | | 98 | | 0.5 | | 0.8 | | 44 | | -1.3 | | 0.1 | |  |
| σ^E^ | *CD3551B* | CD630_35512 | hypothetical protein | 42 | | -6.6 | | 8.5x10^-13^ | | 48 | | -3.3 | | 7.5x10^-8^ | | 48 | | -3.2 | | 1.0x10^-6^ | | 67 | | -1.2 | | 0.1 | | 54 | | -0.9 | | 0.7 | |  |
| σ^E^ | *CD1740* | CD630_17400 | glycine/sarcosine/betaine reductase complex component B subunits alpha and beta | 41 | | -3.4 | | 8.8x10^-9^ | | 46 | | -2.7 | | 3.7x10^-8^ | | 43 | | -3.5 | | 5.9x10^-14^ | | 69 | | -0.7 | | 0.5 | | 44 | | -1.5 | | 0.05 | |  |
| ^σ^E^ | *CD3007* | CD630_30070 | hypothetical protein | 41 | | -3.3 | | 2.5x10^-8^ | | 52 | | -1.6 | | 1.8x10^-3^ | | 48 | | -2.1 | | 2.1x10^-6^ | | 81 | | -0.1 | | 1 | | 41 | | -1.8 | | 5.3x10^-3^ | |  |
| Spo0A | *CD2374* | CD630_23740 | hypothetical protein | 41 | | -4.6 | | 4.4x10^-13^ | | 58 | | -1.3 | | 0.02 | | 53 | | -1.7 | | 1.2x10^-4^ | | 79 | | -0.3 | | 1 | | 58 | | -0.5 | | 1 | |  |
| σ^E^ | *CD3440* | CD630_34400 | glycoside hydrolase-type carbohydrate-binding protein | 40 | | -3.5 | | 1.2x10^-9^ | | 45 | | -2.7 | | 3.8x10^-8^ | | 41 | | -3.9 | | 2.5x10^-15^ | | 68 | | -0.7 | | 0.5 | | 50 | | -0.8 | | 0.8 | |  |
| ^σ^G^ | *CD2635* | CD630_26350 | hypothetical protein (YIEGIA family) | 40 | | -3.8 | | 9.1x10^-11^ | | 43 | | -3.7 | | 5.6x10^-13^ | | 58 | | -1.0 | | 0.05 | | 45 | | -4.0 | | 5.7x10^-17^ | | 49 | | -0.9 | | 0.5 | |  |
| σ^K^ | *CD3350* | CD630_33500 | family 2 glycosyl transferase | 40 | | -5.8 | | 2.9x10^-6^ | | 45 | | -3.5 | | 4.0x10^-4^ | | 42 | | -6.7 | | 4.4x10^-8^ | | 65 | | -1.1 | | 0.6 | | 34 | | –Inf | | 1.9x10^-8^ | |  |
| σ^E^ | *tepA* | CD630_13230 | protein export-enhancing factor | 40 | | -3.2 | | 1.5x10^-3^ | | 45 | | -2.4 | | 9.3x10^-3^ | | 44 | | -2.5 | | 8.6x10^-3^ | | 57 | | -1.3 | | 0.4 | | 43 | | -1.3 | | 0.6 | |  |
| σ^E^ | *CD3457* | CD630_34570 | hypothetical protein | 40 | | -3.6 | | 1.0x10^-9^ | | 46 | | -2.3 | | 2.4x10^-6^ | | 43 | | -3.1 | | 6.8x10^-11^ | | 66 | | -0.7 | | 0.5 | | 46 | | -1.1 | | 0.3 | |  |
| Spo0A | *CD3289* | CD630_32890 | hypothetical protein | 38 | | -2.8 | | 5.8x10^-7^ | | 85 | | 0.5 | | 0.7 | | 97 | | 0.9 | | 0.1 | | 99 | | 0.7 | | 0.4 | | 52 | | -0.3 | | 1 | |  |
| Spo0A | *CD1233B* | CD630_12332 | hypothetical protein | 38 | | -6.4 | | 8.9x10^-18^ | | 66 | | -0.6 | | 0.7 | | 69 | | -0.4 | | 0.8 | | 71 | | -0.6 | | 0.7 | | 51 | | -0.8 | | 0.7 | |  |
| ^σ^G^ | *CD1707* | CD630_17070 | C4-dicarboxylate anaerobic carrier, DcuC family | 38 | | -4.3 | | 1.5x10^-11^ | | 38 | | -6.2 | | 4.5x10^-20^ | | 50 | | -1.5 | | 3.0x10^-4^ | | 41 | | -5.8 | | 1.0x10^-23^ | | 42 | | -1.4 | | 0.1 | |  |
| Spo0A | *pheA* | CD630_18360 | bifunctional P-protein, chorismate mutase/prephenate dehydratase | 38 | | -2.2 | | 0.03 | | 71 | | 0.2 | | 1 | | 78 | | 0.5 | | 0.8 | | 74 | | 0.2 | | 1 | | 42 | | -0.7 | | 0.9 | |  |
| σ^E^ | *CD2639* | CD630_26390 | cytotoxic factor | 37 | | -2.3 | | 4.9x10^-4^ | | 41 | | -1.9 | | 1.8x10-4 | | 38 | | -2.5 | | 2.3x10^-7^ | | 51 | | -1.1 | | 0.1 | | 37 | | -1.3 | | 0.2 | |  |
| σ^E^ | *CD1397* | CD630_13970 | hypothetical protein (VPF066 superfamily) | 37 | | -3.8 | | 5.1x10^-10^ | | 44 | | -2.3 | | 4.0x10-6 | | 40 | | -2.9 | | 1.8x10^-10^ | | 61 | | -0.8 | | 0.4 | | 47 | | -0.7 | | 0.7 | |  |
| σ^E^ | *CD3248* | CD630_32480 | polysaccharide deacetylase | 37 | | -3.0 | | 8.3x10^-8^ | | 39 | | -3.0 | | 6.3x10-9 | | 35 | | -4.6 | | 2.2x10^-16^ | | 56 | | -0.9 | | 0.2 | | 40 | | -1.2 | | 0.3 | |  |
| σ^K^ | *CD1904* | CD630_19040 | ABC transporter permease | 36 | | -5.0 | | 1.6x10^-13^ | | 41 | | -3.4 | | 7.5x10^-10^ | | 38 | | -5.8 | | 5.1x10^-16^ | | 72 | | -0.3 | | 1 | | 32 | | -3.9 | | 2.9x10^-10^ | |  |
| σ^E^ | *ssb* | CD630_32350 | single-stranded DNA-binding protein | 36 | | -4.8 | | 3.2x10^-12^ | | 41 | | -3.0 | | 4.7x10^-9^ | | 37 | | -5.4 | | 7.3x10^-21^ | | 67 | | -0.5 | | 0.8 | | 52 | | -0.4 | | 1 | |  |
| ^σ^G^(σ^E^) | *CD2868* | CD630_28680 | oxidoreductase | 36 | | -4.8 | | 1.2x10^-12^ | | 38 | | -5.0 | | 3.0x10^-17^ | | 42 | | -2.6 | | 2.2x10^-8^ | | 42 | | -4.0 | | 2.6x10^-16^ | | 43 | | -1.2 | | 0.2 | |  |
| σ^E^ | *spoIV* | CD630_24420 | stage IV sporulation protein | 36 | | -5.8 | | 5.1x10^-15^ | | 41 | | -3.1 | | 8.5x10^-10^ | | 38 | | -4.6 | | 3.3x10^-18^ | | 67 | | -0.6 | | 0.7 | | 47 | | -0.9 | | 0.6 | |  |
| ^σ^F^ | *gpr* | CD630_24700 | germination protease | 36 | | -5.7 | | 1.6x10^-3^ | | 39 | | -4.4 | | 3.5x10^-3^ | | 72 | | 0.0 | | 1 | | 51 | | -1.8 | | 0.5 | | 43 | | -1.1 | | 0.8 | |  |
| σ^K^ | *CD0196* | CD630_01960 | pseudo | 35 | | -2.7 | | 1.5x10^-4^ | | 53 | | -0.6 | | 0.8 | | 43 | | -1.4 | | 0.03 | | 128 | | 1.5 | | 0.1 | | 32 | | -2.0 | | 0.03 | |  |
| ^σ^G^ | *dacF* | CD630_12910 | D-alanyl-D-alanine carboxypeptidase | 35 | | -4.5 | | 3.4x10^-11^ | | 37 | | -4.6 | | 7.1x10^-13^ | | 76 | | 0.2 | | 1 | | 38 | | -5.5 | | 1.1x10^-14^ | | 45 | | -0.8 | | 0.8 | |  |
| Spo0A | *CD1941* | CD630_19410 | hypothetical protein | 34 | | -4.2 | | 2.9x10^-7^ | | 87 | | 0.6 | | 0.5 | | 65 | | -0.1 | | 1 | | 82 | | 0.3 | | 1 | | 51 | | -0.3 | | 1 | |  |
| Spo0A | *CD1234* | CD630_12340 | hypothetical protein | 34 | | -2.4 | | 4.3x10^-5^ | | 57 | | -0.2 | | 1 | | 57 | | -0.1 | | 1 | | 65 | | 0.0 | | 1 | | 48 | | -0.1 | | 1 | |  |
| Spo0A | *CD1232* | CD630_12320 | lipoprotein | 34 | | -2.8 | | 6.9x10^-6^ | | 58 | | -0.2 | | 1 | | 58 | | -0.2 | | 1 | | 60 | | -0.3 | | 1 | | 41 | | -0.7 | | 0.7 | |  |
| Spo0A | *vanZ* | CD630_12400 | Teicoplanin resistance protein | 34 | | -3.4 | | 1.8x10^-8^ | | 46 | | -1.3 | | 0.05 | | 53 | | -0.6 | | 0.5 | | 52 | | -1.0 | | 0.2 | | 40 | | -1.0 | | 0.6 | |  |
| σ^E^ | *spoIID* | CD630_01240 | stage II sporulation protein D | 33 | | -2.7 | | 6.4x10^-6^ | | 39 | | -1.8 | | 6.7x10^-4^ | | 35 | | -2.6 | | 7.0x10^-8^ | | 54 | | -0.6 | | 0.7 | | 38 | | -1.0 | | 0.5 | |  |
| ^σ^E^ | *cotJB1* | CD630_05970 | spore coat peptide assembly protein | 33 | | -4.9 | | 0.02 | | 37 | | -3.5 | | 0.1 | | 34 | | -6.8 | | 2.8x10^-3^ | | 78 | | 0.2 | | 1 | | 29 | | -4.8 | | 0.1 | |  |
| Spo0A | *CD0146* | CD630_01460 | hydrolase | 33 | | -2.1 | | 5.8x10^-4^ | | 56 | | 0.0 | | 1 | | 64 | | 0.4 | | 0.8 | | 55 | | -0.3 | | 1 | | 39 | | -0.5 | | 0.9 | |  |
| ^σ^G^ | *spoVT* | CD630_34990 | stage V sporulation protein T | 33 | | -5.5 | | 2.5x10^-13^ | | 36 | | -3.6 | | 3.1x10^-11^ | | 51 | | -0.9 | | 0.1 | | 38 | | -4.5 | | 4.4x10^-17^ | | 41 | | -1.0 | | 0.4 | |  |
| σ^E^ | *glpQ* | CD630_14020 | glycerophosphoryl diester phosphodiesterase | 32 | | -4.2 | | 1.4x10^-6^ | | 38 | | -2.5 | | 1.7x10^-3^ | | 35 | | -3.2 | | 2.5x10^-5^ | | 58 | | -0.5 | | 0.9 | | 40 | | -1.0 | | 0.6 | |  |
| ^σ^G^ | *CD2809* | CD630_28090 | hypothetical protein (DUF1540) | 32 | | -4.1 | | 3.6x10^-10^ | | 34 | | -4.1 | | 2.6x10^-11^ | | 62 | | -0.1 | | 1 | | 37 | | -3.5 | | 1.0x10^-8^ | | 43 | | -0.6 | | 1 | |  |
| σ^E^ | *CD3456* | CD630_34560 | 5-formyltetrahydrofolate cyclo-ligase | 32 | | -2.8 | | 1.3x10^-5^ | | 37 | | -1.9 | | 3.7x10^-4^ | | 33 | | -2.9 | | 7.0x10-9 | | 51 | | -0.6 | | 0.7 | | 38 | | -0.8 | | 0.7 | |  |
| Spo0A | *CD1233A* | CD630_12331 | hypothetical protein | 32 | | -5.1 | | 5.4x10^-14^ | | 51 | | -0.8 | | 0.4 | | 55 | | -0.5 | | 0.7 | | 59 | | -0.5 | | 0.8 | | 42 | | -0.7 | | 0.7 | |  |
| σ^E^ | *CD3638* | CD630_36380 | hypothetical protein | 32 | | -3.0 | | 1.6x10^-5^ | | 33 | | -2.9 | | 3.7x10^-8^ | | 31 | | -4.2 | | 4.6x10^-14^ | | 46 | | -1.1 | | 0.1 | | 34 | | -1.2 | | 0.2 | |  |
| Spo0A | *CD1404A* | CD630_14041 | hypothetical protein | 30 | | -3.2 | | 2.7x10^-4^ | | 52 | | -0.3 | | 1 | | 47 | | -0.5 | | 0.7 | | 63 | | 0.1 | | 1 | | 40 | | -0.5 | | 1 | |  |
| σ^E^ | *CD0629* | CD630_06290 | Crp family transcriptional regulator | 30 | | -3.1 | | 8.5x10^-6^ | | 33 | | -2.3 | | 1.5x10^-5^ | | 31 | | -3.2 | | 1.9x10^-9^ | | 45 | | -1.0 | | 0.2 | | 34 | | -1.0 | | 0.5 | |  |
| σ^F^ | *CD2266* | CD630_22660 | oxidoreductase, FAD dependent | 29 | | -3.8 | | 2.0x10^-5^ | | 36 | | -2.0 | | 7.8x10^-3^ | | 38 | | -1.6 | | 0.01 | | 45 | | -1.1 | | 0.3 | | 37 | | -0.9 | | 0.8 | |  |
| σ^E^ | *isp* | CD630_20000 | intracellular serine protease | 29 | | -5.2 | | 3.3x10^-11^ | | 34 | | -3.0 | | 1.5x10^-8^ | | 30 | | -5.6 | | 6.5x10^-19^ | | 47 | | -1.1 | | 0.1 | | 33 | | -1.4 | | 0.4 | |  |
| Spo0A | *CD0573* | CD630_05730 | membrane protein (COG4399) | 28 | | -2.4 | | 2.1x10^-4^ | | 34 | | -1.4 | | 0.02 | | 37 | | -1.0 | | 0.1 | | 41 | | -0.9 | | 0.4 | | 30 | | -1.1 | | 0.4 | |  |
| Spo0A | *eutW* | CD630_19110 | two-component sensor histidine kinase, ethanolamine specific | 28 | | -2.5 | | 9.8x10^-5^ | | 48 | | -0.2 | | 1 | | 46 | | -0.3 | | 0.9 | | 52 | | -0.1 | | 1 | | 30 | | -1.1 | | 0.3 | |  |
| σ^F^ | *fruABC* | CD630_22690 | PTS system fructose-specific transporter subunit IIABC | 28 | | -2.2 | | 9.0x10^-4^ | | 30 | | -2.1 | | 1.3x10^-4^ | | 32 | | -1.6 | | 2.1x10^-3^ | | 34 | | -1.7 | | 5.0x10^-3^ | | 32 | | -0.7 | | 0.7 | |  |
| Spo0A | *vexP3* | CD630_18750 | ABC transporter permease | 28 | | -2.7 | | 6.8x10^-5^ | | 35 | | -1.5 | | 8.8x10^-3^ | | 32 | | -1.9 | | 9.7x10^-5^ | | 63 | | 0.4 | | 1 | | 29 | | -1.3 | | 0.2 | |  |
| Spo0A | *CD1218* | CD630_12180 | glycosyl transferase family protein | 28 | | -2.7 | | 3.7x10^-5^ | | 49 | | -0.2 | | 1 | | 50 | | 0.0 | | 1 | | 52 | | -0.2 | | 1 | | 34 | | -0.7 | | 0.8 | |  |
| σ^E^ | *CD1555* | CD630_15550 | amino acid permease | 28 | | -3.5 | | 4.2x10^-7^ | | 31 | | -2.7 | | 7.2x10^-7^ | | 31 | | -2.8 | | 1.9x10^-8^ | | 38 | | -1.8 | | 4.9x10^-4^ | | 33 | | -1.0 | | 0.4 | |  |
| Spo0A | *CD1233D* | CD630_12334 | hypothetical protein | 28 | | -2.5 | | 2.5x10^-5^ | | 45 | | -0.3 | | 0.9 | | 44 | | -0.3 | | 0.8 | | 48 | | -0.3 | | 1 | | 34 | | -0.6 | | 0.9 | |  |
| σ^E^ | *pyrD* | CD630_31790 | dihydroorotate dehydrogenase, catalytic subunit | 28 | | -3.4 | | 8.1x10^-7^ | | 32 | | -2.5 | | 7.6x10^-5^ | | 30 | | -3.1 | | 7.9x10^-7^ | | 49 | | -0.5 | | 0.9 | | 41 | | -0.2 | | 1 | |  |
| σ^G^ | *CD0543* | CD630_05430 | hypothetical protein (DUF3298) | 27 | | -2.0 | | 0.04 | | 28 | | -2.2 | | 0.04 | | 42 | | -0.2 | | 1 | | 29 | | -2.6 | | 0.03 | | 29 | | -0.8 | | 0.8 | |  |
| Spo0A | *CD0572* | CD630_05720 | sporulation protein | 26 | | -3.6 | | 0.01 | | 34 | | -1.5 | | 0.3 | | 34 | | -1.5 | | 0.3 | | 38 | | -1.3 | | 0.7 | | 29 | | -1.3 | | 0.6 | |  |
| Spo0A | *CD3563* | CD630_35630 | spore cortex-lytic hydrolase | 26 | | -2.6 | | 2.5x10^-5^ | | 33 | | -1.3 | | 0.05 | | 37 | | -0.7 | | 0.3 | | 37 | | -1.1 | | 0.2 | | 35 | | -0.2 | | 1 | |  |
| σ^E^ | *acpS* | CD630_34660 | 4'-phosphopantetheinyl transferase | 26 | | -2.3 | | 6.1x10^-4^ | | 27 | | -2.3 | | 7.8x10^-5^ | | 27 | | -2.2 | | 2.3x10^-5^ | | 40 | | -0.6 | | 0.8 | | 31 | | -0.5 | | 0.9 | |  |
| σ^E^ | *CD1846* | CD630_18460 | conjugative transposon protein | 25 | | -3.1 | | 1.8x10^-3^ | | 28 | | -2.5 | | 7.7x10^-3^ | | 27 | | -2.7 | | 2.0x10^-3^ | | 54 | | 0.1 | | 1 | | 25 | | -2.0 | | 0.1 | |  |
| σ^E^ | *mviN* | CD630_27810 | transmembrane virulence factor, MviN family protein | 25 | | -2.6 | | 2.5x10^-4^ | | 26 | | -2.7 | | 1.5x10^-6^ | | 25 | | -3.0 | | 4.2x10^-8^ | | 34 | | -1.3 | | 0.1 | | 29 | | -0.8 | | 0.7 | |  |
| σ^E^ | *CD3257* | CD630_32570 | polysaccharide deacetylase | 25 | | -4.6 | | 5.3x10^-10^ | | 27 | | -3.6 | | 1.3x10^-9^ | | 26 | | -4.4 | | 6.1x10^-13^ | | 40 | | -1.1 | | 0.2 | | 32 | | -0.8 | | 0.7 | |  |
| σ^E^ | *CD1884* | CD630_18840 | hypothetical protein | 25 | | -3.9 | | 2.0x10^-7^ | | 27 | | -3.2 | | 2.1x10^-8^ | | 25 | | -6.3 | | 1.4x10^-13^ | | 42 | | -0.7 | | 0.7 | | 30 | | -1.0 | | 0.6 | |  |
| Spo0A | *nudF* | CD630_12200 | NUDIX family hydrolase | 25 | | -3.2 | | 5.1x10^-6^ | | 55 | | 0.4 | | 0.8 | | 57 | | 0.5 | | 0.5 | | 61 | | 0.5 | | 0.8 | | 36 | | -0.2 | | 1 | |  |
| σ^G^ | *CD2808* | CD630_28080 | hypothetical protein | 25 | | -4.2 | | 3.5x10^-9^ | | 25 | | -7.4 | | 2.9x10^-17^ | | 55 | | 0.3 | | 1 | | 30 | | -2.8 | | 4.4x10^-8^ | | 30 | | -1.0 | | 0.5 | |  |
| σ^E^ | *CD1741* | CD630_17410 | pseudo | 24 | | -2.6 | | 1.7x10^-4^ | | 26 | | -2.1 | | 2.9x10^-4^ | | 23 | | -3.3 | | 8.2x10^-8^ | | 39 | | -0.5 | | 0.9 | | 25 | | -1.3 | | 0.3 | |  |
| σ^E^ | *CD2440* | CD630_24400 | metal-dependent hydrolase | 24 | | -1.4 | | 0.1 | | 23 | | -1.8 | | 5.6x10^-3^ | | 21 | | -2.5 | | 2.5x10^-5^ | | 33 | | -0.5 | | 0.9 | | 23 | | -0.8 | | 0.8 | |  |
| σ^K^ | *CD2346* | CD630_23460 | membrane protein | 24 | | -2.9 | | 1.9x10^-5^ | | 29 | | -1.5 | | 0.01 | | 26 | | -2.3 | | 7.6x10^-6^ | | 53 | | 0.4 | | 1 | | 22 | | -2.1 | | 0.01 | |  |
| ^σ^G^ | *sodA* | CD630_16310 | superoxide dismutase (Mn) | 23 | | -5.7 | | 2.3x10^-6^ | | 24 | | –Inf | | 1.6x10^-9^ | | 35 | | -1.0 | | 0.4 | | 26 | | –Inf | | 6.1x10^-9^ | | 31 | | -0.7 | | 0.9 | |  |
| Spo0A | *aroB* | CD630_18330 | 3-dehydroquinate synthase | 23 | | -2.2 | | 0.01 | | 42 | | 0.1 | | 1 | | 49 | | 0.6 | | 0.6 | | 45 | | 0.1 | | 1 | | 25 | | -0.9 | | 0.6 | |  |
| σ^G^ | *CD2636* | CD630_26360 | membrane protein (YIEGIA family) | 23 | | -4.6 | | 7.2x10^-9^ | | 23 | | -6.3 | | 1.9x10^-15^ | | 32 | | -1.3 | | 0.02 | | 25 | | -5.3 | | 4.1x10^-16^ | | 27 | | -1.2 | | 0.4 | |  |
| σ^E^ | *nrdR* | CD630_26400 | NrdR family transcriptional regulator | 23 | | -2.8 | | 2.8x10^-4^ | | 23 | | -3.1 | | 4.7x10^-7^ | | 21 | | -5.2 | | 4.4x10^-13^ | | 31 | | -1.3 | | 0.1 | | 22 | | -1.7 | | 0.1 | |  |
| Spo0A | *CD1233C* | CD630_12333 | regulator | 22 | | -2.2 | | 7.8x10^-4^ | | 37 | | -0.2 | | 1 | | 36 | | -0.1 | | 1 | | 39 | | -0.2 | | 1 | | 29 | | -0.2 | | 1 | |  |
| σ^E^ | *CD1845* | CD630_18450 | membrane protein | 22 | | -4.9 | | 5.0x10^-3^ | | 26 | | -2.9 | | 0.1 | | 24 | | -4.3 | | 6.6x10^-3^ | | 46 | | -0.1 | | 1 | | 23 | | -2.1 | | 0.5 | |  |
| ^σ^F^ | *CD1132* | CD630_11320 | heavy-metal transport/detoxification protein | 22 | | -2.6 | | 8.4x10^-5^ | | 23 | | -2.8 | | 3.6x10^-6^ | | 25 | | -1.8 | | 1.2x10^-3^ | | 29 | | -1.5 | | 0.01 | | 21 | | -1.9 | | 0.02 | |  |
| Spo0A | *CD1819* | CD630_18190 | membrane protein | 22 | | -2.1 | | 0.03 | | 39 | | 0.2 | | 1 | | 37 | | 0.1 | | 1 | | 42 | | 0.2 | | 1 | | 26 | | -0.5 | | 1 | |  |
| σ^G^ | *CD2315* | CD630_23150 | hypothetical protein (PIG-L superfamily) | 21 | | -3.2 | | 5.8x10^-5^ | | 24 | | -2.3 | | 6.8x10^-5^ | | 27 | | -1.5 | | 0.01 | | 26 | | -2.2 | | 1.9x10^-4^ | | 25 | | -0.9 | | 0.7 | |  |
| Spo0A | *CD1965* | CD630_19650 | permease | 21 | | -2.0 | | 7.1x10^-3^ | | 34 | | -0.1 | | 1 | | 34 | | 0.0 | | 1 | | 37 | | -0.1 | | 1 | | 21 | | -1.1 | | 0.5 | |  |
| σ^E^ | *CD3234* | CD630_32340 | hypothetical protein (methyltransferase domain) | 21 | | -2.2 | | 5.0x10^-3^ | | 22 | | -2.3 | | 2.0x10^-4^ | | 19 | | -4.1 | | 8.2x10^-10^ | | 32 | | -0.6 | | 0.8 | | 25 | | -0.5 | | 0.9 | |  |
| Spo0A | *CD0549* | CD630_05490 | hypothetical protein (FH2 domain) | 21 | | -3.0 | | 5.6x10^-5^ | | 54 | | 0.8 | | 0.4 | | 65 | | 1.3 | | 0.1 | | 41 | | 0.0 | | 1 | | 29 | | -0.3 | | 1 | |  |
| σ^E^ | *CD0556* | CD630_05560 | sugar isomerase / endonuclease | 20 | | -2.9 | | 1.3x10^-4^ | | 27 | | -1.2 | | 0.1 | | 23 | | -2.3 | | 9.9x10^-5^ | | 33 | | -0.7 | | 0.8 | | 25 | | -0.6 | | 0.9 | |  |
| Spo0A | *CD1423* | CD630_14230 | hypothetical protein | 20 | | -2.6 | | 5.5x10^-4^ | | 50 | | 0.8 | | 0.4 | | 42 | | 0.4 | | 0.7 | | 48 | | 0.5 | | 0.9 | | 37 | | 0.5 | | 0.9 | |  |
| Spo0A | *CD0621* | CD630_06210 | membrane protein | 20 | | -5.0 | | 6.5x10^-3^ | | 36 | | -0.4 | | 1 | | 30 | | -1.1 | | 0.6 | | 38 | | -0.5 | | 1 | | 28 | | -0.5 | | 1 | |  |
| Spo0A | *eutV* | CD630_19100 | two-component response regulator, Ethanolamine specific | 20 | | -2.7 | | 6.8x10^-5^ | | 33 | | -0.4 | | 0.9 | | 32 | | -0.4 | | 0.9 | | 36 | | -0.3 | | 1 | | 24 | | -0.8 | | 0.7 | |  |
| σ^E^ | *spoIIIAC* | CD630_11940 | stage III sporulation protein AC | 20 | | -4.3 | | 3.8x10^-7^ | | 20 | | -4.4 | | 2.0x10^-10^ | | 19 | | -6.7 | | 1.8x10^-15^ | | 32 | | -0.9 | | 0.4 | | 26 | | -0.7 | | 0.8 | |  |
| σ^E^ | *CD3150A* | CD630_31501 | hypothetical protein | 19 | | -3.4 | | 1.6x10^-4^ | | 21 | | -3.1 | | 5.2x10^-4^ | | 19 | | -4.1 | | 5.7x10^-6^ | | 29 | | -1.1 | | 0.4 | | 22 | | -1.0 | | 0.8 | |  |
| ^σ^E^ | *CD24390* | CD630_24390 | diacylglycerol kinase | 19 | | -1.4 | | 0.2 | | 19 | | -1.6 | | 0.03 | | 17 | | -2.1 | | 2.2x10^-3^ | | 29 | | -0.2 | | 1 | | 19 | | -0.7 | | 0.9 | |  |
| σ^E^ | *CD2641* | CD630_26410 | sporulation protein | 19 | | -3.3 | | 5.2x10^-3^ | | 20 | | -3.3 | | 8.7x10^-4^ | | 18 | | -5.5 | | 4.7x10^-6^ | | 26 | | -1.6 | | 0.3 | | 19 | | -1.9 | | 0.3 | |  |
| σ^E^ | *CD1301* | CD630_13010 | membrane protein (TP0381 superfamily) | 19 | | -3.7 | | 2.5x10^-3^ | | 20 | | -3.2 | | 3.3x10^-3^ | | 19 | | -4.3 | | 7.7x10^-5^ | | 30 | | -0.9 | | 0.7 | | 20 | | -1.5 | | 0.5 | |  |
| σ^E^ | *gltC* | CD630_17460 | Sodium/glutamate symporter | 18 | | -1.8 | | 0.05 | | 20 | | -1.8 | | 0.01 | | 17 | | -2.6 | | 1.3x10^-4^ | | 27 | | -0.5 | | 0.9 | | 25 | | 0.0 | | 1 | |  |
| σ^F^ | *CD3180* | CD630_31800 | purine permease | 18 | | -3.1 | | 0.03 | | 19 | | -3.2 | | 0.03 | | 21 | | -2.3 | | 0.1 | | 30 | | -0.6 | | 1 | | 26 | | -0.2 | | 1 | |  |
| σ^E^ | *CD1724* | CD630_17240 | hypothetical protein (DUF3795) | 18 | | -4.1 | | 4.2x10^-4^ | | 20 | | -2.9 | | 3.2x10^-3^ | | 18 | | -4.0 | | 4.1x10^-4^ | | 28 | | -1.0 | | 0.8 | | 20 | | -1.4 | | 0.6 | |  |
| Spo0A | *CD2211* | CD630_22110 | ABC transporter multidrug-family ATP-binding/permease | 18 | | -2.1 | | 0.02 | | 40 | | 0.8 | | 0.9 | | 36 | | 0.5 | | 0.8 | | 36 | | 0.3 | | 1 | | 22 | | -0.3 | | 1 | |  |
| Spo0A | *CD0571* | CD630_05710 | hypothetical protein | 17 | | -2.3 | | 5.8x10^-3^ | | 23 | | -1.0 | | 0.4 | | 24 | | -0.8 | | 0.5 | | 28 | | -0.4 | | 1 | | 22 | | -0.3 | | 1 | |  |
| σ^E^ | *spoIIIAD* | CD630_11950 | stage III sporulation protein AD | 17 | | -4.4 | | 1.4x10^-6^ | | 18 | | -5.4 | | 1.6x10^-11^ | | 18 | | -4.9 | | 1.5x10^-11^ | | 29 | | -0.9 | | 0.4 | | 22 | | -0.9 | | 0.7 | |  |
| ^σ^G^ | *CD2841* | CD630_28410 | amidohydrolase | 17 | | -3.5 | | 1.1x10^-4^ | | 18 | | -3.4 | | 7.1x10^-7^ | | 27 | | -0.7 | | 0.5 | | 20 | | -3.1 | | 1.6x10^-6^ | | 18 | | -1.7 | | 0.2 | |  |
| σ^E^ | *CD1124A* | CD630_11241 | hypothetical protein | 17 | | -1.5 | | 0.1 | | 17 | | -1.9 | | 0.01 | | 16 | | -2.0 | | 3.0x10^-3^ | | 22 | | -0.9 | | 0.6 | | 18 | | -0.6 | | 0.9 | |  |
| σ^E^ | *CD3637* | CD630_36370 | NADPH-dependent FMN reductase | 17 | | -1.9 | | 0.1 | | 16 | | -3.0 | | 0.02 | | 15 | | -3.6 | | 8.6x10^-3^ | | 23 | | -0.9 | | 0.9 | | 18 | | -0.8 | | 0.9 | |  |
| σ^E^ | *CD2638* | CD630_26380 | two-component response regulator | 17 | | -2.3 | | 7.4x10^-3^ | | 18 | | -2.0 | | 3.1x10^-3^ | | 17 | | -2.2 | | 1.3x10^-3^ | | 22 | | -1.2 | | 0.2 | | 15 | | -1.9 | | 0.1 | |  |
| σ^F^ | *CD0906* | CD630_09060 | DNA-binding protein | 17 | | -1.1 | | 0.5 | | 13 | | -3.1 | | 0.03 | | 14 | | -2.6 | | 0.1 | | 19 | | -1.1 | | 0.9 | | 21 | | 0.2 | | 1 | |  |
| σ^E^ | *CD1844A* | CD630_18441 | pseudo | 16 | | -6.5 | | 7.4x10^-7^ | | 20 | | -2.5 | | 4.9x10^-3^ | | 17 | | -4.4 | | 3.7x10^-5^ | | 35 | | 0.0 | | 1 | | 16 | | -2.4 | | 0.06 | |  |
| σ^F^ | *CD2685* | CD630_26850 | sporulation stage II, protein E | 16 | | -0.6 | | 0.8 | | 13 | | -2.0 | | 0.02 | | 15 | | -1.1 | | 0.3 | | 15 | | -1.5 | | 0.2 | | 12 | | -1.2 | | 0.6 | |  |
| σ^E^ | *CD1575* | CD630_15750 | hypothetical protein (DUF348, COG3584) | 16 | | -3.7 | | 2.4x10^-5^ | | 19 | | -2.0 | | 3.3x10^-3^ | | 16 | | -3.8 | | 4.9x10^-8^ | | 30 | | -0.2 | | 1 | | 20 | | -0.8 | | 0.8 | |  |
| Spo0A | *spoIIR* | CD630_35640 | pro-σ^E^ endopeptidase (stage II sporulation) | 15 | | -2.0 | | 0.01 | | 18 | | -1.1 | | 0.5 | | 20 | | -0.7 | | 0.5 | | 19 | | -1.2 | | 0.5 | | 20 | | 0.0 | | 1 | |  |
| Spo0A | *CD1966* | CD630_19660 | acyl-CoA thioesterase | 15 | | -4.1 | | 4.6x10^-3^ | | 31 | | 0.1 | | 1 | | 28 | | -0.1 | | 1 | | 26 | | -0.7 | | 1 | | 17 | | -1.2 | | 0.6 | |  |
| σ^G^ | *CD1354* | CD630_13540 | hypothetical protein | 14 | | -3.3 | | 6.1x10^-4^ | | 14 | | -5.0 | | 3.1x10^-9^ | | 29 | | 0.2 | | 1 | | 15 | | -4.3 | | 1.0x10^-8^ | | 19 | | -0.5 | | 1 | |  |
| σ^K^ | *CD0902* | CD630_09020 | cation efflux protein | 14 | | -3.6 | | 2.1x10^-3^ | | 15 | | -3.3 | | 2.0x10^-3^ | | 13 | | -7.0 | | 3.8x10^-6^ | | 36 | | 0.6 | | 0.9 | | 12 | | -3.8 | | 7.2x10^-3^ | |  |
| σ^F^ | *CD1031* | CD630_10310 | cell wall anchored protein | 14 | | -1.8 | | 0.1 | | 13 | | -2.4 | | 1.9x10^-3^ | | 15 | | -1.6 | | 0.03 | | 15 | | -1.9 | | 0.02 | | 14 | | -1.0 | | 0.7 | |  |
| σ^E^ | *CD2427* | CD630_24270 | flavodoxin/ferredoxin oxidoreductase subunit gamma | 14 | | -1.8 | | 0.05 | | 16 | | -1.3 | | 0.3 | | 14 | | -2.0 | | 0.03 | | 19 | | -0.7 | | 0.9 | | 16 | | -0.4 | | 1 | |  |
| ^σ^G^ | *CD24310* | CD630_24310 | nitrite/sulfite reductase | 13 | | -3.8 | | 1.6x10^-4^ | | 15 | | -2.4 | | 1.2x10^-3^ | | 18 | | -1.2 | | 0.2 | | 16 | | -2.3 | | 1.4x10^-3^ | | 17 | | -0.6 | | 0.9 | |  |
| σ^E^ | *CD2316* | CD630_23160 | two-component response regulator | 13 | | -3.1 | | 1.8x10^-3^ | | 16 | | -1.7 | | 0.03 | | 15 | | -2.2 | | 3.2x10^-3^ | | 20 | | -0.9 | | 0.6 | | 15 | | -0.8 | | 0.8 | |  |
| σ^E^ | *CD2445* | CD630_24450 | transmembrane signaling protein,TspO/MBR family | 13 | | -2.2 | | 0.03 | | 16 | | -1.1 | | 0.4 | | 13 | | -2.6 | | 6.4x10^-4^ | | 24 | | 0.0 | | 1 | | 16 | | -0.3 | | 1 | |  |
| Spo0A | *CD1678* | CD630_16780 | membrane protein (DUF969) | 13 | | -3.3 | | 4.4x10^-3^ | | 16 | | -1.9 | | 0.1 | | 16 | | -1.5 | | 0.2 | | 16 | | -2.0 | | 0.1 | | 18 | | -0.4 | | 1 | |  |
| Spo0A | *CD2142* | CD630_21420 | transporter | 13 | | -3.1 | | 0.02 | | 15 | | -1.8 | | 0.2 | | 17 | | -1.2 | | 0.3 | | 19 | | -0.9 | | 0.8 | | 14 | | -1.1 | | 0.8 | |  |
| σ^G^ | *CD1028* | CD630_10280 | signaling protein | 12 | | -2.8 | | 3.6x10^-3^ | | 17 | | -1.1 | | 0.3 | | 19 | | -0.6 | | 0.9 | | 14 | | -2.6 | | 1.5x10^-3^ | | 23 | | 0.5 | | 0.9 | |  |
| σ^E^ | *CD1726* | CD630_17260 | hypothetical protein | 12 | | -7.1 | | 8.5x10^-8^ | | 15 | | -2.6 | | 8.3x10^-4^ | | 13 | | -4.5 | | 1.1x10^-5^ | | 20 | | -1.0 | | 0.5 | | 16 | | -1.0 | | 0.7 | |  |
| σ^E^ | *CD0131* | CD630_01310 | membrane protein | 12 | | -3.4 | | 1.8x10^-3^ | | 13 | | -3.0 | | 1.2x10^-4^ | | 12 | | -5.3 | | 1.1x10^-8^ | | 18 | | -1.1 | | 0.4 | | 12 | | -1.8 | | 0.2 | |  |
| Spo0A | *CD1239* | CD630_12390 | beta-lactams repressor | 12 | | -2.9 | | 2.5x10^-3^ | | 15 | | -1.3 | | 0.2 | | 17 | | -0.8 | | 0.4 | | 17 | | -1.1 | | 0.5 | | 12 | | -1.8 | | 0.2 | |  |
| σ^G^ | *CD3551A* | CD630_35511 | membrane protein (DUF37) [[6](#_ENREF_6)] | 12 | | -4.2 | | 0.04 | | 12 | | -5.6 | | 4.7x10^-3^ | | 16 | | -1.4 | | 0.6 | | 13 | | -5.6 | | 0.01 | | 14 | | -1.3 | | 0.8 | |  |
| σ^E^ | *CD1929* | CD630_19290 | membrane protein | 12 | | -4.4 | | 5.2x10^-5^ | | 13 | | -3.5 | | 1.1x10^-5^ | | 13 | | -3.5 | | 1.1x10^-6^ | | 19 | | -0.9 | | 0.7 | | 14 | | -1.1 | | 0.6 | |  |
| Spo0A | *CD2657* | CD630_26570 | hypothetical protein | 12 | | -3.2 | | 1.5x10^-3^ | | 21 | | -0.1 | | 1 | | 16 | | -1.1 | | 0.3 | | 25 | | 0.1 | | 1 | | 14 | | -0.9 | | 0.8 | |  |
| Spo0A | *mnaA* | CD630_10330 | UDP-N-acetylglucosamine 2-epimerase | 11 | | -2.5 | | 5.8x10^-3^ | | 14 | | -1.5 | | 0.1 | | 13 | | -1.6 | | 0.1 | | 18 | | -0.6 | | 0.9 | | 13 | | -0.9 | | 0.8 | |  |
| σ^F^ | *CD1297* | CD630_12970 | hypothetical protein (DUF2953) | 11 | | -3.4 | | 9.6x10^-4^ | | 11 | | -5.3 | | 6.6x10^-8^ | | 19 | | -0.3 | | 1 | | 15 | | -1.7 | | 0.05 | | 14 | | -0.6 | | 0.9 | |  |
| σ^G^ | *CD1298* | CD630_12980 | hypothetical protein (YtfJ sporulation protein [[7](#_ENREF_8)]) | 11 | | -3.0 | | 3.4x10^-3^ | | 11 | | -6.2 | | 2.1x10^-8^ | | 22 | | 0.2 | | 1 | | 14 | | -2.2 | | 8.8x10^-3^ | | 13 | | -1.0 | | 0.8 | |  |
| σ^E^ | *CD1185* | CD630_11850 | diguanylate kinase signaling protein | 11 | | -2.5 | | 0.02 | | 12 | | -2.0 | | 0.02 | | 11 | | -2.6 | | 2.3x10^-3^ | | 16 | | -1.0 | | 0.7 | | 10 | | -2.0 | | 0.2 | |  |
| ^σ^G^(σ^E^) | *CD2598* | CD630_25980 | oligosaccharide deacetylase | 11 | | -3.2 | | 1.8x10^-3^ | | 11 | | -5.2 | | 9.2x10^-8^ | | 12 | | -2.4 | | 5.0x10^-4^ | | 12 | | -3.9 | | 3.0x10^-6^ | | 13 | | -1.0 | | 0.7 | |  |
| σ^F^ | *CD2377* | CD630_23770 | NUDIX family hydrolase | 11 | | -1.8 | | 0.2 | | 10 | | -2.4 | | 5.6x10^-3^ | | 11 | | -1.9 | | 0.04 | | 14 | | -1.0 | | 0.6 | | 10 | | -1.2 | | 0.6 | |  |
| ^σ^G^ | *CD1595A* | CD630_15951 | ferredoxin | 11 | | -2.9 | | 3.8x10^-3^ | | 12 | | -2.3 | | 4.4x10^-3^ | | 15 | | -1.1 | | 0.3 | | 13 | | -2.4 | | 6.0x10^-3^ | | 15 | | -0.3 | | 1 | |  |
| Spo0A | *CD1170* | CD630_11700 | hypothetical protein | 11 | | -2.3 | | 0.03 | | 17 | | -0.2 | | 1 | | 13 | | -1.3 | | 0.2 | | 16 | | -0.7 | | 0.9 | | 14 | | -0.2 | | 1 | |  |
| σ^K^ | *CD2409* | CD630_24090 | hypothetical protein | 11 | | -3.8 | | 8.5x10^-4^ | | 14 | | -1.7 | | 0.1 | | 14 | | -1.6 | | 0.03 | | 24 | | 0.2 | | 1 | | 10 | | -2.7 | | 0.02 | |  |
| σ^E^ | *CD3635* | CD630_36350 | hypothetical protein | 10 | | -3.5 | | 0.1 | | 11 | | -3.9 | | 0.04 | | 11 | | -3.9 | | 0.03 | | 17 | | -0.9 | | 1 | | 12 | | -1.3 | | 0.9 | |  |
| σ^E^ | *cwlD* | CD630_01060 | Germination-specific N-acetylmuramoyl-L-alanine amidase, Autolysin | 10 | | -4.9 | | 4.1x10^-5^ | | 13 | | -2.3 | | 4.2x10^-3^ | | 11 | | -4.2 | | 3.0x10^-6^ | | 18 | | -0.8 | | 0.8 | | 12 | | -1.1 | | 0.7 | |  |
| ^σ^G^(σ^E^) | *CD0214* | CD630_02140 | hypothetical protein | 10 | | -3.3 | | 1.6x10^-3^ | | 11 | | -2.9 | | 7.3x10^-4^ | | 10 | | -3.6 | | 2.7x10^-5^ | | 11 | | -3.1 | | 3.9x10^-4^ | | 11 | | -1.4 | | 0.6 | |  |
| σ^E^ | *ddl* | CD630_14080 | D-Ala-D-Ala ligase | 9 | | -2.3 | | 0.04 | | 11 | | -1.5 | | 0.2 | | 10 | | -2.0 | | 0.03 | | 16 | | -0.2 | | 1 | | 9 | | -1.4 | | 0.6 | |  |
| ^σ^E^ | *CD2865* | CD630_28650 | bacterioferritin | 9 | | -2.9 | | 0.03 | | 11 | | -1.6 | | 0.3 | | 10 | | -2.7 | | 0.02 | | 16 | | -0.3 | | 1 | | 12 | | -0.5 | | 1 | |  |
| Spo0A | *CD1045* | CD630_10450 | sporulation integral membrane protein | 9 | | -3.0 | | 4.5x10^-3^ | | 11 | | -1.8 | | 0.1 | | 11 | | -1.6 | | 0.1 | | 13 | | -1.2 | | 0.5 | | 10 | | -1.0 | | 0.8 | |  |
| σ^E^ | *spmA* | CD630_35420 | spore maturation protein A | 8 | | -3.5 | | 7.6x10^-3^ | | 9 | | -3.6 | | 1.7x10^-4^ | | 8 | | –Inf | | 1.1x10^-7^ | | 14 | | -0.8 | | 0.8 | | 9 | | -1.4 | | 0.6 | |  |
| σ^F^ | *CD2863* | CD630_28630 | sigma-54 dependent transcriptional regulator | 8 | | -1.5 | | 0.4 | | 7 | | -2.8 | | 9.3x10^-3^ | | 8 | | -1.7 | | 0.2 | | 10 | | -1.1 | | 0.7 | | 8 | | -1.1 | | 0.8 | |  |
| σ^E^ | *CD1063* | CD630_10630 | hypothetical protein | 8 | | -4.5 | | 0.01 | | 10 | | -2.0 | | 0.2 | | 8 | | –Inf | | 5.0x10^-4^ | | 17 | | -0.1 | | 1 | | 9 | | -1.8 | | 0.7 | |  |
| ^σ^F^ | *CD2245A* | CD630_22451 | hypothetical protein (Yqz-like) | 8 | | -6.5 | | 2.2x10^-5^ | | 8 | | -5.9 | | 1.7x10^-5^ | | 11 | | -1.5 | | 0.1 | | 11 | | -2.3 | | 0.1 | | 10 | | -1.3 | | 0.7 | |  |
| σ^G^ | *fruK* | CD630_22700 | fructose 1-phosphate kinase | 8 | | -1.9 | | 0.2 | | 7 | | -3.2 | | 2.7x10^-3^ | | 9 | | -1.4 | | 0.2 | | 8 | | -2.5 | | 0.04 | | 7 | | -2.0 | | 0.3 | |  |
| σ^F^ | *spoIIP* | CD630_24690 | stage II sporulation protein P | 7 | | -2.4 | | 0.1 | | 8 | | -2.2 | | 0.03 | | 14 | | 0.1 | | 1 | | 11 | | -1.0 | | 0.7 | | 9 | | -0.5 | | 1 | |  |
| σ^E^ | *CD3368* | CD630_33680 | ribosome biogenesis GTPase RsgA; putative EngC-like GTPase | 7 | | -2.9 | | 0.05 | | 8 | | -2.2 | | 0.1 | | 8 | | -3.2 | | 0.02 | | 15 | | 0.0 | | 1 | | 6 | | -2.7 | | 0.1 | |  |
| σ^E^ | *CD2055* | CD630_20550 | hypothetical protein | 7 | | -3.7 | | 0.02 | | 10 | | -1.2 | | 0.5 | | 8 | | -3.1 | | 0.01 | | 28 | | 1.4 | | 0.2 | | 6 | | -2.7 | | 0.1 | |  |
| σ^E^ | *fliI* | CD630_02510 | ATP synthase subunit beta FliI | 7 | | -0.3 | | 1 | | 6 | | -1.4 | | 0.6 | | 4 | | -4.3 | | 0.01 | | 6 | | -1.7 | | 0.5 | | 5 | | -1.1 | | 0.9 | |  |
| σ^E^ | *CD3636* | CD630_36360 | membrane protein | 7 | | -3.3 | | 0.05 | | 7 | | -4.5 | | 2.7x10^-3^ | | 6 | | -5.9 | | 1.0x10^-3^ | | 10 | | -1.0 | | 0.8 | | 6 | | -2.0 | | 0.4 | |  |
| Spo0A | *CD0620* | CD630_06200 | hypothetical protein | 6 | | -5.1 | | 1.6x10^-3^ | | 11 | | -0.3 | | 1 | | 9 | | -1.4 | | 0.4 | | 10 | | -1.0 | | 0.8 | | 8 | | -0.8 | | 1 | |  |
| σ^E^ | *CD2395* | CD630_23950 | hypothetical protein | 6 | | -1.7 | | 0.5 | | 5 | | -4.2 | | 5.5x10^-3^ | | 5 | | –Inf | | 3.7x10^-4^ | | 7 | | -1.2 | | 0.7 | | 5 | | -2.7 | | 0.3 | |  |
| σ^E^ | *CD1189* | CD630_11890 | amino acid/polyamine transporter I | 6 | | -1.2 | | 0.7 | | 5 | | -2.5 | | 0.2 | | 5 | | -3.7 | | 0.03 | | 6 | | -1.9 | | 0.6 | | 5 | | -1.8 | | 0.6 | |  |
| σ^G^ | *CD0793* | CD630_07930 | hypothetical protein | 6 | | -4.3 | | 3.6x10^-3^ | | 6 | | -5.6 | | 1.6x10^-4^ | | 7 | | -1.8 | | 0.1 | | 7 | | -3.5 | | 4.7x10^-3^ | | 6 | | -2.3 | | 0.3 | |  |
| ^σ^G^ | *CD2599* | CD630_25990 | transcriptional regulator | 5 | | -3.4 | | 0.04 | | 5 | | -4.1 | | 2.7x10^-3^ | | 7 | | -1.7 | | 0.2 | | 6 | | -3.0 | | 0.03 | | 6 | | -1.0 | | 0.9 | |  |
| σ^E^ | *CD3151* | CD630_31510 | hypothetical protein | 5 | | -1.6 | | 0.7 | | 5 | | -2.6 | | 0.1 | | 4 | | -5.3 | | 4.7x10^-3^ | | 6 | | -1.5 | | 0.7 | | 5 | | -1.3 | | 0.8 | |  |
| σ^E^ | *CD0213* | CD630_02130 | spore coat protein | 5 | | -3.4 | | 0.05 | | 6 | | -2.1 | | 0.3 | | 5 | | -3.6 | | 0.04 | | 6 | | -1.9 | | 0.5 | | 6 | | -1.1 | | 0.9 | |  |
| σ^G^ | *spoVAC* | CD630_07730 | stage V sporulation protein AC | 4 | | -4.0 | | 0.05 | | 4 | | -5.2 | | 8.8x10^-3^ | | 6 | | -1.0 | | 0.7 | | 5 | | -5.5 | | 0.02 | | 4 | | -1.9 | | 0.6 | |  |
| σ^E^ | *CD2687* | CD630_26870 | hypothetical protein | 4 | | -1.6 | | 0.6 | | 3 | | –Inf | | 2.8x10^-3^ | | 5 | | -0.7 | | 1 | | 5 | | -1.8 | | 0.5 | | 5 | | -0.3 | | 1 | |  |
| Spo0A | *CD0619* | CD630_06190 | hypothetical protein | 4 | | -5.5 | | 0.01 | | 8 | | 0.0 | | 1 | | 6 | | -1.3 | | 0.6 | | 9 | | 0.0 | | 1 | | 8 | | 0.3 | | 1 | |  |
| σ^E^ | *CD3636A* | CD630_36361 | hypothetical protein | 4 | | -2.4 | | 0.3 | | 4 | | -4.9 | | 0.01 | | 4 | | -3.5 | | 0.04 | | 5 | | -1.8 | | 0.6 | | 4 | | -2.1 | | 0.6 | |  |
| σ^F^ | *CD1290* | CD630_12900 | small acid-soluble spore protein SASP | 3 | | -4.1 | | 0.1 | | 3 | | –Inf | | 6.5x10^-3^ | | 5 | | -1.0 | | 0.7 | | 4 | | -2.5 | | 0.2 | | 4 | | -1.3 | | 0.9 | |  |
| σ^E^ | *CD1926* | CD630_19260 | pseudo | 3 | | -4.1 | | 0.1 | | 3 | | -4.4 | | 0.1 | | 3 | | –Inf | | 0.02 | | 5 | | -1.2 | | 0.9 | | 3 | | -1.6 | | 0.8 | |  |
| ^σ^E^ | *CD2634* | CD630_26340 | hypothetical protein | 3 | | -2.6 | | 0.4 | | 2 | | –Inf | | 0.04 | | 3 | | -1.2 | | 0.8 | | 3 | | -2.8 | | 0.5 | | 3 | | -1.3 | | 1 | |  |

^†^ Two factors are listed in the table for genes whose expression was dependent on both σ^E^ and σ^G^ (adjusted p-value ≤ 0.05, log_2_FC ≤ -2). *Dep.* indicates the most downstream sigma factor on which gene expression depends upon. *BM* refers to base mean, the mean of the counts after they were divided by the size factors to adjust for different sequencing depths. This value is the mean for the sample relative to wild type. *log_2_FC* denotes log_2_fold-change. A negative value indicates that the gene was downregulated relative to wild type. ^ Indicates that gene product was detected in Lawley *et al*. proteomic analysis of purified spores [[8](#_ENREF_7)]. *–Inf* indicates that no transcript was detected in the mutant relative to wild type. See Text S2 for the references.
