# Supplementary material for: Global Analysis of the Sporulation Pathway of Clostridium difficile
Source: PLoS Genet. 2013 Aug 8;9(8):e1003660. doi: 10.1371/journal.pgen.1003660 (PMC3738446; doi:10.1371/journal.pgen.1003660)
Supplement: Table S10 — Genes encoding hypothetical proteins induced during sporulation. † Two factors are listed in the table for genes whose expression was dependent on both σE and σG (adjusted p-value≤0.05, log2FC≤−2). Dep. indicates the most downstream sigma factor on which gene expression depends upon. BM refers to base mean, the mean of the counts after they were divided by the size factors to adjust for different sequencing depths. This value is the mean for the sample relative to wild type. log2FC denotes log2fold-change. A negative value indicates that the gene was downregulated relative to wild type. ∧ Indicates that gene product was detected in Lawley et al. proteomic analysis of purified spores [70]. −Inf indicates that no transcript was detected in the mutant relative to wild type. See Text S2 for the references. (DOCX) [file pgen.1003660.s017.docx]

**Table S10. Genes encoding hypothetical proteins induced during sporulation.**

|  |  |  |  | **Spo0A** | | | **σ^F^** | | | **σ^E^** | | | **σ^G^** | | | **σ^K^** | | |
| --- | --- | --- | --- | --- | --- | --- | --- | --- | --- | --- | --- | --- | --- | --- | --- | --- | --- | --- |
| **Dep.^†^** | **Name** | **locus_tag** | **description** | **BM** | **log_2_FC** | **adjP** | **BM** | **log_2_FC** | **adjP** | **BM** | **log_2_FC** | **adjP** | **BM** | **log_2_FC** | **adjP** | **BM** | **log_2_FC** | **adjP** |
| σ^E^ | *CD0129* | CD630_01290 | hypothetical protein (YyaC, in sporulating bacteria) | 105 | -5.1 | 8.9x10^-23^ | 126 | -2.5 | 6.7x10^-10^ | 109 | -5.1 | 3.0x10^-33^ | 198 | -0.5 | 0.8 | 148 | -0.5 | 0.9 |
| ^σ^G^(σ^E^) | *CD0214* | CD630_02140 | hypothetical protein | 10 | -3.3 | 1.6x10^-3^ | 11 | -2.9 | 7.3x10^-4^ | 10 | -3.6 | 2.7x10^-5^ | 11 | -3.1 | 3.9x10^-4^ | 11 | -1.4 | 0.6 |
| σ^E^ | *CD0296* | CD630_02960 | hypothetical protein | 43 | -2.5 | 1.3x10^-5^ | 51 | -1.6 | 2.0x10^-3^ | 44 | -2.8 | 2.5x10^-5^ | 81 | -0.1 | 1 | 44 | -1.3 | 0.2 |
| σ^E^ | *CD0311* | CD630_03110 | hypothetical protein | 926 | -6.8 | 2.3x10^-46^ | 1086 | -3.0 | 5.2x10^-16^ | 966 | -6.4 | 3.1x10^-68^ | 1715 | -0.6 | 0.4 | 1077 | -1.4 | 4.4x10^-3^ |
| σ^G^ | *CD0543* | CD630_05430 | hypothetical protein (DUF3298) | 27 | -2.0 | 0.04 | 28 | -2.2 | 0.04 | 42 | -0.2 | 1 | 29 | -2.6 | 0.03 | 29 | -0.8 | 0.8 |
| Spo0A | *CD0549* | CD630_05490 | hypothetical protein (FH2 domain) | 21 | -3.0 | 5.6x10^-5^ | 54 | 0.8 | 0.4 | 65 | 1.3 | 0.1 | 41 | 0.0 | 1 | 29 | -0.3 | 1 |
| Spo0A | *CD0571* | CD630_05710 | hypothetical protein | 17 | -2.3 | 5.8x10^-3^ | 23 | -1.0 | 0.4 | 24 | -0.8 | 0.5 | 28 | -0.4 | 1 | 22 | -0.3 | 1 |
| ^σ^K^ | *CD0596* | CD630_05960 | hypothetical protein (CotJA homolog) | 67 | -7.0 | 1.2x10^-5^ | 75 | -3.7 | 4.7x10^-3^ | 69 | -7.5 | 7.3x10^-7^ | 159 | 0.2 | 1 | 58 | -6.1 | 1.4x10^-4^ |
| Spo0A | *CD0619* | CD630_06190 | hypothetical protein | 4 | -5.5 | 0.01 | 8 | 0.0 | 1 | 6 | -1.3 | 0.6 | 9 | 0.0 | 1 | 8 | 0.3 | 1 |
| Spo0A | *CD0620* | CD630_06200 | hypothetical protein | 6 | -5.1 | 1.6x10^-3^ | 11 | -0.3 | 1 | 9 | -1.4 | 0.4 | 10 | -1.0 | 0.8 | 8 | -0.8 | 1 |
| Spo0A | *CD0622* | CD630_06220 | hypothetical protein (DUF1629) | 113 | -7.5 | 2.1x10^-10^ | 192 | -0.7 | 0.7 | 167 | -1.2 | 0.2 | 210 | -0.6 | 0.9 | 171 | -0.4 | 1 |
| σ^G^ | *CD0793* | CD630_07930 | hypothetical protein | 6 | -4.3 | 3.6x10^-3^ | 6 | -5.6 | 1.6x10^-4^ | 7 | -1.8 | 0.1 | 7 | -3.5 | 4.7x10^-3^ | 6 | -2.3 | 0.3 |
| σ^K^ | *CD0896* | CD630_08960 | hypothetical protein | 52 | -6.8 | 5.7x10^-6^ | 59 | -3.6 | 4.5x10^-3^ | 57 | -4.4 | 6.4x10^-4^ | 100 | -0.5 | 1 | 46 | -5.6 | 4.8x10^-4^ |
| σ^E^ | *CD1063* | CD630_10630 | hypothetical protein | 8 | -4.5 | 0.01 | 10 | -2.0 | 0.2 | 8 | –Inf | 5.0x10^-4^ | 17 | -0.1 | 1 | 9 | -1.8 | 0.7 |
| ^σ^K^ | *CD1063A* | CD630_10631 | hypothetical protein | 71 | -7.7 | 6.8x10^-9^ | 86 | -2.7 | 3.3x10^-3^ | 74 | -8.3 | 3.1x10^-10^ | 167 | 0.1 | 1 | 63 | -4.8 | 2.5x10^-5^ |
| σ^K^ | *CD1063B* | CD630_10632 | hypothetical protein | 1496 | -7.5 | 4.7x10^-27^ | 1802 | -2.7 | 4.7x10^-7^ | 1569 | -6.2 | 3.8x10^-21^ | 3044 | -0.3 | 1 | 1317 | -5.1 | 2.2x10^-15^ |
| σ^K^ | *CD1063C* | CD630_10633 | hypothetical protein | 1552 | -7.5 | 4.2x10^-27^ | 1879 | -2.7 | 5.8x10^-7^ | 1632 | -6.0 | 2.9x10^-20^ | 3104 | -0.4 | 0.9 | 1369 | -4.9 | 3.4x10^-15^ |
| σ^K^ | *CD1065* | CD630_10650 | hypothetical protein | 411 | -7.6 | 8.1x10^-32^ | 495 | -2.7 | 2.3x10^-10^ | 429 | -7.3 | 6.5x10^-33^ | 769 | -0.6 | 0.5 | 394 | -3.0 | 3.0x10^-11^ |
| σ^E^ | *CD1066* | CD630_10660 | hypothetical protein | 91 | -4.0 | 1.8x10^-15^ | 105 | -2.7 | 2.4x10^-10^ | 95 | -4.1 | 4.6x10^-24^ | 149 | -0.9 | 0.1 | 96 | -1.7 | 2.3x10^-3^ |
| ^σ^K^ | *CD1067* | CD630_10670 | hypothetical protein | 9888 | -7.6 | 1.3x10^-60^ | 11923 | -2.7 | 3.9x10^-14^ | 10380 | -6.1 | 1.5x10^-50^ | 21003 | -0.2 | 1 | 8776 | -4.7 | 2.4x10^-32^ |
| σ^E^ | *CD1124A* | CD630_11241 | hypothetical protein | 17 | -1.5 | 0.1 | 17 | -1.9 | 0.01 | 16 | -2.0 | 3.0x10^-3^ | 22 | -0.9 | 0.6 | 18 | -0.6 | 0.9 |
| ^σ^K^ | *CD1133* | CD630_11330 | hypothetical protein | 96 | -4.9 | 8.8x10^-21^ | 119 | -2.2 | 7.1x10^-7^ | 101 | -4.5 | 1.7x10^-18^ | 255 | 0.5 | 0.6 | 86 | -3.6 | 9.0x10^-13^ |
| Spo0A | *CD1170* | CD630_11700 | hypothetical protein | 11 | -2.3 | 0.03 | 17 | -0.2 | 1 | 13 | -1.3 | 0.2 | 16 | -0.7 | 0.9 | 14 | -0.2 | 1 |
| Spo0A | *CD1219* | CD630_12190 | hypothetical protein | 48 | -2.5 | 4.7x10^-6^ | 83 | 0.0 | 1 | 83 | 0.0 | 1 | 85 | -0.2 | 1 | 59 | -0.5 | 0.9 |
| Spo0A | *CD1233A* | CD630_12331 | hypothetical protein | 32 | -5.1 | 5.4x10^-14^ | 51 | -0.8 | 0.4 | 55 | -0.5 | 0.7 | 59 | -0.5 | 0.8 | 42 | -0.7 | 0.7 |
| Spo0A | *CD1233B* | CD630_12332 | hypothetical protein | 38 | -6.4 | 8.9x10^-18^ | 66 | -0.6 | 0.7 | 69 | -0.4 | 0.8 | 71 | -0.6 | 0.7 | 51 | -0.8 | 0.7 |
| Spo0A | *CD1233D* | CD630_12334 | hypothetical protein | 28 | -2.5 | 2.5x10^-5^ | 45 | -0.3 | 0.9 | 44 | -0.3 | 0.8 | 48 | -0.3 | 1 | 34 | -0.6 | 0.9 |
| Spo0A | *CD1234* | CD630_12340 | hypothetical protein | 34 | -2.4 | 4.3x10^-5^ | 57 | -0.2 | 1 | 57 | -0.1 | 1 | 65 | 0.0 | 1 | 48 | -0.1 | 1 |
| Spo0A | *CD1235* | CD630_12350 | hypothetical protein | 45 | -2.8 | 4.3x10^-3^ | 87 | 0.2 | 1 | 80 | 0.0 | 1 | 98 | 0.3 | 1 | 60 | -0.3 | 1 |
| σ^F^ | *CD1297* | CD630_12970 | hypothetical protein (DUF2953) | 11 | -3.4 | 9.6x10^-4^ | 11 | -5.3 | 6.6x10^-8^ | 19 | -0.3 | 1 | 15 | -1.7 | 0.05 | 14 | -0.6 | 0.9 |
| σ^G^ | *CD1298* | CD630_12980 | [hypothetical protein (YtfJ sporulation protein [7])](#RANGE!_ENREF_8) | 11 | -3.0 | 3.4x10^-3^ | 11 | -6.2 | 2.1x10^-8^ | 22 | 0.2 | 1 | 14 | -2.2 | 8.8x10^-3^ | 13 | -1.0 | 0.8 |
| σ^G^ | *CD1354* | CD630_13540 | hypothetical protein | 14 | -3.3 | 6.1x10^-4^ | 14 | -5.0 | 3.1x10^-9^ | 29 | 0.2 | 1 | 15 | -4.3 | 1.0x10^-8^ | 19 | -0.5 | 1 |
| σ^E^ | *CD1397* | CD630_13970 | hypothetical protein (VPF066 superfamily) | 37 | -3.8 | 5.1x10^-10^ | 44 | -2.3 | 4.0x10^-6^ | 40 | -2.9 | 1.8x10^-10^ | 61 | -0.8 | 0.4 | 47 | -0.7 | 0.7 |
| Spo0A | *CD1404A* | CD630_14041 | hypothetical protein | 30 | -3.2 | 2.7x10^-4^ | 52 | -0.3 | 1 | 47 | -0.5 | 0.7 | 63 | 0.1 | 1 | 40 | -0.5 | 1 |
| Spo0A | *CD1423* | CD630_14230 | hypothetical protein | 20 | -2.6 | 5.5x10^-4^ | 50 | 0.8 | 0.4 | 42 | 0.4 | 0.7 | 48 | 0.5 | 0.9 | 37 | 0.5 | 0.9 |
| ^Spo0A | *CD1463* | CD630_14630 | hypothetical protein | 138 | -2.3 | 9.6x10^-8^ | 185 | -0.9 | 0.1 | 212 | -0.4 | 0.7 | 189 | -1.2 | 8.8x10^-3^ | 189 | -0.1 | 1 |
| ^σ^E^ | *CD1511* | CD630_15110 | [hypothetical protein (coat protein - "CotB," [1])](#RANGE!_ENREF_1) | 137 | -5.5 | 1.3x10^-25^ | 163 | -2.7 | 1.3x10^-11^ | 146 | -4.5 | 3.5x10^-32^ | 270 | -0.3 | 1 | 200 | -0.4 | 1 |
| σ^E^ | *CD1575* | CD630_15750 | hypothetical protein (DUF348, COG3584) | 16 | -3.7 | 2.4x10^-5^ | 19 | -2.0 | 3.3x10^-3^ | 16 | -3.8 | 4.9x10^-8^ | 30 | -0.2 | 1 | 20 | -0.8 | 0.8 |
| ^Spo0A | *CD1581* | CD630_15810 | hypothetical protein | 306 | -2.0 | 2.1x10^-5^ | 387 | -1.0 | 0.03 | 398 | -0.8 | 0.1 | 522 | -0.2 | 1 | 287 | -1.5 | 6.3x10^-3^ |
| ^σ^E^ | *CD1613* | CD630_16130 | hypothetical protein (coat protein "CotA," [1]) | 151 | -4.2 | 6.7x10^-3^ | 174 | -2.7 | 0.1 | 161 | -3.6 | 0.02 | 341 | 0.2 | 1 | 135 | -3.4 | 0.1 |
| σ^E^ | *CD1724* | CD630_17240 | hypothetical protein (DUF3795) | 18 | -4.1 | 4.2x10^-4^ | 20 | -2.9 | 3.2x10^-3^ | 18 | -4.0 | 4.1x10^-4^ | 28 | -1.0 | 0.8 | 20 | -1.4 | 0.6 |
| σ^E^ | *CD1726* | CD630_17260 | hypothetical protein | 12 | -7.1 | 8.5x10^-8^ | 15 | -2.6 | 8.3x10^-4^ | 13 | -4.5 | 1.1x10^-5^ | 20 | -1.0 | 0.5 | 16 | -1.0 | 0.7 |
| Spo0A | *CD1823* | CD630_18230 | hypothetical protein (DUF328) | 185 | -2.1 | 2.9x10^-6^ | 327 | 0.1 | 1 | 273 | -0.4 | 0.7 | 298 | -0.4 | 0.9 | 201 | -0.8 | 0.4 |
| ^Spo0A | *CD1880* | CD630_18800 | hypothetical protein (Cupin_2 superfamily) | 215 | -3.5 | 2.5x10^-16^ | 268 | -1.8 | 8.3x10^-6^ | 319 | -0.9 | 0.04 | 300 | -1.5 | 1.8x10^-5^ | 274 | -0.7 | 0.7 |
| σ^E^ | *CD1884* | CD630_18840 | hypothetical protein | 25 | -3.9 | 2.0x10^-7^ | 27 | -3.2 | 2.1x10^-8^ | 25 | -6.3 | 1.4x10^-13^ | 42 | -0.7 | 0.7 | 30 | -1.0 | 0.6 |
| σ^E^ | *CD1930* | CD630_19300 | hypothetical protein (ComEC-related) | 99 | -1.8 | 4.8x10^-5^ | 94 | -2.7 | 1.5x10^-10^ | 85 | -4.1 | 1.6x10^-24^ | 149 | -0.5 | 0.8 | 102 | -0.9 | 0.6 |
| Spo0A | *CD1941* | CD630_19410 | hypothetical protein | 34 | -4.2 | 2.9x10^-7^ | 87 | 0.6 | 0.5 | 65 | -0.1 | 1 | 82 | 0.3 | 1 | 51 | -0.3 | 1 |
| Spo0A | *CD1967* | CD630_19670 | hypothetical protein | 53 | -3.7 | 4.6x10^-11^ | 159 | 1.0 | 0.04 | 125 | 0.5 | 0.6 | 137 | 0.5 | 0.7 | 90 | 0.2 | 1 |
| σ^E^ | *CD2055* | CD630_20550 | hypothetical protein | 7 | -3.7 | 0.02 | 10 | -1.2 | 0.5 | 8 | -3.1 | 0.01 | 28 | 1.4 | 0.2 | 6 | -2.7 | 0.1 |
| ^σ^G^ | *CD2112* | CD630_21120 | hypothetical protein | 368 | -4.9 | 3.2x10^-28^ | 391 | -4.5 | 1.6x10^-28^ | 633 | -0.5 | 0.4 | 414 | -4.9 | 1.4x10^-35^ | 411 | -1.5 | 5.2x10^-3^ |
| σ^E^ | *CD2121* | CD630_21210 | hypothetical protein | 54 | -5.4 | 2.9x10^-8^ | 64 | -2.7 | 5.1x10^-4^ | 56 | -5.5 | 2.1x10^-8^ | 96 | -0.7 | 0.8 | 62 | -1.4 | 0.4 |
| ^σ^F^ | *CD2245A* | CD630_22451 | hypothetical protein (Yqz-like) | 8 | -6.5 | 2.2x10^-5^ | 8 | -5.9 | 1.7x10^-5^ | 11 | -1.5 | 0.1 | 11 | -2.3 | 0.1 | 10 | -1.3 | 0.7 |
| σ^G^ | *CD2315* | CD630_23150 | hypothetical protein (PIG-L superfamily) | 21 | -3.2 | 5.8x10^-5^ | 24 | -2.3 | 6.8x10^-5^ | 27 | -1.5 | 0.01 | 26 | -2.2 | 1.9x10^-4^ | 25 | -0.9 | 0.7 |
| Spo0A | *CD2374* | CD630_23740 | hypothetical protein | 41 | -4.6 | 4.4x10^-13^ | 58 | -1.3 | 0.02 | 53 | -1.7 | 1.2x10^-4^ | 79 | -0.3 | 1 | 58 | -0.5 | 1 |
| σ^F^ | *CD2375* | CD630_23750 | hypothetical protein (DUF1540) | 119 | -5.2 | 5.8x10^-24^ | 143 | -2.5 | 5.1x10^-10^ | 240 | 0.0 | 1 | 174 | -1.6 | 1.4x10^-5^ | 171 | -0.5 | 1 |
| σ^E^ | *CD2395* | CD630_23950 | hypothetical protein | 6 | -1.7 | 0.5 | 5 | -4.2 | 5.5x10^-3^ | 5 | –Inf | 3.7x10^-4^ | 7 | -1.2 | 0.7 | 5 | -2.7 | 0.3 |
| ^σ^K^ | *CD2399* | CD630_23990 | hypothetical protein (CotJA superfamily) | 364 | -8.9 | 4.2x10^-21^ | 427 | -3.1 | 4.0x10^-6^ | 379 | -7.6 | 3.5x10^-19^ | 702 | -0.5 | 0.9 | 314 | -7.0 | 4.0x10^-16^ |
| σ^K^ | *CD2409* | CD630_24090 | hypothetical protein | 11 | -3.8 | 8.5x10^-4^ | 14 | -1.7 | 0.1 | 14 | -1.6 | 0.03 | 24 | 0.2 | 1 | 10 | -2.7 | 0.02 |
| Spo0A | *CD2435* | CD630_24351 | hypothetical protein (Yqz-like) | 64 | -2.0 | 7.2x10^-5^ | 73 | -1.4 | 2.8x10^-3^ | 75 | -1.2 | 4.4x10^-3^ | 87 | -0.9 | 0.1 | 70 | -0.7 | 0.7 |
| ^σ^E^ | *CD2634* | CD630_26340 | hypothetical protein | 3 | -2.6 | 0.4 | 2 | –Inf | 0.04 | 3 | -1.2 | 0.8 | 3 | -2.8 | 0.5 | 3 | -1.3 | 1 |
| ^σ^G^ | *CD2635* | CD630_26350 | hypothetical protein (YIEGIA family) | 40 | -3.8 | 9.1x10^-11^ | 43 | -3.7 | 5.6x10^-13^ | 58 | -1.0 | 0.05 | 45 | -4.0 | 5.7x10^-17^ | 49 | -0.9 | 0.5 |
| Spo0A | *CD2657* | CD630_26570 | hypothetical protein | 12 | -3.2 | 1.5x10^-3^ | 21 | -0.1 | 1 | 16 | -1.1 | 0.3 | 25 | 0.1 | 1 | 14 | -0.9 | 0.8 |
| σ^E^ | *CD2687* | CD630_26870 | hypothetical protein | 4 | -1.6 | 0.6 | 3 | –Inf | 2.8x10^-3^ | 5 | -0.7 | 1 | 5 | -1.8 | 0.5 | 5 | -0.3 | 1 |
| ^σ^G^ | *CD2687A* | CD630_26871 | hypothetical protein | 73 | -4.9 | 3.0x10^-18^ | 80 | -3.6 | 1.0x10^-15^ | 118 | -0.7 | 0.1 | 98 | -2.1 | 7.0x10^-8^ | 92 | -0.9 | 0.4 |
| σ^E^ | *CD2799A* | CD630_27991 | hypothetical protein | 50 | -3.3 | 3.5x10^-10^ | 58 | -2.4 | 2.2x10^-7^ | 50 | -4.1 | 3.9x10^-18^ | 79 | -0.9 | 0.1 | 59 | -1.0 | 0.4 |
| σ^G^ | *CD2808* | CD630_28080 | hypothetical protein | 25 | -4.2 | 3.5x10^-9^ | 25 | -7.4 | 2.9x10^-17^ | 55 | 0.3 | 1 | 30 | -2.8 | 4.4x10^-8^ | 30 | -1.0 | 0.5 |
| ^σ^G^ | *CD2809* | CD630_28090 | hypothetical protein (DUF1540) | 32 | -4.1 | 3.6x10^-10^ | 34 | -4.1 | 2.6x10^-11^ | 62 | -0.1 | 1 | 37 | -3.5 | 1.0x10^-8^ | 43 | -0.6 | 1 |
| ^σ^E^ | *CD3007* | CD630_30070 | hypothetical protein | 41 | -3.3 | 2.5x10^-8^ | 52 | -1.6 | 1.8x10^-3^ | 48 | -2.1 | 2.1x10^-6^ | 81 | -0.1 | 1 | 41 | -1.8 | 5.3x10^-3^ |
| σ^E^ | *CD3150A* | CD630_31501 | hypothetical protein | 19 | -3.4 | 1.6x10^-4^ | 21 | -3.1 | 5.2x10^-4^ | 19 | -4.1 | 5.7x10^-6^ | 29 | -1.1 | 0.4 | 22 | -1.0 | 0.8 |
| σ^E^ | *CD3151* | CD630_31510 | hypothetical protein | 5 | -1.6 | 0.7 | 5 | -2.6 | 0.1 | 4 | -5.3 | 4.7x10^-3^ | 6 | -1.5 | 0.7 | 5 | -1.3 | 0.8 |
| σ^E^ | *CD3234* | CD630_32340 | hypothetical protein (methyltransferase domain) | 21 | -2.2 | 5.0x10^-3^ | 22 | -2.3 | 2.0x10^-4^ | 19 | -4.1 | 8.2x10^-10^ | 32 | -0.6 | 0.8 | 25 | -0.5 | 0.9 |
| Spo0A | *CD3289* | CD630_32890 | hypothetical protein | 38 | -2.8 | 5.8x10^-7^ | 85 | 0.5 | 0.7 | 97 | 0.9 | 0.1 | 99 | 0.7 | 0.4 | 52 | -0.3 | 1 |
| Spo0A | *CD3290* | CD630_32900 | hypothetical protein | 120 | -2.4 | 4.4x10^-8^ | 280 | 0.7 | 0.3 | 278 | 0.7 | 0.2 | 284 | 0.6 | 0.6 | 171 | 0.0 | 1 |
| σ^E^ | *CD3457* | CD630_34570 | hypothetical protein | 40 | -3.6 | 1.0x10^-9^ | 46 | -2.3 | 2.4x10^-6^ | 43 | -3.1 | 6.8x10^-11^ | 66 | -0.7 | 0.5 | 46 | -1.1 | 0.3 |
| σ^E^ | *CD3464* | CD630_34640 | hypothetical protein YdcC involved in sporulation [3] | 557 | -4.6 | 1.5x10^-26^ | 609 | -3.6 | 2.3x10^-21^ | 574 | -5.0 | 2.3x10^-42^ | 965 | -0.7 | 0.2 | 698 | -0.9 | 0.2 |
| σ^E^ | *CD3465* | CD630_34650 | hypothetical protein (CBS domain) | 69 | -2.8 | 1.7x10^-8^ | 72 | -2.9 | 4.0x10^-10^ | 71 | -3.0 | 1.9x10^-11^ | 109 | -0.7 | 0.3 | 89 | -0.5 | 0.9 |
| ^σ^E^ | *CD3522* | CD630_35220 | hypothetical protein | 286 | -6.1 | 2.8x10^-10^ | 317 | -4.0 | 2.1x10^-6^ | 298 | -6.0 | 8.7x10^-11^ | 449 | -1.3 | 0.2 | 312 | -1.8 | 0.2 |
| σ^E^ | *CD3551B* | CD630_35512 | hypothetical protein | 42 | -6.6 | 8.5x10^-13^ | 48 | -3.3 | 7.5x10^-8^ | 48 | -3.2 | 1.0x10^-6^ | 67 | -1.2 | 0.1 | 54 | -0.9 | 0.7 |
| ^σ^K^ | *CD3580* | CD630_35800 | hypothetical protein | 59 | -5.6 | 1.5x10^-19^ | 68 | -3.0 | 9.2x10^-11^ | 62 | -5.1 | 6.6x10^-24^ | 111 | -0.5 | 0.8 | 51 | -4.7 | 2.7x10^-16^ |
| σ^E^ | *CD3635* | CD630_36350 | hypothetical protein | 10 | -3.5 | 0.1 | 11 | -3.9 | 0.04 | 11 | -3.9 | 0.03 | 17 | -0.9 | 1 | 12 | -1.3 | 0.9 |
| σ^E^ | *CD3636A* | CD630_36361 | hypothetical protein | 4 | -2.4 | 0.3 | 4 | -4.9 | 0.01 | 4 | -3.5 | 0.04 | 5 | -1.8 | 0.6 | 4 | -2.1 | 0.6 |
| σ^E^ | *CD3638* | CD630_36380 | hypothetical protein | 32 | -3.0 | 1.6x10^-5^ | 33 | -2.9 | 3.7x10^-8^ | 31 | -4.2 | 4.6x10^-14^ | 46 | -1.1 | 0.1 | 34 | -1.2 | 0.2 |

^†^ Two factors are listed in the table for genes whose expression was dependent on both σ^E^ and σ^G^ (adjusted p-value ≤ 0.05, log_2_FC ≤ -2). *Dep.* indicates the most downstream sigma factor on which gene expression depends upon. *BM* refers to base mean, the mean of the counts after they were divided by the size factors to adjust for different sequencing depths. This value is the mean for the sample relative to wild type. *log_2_FC* denotes log_2_fold-change. A negative value indicates that the gene was downregulated relative to wild type. ^ Indicates that gene product was detected in Lawley *et al*. proteomic analysis of purified spores [[8](#_ENREF_7)]. *–Inf* indicates that no transcript was detected in the mutant relative to wild type.
