# Supplementary material for: Global Analysis of the Sporulation Pathway of Clostridium difficile
Source: PLoS Genet. 2013 Aug 8;9(8):e1003660. doi: 10.1371/journal.pgen.1003660 (PMC3738446; doi:10.1371/journal.pgen.1003660)
Supplement: Table S11 — E. coli strains used in this study. (DOCX) [file pgen.1003660.s018.docx]

**Table S11. *E. coli* strains used in this study.**

| **Strain** | **Relevant genotype or features** | **Source or reference** |
| --- | --- | --- |
| DH5α | F– Φ80*lacZ*ΔM15 Δ(*lacZYA-argF*) U169 *recA1 endA1 hsdR17* (rK–, mK+) *phoA supE44* λ– *thi-1 gyrA96 relA1* | D. Cameron |
| BL21(DE3) | F– *ompT hsdSB*(rB–, mB–) *gal dcm* (DE3) | Novagen |
| HB101 | F- *mcrB mrr hsdS20*(rB- mB-) *recA13 leuB6 ara-13 proA2 lavYI galK2 xyl-6 mtl-1 rpsL20* | C. Ellermeier |
| 7 | pET22b in DH5α | D. Higgins |
| 269 | pET28a in DH5α | M. Bogyo |
| 547 | pET28a-*cd3580* in BL21(DE3) | Novagen |
| 556 | pJS107 in DH5α | J. Sorg |
| 577 | pET29a-*cd3522* clone 4 in BL21(DE3) | This study |
| 659 | pJS107-*sigE* targeting bp 119 HB101/pK424 | This study |
| 680 | pJS107-*sigK* targeting bp 230 in HB101/pK424 | This study |
| 686 | pMTL83151 in HB101/pK424 | This study |
| 655 | pMTL83151 in DH5α | This study |
| 701 | pMTL84151 in DH5α | This study |
| 703 | pMTL843151 in HB101/pK424 | This study |
| 735 | pJS107-*sigG* targeting bp 546 in HB101/pK424 | This study |
| 743 | pET22b-*sigG* in BL21(DE3) | This study |
| 755 | pET22b-∆23aa *sigE* in BL21(DE3) | This study |
| 756 | pET30a-*sigK*(TAA) in BL21(DE3) | This study - SOE |
| 782 | pJS107-*sigF* 459 in HB101/pK424 | This study |
| 811 | pMTL83151-*spoIIGA-σE* in HB101/pK424 | This study |
| 812 | pMTL83151-*sigG* in HB101/pK424 | This study |
| 813 | pMTL83151-*sigK* in HB101/pK424 | This study - SOE |
| 853 | pET21a-*GPR* in BL21(DE3) | This study |
| 854 | pET21a-*sspA* in BL21(DE3) | This study |
| 869 | pMTL84151-*spoIIA-spoIIAB-sigF* in HB101/pK424 | This study |
| 881 | pET22b-*spoVT* in BL21(DE3) | This study |
| 921 | pET22b-*sigF* in BL21(DE3) | This study |
