# Supplementary material for: Global Analysis of the Sporulation Pathway of Clostridium difficile
Source: PLoS Genet. 2013 Aug 8;9(8):e1003660. doi: 10.1371/journal.pgen.1003660 (PMC3738446; doi:10.1371/journal.pgen.1003660)
Supplement: Table S12 — Plasmids used in this study. (DOCX) [file pgen.1003660.s019.docx]

**Table S12. Plasmids used in this study.**

| **Plasmids** | **Relevant features** | **Source or reference** |
| --- | --- | --- |
| pET22b | *bla* | Novagen |
| pET28a | *kan* | Novagen |
| pRSFduet1 | *kan* | Novagen |
| pK424 | Tra^+^ Mob^+^; *bla, tet* | C. Ellermeier |
| pJS107 | TargeTron construct based on pJIR750ai (group II intron *ermB*::RAM, *ltrA*); *catP* | J. Sorg |
| pCE245 | TargeTron construct based on pJIR750ai (group II intron *ermB*::RAM, *ltrA*); *catP* | C. Ellermeier |
| pMTL83151 | pCB102, Tra^+^; *catP* | N. Minton, [[9](#_ENREF_9)] |
| pMTL84151 | pCD6, Tra^+^; *catP* | N. Minton, [[9](#_ENREF_9)] |
