# Supplementary material for: Global Analysis of the Sporulation Pathway of Clostridium difficile
Source: PLoS Genet. 2013 Aug 8;9(8):e1003660. doi: 10.1371/journal.pgen.1003660 (PMC3738446; doi:10.1371/journal.pgen.1003660)
Supplement: Table S13 — Primers used in this study. (DOCX) [file pgen.1003660.s020.docx]

**Table S13. Primers used in this study.**

| **Primer** | **Name** | **Sequence** |
| --- | --- | --- |
| 596 | 5' NdeI *CD3522* | AGCCATATGGGTTCTAAAAGAAAGTTTAAAAGAG |
| 597 | 3' XhoI *CD3522* | AACGCTCGAGCCTAACTATATTTCTTTGTCTAG |
| 532 | 3' Universal EBS | CGAAATTAGAAACTTGCGTTCAGTAAAC |
| 539 | 5' IBS1.2 *spo0A* 178 | AAAAAAGCTTATAATTATCCTTATTATTCCATCTAGTGCGCCCAGATAGGGTG |
| 540 | 3' EBS1d *spo0A* 178 | CAGATTGTACAAATGTGGTGATAACAGATAAGTCCATCTAGTTAACTTACCTTTCTTTGT |
| 541 | 5' EBS2 *spo0A* 178 | TGAACGCAAGTTTCTAATTTCGGTTAATAATCGATAGAGGAAAGTGTCT |
| 556 | 5' NdeI *spo0A* | CCCCATATGGGGGGATTTTTAGTGGAAAAAATC |
| 557 | 3' XhoI *spo0A* | GCCCCTCGAGTTTAACCATACTATGTTCTAGTCTTAATTTATC |
| 575 | 5’ *sleC* 296-463 | TTGAAGCAAGACAAGGAGTTCCC |
| 576 | 3’ *sleC* 296-463 | CGAAACCAGTAGGAGGAGGTAATGG |
| 653 | 5' IBS1 *sigE* 119 | AAAAAAGCTTATAATTATCCTTAAAAGCCCCATTTGTGCGCCCAGATAGGGTG |
| 654 | 3' EBS1d *sigE* 119 | CAGATTGTACAAATGTGGTGATAACAGATAAGTCCCATTTCTTAACTTACCTTTCTTTGT |
| 655 | 5' EBS2 *sigE* 119 | TGAACGCAAGTTTCTAATTTCGGTTGCTTTCCGATAGAGGAAAGTGTCT |
| 681 | 5' IBS1 *sigK* 265 | AAAAAAGCTTATAATTATCCTTACATATCCTTCTAGTGCGCCCAGATAGGGTG |
| 682 | 3' EBS1d *sigK* 265 | CAGATTGTACAAATGTGGTGATAACAGATAAGTCCTTCTAGATAACTTACCTTTCTTTGT |
| 683 | 5' EBS2 *sigK* 265 | TGAACGCAAGTTTCTAATTTCGGTTATATGTCGATAGAGGAAAGTGTCT |
| 687 | 5' NdeI *sigE* | AAAGCATATGTTACGATTGAAAGAAAGAATAATAAGC |
| 688 | 3' XhoI *sigE* | AAAGCTCGAGTACAAATTTTTTCATTTCTTTTTGCAACCTTGAG |
| 689 | 5' NcoI *sigK* | AAAGCCATGGCAGCTCTTAAATCTTTTGAAAAACCC |
| 690 | 3' XhoI *sigK* pre skin | AAAGCTCGAGATTTCATTACCTTCTTTATCTGTTCC |
| 723 | 5' NdeI *sigG* | AAATCATATGCAAGTTAATAAGGTTGAAATATG |
| 724 | 3' XhoI *sigG* | AAGACTCGAGTACATATTTTCTCATATTTTTTAAAGC |
| 725 | 5' NotI *spoIIGA* | AGAATGCGGCCGCGTAAATATACCAAAAGTAGAGCG |
| 726 | 3' XhoI *sigE* down | AAAGCTCGAGGAACTGGAAGTTCTGATGTATTAACACC |
| 727 | 5' NdeI ∆23aa *sigE* | AAATCATATGCCAAAGGGAATATACTATATGGGA |
| 728 | 5' IBS1 *sigG* 546 | AAAAAAGCTTATAATTATCCTTAGATGCCATATTCGTGCGCCCAGATAGGGTG |
| 729 | 3' EBS1d *sigG* 546 | CAGATTGTACAAATGTGGTGATAACAGATAAGTCATATTCGTTAACTTACCTTTCTTTGT |
| 730 | 5' EBS2 *sigG* 546 | TGAACGCAAGTTTCTAATTTCGATTGCATCTCGATAGAGGAAAGTGTCT |
| 734 | 5' NotI *sigK* up | AGAATGCGGCCGCCTATTTCCTTTTATATTTATTGACACCTC |
| 735 | 5' *sigK* SOE | CGGAACAGATAAAGAAGGTAATGAAATAAGTCTTTTAGATATACTTGGTACAGAGG |
| 736 | 3' *sigK* rev oes | CCTCTGTACCAAGTATATCTAAAAGACTTATTTCATTACCTTCTTTATCTGTTCCG |
| 737 | 3' XhoI *sigK* TAA | AAATCTCGAGTTAACTTTCTTGAACAAGCTCTTTTTCTAG |
| 775 | 5' IBS1 *sigF* 459 | AAAAAAGCTTATAATTATCCTTAGATTTCGTAATGGTGCGCCCAGATAGGGTG |
| 776 | 3' EBS1d *sigF* 459 | CAGATTGTACAAATGTGGTGATAACAGATAAGTCGTAATGGCTAACTTACCTTTCTTTGT |
| 777 | 5' EBS2 *sigF* 459 | TGAACGCAAGTTTCTAATTTCGATTAAATCTCGATAGAGGAAAGTGTCT |
| 790 | 5' NdeI *sigF* | AAATCATATGGAAGTAACTGTTGCCAG |
| 791 | 3' XhoI *sigF* | AATACTCGAGCGATATATATTCTTTTAACTTAGACAATACTTTC |
| 792 | 5' *CD1433* 531-1102 | TGGAATGCCAGTGATTTTACC |
| 793 | 3' *CD1433* 531-1102 | TGTATGGGTCTGTTATTTGTGGTC |
| 794 | 5' *CD3580* 207-622 | TGGCTTACAGGAAGAAGAACG |
| 795 | 3' *CD3580* 207-622 | CAAAACTTTCAGGTGCTATTTCGTC |
| 796 | 5' *CD1511* 76-348 | GCTCTTTTTTTAGAAGCAGGATTC |
| 797 | 3' *CD1511* 76-348 | ACCAACTTGTGGGTTTACACC |
| 798 | 5' *spoIVA* 171-415 | GGATAGAACAAGAGATGAGATACCC |
| 799 | 3' *spoIVA* 171-415 | CTGCTGCCTTTTCAAATGTC |
| 810 | 5' *spoVT* 26-441 | GAATAGATGATCTTGGAAGGGTAG |
| 811 | 3' *spoVT* 26-441 | ACCTATACAGTCACCAGATGCAC |
| 835 | 5' NotI *sigG* | AGAATGCGGCCGCGTTGGTTATGGCACTTGACAG |
| 836 | 3' XhoI *sigG* | ACAAGCTCGAGCAGTCCATGGATTACTTATAC |
| 883 | 5' NheI *CD2470* (*gpr*) | AAGAGCTAGCATTAGTGTAAGAACAGATTTAGCTTTAG |
| 884 | 3' XhoI *CD2470* (*gpr*) | AAGACTCGAGTACCAATCTACCTGGATGTAAAGAC |
| 885 | 5' NdeI *CD2688* (*sspA*) | AAGACATATGGCAAGTAACAATAACAACAACAGAACA |
| 886 | 3' XhoI *CD2688* (*sspA*) | AAGACTCGAGTCTGTTGCTTTTTCCAGCCATTTG |
| 954 | 5' Not1 *spoIIAA* | ACAAGGCGGCCGCCATTGAAGGAATAAAAATATAATTATAG |
| 956 | 3' Xho1 *sigF* TAA | ACAAGCTCGAGCTACATATCATAAGTATTATCACG |
| 989 | 5' *CD3522* 502-853 | GAGTATGAAGACTATTGGGAAGATG |
| 990 | 3' *CD3522* 502-853 | GTTTATTTCCACTCTCTCCTTGAC |
| 995 | 5' *sspB* 1-207 | TTGAGGAGGAAATTTACTATG |
| 996 | 3' *sspB* 1-207 | TTGTCTTTCAGCCATTTCAAC |
| 975 | 5' NdeI *spoVT* | AGAATCATATGAAAGCAACAGGTATAGTTAGAAG |
| 976 | 3' XhoI *spoVT* | ACAAGCTCGAGTTGAACTTGTTTTCCTAAAAAG |
| 1002 | 5' *rpoB* 235-442 | GAGTGTAAAGAGAGAGATGC |
| 1003 | 3' *rpoB* 235-442 | CTTCCGCATAGTAAACACC |
| 1032 | 5' *dacF* 1129-340 | GATGGATGTTGGTAGTGGAC |
| 1033 | 3' *dacF* 1129-340 | CCTTTTGAGTTTCTCCTGGC |
| 1187 | 5’ *GPR* 332-626 | CTTCTGATGCGTTAGGACC |
| 1188 | 3’ *GPR* 332-626 | GTATTGCCAACACCTCCAC |
| 1191 | 5’ *CD2376* 297-439 | GTTGAGTAGGGTTTTTAGTG |
| 1192 | 3’ *CD2376* 297-439 | CTTTTGTTGCTGTTGATGC |
| 1213 | 5’ *CD0125 (spoIIQ)* 190-553 | GATGCTATCCCTACTGCAACG |
| 1214 | 5’ *CD0125 (spoIIQ)* 190-553 | GTCCTTCTGTTACCTTCTGTTC |
