# Supplementary material for: Global Analysis of the Sporulation Pathway of Clostridium difficile
Source: PLoS Genet. 2013 Aug 8;9(8):e1003660. doi: 10.1371/journal.pgen.1003660 (PMC3738446; doi:10.1371/journal.pgen.1003660)
Supplement: Table S14 — Genes whose expression is increased in the absence of spo0A. BM refers to base mean, the mean of the counts after they were divided by the size factors to adjust for different sequencing depths. This value is the mean for the sample relative to wild type. log2FC denotes log2fold-change. A positive value indicates that the gene was upregulated in the spo0A− mutant relative to wild type. +Inf indicates that no transcript was detected in wild type relative to the spo0A − mutant. (DOCX) [file pgen.1003660.s021.docx]

**Table S14. Genes whose expression is increased in the absence of *spo0A*.**

|  |  |  | **Spo0A** | | | **σ^F^** | | | **σ^E^** | | | **σ^G^** | | | **σ^K^** | | |
| --- | --- | --- | --- | --- | --- | --- | --- | --- | --- | --- | --- | --- | --- | --- | --- | --- | --- |
| **Name** | **locus_tag** | **description** | **BM** | **log_2_FC** | **adjP** | **BM** | **log_2_FC** | **adjP** | **BM** | **log_2_FC** | **adjP** | **BM** | **log_2_FC** | **adjP** | **BM** | **log_2_FC** | **adjP** |
| *CD3246* | CD630_32460 | surface protein | 4765 | 3.5 | 6.7x10^-18^ | 984 | 0.5 | 0.7 | 960 | 0.5 | 0.4 | 1244 | 1.5 | 0.6 | 901 | 0.1 | 1 |
| *CD3406* | CD630_34060 | iron-only hydrogenase,electron-transferring subunit HymB-like | 685 | 2.0 | 1.1x10^-3^ | 269 | -0.2 | 1 | 281 | 0.0 | 1 | 256 | 0.2 | 1 | 300 | -0.1 | 1 |
| *gabT* | CD630_21580 | 4-aminobutyrate aminotransferase | 675 | 3.1 | 3.2x10^-15^ | 192 | 0.6 | 0.8 | 183 | 0.5 | 0.3 | 234 | 1.5 | 0.7 | 162 | 0.0 | 1 |
| *CD3458* | CD630_34580 | membrane protein | 602 | 2.1 | 5.5x10^-8^ | 280 | 0.5 | 0.7 | 263 | 0.3 | 0.7 | 258 | 0.8 | 0.4 | 277 | 0.3 | 1 |
| *CD3006* | CD630_30060 | alcohol dehydrogenase | 541 | 2.9 | 3.2x10^-13^ | 138 | 0.1 | 1 | 177 | 0.8 | 0.1 | 127 | 0.4 | 0.9 | 152 | 0.2 | 1 |
| *CD2502* | CD630_25020 | pyridoxal phosphate-dependent transferase | 530 | 3.0 | 3.0x10^-14^ | 158 | 0.6 | 0.7 | 159 | 0.6 | 0.2 | 184 | 1.4 | 0.3 | 150 | 0.3 | 1 |
| *proC* | CD630_14950 | pyrroline-5-carboxylate reductase | 454 | 2.6 | 1.5x10^-10^ | 152 | 0.3 | 0.9 | 156 | 0.4 | 0.7 | 146 | 0.7 | 0.6 | 164 | 0.3 | 1 |
| *nth* | CD630_05650 | endonuclease III | 377 | 2.8 | 5.1x10^-12^ | 137 | 0.8 | 0.7 | 135 | 0.8 | 0.1 | 173 | 1.7 | 0.7 | 138 | 0.6 | 0.7 |
| *ermB* | CD630_20070 | ribosomal RNA adenine N-6-methyltransferase | 364 | 6.2 | 8.8x10^-21^ | 146 | 4.8 | 7.0x10^-25^ | 146 | 4.8 | 7.0x10^-33^ | 146 | 5.1 | 5.0x10^-27^ | 169 | 4.9 | 5.1x10^-35^ |
| *ermB* | CD630_20100 | ribosomal RNA adenine N-6-methyltransferase | 359 | 6.4 | 8.7x10^-23^ | 144 | 4.9 | 1.6x10^-25^ | 142 | 5.0 | 3.9x10^-29^ | 152 | 5.3 | 8.8x10^-29^ | 167 | 5.1 | 1.0x10^-35^ |
| *CD2448* | CD630_24480 | MiaB-like tRNA modifying enzyme | 344 | 2.1 | 2.1x10^-3^ | 124 | -0.2 | 1 | 120 | -0.3 | 0.9 | 106 | -0.1 | 1 | 143 | 0.0 | 1 |
| *CD0588* | CD630_05880 | hypothetical protein | 328 | 3.2 | 3.7x10^-15^ | 85 | 0.6 | 0.7 | 77 | 0.4 | 0.7 | 86 | 1.1 | 0.6 | 72 | 0.0 | 1 |
| *CD0587* | CD630_05870 | hypothetical protein | 258 | 3.3 | 2.0x10^-15^ | 65 | 0.7 | 0.8 | 58 | 0.5 | 0.7 | 59 | 1.0 | 0.7 | 53 | 0.0 | 1 |
| *CD0545* | CD630_05450 | hypothetical protein | 232 | 2.3 | 3.6x10^-8^ | 90 | 0.3 | 0.9 | 90 | 0.3 | 0.8 | 90 | 0.8 | 0.7 | 89 | 0.1 | 1 |
| *CD0340* | CD630_03400 | hypothetical protein | 183 | 3.8 | 1.3x10^-12^ | 27 | 0.2 | 1 | 26 | 0.0 | 1 | 22 | 0.2 | 1 | 23 | -0.6 | 0.9 |
| *CD2768* | CD630_27680 | cell-wall hydrolase | 180 | 2.3 | 6.0x10^-8^ | 70 | 0.3 | 0.9 | 63 | 0.1 | 1 | 72 | 0.9 | 0.4 | 78 | 0.4 | 0.9 |
| *CD1494* | CD630_14940 | HTH-type transcriptional regulator | 176 | 2.8 | 3.1x10^-4^ | 49 | 0.1 | 1 | 51 | 0.3 | 0.9 | 45 | 0.4 | 1 | 53 | 0.2 | 1 |
| *ssuB2* | CD630_29900 | ABC transporter sulfonate-family ATP-binding protein | 153 | 3.4 | 9.2x10^-9^ | 29 | 0.1 | 1 | 25 | -0.3 | 1 | 28 | 0.5 | 0.9 | 31 | 0.1 | 1 |
| *CD2830* | CD630_28300 | hypothetical protein | 143 | 2.9 | 2.7x10^-11^ | 58 | 1.2 | 0.03 | 51 | 1.0 | 0.1 | 45 | 1.1 | 0.2 | 62 | 1.2 | 0.04 |
| *folE* | CD630_14490 | GTP cyclohydrolase I | 120 | 2.1 | 1.8x10^-4^ | 58 | 0.5 | 0.8 | 54 | 0.3 | 0.9 | 69 | 1.3 | 0.7 | 59 | 0.4 | 1 |
| *ssuA2* | CD630_29890 | ABC transporter sulfonate-family extracellular solute-binding protein | 115 | 3.1 | 1.4x10^-6^ | 31 | 0.6 | 0.7 | 25 | 0.1 | 1 | 23 | 0.4 | 1 | 28 | 0.2 | 1 |
| *aspC* | CD630_01070 | Aspartate aminotransferase | 97 | 2.1 | 7.5x10^-6^ | 33 | -0.4 | 0.9 | 47 | 0.6 | 0.6 | 31 | 0.0 | 1 | 35 | -0.5 | 1 |
| *CD1522* | CD630_15220 | polysaccharide deacetylase | 93 | 2.2 | 5.8x10^-4^ | 37 | 0.1 | 1 | 31 | -0.3 | 1 | 40 | 0.8 | 0.8 | 37 | 0.0 | 1 |
| *nrdE* | CD630_29950 | ribonucleoside-diphosphate reductase subunit alpha (Ribonucleotide reductase large subunit) | 88 | 2.9 | 2.1x10^-9^ | 40 | 1.4 | 0.02 | 32 | 1.0 | 0.2 | 27 | 1.0 | 0.6 | 37 | 1.1 | 0.2 |
| *CD2499* | CD630_24990 | hypothetical protein | 85 | 2.3 | 8.4x10^-3^ | 58 | 1.5 | 3.4x10^-3^ | 42 | 0.9 | 0.2 | 55 | 1.8 | 2.3x10^-3^ | 50 | 1.1 | 0.1 |
| *CD0893* | CD630_08930 | iron-dependent hydrogenase | 76 | 2.6 | 8.6x10^-6^ | 27 | 0.5 | 0.8 | 30 | 0.8 | 0.3 | 26 | 0.9 | 0.6 | 28 | 0.4 | 1 |
| *CD2009A* | CD630_20091 | hypothetical protein | 75 | 6.0 | 5.4x10^-10^ | 19 | 3.9 | 1.4x10^-7^ | 21 | 4.1 | 1.5x10^-9^ | 32 | 5.0 | 4.2x10^-14^ | 33 | 4.7 | 1.5x10^-14^ |
| *CD2006A* | CD630_20061 | hypothetical protein | 73 | 6.6 | 1.8x10^-9^ | 21 | 4.6 | 9.5x10^-10^ | 23 | 4.8 | 3.6x10^-12^ | 29 | 5.4 | 2.6x10^-14^ | 34 | 5.3 | 1.7x10^-16^ |
| *CD3405* | CD630_34050 | iron-only hydrogenase,electron-transferring subunit HymA-like | 69 | 2.4 | 1.8x10^-6^ | 24 | 0.1 | 1 | 27 | 0.5 | 0.9 | 25 | 0.8 | 0.7 | 25 | 0.1 | 1 |
| *rbsK* | CD630_02990 | ribokinase, pfkB family | 62 | 2.2 | 2.6x10^-5^ | 52 | 1.7 | 0.02 | 59 | 2.0 | 1.9x10^-6^ | 47 | 1.9 | 0.4 | 46 | 1.4 | 0.4 |
| *CD0566* | CD630_05660 | tRNA/rRNA methyltransferase | 61 | 2.3 | 1.3x10^-5^ | 25 | 0.3 | 1 | 27 | 0.5 | 0.7 | 36 | 1.6 | 0.4 | 29 | 0.5 | 1 |
| *CD2449* | CD630_24490 | methyltransferase | 54 | 2.3 | 1.8x10^-4^ | 18 | -0.2 | 1 | 19 | -0.1 | 1 | 17 | 0.1 | 1 | 22 | 0.2 | 1 |
| *ssuC2* | CD630_29910 | ABC transporter sulfonate-family permease | 52 | 3.3 | 3.3x10^-9^ | 9 | -0.5 | 1 | 9 | -0.3 | 1 | 8 | -0.1 | 1 | 11 | 0.0 | 1 |
| *CD3608* | CD630_36080 | ABC transporter ATP-binding protein | 52 | 2.3 | 0.02 | 18 | -0.1 | 1 | 20 | 0.3 | 1 | 13 | -0.4 | 1 | 17 | -0.5 | 1 |
| *CD2052* | CD630_20520 | lipoprotein | 50 | 2.1 | 3.9x10^-5^ | 24 | 0.5 | 0.8 | 25 | 0.6 | 0.6 | 22 | 0.8 | 0.8 | 30 | 0.9 | 0.5 |
| *CD0350* | CD630_03500 | HAD-superfamily hydrolase | 49 | 2.6 | 1.7x10^-6^ | 19 | 0.7 | 0.7 | 15 | 0.1 | 1 | 20 | 1.2 | 0.3 | 21 | 0.8 | 0.7 |
| *rbsR* | CD630_02980 | LacI family transcriptional regulator | 47 | 2.1 | 1.3x10^-4^ | 32 | 1.3 | 0.5 | 35 | 1.5 | 7.4x10^-3^ | 31 | 1.7 | 0.8 | 25 | 0.6 | 0.9 |
| *tRNA-Thr* | CD630_t0510 | tRNA | 43 | 2.2 | 4.5x10^-5^ | 24 | 0.9 | 0.7 | 18 | 0.3 | 1 | 29 | 1.7 | 0.4 | 19 | 0.2 | 1 |
| *tdcF* | CD630_31870 | regulatory endoribonuclease | 37 | 2.5 | 1.6x10^-5^ | 15 | 0.7 | 0.7 | 14 | 0.5 | 0.9 | 14 | 1.0 | 0.7 | 15 | 0.5 | 1 |
| *CD2177* | CD630_21770 | ABC transporter cystine/aminoacid-family extracellular solute-binding protein | 31 | 2.2 | 1.9x10^-3^ | 12 | 0.0 | 1 | 12 | 0.1 | 1 | 14 | 0.9 | 0.8 | 12 | -0.1 | 1 |
| *CD2970* | CD630_29700 | thiolase | 28 | 2.7 | 1.6x10^-5^ | 11 | 0.7 | 0.8 | 12 | 0.9 | 0.5 | 14 | 1.8 | 0.6 | 11 | 0.7 | 1 |
| *CD0351* | CD630_03510 | [hypothetical protein](#RANGE!_ENREF_1) | 28 | 2.6 | 1.5x10^-4^ | 10 | 0.4 | 1 | 9 | 0.3 | 1 | 13 | 1.5 | 0.6 | 13 | 0.9 | 0.8 |
| *CD0589* | CD630_05890 | hypothetical protein | 27 | 2.2 | 5.1x10^-4^ | 9 | -0.4 | 1 | 10 | 0.0 | 1 | 7 | -0.3 | 1 | 8 | -1.0 | 0.8 |
| *CD3071* | CD630_30710 | glycosyl hydrolase | 25 | 2.1 | 1.6x10^-3^ | 11 | 0.2 | 1 | 11 | 0.4 | 1 | 12 | 1.0 | 0.8 | 11 | 0.1 | 1 |
| *CD2992* | CD630_29920 | hypothetical protein | 23 | 3.2 | 6.0x10^-4^ | 4 | -0.5 | 1 | 5 | 0.3 | 1 | 4 | 0.1 | 1 | 4 | -0.7 | 1 |
| *CD2069* | CD630_20690 | molybdopterin cofactor biosynthesis protein | 23 | 2.5 | 5.1x10^-4^ | 11 | 1.1 | 0.5 | 10 | 0.9 | 0.8 | 9 | 1.2 | 0.8 | 14 | 1.4 | 0.3 |
| *CD0341* | CD630_03410 | hypothetical protein | 21 | 6.7 | 7.4x10^-5^ | 1 | 2.2 | 0.9 | 2 | 2.6 | 0.7 | 1 | 2.0 | 1 | 1 | 1.7 | 1 |
| *uxaA* | CD630_30020 | D-galactate dehydratase/Altronate hydrolase | 21 | 2.7 | 1.3x10^-3^ | 6 | 0.2 | 1 | 13 | 1.9 | 0.03 | 7 | 0.9 | 0.9 | 7 | 0.3 | 1 |
| *CD0594* | CD630_05940 | hypothetical protein | 20 | 2.0 | 0.01 | 12 | 0.9 | 0.7 | 7 | -0.3 | 1 | 9 | 0.8 | 0.9 | 10 | 0.4 | 1 |
| *CD1037* | CD630_10370 | hypothetical protein | 19 | 2.3 | 1.7x10^-3^ | 10 | 1.0 | 0.6 | 8 | 0.4 | 1 | 9 | 1.1 | 0.8 | 10 | 0.7 | 1 |
| *CD0917* | CD630_09170 | recombination protein Bet | 17 | 3.1 | 3.6x10^-4^ | 4 | 0.1 | 1 | 3 | -1.6 | 0.8 | 3 | -0.5 | 1 | 4 | -0.6 | 1 |
| *CD0293* | CD630_02930 | ABC transporter bacitracin/multidrug-family ATP-binding protein | 16 | 2.0 | 0.02 | 14 | 1.6 | 0.1 | 13 | 1.5 | 0.1 | 8 | 0.8 | 0.9 | 12 | 1.1 | 0.6 |
| *CD3272* | CD630_32720 | membrane protein | 15 | 2.2 | 0.01 | 7 | 0.5 | 1 | 5 | -0.4 | 1 | 5 | 0.1 | 1 | 7 | 0.4 | 1 |
| *CD2417* | CD630_24170 | PTS system glucitol/sorbitol-specific transporter subunit IIB | 14 | 3.9 | 1.4x10^-5^ | 2 | -0.8 | 1 | 2 | -0.3 | 1 | 2 | 0.6 | 1 | 2 | -0.2 | 1 |
| *CD0283* | CD630_02830 | PTS operon transcription antiterminator | 14 | 2.2 | 7.2x10^-3^ | 5 | 0.0 | 1 | 4 | -0.6 | 1 | 5 | 0.5 | 1 | 6 | 0.2 | 1 |
| *CD0294* | CD630_02940 | ABC transporter bacitracin/multidrug-family permease | 13 | 2.1 | 0.01 | 12 | 1.9 | 0.05 | 14 | 2.2 | 4.8x10^-3^ | 6 | 1.0 | 0.8 | 12 | 1.6 | 0.2 |
| *CD3271* | CD630_32710 | hypothetical protein | 13 | 2.3 | 0.01 | 5 | 0.4 | 1 | 4 | -0.1 | 1 | 4 | 0.1 | 1 | 6 | 0.3 | 1 |
| *feoA* | CD630_17451 | ferrous iron transport protein | 13 | 2.3 | 0.05 | 10 | 1.9 | 0.2 | 9 | 1.6 | 0.3 | 14 | 2.8 | 5.2x10^-3^ | 11 | 1.9 | 0.2 |
| *tRNA-Phe* | CD630_t0630 | tRNA | 11 | 2.2 | 0.03 | 5 | 0.4 | 1 | 5 | 0.5 | 1 | 5 | 0.9 | 0.9 | 6 | 0.6 | 1 |
| *CD2022* | CD630_20220 | hypothetical protein | 11 | 2.1 | 0.05 | 5 | 0.2 | 1 | 6 | 0.9 | 0.8 | 4 | 0.6 | 1 | 13 | 2.2 | 0.03 |
| *CD2305* | CD630_23050 | pilin protein | 11 | +Inf | 2.2x10^-7^ | 1 | +Inf | 0.8 | 0 | +Inf | 1 | 2 | +Inf | 0.3 | 2 | +Inf | 0.6 |
| *CD1716* | CD630_17160 | permease | 10 | 2.9 | 2.8x10^-3^ | 1 | -2.2 | 0.8 | 1 | -2.7 | 0.7 | 1 | -1.5 | 1 | 2 | -1.8 | 1 |
| *kdgT2* | CD630_30040 | 2-keto-3-deoxygluconate permease | 9 | 3.6 | 1.0x10^-3^ | 2 | 0.8 | 1 | 9 | 3.5 | 7.6x10^-4^ | 2 | 0.7 | 1 | 3 | 1.4 | 1 |
| *CD0918* | CD630_09180 | hypothetical protein | 9 | 3.5 | 7.9x10^-4^ | 2 | 0.8 | 1 | 2 | 0.3 | 1 | 2 | 1.0 | 1 | 1 | -0.8 | 1 |
| *CD1717* | CD630_17170 | [hypothetical protein](#RANGE!_ENREF_8) | 9 | 2.8 | 6.6x10^-3^ | 2 | -1.2 | 1 | 2 | -0.5 | 1 | 1 | –Inf | 0.8 | 2 | -2.4 | 0.9 |
| *CD3114* | CD630_31140 | membrane protein | 8 | 3.7 | 1.6x10^-3^ | 1 | -1.2 | 1 | 1 | 0.4 | 1 | 2 | 1.9 | 1 | 1 | -1.2 | 1 |
| *CD3594* | CD630_35940 | hypothetical protein | 7 | 3.4 | 0.01 | 2 | 1.6 | 0.8 | 2 | 0.7 | 1 | 3 | 2.3 | 0.6 | 3 | 1.6 | 0.9 |
| *CD0908* | CD630_09080 | hypothetical protein | 6 | +Inf | 5.8x10^-4^ | 1 | +Inf | 0.9 | 1 | +Inf | 0.9 | 1 | +Inf | 0.7 | 0 | +Inf | 1 |
| *bioB* | CD630_02970 | biotin synthase | 5 | 3.8 | 0.02 | 2 | 2.4 | 0.7 | 3 | 2.6 | 0.5 | 2 | 2.2 | 0.9 | 2 | 2.4 | 0.9 |

BM refers to base mean, the mean of the counts after they were divided by the size factors to adjust for different sequencing depths. This value is the mean for the sample relative to wild type. log_2_FC denotes log_2_fold-change. A positive value indicates that the gene was up-regulated in the *spo0A^–^* mutant relative to wild type. Inf+ indicates that no transcript was detected in wild type relative to the *spo0A^–^* mutant..
