# Supplementary material for: Global Analysis of the Sporulation Pathway of Clostridium difficile
Source: PLoS Genet. 2013 Aug 8;9(8):e1003660. doi: 10.1371/journal.pgen.1003660 (PMC3738446; doi:10.1371/journal.pgen.1003660)
Supplement: Text S1 — Sigma factor regulation network topology in C. difficile. (DOCX) [file pgen.1003660.s022.docx]

**Text S1.**

**Network analysis of sporulation sigma factor regulatory pathway topology.**

Given the importance of the conclusion that the expression data does not support a linear network topology we felt it appropriate to consider alternative statistical methods. This analysis is presented as supplementary material rather than in the main body because we did not want its technical nature to detract from the simplicity and clarity of our central result.

***Background.*** Consider a collection of four genes (circles shown in Figure S4) $\{s0, e, g, k\}$, each of which has two states with respect to expression $\left\{ \downarrow,\uparrow\right\}$. Consider also ordered pairs of genes (edges), for example $\left( s0,e \right)$, interpreted to mean that if the first gene $(s0$) is $\uparrow$ then as a direct^[[1]](#footnote-1)^ consequence the second gene ($e$) also is $\uparrow$. The network topology proposed for *B. subtilis* is shown in Figure S4A.

Consider another gene, *q* (for query), that is $\uparrow$ during sporulation in the wildtype strain, but otherwise $\downarrow$, and that we wish to attach to the network. Finally, consider a set of four mutants, each obtained by knocking out (that is, setting the state to $\downarrow$ for) one gene of $\{s0, e, g, k\}$. The expression level of *q* in each of the mutant strains (*spo0A^–^, sigE^–^, sigG^–^*, and *sigK^–^*) provides information that allows us to attach Q to the network. A novel result of this work is that the topology proposed for *B. subtilis* does not account for the observed expression pattern of many genes ($\uparrow$ in the *sigE^–^* mutant but $\downarrow$ in the *sigG^–^* mutant, inconsistent with an (*sigE^–^, sigG^–^*) edge (Figure S4A-B). Other topologies are consistent with this expression pattern (Figure S4A).

A primary goal of this work is to identify genes genome-wide that are increased in expression during sporulation in a sporulation sigma factor-dependent manner (that is, identify a network topology and assign genes to it). In the main body of the paper this was accomplished with pairwise comparisons. For example, *q* was identified as a σ^G^-dependent gene (Figure S4C) if it was highly expressed (with high statistical significance) in wild type compared with the *sigG^–^* mutant. *q* was identified as a σ^E^-dependent gene (Figure S4D) if it was highly expressed in wild type compared with the *sigE^–^* mutant but not compared with the *sigK^–^* mutant.

**Rejecting the linear topology.**

The linear topology (any topology such as Figure S4 that has one root, one leaf and internal nodes having one inbound and one outbound edge) can be rejected if we find statistical evidence for genes that partition the strains according to the $S0G:EKJ$ (σ^G^-dependent gene) (with *S*0, *G, E, K*, and *J* referring to *spo0A^–^, sigG^–^, sigE^–^, sigK^–^*, and JIR8094 (WT) but not $S0E:GKJ$ (σ^E^-dependent gene) (Figure S5) *as well as* genes that partition the strains according to $S0E:GKJ$ but not $S0G:EKJ$.

A graph having root $S0$, leaf $Q$, and additional vertices $\left\{ E,G,K \right\}$ defines a partition, $p$, of the *strains* $\left\{ S0,E,G,K,J \right\}$, namely, strain $S0$ and strains on the path from $S0$ to $Q$ are $\downarrow$ while all other stains are $\uparrow$. We employed network-based models

$$Y_{Q}=X_{p}\beta+\in$$

where the design matrix,$X^{p}$, is defined by the partition

$$X_{ie}^{p}=\left\{ \begin{matrix} 1 & i\in e \\ 0 & i\in e^{C} \end{matrix} \right.$$

where $e$ is one element of the partition ($\downarrow$ or $\uparrow$) and $e^{C}$ is its complement. We fit each gene to the null model (uniform expression across strains) as well as the alternatives $S0G:EKJ$ and $S0E:GKJ$. We derived a p-value and fold-change using analysis of variance. Comparison of p-values associated with alternative topologies (Figure S5) makes it clear that we can reject the linear topology.

1. ‘Direct’ refers to direct with respect to knockout studies on these four genes. We do not rule out the possibility that a gene not studied here mediates this ‘direct’ effect. [↑](#footnote-ref-1)
