# Supplementary material for: Global Analysis of the Sporulation Pathway of Clostridium difficile
Source: PLoS Genet. 2013 Aug 8;9(8):e1003660. doi: 10.1371/journal.pgen.1003660 (PMC3738446; doi:10.1371/journal.pgen.1003660)
Supplement: Text S2 — References found in Tables S3, S4, S5, S6, S7, S8, S9 and S10. (DOCX) [file pgen.1003660.s023.docx]

**Text S2. Supplementary Table References.**

1. Permpoonpattana P, Tolls E, Nadem R, Tan S, Brisson A, et al. (2011) Surface layers of *Clostridium difficile* endospores. J Bacteriol 193: 6461-6470.

2. Putnam EE, Nock AM, Lawley TD, Shen A (2013) SpoIVA and SipL are *Clostridium difficile* spore morphogenetic proteins. J Bacteriol 195: 1214-1225.

3. Feucht A, Evans L, Errington J (2003) Identification of sporulation genes by genome-wide analysis of the sigmaE regulon of *Bacillus subtilis*. Microbiology 149: 3023-3034.

4. Eichenberger P, Jensen ST, Conlon EM, van Ooij C, Silvaggi J, et al. (2003) The sigmaE regulon and the identification of additional sporulation genes in *Bacillus subtilis*. J Mol Biol 327: 945-972.

5. Galperin MY, Mekhedov SL, Puigbo P, Smirnov S, Wolf YI, et al. (2012) Genomic determinants of sporulation in Bacilli and Clostridia: towards the minimal set of sporulation-specific genes. Environ Microbiol 14: 2870-2890.

6. de Hoon M, Eichenberger P, Vitkup D (2010) Hierarchical evolution of the bacterial sporulation network. Current Biol : CB 20: 45.

7. Wang S, Setlow B, Conlon E, Lyon J, Imamura D, et al. (2006) The forespore line of gene expression in *Bacillus subtilis*. J Mol Biol 358: 16-37.

8. Lawley T, Croucher N, Yu L, Clare S, Sebaihia M, et al. (2009) Proteomic and genomic characterization of highly infectious *Clostridium difficile* 630 spores. J Bacteriol 191: 5377-5386.
